# A Proteomic View of an Important Human Pathogen – Towards the Quantification of the Entire *Staphylococcus aureus* Proteome

**DOI:** 10.1371/journal.pone.0008176

**Published:** 2009-12-04

**Authors:** Dörte Becher, Kristina Hempel, Susanne Sievers, Daniela Zühlke, Jan Pané-Farré, Andreas Otto, Stephan Fuchs, Dirk Albrecht, Jörg Bernhardt, Susanne Engelmann, Uwe Völker, Jan Maarten van Dijl, Michael Hecker

**Affiliations:** 1 Institute for Microbiology, Ernst-Moritz-Arndt-University Greifswald, Greifswald, Germany; 2 Interfaculty Institute for Genetics and Functional Genomics, Ernst-Moritz-Arndt-University Greifswald, Greifswald, Germany; 3 Department of Medical Microbiology, University Medical Center Groningen and University of Groningen, Groningen, The Netherlands; National Institutes of Health, United States of America

## Abstract

The genome sequence is the “blue-print of life,” but proteomics provides the link to the actual physiology of living cells. Because of their low complexity bacteria are excellent model systems to identify the entire protein assembly of a living organism. Here we show that the majority of proteins expressed in growing and non-growing cells of the human pathogen *Staphylococcus aureus* can be identified and even quantified by a metabolic labeling proteomic approach. *S. aureus* has been selected as model for this proteomic study, because it poses a major risk to our health care system by combining high pathogenicity with an increasing frequency of multiple antibiotic resistance, thus requiring the development of new anti-staphylococcal therapy strategies. Since such strategies will likely have to target extracellular and surface-exposed virulence factors as well as staphylococcal survival and adaptation capabilities, we decided to combine four subproteomic fractions: cytosolic proteins, membrane-bound proteins, cell surface-associated and extracellular proteins, to comprehensively cover the entire proteome of *S. aureus*. This quantitative proteomics approach integrating data ranging from gene expression to subcellular localization in growing and non-growing cells is a proof of principle for whole-cell physiological proteomics that can now be extended to address physiological questions in infection-relevant settings. Importantly, with more than 1700 identified proteins (and 1450 quantified proteins) corresponding to a coverage of about three-quarters of the expressed proteins, our model study represents the most comprehensive quantification of a bacterial proteome reported to date. It thus paves the way towards a new level in understanding of cell physiology and pathophysiology of *S. aureus* and related pathogenic bacteria, opening new avenues for infection-related research on this crucial pathogen.

## Introduction

*Staphylococcus aureus* is a Gram-positive human pathogen of increasing significance, mainly due to its high incidence and the increasing spread of antibiotic resistances. Multiple virulence factors allow *S. aureus* to cause a broad spectrum of infectious diseases, ranging from superficial abscesses of the skin to severe diseases such as endocarditis, osteomyelitis, toxic shock syndrome or sepsis. Furthermore, *S. aureus* is particularly important in healthcare settings, where it is causing up to 40% of nosocomial infections. Vancomycin and related antibiotics form the drugs of last resort against resistant strains. Therefore, the recent emergence of vancomycin/methicillin-resistant *S. aureus* strains represents a major threat for the health care system, requiring the development of new therapeutic options against *S. aureus* infections [1]. Since new therapeutic strategies could involve neutralization of virulence factors or direct elimination of *S. aureus* a better understanding of both cell physiology and pathophysiology is urgently required to successfully combat *S. aureus* infections. This crucial goal can be reached by a functional genomics approach which opens a new perspective in *S. aureus* research because cell physiology and pathogenicity can be analyzed at a genome-wide scale.

The release of the first complete bacterial genome sequence in 1995 opened the era of functional genomics. In the same year, the term proteome was coined to describe the complete set of proteins synthesized under specific physiological circumstances [2]. Whereas the genome sequence only provides the “blue-print of life”, proteomics is required to bring this “blue print of life” to cell function, because proteins can be regarded as the main players of life. On the other hand the genome sequence is necessary to identify all cellular proteins expressed at genome-wide scale by mass spectrometry. Clearly, the route from the genome sequence via transcriptomics, proteomics and metabolomics towards a systems biology perspective leads to a new quality in understanding life in general. Because of their low complexity - only a few hundred different proteins make a cell viable - bacteria are extremely attractive model systems to bring the genome sequence to real life.

First proteomics studies date back to 1975 when O'Farrell published the two-dimensional gel electrophoresis technique that allows the separation of thousands of protein spots in an area of 20×20 cm in size [3]. After the initial enthusiasm for gel-based proteomics up to the early nineties, it became evident that only parts of a bacterial proteome can be visualized by this method. Many proteins escape detection by gel-based proteomics, hydrophobic membrane proteins and low abundance proteins being the most prominent classes. The implementation of mass spectrometry of peptides with mild ionization methods and online LC-MS/MS provides a suitable alternative to gel-based proteomics that has seen spectacular advances over the past 10 years, providing a completely new quality in proteomic research [4]–[6].

Comprehensive proteomic coverage has recently been reported for the non-pathogenic eukaryotic model organism *Saccharomyces cerevisiae* [7]. Mann and co-workers were the first to show that a combination of state of the art proteomics consisting of highly sensitive mass spectrometry, *in vivo* labeling with stable isotopes and appropriate bioinformatic analysis, allows the identification and even comparative quantification of almost the entire proteome of exponentially growing haploid and diploid *S. cerevisiae* cells.

Here we show that almost the entire proteome of *S. aureus* COL, whose genome sequence became available in 2005 [8], can be identified and even quantified by a combination of 2-D gel-based and LC-MS/MS-based proteomic techniques. Since therapeutic strategies directed against *S. aureus* could either target extracellular and secreted proteins or the pathogens metabolism, we integrated the analysis of the different subproteomes to provide evidence that an integrated proteomic view of cell physiology and virulence will help to come to a new quality in the understanding of general cellular processes of *S. aureus*. This approach would thus generate the prerequisites for a deeper understanding of the behaviour of this pathogen in its human host. The proteomic view of metabolism and virulence of *S. aureus* presented here constitutes to our knowledge the most comprehensive quantitative study of a bacterial proteome to date and opens a new dimension in infection-related research of this crucial pathogen.

## Results and Discussion

**Figure 1:**
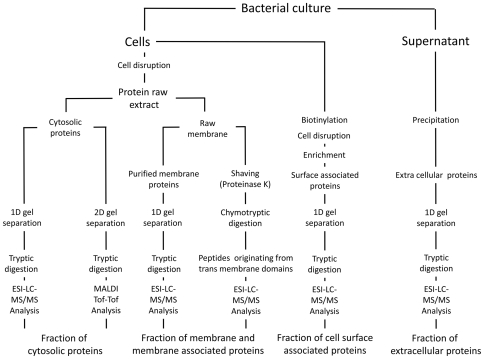
Analysis strategy for investigation of all sub-proteomic fractions of *S. aureus*. The mixture of the differentially labeled cells has been carried out on different levels. For analysis of the cytosolic proteins and membrane proteins by LC-MS/MS the samples were mixed on protein level using aliquots with the same protein amount. For 2-D gel analysis identical amounts of protein were separated on individual gel. For the fraction of cell surface-associated proteins the cells were mixed prior to the biotinylation step in equal amounts. Extracellular proteins were mixed after the precipitation step.

In order to visualize the entire proteome of *S. aureus*, we started with the hypothesis that roughly 2000 proteins are being synthesized from the genome that contains about 2600 genes [8]. This assumption is supported by our transcriptional profiling studies. Using DNA microarray techniques from about 2400 genes spotted on the DNA array about 1975 (82%) genes were found to be expressed above background level in growing and 1111 (46%) genes in stationary-phase cells (see Text S1, Table S1). Thus, approximately two thousand genes are expressed in total in both conditions corresponding to an expression level of 83% (see Table S1). In order to visualize the entire proteome we intentionally aimed at a combination of different subproteomic fractions (Figure 1) instead of a comprehensive analysis of one single fraction. Most of the virulence factors that are in the focus of infection-related proteomic studies of *S. aureus* are either cell surface-exposed or even secreted into the extracellular space. Combined analysis of different subproteomes would allow simultaneous coverage of infection-relevant immunogenic, surface-exposed and extracellular proteins besides the intracellular proteins that are required for basic metabolism and other cellular processes. Furthermore, such a pre-fractionation would also reveal detailed information about the subproteomic distribution of proteins. Therefore, four particular proteomic fractions, namely the cytosolic, membrane-bound, cell surface-associated and extracellular subproteomes were analyzed in order to define and assemble the entire proteome.

Even if the visualization of the protein inventory is the main topic of this project physiological studies require protein expression profiling and quantification to follow the up- and down-regulation of single proteins in response to physiological stimuli. In this study exponentially growing cells were compared to non-growing cells of the stationary phase as a proof of principle, because a shift from growing to non-growing conditions is probably a typical situation in most of the natural microhabitats in the human host.

### Cytosolic Proteins

To initiate the analysis of cytosolic proteins we first employed gel-based proteomics. Gel-based proteomics techniques have been extensively used to characterize the regulation of gene expression and metabolism of *S. aureus* in response to infection-related stimuli such as oxygen starvation [9], different kinds of stress such as oxidative stress [10], and were also used to assign the involved proteins to regulons [11], the building blocks of the genetic adaptation network. Two-dimensional gel electrophoresis (2-D PAGE) is still a powerful tool to address physiological issues, in particular because most of the metabolic pathways and the most obvious stress/starvation responses are visualized by gel-based proteomics in a convenient, eye-catching and intuitive way (“metabolism at a glance” [12], [13]).

**Figure 2:**
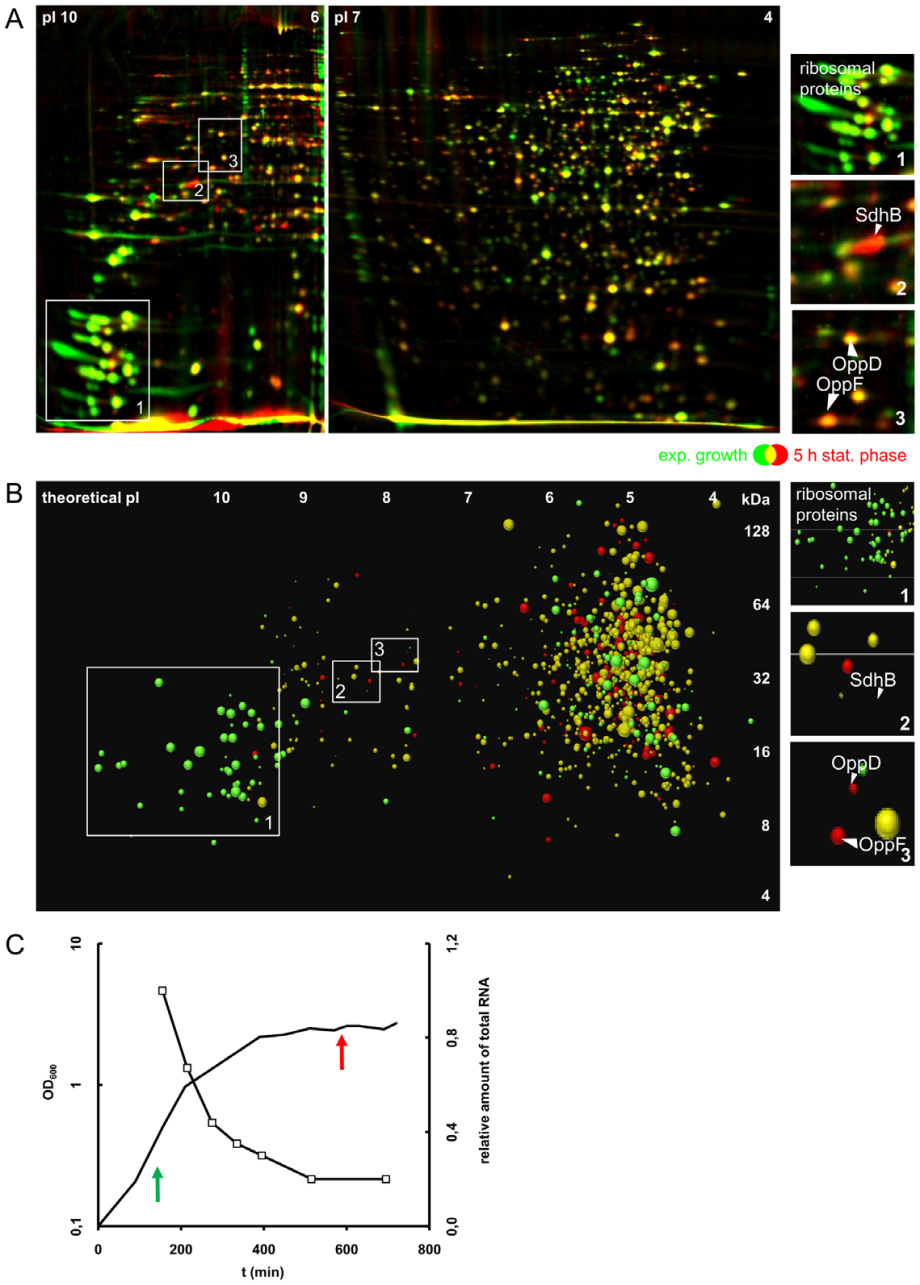
Changes of protein expression pattern. Framed sections are presented separately on right side of the figures. **A 2-D gel-based expression analysis by Dual Channel Imaging:** The overlay of the false color image of growing cells (green) and non-growing cells (red) is shown. This overlay results in the following color code: I. proteins probably no longer synthesized in non-growing cells but still present and more or less stable (yellow color), II. proteins no longer synthesized and even degraded in non-growing cells (green color) and III. proteins enriched in non-growing cells (red labelled). **B GeLC-MS/MS-based expression analysis:** Spot position derived from theoretical pI and theoretical molecular weight, spot size derived from spectral counts determined by Census software, spot color derived from log2 ratio of stat/exp, (green<−0.8, red>+0.8, yellow≥−0.8, ≤+0.8), Figure has been created in Microsoft Excel. **C Growth curve and RNA content:** Sampling points (arrows) are indicated in the growth curve (line graph). The relative decrease of total RNA content with decreasing growth rate is shown in the same graph (line with squares).

For this study we analyzed the proteome pattern in growing and non-growing cells cultivated in Bioexpress medium in the analytical windows of pI 4–7 and for the first time also of pI 6–11. In total 683 proteins were identified (Figure 2, Figure S1) covering most of the metabolic pathways (Figure S2). Different patterns of cytosolic proteins that respond to the transition from growth to stationary phase can be visualized and easily be quantified by image analysis.

The data indicate a dramatic reprogramming of protein synthesis in the non-growing state with many vegetative proteins repressed (e.g. by the stringent response) and others strongly induced (red labeled in Figure 2A) forming a complex adaptational protein network in order to face starvation and stress. A comprehensive stress/starvation proteomics signature library [14] is a useful toolbox for prediction of the physiological state of cells [15], [16], which will be important for physiological studies as for defining the main nutrients (metabolic proteome signatures) and the predominant stress/starvation stimuli of *S. aureus* cells under infection-relevant conditions [for related studies see 17], [18].

Because 2-D gels cover only about 25% of the proteome, 1-D gel-based protein prefractionation in combination with LC-MS/MS (GeLC-MS/MS) was employed to detect the missing proteins, and to identify and quantify the majority of expressed proteins. Using this technique, more than 1100 proteins were identified in the cytosolic fraction of growing and non-growing cells, most of them having a predicted pI in the range of 3.5 to 7.5 (Figure 2B).

**Figure 3:**
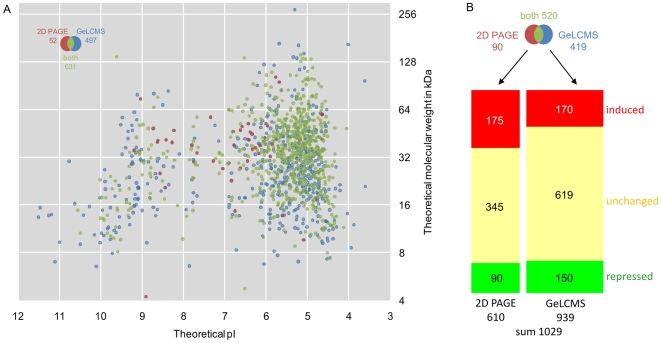
Comparative analysis of the cytosolic protein fraction. **A Qualitative analysis:** In summary 1180 proteins were identified, including 1095 proteins predicted as cytosolic proteins (but also proteins predicted to be cell wall-associated - 13, membrane proteins – 29, extracellular proteins - 23 and lipoproteins -20). Thus, 61% of the predicted cytosolic proteins were identified. **B Quantitative analysis:** 1029 proteins have been quantified in summary in both approaches with an overlap of 520 proteins. With the 2-D gel-based approach 610 proteins could be quantified and 265 proteins were found altered in amount (175 proteins - labeled in red - in higher anount and 90 proteins - labeled in green - in lower amount in the stationary phaset). By GeLC-MS 939 proteins could be quantified and 320 proteins were changed in quantity (170 proteins - labeled in red - in higher and 150 proteins - labeled in green - in lower amount in the stationary phase). Therefore, about one third of the cytosolic proteome has to be considered as regulated.

**Figure 4:**
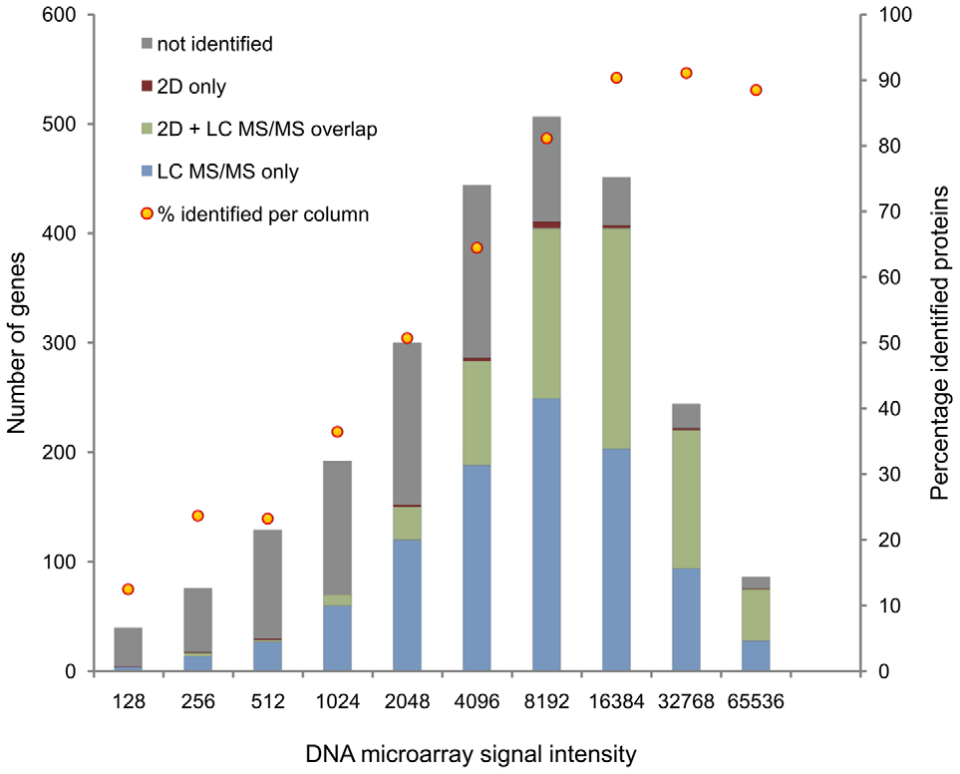
Comparison of identified proteins depending on the expression strength on RNA level. Up to 90% of the gene products of strongly expressed genes (definition of expressed genes – cf. Text S1) are identified, whereas low abundance proteins are clearly underrepresented among the identified proteins.

Besides the proteins already covered by 2-D PAGE, about 500 new proteins were identified by the GeLC-MS/MS approach, mostly low abundant proteins, but also very acidic or alkaline, as well as very large or small proteins (Figure 3). In total, a proteomic coverage of about 67% was achieved from 1796 predicted cytosolic proteins. Notably, the proteomic coverage of highly expressed genes is up to 90% while for low-expressed genes the coverage is lower (Figure 4).

Importantly, the dynamics of protein synthesis in growing and non-growing cells can be followed and compared by integrating 2-D gel-based and GeLC-MS/MS-based approaches. In Figure 2B, the quantitative GeLC-MS/MS data are combined with the experimental 2-D gel data (Figure 2A) in a virtual 2-D gel, using the same color code for up- and down-regulated proteins. Importantly, the two approaches revealed similar levels of up- or down-regulation of proteins in non-growing cells (Figure 3, Table S2).

The color code used in Figure 2 visualizes three major classes of proteins:

- yellow labeled proteins probably no longer synthesized in non-growing cells but still present and more or less stable
- green labeled proteins no longer synthesized and even degraded in non-growing cells and
- red labeled proteins enriched in non-growing cells due to increased synthesis

Transcription of class I and class II proteins have been analyzed by Northern blotting for a few typical candidate genes (Table S3, Figure S3). In non-growing cells the transcription of the genes encoding “yellow and green proteins” was repressed at similar levels at the onset of stationary phase, leading to a shut off of the new synthesis of the proteins. These proteins, however, already synthesized in growing cells and still present in non-growing cells, have two different fates: Either they are stable and kept at the same level as in growing cells or they are present in lower levels than in growing cells strongly indicating degradation. The reason for this different behavior- either stable or degraded- might be a reflection of the activity of the proteins. Those proteins that are still active might be stable (yellow proteins) whereas the proteins no longer active and thus not required might be degraded (green proteins), thus at least partially relieving nutrient starvation. We suggest that such unemployed proteins synthesized and required in growing cells are no longer active and integrated in functional complexes in non-growing cells, which makes them vulnerable to proteolytic attack, for example by Clp proteases [unpublished data, see also 19]. Ribosomal proteins, translational factors and some key enzymes for amino acid biosynthesis are the most obvious members of this group of “green proteins” (Figure 2). The molecular mechanisms that make the unemployed proteins accessible to the Clp machine have to be studied in future experiments.

This recycling of inactive proteins is probably a major nutrient resource for non-growing cells starved for carbon and energy sources. Particularly the ribosomes synthesized in high amounts in growing and partly inactive in non-growing cells form a major endogenous nutrient source. This degradation of ribosomes is observed at the RNA and protein level (Figure 2, Figure S4). The recycling of ribosomes is probably a crucial component of the physiology of starving cell [20].

The proteins particularly synthesized in the stationary phase (class III, red labeled proteins in Figure 2) will likely have adaptive functions in the non-growing state, among them specific starvation proteins that may help to make new substrates available (e.g. carbon overflow products) and even specific and general stress proteins to make the non-growing cells more stress resistant than growing cells [21]. Among the “red proteins” we found e.g. the PEP carboxykinase (PckA), a gluconeogenetic enzyme indicating the absence of glucose, some TCA cycle enzymes indicating carbon source limitation in non-growing cells and proteins belonging to the phosphate regulon (e.g. PhoP, SACOL1424) which suggests phosphate starvation. These proteomic signatures are useful tools for a general evaluation of cell physiology and metabolism (detailed information see Table S2, Figure S4).

**Figure 5:**
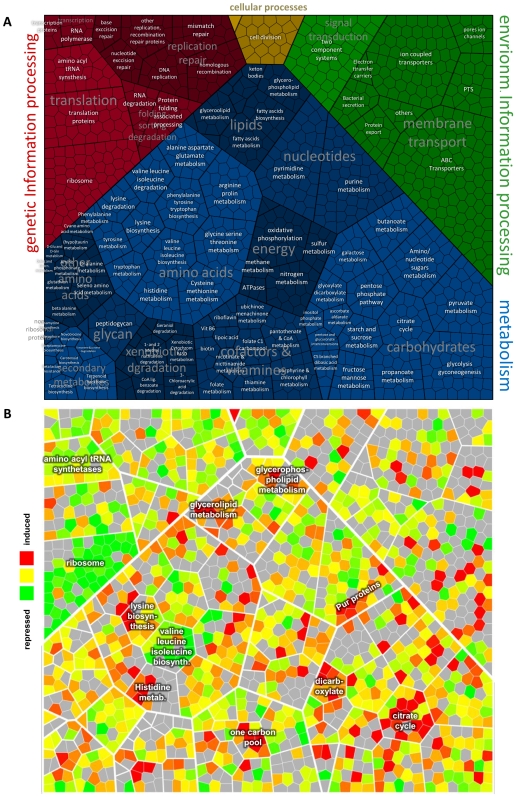
Coverage of gene functional categories. The coverage of gene functional categories was displayed by using the KEGG orthology of *S. aureus* COL which was mapped in a Voronoi treemap layout [38]. Treemaps display hierarchically organized information by using a space filling approach according to their hierarchy level (A). Smallest blue mosaic tiles correspond to proteins belonging to metabolic pathways (glycolysis, TCA etc.) which depict the metabolic brunches (e.g. carbohydrates, nucleotides) and which belong to metabolism at all. Changes in protein amount caused by starvation are indicated in shade of green (reduced level) and red (increased level). Proteins labeled in grey were not quantified. (B). Homogeneously regulated clusters have been indicated.

A Voronoi treemap-based layout of KEGG's *S. aureus* COL gene orthology was used to intuitively visualize the changes in the protein expression pattern in non-growing cells compared to growing cells. The up- (red color) and down-regulation (green color) of most of the proteins assigned to functional categories can be visualized which is illustrated for the already mentioned down-regulation of the translational machines such as ribosomal proteins or aminoacyl-tRNA-synthetases in non-growing cells. Additional pronounced changes in different branches of metabolism are highlighted in Figure 5.

### Membrane Proteins

**Figure 6:**
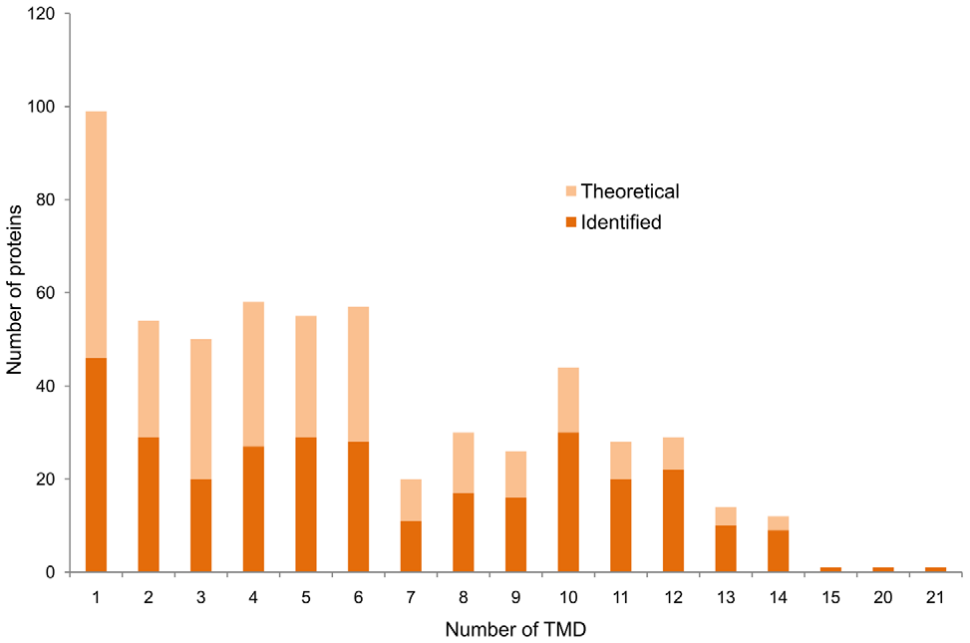
Numbers of theoretical and identified integral membrane proteins. Comparison of identified integral membrane proteins with the numbers of theoretical membrane proteins considering predicted trans-membrane domains (TMDs). This comparison indicates that there was no bias against proteins with a higher number of TMDs.

The membrane proteome was analyzed by a combination of a GeLC-MS/MS [21] and a shaving approach [22]. Whereas the first approach also identified a large fraction of cytosolic proteins - either membrane-associated or cytosolic contaminations - membrane shaving only revealed proteins with 1 to 21 trans-membrane domains. It is interesting to note that only by the shaving approach the most hydrophobic species of membrane-spanning peptides, usually lacking lysine- and arginine residues as targets for tryptic digestion, were identified. Employing a combination of both approaches, 56% of all predicted membrane proteins could be detected (Figure 6). The coverage is even higher if we consider that not all membrane proteins were expressed under the physiological conditions tested in this study. In addition to integral membrane proteins, the membrane-targeted analysis resulted in the identification of 125 proteins with a cytoplasmic location, but temporary or permanent membrane association. It should be noted that this number represents only an estimate of peripheral membrane proteins. Nevertheless, this group of membrane-associated proteins is important, due to their essential roles in processes such as membrane and cell wall metabolism, signal transduction and respiration.

**Figure 7:**
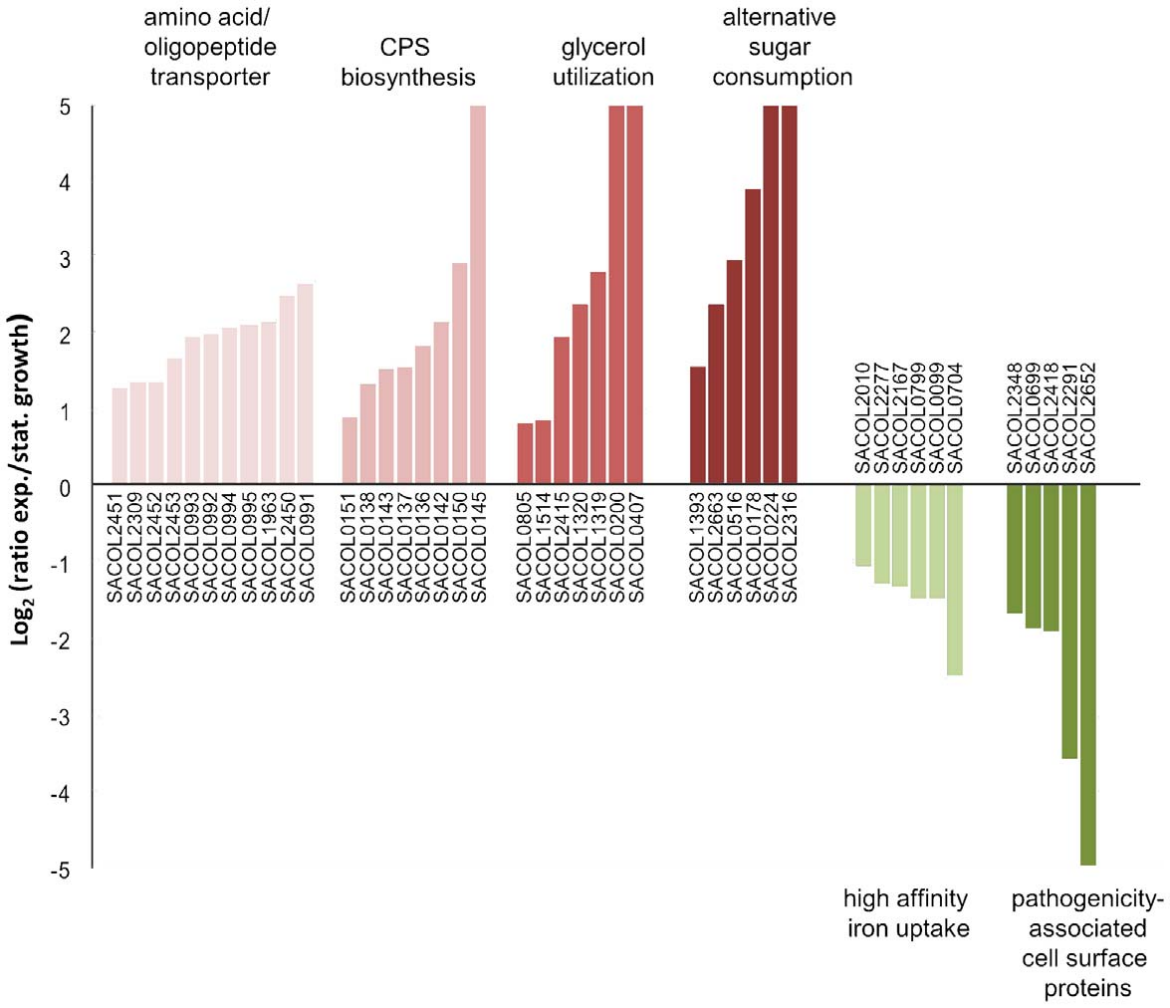
Membrane proteins which were found in altered amount. Several of the membrane proteins increased in amount in stationary phase are involved in the transport of amino acids or oligopeptides [from left to right: SACOL2451, SACOL2309, SACOL2452, SACOL2453, SACOL0993 (OppD), SACOL0992 (OppC), SACOL0994 (OppF), SACOL0995, SACOL1963, SACOL2450, SACOL0991 (OppB)], CPS (capsular polysaccharide) biosynthesis [SACOL0151 (Cap5P), SACOL0138 (Cap5C), SACOL0143 (Cap5H), SACOL0137 (Cap5B), SACOL0136 (Cap5A), SACOL0142 (Cap5G), SACOL0150 (Cap5O), SACOL0145 (Cap5J), glycerol utilization [SACOL0805, SACOL1514 (GpsA), SACOL2415 (Gpm), SACOL1320 (GlpK), SACOL1319 (GlpF), SACOL0200, SACOL0407 (GlpT)], or consumption of alternative sugars [SACOL1393 (GlcT), SACOL2663, SACOL0516, SACOL0178, SACOL0224, SACOL2316]. Of the proteins down-regulated in stationary phase numerous could be assigned to the category of iron uptake facilitation [SACOL2010, SACOL2277, SACOL2167, SACOL0799, SACOL0099 (SirA), SACOL0704] or to the class of cell surface proteins which are associated to pathogenicity of *S. aureus* [SACOL2348, SACOL0699 (Pbp4), SACOL2418 (Sbi), SACOL2291 (SsaA2), SACOL2652 (ClfB)]. Proteins indicated with a log2 ratio of 5 and -5 represent “on”/“off” proteins, respectively.

In this study the membrane proteome of *S. aureus* was quantified for the first time. Several integral- and membrane-associated proteins, such as the fructose-specific enzyme II (EII), four further EII components of unknown substrate specificity of the phosphotransferase system (PTS) and the glycerol uptake facilitator GlpF were up-regulated in non-growing cells due to carbon source limitation (Figure 7). Besides up-regulation of GlpF, two further transporters importing C_3_ carbon sources, namely, phosphoglycerate and glycerol-3-phosphate and four cytosolic enzymes acting downstream of C_3_ catabolite uptake were detected in increased quantity in the membrane fraction. This finding indicates that the glycerol consuming pathway forms enzyme complexes associated with the membrane bound uptake facilitator. Glycerol consumption was tightly connected to virulence in two close relatives of *S. aureus*, the intracellular pathogens *Listeria monocytogenes* [23], [24] and *Mycoplasma pneumonia* [25]. Such observations justify further research to obtain a better understanding of glycerol utilization in *S. aureus* and its potential involvement in pathogenicity. Furthermore, 11 transporters, mostly of the ATP binding cassette (ABC) type, involved in the uptake of amino acids and oligopeptides were also up-regulated. The strong upregulation of many transport proteins is a result of glucose starvation in non-growing cells.

Among the proteins showing decreased quantities, the high affinity iron compound ABC transporters were most striking (Figure 7). Five of the six down-regulated proteins represent lipoproteins that are responsible for binding of iron substrates. However, the physiological significance of this finding remains to be elucidated [26].

In general the transition from exponential growth to stationary phase is associated with broad quantitative changes in the membrane proteome involving both protein induction and degradation in non-growing cells (30% of the proteins covered being regulated). Nevertheless, there are proteome facets which seem to remain constant in quantity throughout different phases of growth. These include the Sec machinery for protein translocation, which serves in membrane protein biogenesis and protein secretion during all growth stages [27]. Summarizing this part, the present study documents the highest quantitative coverage of a bacterial membrane proteome to date.

### Cell Surface-Associated and Extracellular Proteins

From an infection-related point of view, secreted proteins still bound to the cell surface or secreted into the extracellular space form the most crucial class of proteins of *S. aureus*, because most of the virulence factors can be found in these two subproteomic fractions.

The group of cell surface-exposed proteins contains, besides membrane proteins with extended extracellular loops, also lipid-modified proteins, proteins covalently coupled to peptidoglycan by sortase, and cell wall-associated proteins not covalently bound to cell wall components [28]. To define this class of proteins, a biotinylation approach was employed with a reagent that is unable to enter the cell, thus strongly favouring the biotinylation of surface-exposed protein domains. After affinity purification on NeutrAvidin columns biotinylated proteins were enriched, identified and quantified. By this technique146 cell surface-associated proteins were identified, among them 48 membrane proteins, 4 proteins covalently attached to peptidoglycan, 37 lipoproteins and 57 cell wall-associated proteins containing a signal peptide, and various cytosolic contaminants. Some of these cell wall-associated proteins were also found in the extracellular space, indicating that this subgroup of secreted proteins is only loosely associated with the cell surface. Lipoproteins and membrane proteins with peripheral loops were found in the membrane fraction as well as in the biotinylated fraction as expected (Figure S5). Surface adhesins such as the fibronectin-binding proteins (Fnbps) [29] and the fibrinogen-binding protein ClfB [30] are preferentially synthesized during growth, whereas ClfA is higher expressed in non-growing cells [31]. Judged from the quantitative biotinylation approach ClfB showed a six times decreased level whereas the ClfA level increased more than three times in non-growing cells compared to growing cells. Other components like the immunodominant protein IsaB [32], [33] were present in more than tenfold increased amount on the surface of cells in stationary phase (Figure S6). Furthermore, there are also many changes in the level and pattern of membrane and lipoproteins covered by this biotinylation approach. Again, this is the highest coverage of identified and quantified cell surface-exposed proteins published so far.

Following virulence protein production along the growth curve, we observed a shift from the synthesis of certain cell surface-anchored proteins in growing cells, which may facilitate host cell adhesion, biofilm formation or even cell invasion [1] to the synthesis of extracellular proteins in the stationary phase. These extracellular proteins are virulence factors, such as toxins and superantigens, but also enzymes which are required for cell spreading. The analysis of the secretome is the method of choice for covering the entity of extracellular virulence factors which are extremely variable in different *S. aureus* strains. Usually, gel-based approaches guarantee a high coverage of this fraction [34], but gel-free approaches yield protein identification that overlap with those from gel-based approaches and also allow detection of additional extracellular proteins. In this study we used a quantitative GeLC-MS/MS approach and identified 57 extracellular proteins particularly in the stationary phase (Figure S5). It is relevant to note that not all proteins characterized by an N-terminal signal sequence and signal peptidase I cleavage site will be secreted to the extracellular space. The existence of so called Sec-attached proteins which are retained at the cytoplasmic membrane by still unknown mechanisms was discovered recently [35]. Unfortunately, it is still difficult to predict the proteins that belong to this class. Importantly, the present panorama view of the *S. aureus* secretome can now be used to address crucial issues, such as the assignment of secreted proteins to virulence regulons, or assessment of the virulence potential of well-defined clinical isolates.

### A Proteomics and Transcriptomic Comparison

**Figure 8:**
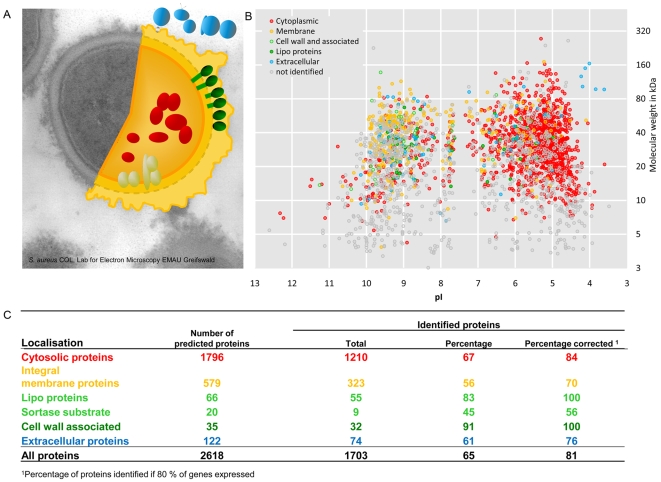
Summary of identified proteins. This summary considers the predicted localization in the cell considering the expected gene expression. **A**: Electron microscopy picture of *S. aureus* COL with illustrated subcellular protein fractions. **B**: Identified proteins colored according to the predicted localization in the cell displayed in a theoretical 2-D gel. **C**: Summary of identified proteins in relation to expected gene expression. Software tools used for the prediction of protein localization are given in the full methods section (Text S1).

Using the genes that were detected as expressed above background level in our transcriptional profiling study as a baseline, the products of about 80% of the expressed genes were identified at the proteome level (Figure 8). Almost all proteins were detected for highly expressed genes, whereas this proportion covered by proteomics decreases with decreasing gene expression level (see Figure 4). These results indicate that some proteins, in most cases probably low-abundance proteins, are still missing in our protein inventory of cells because of the low expression rate of the corresponding genes. Furthermore, we found that gene products present at very low level were exclusively detected by LC/MS-MS-techniques compared to gel-based proteomics reinforcing the statement of their higher sensitivity. In growing cells there is a good correlation between transcriptomics and proteomics. This is no longer true for non-growing cells mainly because many proteins synthesized in growing cells are still present but no longer synthesized.

### Conclusion and Outlook

Our progress on the way towards the entire proteome of *S. aureus* becomes evident from the combined subproteomic data (Figure 8). In total a coverage of about 80% of the expressed genes was obtained. Notably, proteins were identified with an average sequence coverage of 35%, although a much lower coverage would have been sufficient to identify a protein with high confidence.

Besides detailed information on the expression of proteins, reliable information about the localization of almost all proteins in the cell was gained. Several proteins were exclusively identified in one of the four subproteomic fractions indicating their localization in the cytosol or targeting to the cytosolic membrane, cell wall or even to the extracellular milieu. For proteins of still unknown function this information is of special interest as it provides clues for the identification of their cellular role. An interesting example is SACOL 1373, a protein of unknown function that remains membrane-associated during growth, but is secreted into the medium during stationary phase.

The current study does not only provide the protein inventory of *S. aureus* cells, but also quantitative data on the majority of proteins expressed in growing compared to non-growing cells in all four subproteomic fractions. A list containing all data on up- and down-regulation of the entire protein inventory, including information on subcellular localization of proteins, is presented in Table S2. The clustering of major protein groups is indicated by the color code used in Figure 2. In many cases proteins are either down- (green) or up-regulated (red). On the other hand there are also many proteins probably no longer synthesized but stable in non-growing cells (yellow). This quantitative proteomics approach comparing growing *vs*. non-growing cells is only a starting point, and the huge amount of proteome data requires many follow-up studies in order to understand the protein dynamics in a physiological context. Furthermore, this approach can now be extended to many other physiological issues, infection-related ones included. The future challenge will now be to transfer this huge amount of proteome data to new hypotheses which will finally generate new physiological knowledge.

This progress in bacterial proteomics will have a great impact on future studies on cell physiology of *S. aureus* and related Gram-positive bacteria, and it will thus dramatically improve our knowledge about the behavior of these pathogens in the human host. First of all protein expression profiling in response to environmental stimuli can now be accomplished with a high proteomic coverage to identify almost all proteins that make the cell viable. Furthermore, the virulence protein inventory of a cell can be captured, analyzed, and quantified. Protein expression profiling, however, is only the first step towards the understanding of life processes. Using a combination of gel-based and gel-free proteomics the final destination and fate of each single protein can be followed at a proteome-wide scale [13]. Detailed studies on the dynamics of the membrane proteome, on the phosphoproteome and its role in signal transduction, on the damage, repair and degradation of proteins or on the protein interaction network will provide a new quality in understanding cell physiology and pathophysiology of *S. aureus*, thereby opening a new era in infection-related research.

## Materials and Methods

*S. aureus* COL was grown in ^15^N-labeled or unlabeled BioExpress® 1000 medium (Cambridge Isotope Laboratories, Andova, MA, USA.) under vigorous agitation at 37°C. Cells were harvested at an OD_600_ of 0.5 to sample exponentially growing cells or five hours after the cells completely reached stationary phase (OD_600_ about 4). Cells were disrupted and protein raw extracts of cytosolic and membrane proteins were prepared as described [9], [22]. Extracts of extracellular proteins were prepared by TCA precipitation at 4°C over night. For preparation of extracts of surface-associated proteins, cells were treated with Sulfo-NHS-SS-Biotin prior to disruption. Biotinylated proteins were enriched by NeutrAvidin agarose affinity purification. Protein raw extracts of labeled and unlabeled cells were either analyzed separately by 2-D gel image analysis or 1∶1 mixed and further separated via 1-D PAGE (Figure 1). Proteins separated in 2-D PAGE were identified via MALDI-ToF/MS after in-gel digestion [36] using a Proteome Analyzer 4800 (Applied Biosystems, Foster City, CA) and subsequent MASCOT search. Peptides originated from in-gel digestion of 1-D gels were separated by RP chromatography using a nanoACQUITY^TM^ UPLC^TM^ System (Waters, Milford, MA). Separated peptides were online subjected to MS/MS analysis in a LTQ-Orbitrap (Thermo Fisher Scientific, Waltham, MA, USA), which was run in data-dependent mode. Resulting spectra were searched against a target-decoy database using SEQUEST (version 27 rev. 12). The database was composed of all protein sequences of *S. aureus* COL extracted from the National Center for Biotechnology Information (NCBI) bacteria genomes plus common contaminants and an appended set of the reversed sequences created by Bioworks Browser. Quantitative data were obtained implementing the Census software tool [37].

**Full Methods** and any associated references are available as Text S1.

## Supporting Information

Text S1 Supplementary materials and methods.
(0.09 MB DOC)

Figure S1 Reference 2-D map of cytoplasmic proteins of S. aureus COL grown in Bioexpress 1000 (Cambridge Isotope Laboratories, Andova, MA, USA) medium in the pI range 4–7. Cells were harvested at OD600 of 0.5 to sample exponentially growing cells. In this reference map 553 proteins were sketched, represented in 732 spots. It has to be considered that 428 proteins appear in single spots whereas 125 proteins are distributed in at least two spots, indicating post-translational modifications.
(3.87 MB TIF)

Figure S2 Identified proteins in the 2-D map (pI 4–7 and 6–10) involved in the main metabolic pathways. Green labeled proteins were identified whereas yellow labeled proteins were not identified in the 2-D gel based approach.
(4.69 MB TIF)

Figure S3 Northern blot analysis of selected genes. Total RNA was extracted from S. aureus COL cells cultured in BioExpress® medium at different time points during growth and stationary phase. Equal amounts of RNA were separated by denaturating RNA gel electrophoresis and blotted onto positively charged nylon membranes. The membranes were hybridized with digoxigenin-labeled RNA probes for the respective genes. The diagrams show the quantified Northern blot signal intensities bevor (open squars) and after scaling (closed squars) for the decrease in total RNA content. The schematic representations of the gene loci are based on the sequence of S. aureus COL. Major transcripts representing the predicted transcriptional organization of the operons based on the Northern blot analyses are shown as arrows. Dual channel false color images show the results from 2-D gel-based expression analysis for the genes analyzed by Northern blot: growing cells (green) and non-growing cells (red).
(1.26 MB TIF)

Figure S4 Correlation between the protein ratios of biological replicates used to obtain the quantitative data. The calculated correlation was 0.84. Selected regulated functional groups are highlighted.
(0.26 MB TIF)

Figure S5 Regulation of proteins identified in the fraction of cell surface-associated and extracellular proteins.
(1.46 MB TIF)

Figure S6 Regulated cell surface and cell wall-associated proteins. Class I proteins are colored in yellow, class II proteins in green and class III proteins in red. 1-SACOL2652 (ClfB), 2-SACOL1940, 3-SACOL2348, 4-SACOL1725 (RplT), 5-SACOL1066 (Fmt), 6-SACOL1825, 7-SACOL0938 (DltD), 8-SACOL0712, 9-SACOL2383, 10-SACOL1687, 11-SACOL0551, 12-SACOL1111, 13-SACOL1895, 14-SACOL1028 (HtrA), 15-SACOL0021 (YycH), 16-SACOL0539 (PurR), 17-SACOL0189, 18-SACOL1522 (EbpS), 19-SACOL1989, 20-SACOL0022 (YycI), 21-SACOL1140 (IsdA), 22-SACOL0610 (SdrE), 23-SACOL1788, 24-SACOL1836, 25-SACOL0968 (SpsA), 26-SACOL1514 (GpsA), 27-SACOL1168 (Efb), 28-SACOL2549, 29-SACOL0050 (Pls), 30-SACOL2019 (SdrH), 31-SACOL0856 (ClfA), 32-SACOL1062 (Atl), 33-SACOL2002 (Map), 34-SACOL0024, 35-SACOL1847, 36-SACOL2660 (IsaB).
(0.65 MB TIF)

Table S1 Signal intensities and calculated ratios from DNA microarray experiment.
(0.32 MB PDF)

Table S2 Identified proteins with corresponding quantitative value.
(0.29 MB PDF)

Table S3 Oligonucleotides used in this study.
(0.01 MB PDF)
