# Supplementary material for: A Proteomic View of an Important Human Pathogen – Towards the Quantification of the Entire Staphylococcus aureus Proteome
Source: PLoS One. 2009 Dec 4;4(12):e8176. doi: 10.1371/journal.pone.0008176 (PMC2781549; doi:10.1371/journal.pone.0008176)
Supplement: Text S1 — Supplementary materials and methods. (0.09 MB DOC) [file pone.0008176.s001.doc]

**Supplementary information**

**Materials and Methods**

**1. Growth Conditions**

*S. aureus* COL^1^ was grown in ^15^N-labelled or unlabelled BioExpress® 1000 (Cambridge Isotope Laboratories, Inc.) under vigorous agitation at 37 °C. Cells were harvested at an OD_600_ of 0.5 to sample exponentially growing cells or five hours after cells completely reached stationary phase (OD_600_ about 4).

**2. Proteome analysis based on 2-D gel electrophoresis**

**Preparation of protein extracts**

*For isolation of intracellular proteins* 80 ml of the bacterial culture were harvested. After centrifugation (10 min, 4 °C, 8,000 × g) the cell pellets were washed with and resolved in 1 ml TE buffer. The cells were added to 500 µl of glass beads and disrupted using the Precellys 24 homogenizator (PeqLab, Germany) for 30 s at 6,800 rpm. Cell debris and glass beads were separated from the proteins by centrifugation for 10 min at 4 °C at 20,000 × g followed by a second centrifugation step to remove insoluble and aggregated proteins (30 min, 4 °C, 20,000 × g).

*For preparation of extracellular proteins* 500 ml of exponential growth phase cultures and 250 ml of stationary phase cultures were centrifuged for 10 min at 4 °C and 8,000 × g. Proteins were precipitated by overnight incubation of the supernatant at 4 °C with 10% w/v TCA and collected by centrifugation for 1 hour at 4 °C and 8,000 × g. The pellet was washed 4 times with 70% ethanol, followed by a washing step with 100% ethanol. The protein extracts were dried and resolved in 1 ml 8 M urea/2 M thiourea. The proteins were again precipitated by overnight incubation with 4 volumes of 100% acetone at room temperature, harvested, washed twice with 80% acetone and once with 100% acetone, dried and again resolved in 500 µl 8 M urea/2 M thiourea.

The concentration of the protein extracts was determined using Roti Nanoquant (Roth, Germany).

**2-D gel electrophoresis and image analysis.**

*Isoelectric focussing (IEF)* was carried out using commercially available 18 cm IPG strips (GE Healthcare) in the pI ranges of 4-7 and 6-11 for cytosolic proteins and in the pI range of 3-10 for extracellular proteins. IPG strips were loaded with 80 µg protein extract in the case of pI 4-7 or 70 µg in the case of extracellular proteins by rehydratization for 18 h in a solution containing 8 M urea, 2 M thiourea, 1% w/v Chaps, 20 mM DTT and 0.1% v/v Pharmalyte 3-10. IPG strips with a pI range of 6-11 were incubated for 18 h with a solution containing 7 M urea, 2 M thiourea, 4% w/v Chaps, 2.5% DTT, 10% v/v isopropanol, 5% v/v glycerol, 2% v/v Pharmalyte 6-11. Samples for IEF with IPG strips 6-11 were prepared as follows: For enrichment of alkaline proteins 200 µg of crude protein extract were precipitated with 4 volumes of acetone overnight at -20°C. After centrifugation for 30 min at 20,000 × g and 4 °C the protein pellet was washed with 1 ml of acetone and dried. The pellet was resolved in 50 µl sample buffer (7 M urea, 2 M thiourea, 4% w/v Chaps) and incubated for 5 h at 26 °C. The protein extract was loaded directly on the IPG strips using sample cups at the anode^2^. Additionally, a paper strip soaked with 3.5% DTT was placed near the cathode^3^.

IEF was performed using the MultiPhor II unit (Amersham Biosciences) employing the following voltage profile: linear increase from 0 to 500 V for 1 Vh, linear increase from 500 V to 3500 V for 3000 Vh and a final phase of 3500 V for 23500 Vh. Isoelectric focussing of alkaline proteins was done by the focussing protocol published by Büttner and co-workers^4^, including a initial phase of 300 V for 60 min.

*For second dimension* PAGE was carried out as described previously^4^. The resulting gels were fixed with a solution containing 40% ethanol and 10% acetic acid, washed with distilled water and then stained overnight with the fluorescent dye Krypton (Thermo Scientific). After destaining with 5% acetic acid and two additional washing steps with distilled water the gels were scanned with a Typhoon 9400 (Amersham Biosciences) (excitation source: 532 nm laser, emission filter: 560 nm longpass).

Image analysis and spot quantitation was realised with the Delta2D software (Decodon GmbH, Germany)^5^.

**Protein identification by MALDI ToF and MALDI ToF/ToF MS.**

*Protein spots* were excised from the gel with the Ettan spot picker (Amersham Biosciences) with a picker head of 2 mm. In-gel digestion with sequencing grade trypsin (Promega, Madison, WI) and extraction of peptides were carried out with the Ettan Spot Handling Workstation (Amersham Biosciences) using a modified standard protocol^6^.

*The MALDI ToF measurement* was carried out on the 4800 MALDI ToF/ToF Analyzer (Applied Biosystems, USA). This instrument is designed for high throughput measurement, with the ability to measure the samples, calibrate the spectra and analyze the data using the 4000 Explorer™ Software V3.6 automatically.

The spectra were recorded in a mass range from 900 to 3700 Da with a focus mass of 2000 Da. For one main spectrum 25 sub-spectra with 100 shots per sub-spectrum were accumulated using a random search pattern. If the autolytical fragment of trypsin with the monoisotopic (M+H)+ m/z at 2211.104 reached a signal to noise (S/N) ratio of at least 10, an internal calibration was automatically performed as one-point-calibration using this peak. The standard mass deviation was less than 0.15 Da. If the automatic mode failed (in less than 1%) the calibration was carried out manually.

After calibration the peak lists were created by using the script of the GPS Explorer™ Software Version 3.6 (build 329) with the following settings: mass range from 900 to 3700 Da, a peak density of 20 peaks per 200 Da, a minimal area of 100 and maximal 65 peaks per spot. The peak list was created for signals with S/N ratios equal to or higher than 15.

The three strongest peaks in a ToF spectrum were selected for MS/MS experiments. For one main spectrum 20 sub-spectra with 125 shots per sub-spectrum were accumulated using a random search pattern. The internal calibration was automatically performed as one-point-calibration with the monoisotopic arginine (M+H)+ m/z at 175,119 or lysine (M+H)+ m/z at 147,107 reaching a (S/N) ratio of at least 10.

*The peak lists* were created by using the script of the GPS Explorer™ Software Version 3.6 (build 329) with the following settings: mass range from 60 to precursor - 20 Da, a peak density of 50 peaks per 200 Da, a minimal area of 100 and maximal 65 peaks per precursor. Peak lists were created for S/N ratios of at least 10.

*For database search* the Mascot search engine Version 2.1.04 (Matrix Science Ltd, London, UK) with a *Staphylococcus aureus* COL sequence database extracted from the National Center for Biotechnology Information (NCBI) bacteria genomes was used. Therefore one missed cleavage site and an RMS error < 20ppm were allowed. Oxidation of methionine and carbamidomethylation of cystein were considered as variable modifications. Proteins that yielded a Mowse score of at least 50 and two MS/MS in one experiment (or at least a score of 50 without MS/MS in two or more experiments) were regarded as positive identifications.

**3. Proteome analysis based on 1-D gel electrophoresis and gel-free methods**

**Preparation of protein extracts**

*For isolation of intracellular proteins* cells were harvested by centrifugation (8,000 × g) for 10 min at 4 °C. Cell pellets were washed twice with ice-cold TBS buffer (50 mM Tris, 150 mM NaCl, pH 8.0) and resuspended in lysis buffer (50 mM Tris, 1 mM PMSF, 1 × Nuclease Mix from Amersham Biosciences). Cell disruption by homogenization with glass beads was carried out in the Precellys 24 homogenizator (PeqLab, Germany) in three cycles of 30 s at 6,800 rpm with intermittent cooling. Cell lysates were kept at room temperature for 30 min to allow nucleolytic digestion before removal of cell debris by centrifugation (8,000 × g at 4 °C for 2 min). Supernatants were centrifuged another 10 min, and crude protein extracts were stored at -20 °C. Protein concentration was determined using Roti-Nanoquant (Roth, Karlsruhe, Germany). Equal amounts of differentially labelled proteins were combined for 1-D gel-based analysis.

*Membrane proteins* were purified from 100 mg of crude protein extract as described before^6^, leaving out an extraction of proteins by n-dodecyl-β-D-maltoside treatment. The purified membrane pellet was homogenized in 50 mM triethylammonium bicarbonate (TEAB) buffer, pH 7.8 before separation of 15 µg of protein by 1-D SDS PAGE (see below).

Additionally to 1-D SDS PAGE a *Proteinase K-Chymotrypsin Digestion approach for membrane proteins (Shaving Approach)* was applied. Therefore an aliquot of 80 mg of protein extract was pelleted by ultracentrifugation at 100,000 × g for 1 h. The crude membrane fraction was homogenized in 500 µl of carbonate buffer (200 mM Na_2_CO_3_, pH 11.0) and further treated and digested with proteinase K (Promega) as described before^7^. Briefly, the protein concentration of the homogenized pellet was adjusted to 1 mg/ml. The sample was incubated for 1 h on an overhead shaker (4 °C) and every 15 min homogenized using an insulin syringe. After the sample was brought to room temperature, solid urea was added to a concentration of 8 M. Protein reduction was performed at 50 °C in 5 mM tris(2-carboxyethyl)phosphine hydrochloride for 30 min followed by an alkylation step in 10 mM iodoacetamide in the dark (15 min at room temperature). Proteinase K was added in an enzyme:protein ratio of 1:50 followed by incubation for 15 h in a thermomixer (37 °C at 900 rpm). One volume of 10% acetonitrile in water was added before the solution was cooled on ice for 15 min. Following an ultracentrifugation step of 1 h (100,000 × g at 4 °C) the supernatant was discarded. To remove residual urea from the pellet, it was rinsed with 50 mM TEAB buffer, pH 7.8, and again ultracentrifuged (100,000 × g at 4 °C for 1 h). For chymotrypsin digestion the pellet was resuspended in 200 µl of digestion buffer [50 mM TEAB, pH 7.8, 10 mM CaCl_2_, and 0.5% RapiGest™ (Waters)] before 4 µg of the enzyme (emp Biotech, Berlin, Germany) were added. Digestion was carried out while shaking (900 rpm) at 30 °C for 6 h. To remove RapiGest™ concentrated HCl was added to give a 250 mM HCl solution (pH < 2) followed by incubation at 37 °C for 45 min. The sample was centrifuged three times for 15 min at 4 °C (20,000 × g), each time keeping the supernatant containing the peptides of chymotryptic digestion. The final supernatant was stored at -20 °C before nHPLC-MS/MS analysis.

*For preparation of cell surface protein fraction* cells were harvested at 4,000 × g for 5 minutes at 4 °C in exponential and stationary phase of growth and 1 g of cells (wet cell weight) were resuspended in 5 ml ice-cold PBS (pH 8.0) with 1 mM PMSF on ice. Equivalent OD units of exponentially grown cells in ^14^N-enriched medium and stationary cells grown in ^15^N-enriched medium were combined. For a biological replicate with label switch samples were combined vice versa. Biotinylation reaction was performed by adding fresh Sulfo-NHS-SS-Biotin (Pierce, Rockford, IL) to the intact cells. Therefore a 1% solution of Sulfo-NHS-SS-Biotin was prepared by adding 5 mg to 500 µl PBS (pH 8.0) directly before use. 100 µl of the biotinylation solution were added to 1 ml of reaction volume resulting in an end concentration of 1.5 mM Sulfo-NHS-SS-Biotin. Cells were incubated by gentle shaking for 2 h on ice. To stop the reaction and to remove non-reacted biotinylation reagent cells were centrifuged carefully at 4,000 × g for 5 min at 4 °C and washed three times with ice-cold PBS (pH 8.0) and 500 mM glycine. A pellet of 1 ml reaction volume was resuspended in 500 µl PBS (pH 8.0) with 1 mM PMSF on ice and transferred to a 1.5 ml tube containing glass beads (Sartorius). Disruption of cells was performed mechanically in a Precellys 24 homogenizator (PeqLab, Germany) at 6,800 rpm twice for 20 s. The lysate was centrifuged (20,000 × *g* for 30 min at 4 °C) and the biotinylated proteins were isolated and purified by NeutrAvidin agarose (Pierce, Rockford, IL).) affinity purification. For a reaction volume of 500 µl protein mixture 50 µl of NeutrAvidin agarose resin were washed twice with PBS (pH 8.0), 1% NP-40 and centrifuged 1,000 × g for 1 min at 4 °C. The washed resin was incubated with the protein lysate for 90 min gently shaking on ice. The supernatant was removed and the resin-bound complex washed 6 times with PBS (pH 8.0), 1% NP-40. Elution of the biotinylated proteins was performed by adding 20 µl of reductive SDS sample buffer containing 62.5 mM Tris/ HCl, pH 6.8, 2% SDS, 20% glycerol, 50 mM DTT and 5% β-mercaptoethanol. Eluted protein samples were separated by 1-D SDS PAGE (see below).

*For preparation of extracellular proteins* the protocol described above has been used (see section 2-D gel-based analysis) with the exception that differentially labelled proteins were combined in equal relation of protein amount. Extracellular protein samples were separated by 1-D PAGE (see below).

**Protein identification and quantification using LC-ESI MS/MS analysis**

*Proteins were separated by 1-D SDS PAGE* according to Laemmli^8^. Therefore 30 µg of protein was applied per lane. After staining with Coomassie Brilliant Blue R-250 the complete protein separation band was cut into 10 to 25 equal gel slices depending on protein amount. In-gel digestion was performed as described by Eymann *et al*.^6^.

*All peptides obtained from an in-gel digestion were separated by liquid chromatography* and measured online by ESI mass spectrometry. LC-MS/MS analyses were performed using a nanoACQUITY UPLC™ system (Waters) coupled to an LTQ Orbitrap™ mass spectrometer (Thermo Fisher Scientific,Waltham, MA) creating an electro spray by the application of 1.5 kV between Picotip™ Emitter (SilicaTip™, FS360-20-10 Coating P200P, New Objective) and transfer capillary. Peptides were loaded onto a trap column (nanoAcquity UPLC ^TM^ column, Symmetry® C18, 5 µm, 180 µm inner diameter x 20 mm, Waters) and washed 3 min with 99% buffer A (0.1% (v/v) acetic acid) with a flow rate of 10 µl/ min. Elution was performed onto an analytical column (nanoAcquity UPLC ^TM^ column, BEH130 C18 1.7 µm, 100 µm inner diameter x 100 mm, Waters) by a binary gradient of buffer A and B (100% (v/v) acetonitrile, 0.1% (v/v) acetic acid) over a period of 80 min with a flow rate of 400 nl/ min.

*Peptides derived from proteinase K-chymotrypsin digestion* were loaded directly via a 5 µl loop onto an analytical column (nanoACQUITY UPLC column, BEH130 C18, 1.7 µm, 100 µm × 100 mm; Waters) and washed for 37.5 min with 99% buffer A (0.1% acetic acid). Peptides were eluted from the column in a 5 h linear gradient going up to 60% buffer B (100% acetonitrile, 0.1% acetic acid). The flow rate during column loading and elution was set to 400 nl/min, and the analytical column was tempered at 60 °C^9^.

*For MS/MS analysis* a full survey scan in the Orbitrap (m/z 300–2000) with a resolution of 30,000 was followed by MS/MS experiments of the five most abundant precursor ions acquired in the LTQ via CID. Precursors were dynamically excluded for 30 s, and unassigned charge states as well as singly charged ions were rejected.

*For protein identification* *.dta files were generated from *.raw files using Bioworks Browser 3.3.1 SP1 (Thermo Fisher Scientific). Charge state deconvolution and deisotoping were not performed. All MS/MS samples were analyzed using SEQUEST version 28 (rev. 12) (Thermo Fisher Scientific), applying the following search parameters: peptide tolerance, 10 ppm; tolerance for fragment ions, 1 amu; b- and y-ion series; an oxidation of methionine (15.99 Da) and a carboxyamidomethylation (57.02 Da) of cysteine (for the shaving approach) were considered as variable modifications (maximal three modifications per peptide). Each *.raw file was searched twice, once without any static modification and a second time allowing for the substitution of all ^14^N with ^15^N atoms. For the two different digestion strategies (the second was used for the analysis of the membrane fraction via membrane shaving) varying settings for the enzyme had to be used. Samples of the in-gel tryptic digestion were searched with trypsin cutting fully enzymatically and allowing two missed cleavage sites. Proteins subjected to membrane shaving and subsequent chymotryptic digestion were searched without any enzyme specificity. All samples were searched against a target-decoy database that was composed of all protein sequences of *S. aureus* COL extracted from the National Center for Biotechnology Information (NCBI) bacteria genomes plus common contaminants and an appended set of the reversed sequences created by BioworksBrowser 3.3.1.SP1.

Resulting *.out files of each sample were combined using DTASelect 2.0^10^. To get the total number of protein identifications, the DTASelect files of one 1-D Gel experiment were combined with the help of Contrast^10^. Overall ^14^N and ^15^N DTASelect files were again merged with Contrast and filtered with DTASelect to obtain protein hits based on at least two peptides and a false discovery rate on peptide level lower than 1% (filter used: DTASelect %* -y 0 -c 2 -C 4 --here --decoy Reverse_ ‑p 2 -t 2 -u --MC 2 -i 0.3 --fp 0.005). The final DTASelect-filter file was adjusted to remove peptide hits of the same sequence (differently charged and post-translationally modified peptides count to be the same) employing a user-written script.

For the prediction of transmembrane domains the TMHMM 2.0 algorithm was used^11^. Signal sequences were estimated by neural networks and a hidden Markov model of the software tool SignalP 3.0^12, 13^. Only when both the presence of a signal sequence and a cleavage site were presumed, and the signal peptide overlapped with a TMD to more than half of the latter’s length, the TMD was considered to be false-predicted and subtracted. Proteins were considered to be lipo-anchored if projected by DOLOP^14^  or Augur^15^. If proteins exhibited an LPXTG- or NPQTN motif they were regarded as sortase substrates covalently bound to the cell wall. Further, cell wall-associated proteins were defined according to previous findings^16, 17^ and PSORT^18^. When proteins with predicted signal peptides but no hint of cell wall location could not be identified from the extracellular medium but via the biotinylation surface protein approach they were regarded as new cell wall -associated proteins.

*For protein quantification* *.raw files had to be transferred to MS1 files (RawExtractor 1.9.3 by Tao Xu, Yates Laboratory at The Scripps Research Institute). The software tool Census^19^ used MS1 files and the adjusted final DTASelect-filter file (see *Protein Identification*) for protein quantification. Quantification results were exported to Excel implementing a determination factor of 0.7 to cull only good quality spectral data. In total two biological replicates with each having a technical parallel were performed, thereby allowing for label switching (1^st^ biological replicate – exponential growth phase = ^15^N; 2^nd^ biological replicate - stationary phase = ^15^N). A protein was considered reliably quantified when its ratio was determined in both biological replicates with not less than two peptides in at least one of the replicates. Normalization of protein ratios was carried out over the median of all log2 ratios. Proteins that have been identified, but were not quantified by Census were manually inspected for poor quality or presence in only one of the samples, ^14^N or ^15^N. If their presence in only one of the samples was based on more than one peptide in both biological replicates they were added to the list of quantified proteins as “on”/“off” proteins.

**4. Transcriptome analysis**

**RNA isolation**

*S. aureus* COL cells were harvested on ice-cold killing buffer (20 mM NaN_3_, 5 mM MgCl_2_, 20 mM Tris, pH 7.5) by centrifugation (9,000 x g, 5 min) at 4 °C and the cells were stored at -70 °C until processing. Total RNA was isolated from *S. aureus* COL using the acid-phenol method with modifications described by Fuchs *et al.*^20^. In addition, to eliminate traces of contaminating DNA, the RNA was DNase treated according to the protocol described in appendix E of the Qiagen RNeasy Mini Handbook (4th edition, April 2006). Cleanup of RNA from DNase digest was performed with the RNA Clean-Up and Concentration Micro Kit according to the manufacturer’s instructions (Norgen, Thorold, ON, Canada). The integrity of the RNA was checked with the Agilent 2100 Bioanalyzer (Agilent Technologies, Palo Alto, CA, USA) and the concentration and purity were assessed with a NanoDrop ND-1000 spectrophotometer (NanoDrop Technologies, Inc., Rockland, DE, USA).

**Northern blot analyses**

Samples for the Northern blot analyses were harvested at exponential growth (OD_600_ = 0.5) and six additional time points (one, two, three, four, six and nine hours) until cells had remained in stationary phase for five hours (OD_600_ about 4). The experiment was done in duplicate with one culture grown in ^14^N-labelled and the other in ^15^N-labelled BioExpress® medium. Northern blot analyses were done according to the protocol published by Wetzstein *et al.*^21^. Digoxigenin-labeled RNA probes were prepared by in vitro transcription with T7 RNA polymerase and appropriate PCR fragments as templates^22^. The PCR fragments were generated with chromosomal DNA isolated from *S. aureus* COL using the Wizard^TM^ Genomic DNA Purification Kit (Promega, Madison, WI) and the oligonucleotides listed in supplementary table 2. The digoxigenin-labeled RNA marker I (Roche, Indianapolis, IN) was used to estimate transcript sizes. The hybridization signals were detected using a Vilber Lourmat Fusion-SL-3500 WL (PeqLab, Germany) and analyzed using the software package Lumi-Analyst (Roche Diagnostics, Mannheim, Germany).

**DNA-microarray analysis**

The design and evaluation of the customized StaphChip oligoarray manufactured by Agilent Technologies (Palo Alto, CA, USA) used in this study has been described previously^23^. The current version of the 15K StaphChip covers the core genome and strain specific chromosomal regions of the eight *S. aureus* strains COL, N315, MRSA252, MSSA476, Mu50, MW2, NCTC8325, and USA300.

For the DNA-microarray experiment RNA was extracted from two independently grown cultures. One was grown in ^14^N-labelled BioExpress® medium and the other in ^15^N-labelled BioExpress medium. Samples of exponentially growing cells (OD_600_=0.5) and of cell at 5h after entry into stationary phase were harvested from every culture. Equal amounts of all RNAs were pooled and the pool was used as common reference (Cy5) for the four sample RNAs (Cy3). Thus, in total four hybridizations, one for each sample versus the common reference, were performed.

Synthesis of Cy5-dCTP or Cy3-dCTP (Perkin-Elmer) labelled cDNA was done with 10 µg of total RNA as template by direct reverse-transcription using Superscript II (Invitrogen) and random hexamers (Promega) as primers. After denaturation of the RNA primer mix for 10 minutes at 70°C the cDNA synthesis was performed in a 50 µl reaction volume at 42°C for 60 minutes. The concentrations of enzyme and reagents in the reverse transcription reaction were as follows: Superscript II (400 units), Cy-dye (1.25 nmol), dATP, dGTP, dTTP (5 nmol each), dCTP (2.5 nmol), random hexamers (1.25 µg), DTT (0.01 M) and 1x first strand buffer. The Superscript II was heat inactivated for 10 minutes at 70°C. For the degradation of the RNA after cDNA synthesis the samples were incubated for 30 min at room temperature with *E. coli* RNase H (2 units) (Invitrogen). Labelled cDNA was then purified with the CyScribe GFX Purification Kit following the instructions of the manufacturer (Amersham Biosciences). The Cy-dye incorporation was analysed with a NanoDrop ND-1000 spectrophotometer (NanoDrop Technologies, Inc., Rockland, DE). Approximately 300 ng of each labelled cDNA corresponding to 6 pmol of incorporated dye were used in two-colour pool-cDNA (Cy3) versus sample cDNA (Cy5) competitive hybridization experiments.

Hybridizations were done in a total sample volume of 40 µl for 17 hours at 65°C at 10 rpm in a dedicated hybridization chamber (Agilent) and hybridisation oven (Robbins Scientific, Sunnyvale, CA, USA). After hybridization the slides were washed for one minute at room temperature in wash buffer 1 followed by a one minute washing step at 37°C with wash buffer 2. The slides were dried by submersion in acetonitrile for 30 seconds. Blocking reagents, hybridization buffer (Gene Expression Hybridization Kit) and washing solutions (Gene Expression Wash Buffer Kit) were purchased from Agilent.

Slides were scanned at a 5 µm resolution (Agilent Technologies Scanner) and fluorescence intensities were extracted and processed using the Feature Extraction^TM^ software version 9.5.3.1 (Agilent). Local background-subtracted signals of both fluorescence channels were normalized with the linear LOWESS function. Intensity profiles for exponential growth and stationary phase samples were calculated with the ratio split pipeline as implemented in the Rosetta Resolver software package (Ceiba Solutions, US) using the pool-cDNA channel as a common reference. In order to compensate for the global repression of RNA synthesis, signal intensities from stationary phase were lineary scaled by a factor of 0.2 prior to ratio building (stationary phase signal/ exponential growth signal). The scaling factor was calculated based on the relative decrease of total RNA comparing exponentially growing cells and stationary phase cells. Total RNA was extracted from equal amounts of *S. aureus* COL cells grown in BioExpress® medium and harvested along the growth curve. Upon entry into stationary phase the amount of total RNA rapidly decreased down to a level corresponding to 20% of that found in exponentially growing cells. Signal intensities and calculated ratios from the DNA microarray experiment are summarized in supplementary table 3.

In order to estimate how much expression (signal intensity on the DNA microarray) is necessary to result in physiological amounts of protein we used the following assumption. A gene had to be among the 1.25% showing the highest induction or repression level. The signal intensity under non induced (for up regulated proteins) and repressed conditions (for down regulated proteins) should be a reasonable estimate for the minimum expression level. The median signal intensity was found to be around 1500 units following the above criteria. Using this cutoff we concidered about 80% of the genome to be expressed.

The microarray data are accessible through GEO Series accession number GSE15060 (<http://www.ncbi.nlm.nih.gov/geo/query/acc.cgi?acc=GSE15060>).

**References.**

1. Shafer, W.M., Iandolo, J.J. Genetics of staphylococcal enterotoxin B in methicillin-resistant isolates of *Staphylococcus aureus*. *Infect Immun*, **25**, 902–911(1979).

2. Görg, A., Obermaier, C., Boguth, G., Csordas, A., Diaz, J.J. *et al.* Very alkaline immobilized pH gradients for two-dimensional electrophoresis of ribosomal and nuclear proteins. *Electrophoresis,* **18,** 328-337 (1997).

3. Görg, A., Boguth, G., Obermaier, C., Posch, A., Weiss, W. Two-dimensional polyacrylamide gel electrophoresis with immobilized pH gradients in the first dimension (IPG-Dalt): The state of the art and the controversy of vertical versus horizontal systems. *Electrophoresis,* **16,** 1079-1086 (1995).

4. Büttner, K., Bernhardt, J., Scharf, C., Schmid, R., Mäder, U. *et al.* A comprehensive two-dimensional map of cytosolic proteins of *Bacillus subtilis*. *Electrophoresis,* **22,** 2908–2935 (2001).

5. Berth, M., Moser, F.M., Kolbe, M., Bernhardt, J. The state of the art in the analysis of two-dimensional gel electrophoresis images. *Appl Microbiol Biotechnol.* **6,** 1223-43 (2007).

6. Eymann, C., Dreisbach, A., Albrecht, D., Bernhardt, J., Becher, D. *et al.* A comprehensive proteome map of growing *Bacillus subtilis* cells. *Proteomics* **4**, 2849-2876 (2004).

7. Wolff, S., Otto, A., Albrecht, D., Zeng, J.S., Büttner, K. *et al.* Complementary analysis of the vegetative membrane proteome of the human pathogen *Staphylococcus aureus.* *Mol Cell Proteomics,* **7,** 1460-8 (2008).

8. Laemmli, U.K., Cleavage of structural proteins during the assembly of the head of bacteriophage T4. *Nature,* **227**, 680-5 (1970).

9. Speers, A.E., Blackler, A.R., Wu, C.C. *et al.* Shotgun analysis of integral membrane proteins facilitated by elevated temperature. *Anal Chem,* **79,** 4613-20 (2007).

10. Tabb, D.L., McDonald, W.H., Yates, J.R. 3rd. DTASelect and Contrast: tools for assembling and comparing protein identifications from shotgun proteomics. *J Proteome Res,* **1**, 21-6 (2002).

11. Krogh, A., Krogh, A., Larsson, B., von Heijne, G., Sonnhammer, E.L. Predicting transmembrane protein topology with a hidden Markov model: application to complete genomes. *J Mol Biol,* **305**, 567-80 (2001).

12. Nielsen, H., Krogh A. Prediction of signal peptides and signal anchors by a hidden Markov model. Proc *Int Conf Intell Syst Mol Biol,* **6**, 122-30 (1998).

13. Bendtsen, J.D., Nielsen, H., von Heijne, G., Brunak, S. *et al*. Improved prediction of signal peptides: SignalP 3.0. *J Mol Biol,* **340**, 783-95 (2004).

14. Madan Babu, M., Sankaran K., DOLOP - database of bacterial lipoproteins, *Bioinformatics,* **18**, 641-643 (2002).

15. Billion, A., Ghai, R., Chakraborty, T., Hain, T. Augur - a computational pipeline for whole genome microbial surface protein prediction and classification. *Bioinformatics,* **22**, 2819-20 (2006).

16. [Gill, S.R](http://www.ncbi.nlm.nih.gov/sites/entrez?Db=pubmed&Cmd=Search&Term="Gill SR"%5BAuthor%5D&itool=EntrezSystem2.PEntrez.Pubmed.Pubmed_ResultsPanel.Pubmed_DiscoveryPanel.Pubmed_RVAbstractPlus)., [Fouts, D.E., Archer, G.L., Mongodin, E.F., Deboy, R.T. *et al*.](http://www.ncbi.nlm.nih.gov/sites/entrez?Db=pubmed&Cmd=Search&Term="Fouts DE"%5BAuthor%5D&itool=EntrezSystem2.PEntrez.Pubmed.Pubmed_ResultsPanel.Pubmed_DiscoveryPanel.Pubmed_RVAbstractPlus) Insights on evolution of virulence and resistance from the complete genome analysis of an early methicillin-resistant *Staphylococcus aureus* strain and a biofilm-producing methicillin-resistant *Staphylococcus epidermidis* strain. *J Bacteriol* **187**, 2426-38 (2005).

17. [Clarke, S.R](http://www.ncbi.nlm.nih.gov/sites/entrez?Db=pubmed&Cmd=Search&Term="Clarke SR"%5BAuthor%5D&itool=EntrezSystem2.PEntrez.Pubmed.Pubmed_ResultsPanel.Pubmed_DiscoveryPanel.Pubmed_RVAbstractPlus)., Foster, S.J. Surface adhesins of *Staphylococcus aureus*. *Adv Microb Physiol,* **51**, 187-224 (2006).

18. [Nakai, K](http://www.ncbi.nlm.nih.gov/sites/entrez?Db=pubmed&Cmd=Search&Term="Nakai K"%5BAuthor%5D&itool=EntrezSystem2.PEntrez.Pubmed.Pubmed_ResultsPanel.Pubmed_DiscoveryPanel.Pubmed_RVAbstractPlus)., Horton, P. PSORT: a program for detecting sorting signals in proteins and predicting their subcellular localization. *Trends Biochem Sci,* **24**, 34-6 (1999).

19. Park, S.K., Venable, J.D., Xu, T., Yates, J.R. 3rd*.* A quantitative analysis software tool for mass spectrometry-based proteomics. *Nat Methods,* **5**, 319-22 (2008).

20. Fuchs, S., Pané-Farré, J., Kohler, C., Hecker, M., Engelmann, S. Anaerobic gene expression in *Staphylococcus aureus*. *J Bacteriol* **189**, 4275-89 (2007).

21. Wetzstein, M., Völker, U., Dedio, J., Löbau, S., Zuber, U., *et al.*. Cloning, sequencing, and molecular analysis of the *dnaK* locus from *Bacillus subtilis*. *J Bacteriol* **174**, 3300–10 (1992).

22. Ziebandt, A. K., Weber, H., Rudolph, J., Schmid, R., Höper, D., *et al*. Extracellular proteins of *Staphylococcus aureus* and the role of SarA and sigmaB. *Proteomics* **1**, 480–93 (2001).

23. Charbonnier, Y., Gettler, B., Francois, P., Bento, M., Renzoni, A., *et al.* A generic approach for the design of whole-genome oligoarrays, validated for genomotyping, deletion mapping and gene expression analysis on *Staphylococcus aureus*. *BMC Genomics* **6**, 95 (2005).
