# Supplementary figures and images for: A Proteomic View of an Important Human Pathogen – Towards the Quantification of the Entire Staphylococcus aureus Proteome
Source: PLoS One. 2009 Dec 4;4(12):e8176. doi: 10.1371/journal.pone.0008176 (PMC2781549; doi:10.1371/journal.pone.0008176)

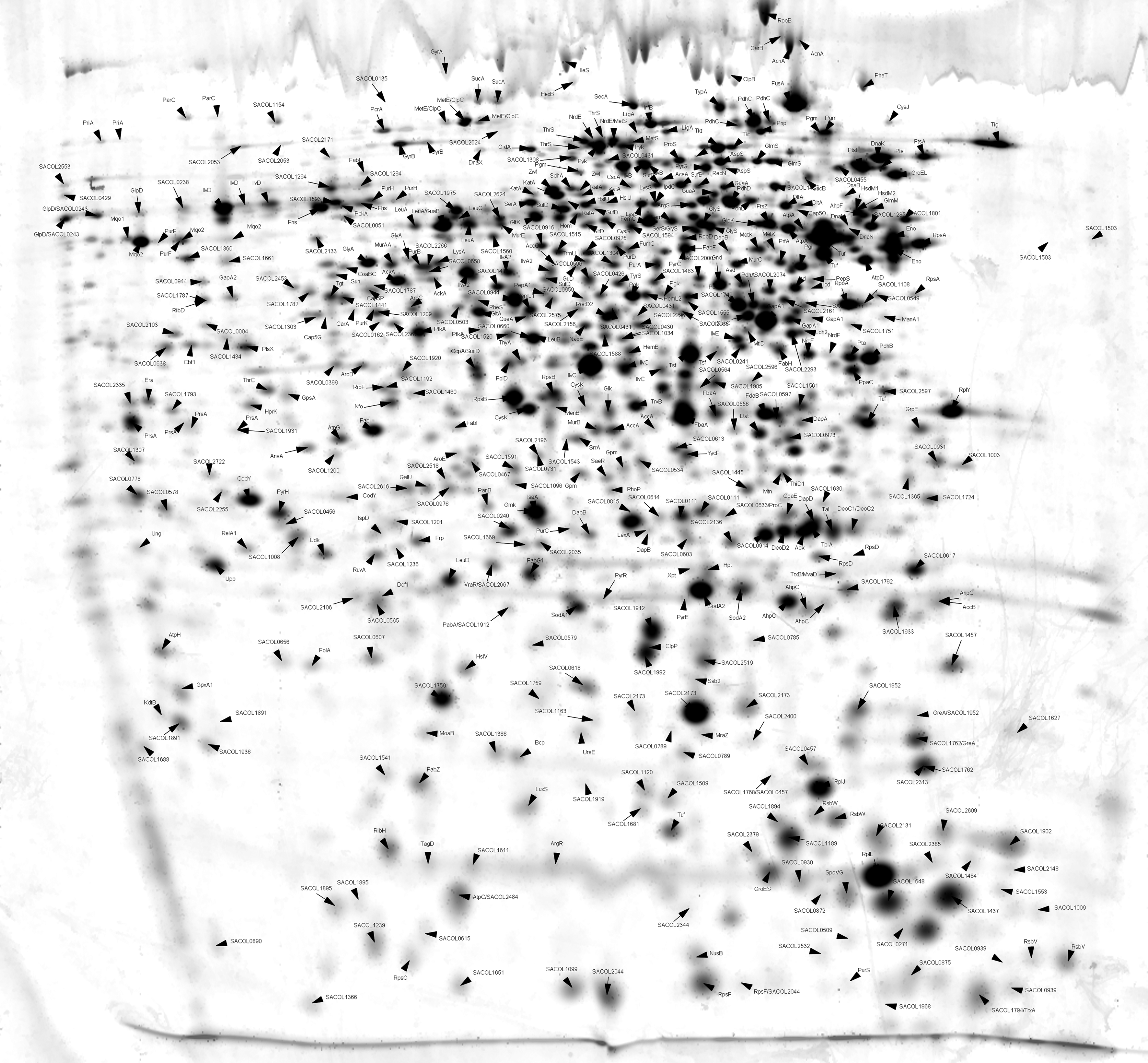

Supplement: Figure S1 — Reference 2-D map of cytoplasmic proteins of S. aureus COL grown in Bioexpress 1000 (Cambridge Isotope Laboratories, Andova, MA, USA) medium in the pI range 4–7. Cells were harvested at OD600 of 0.5 to sample exponentially growing cells. In this reference map 553 proteins were sketched, represented in 732 spots. It has to be considered that 428 proteins appear in single spots whereas 125 proteins are distributed in at least two spots, indicating post-translational modifications. (3.87 MB TIF) [file pone.0008176.s002.tif]

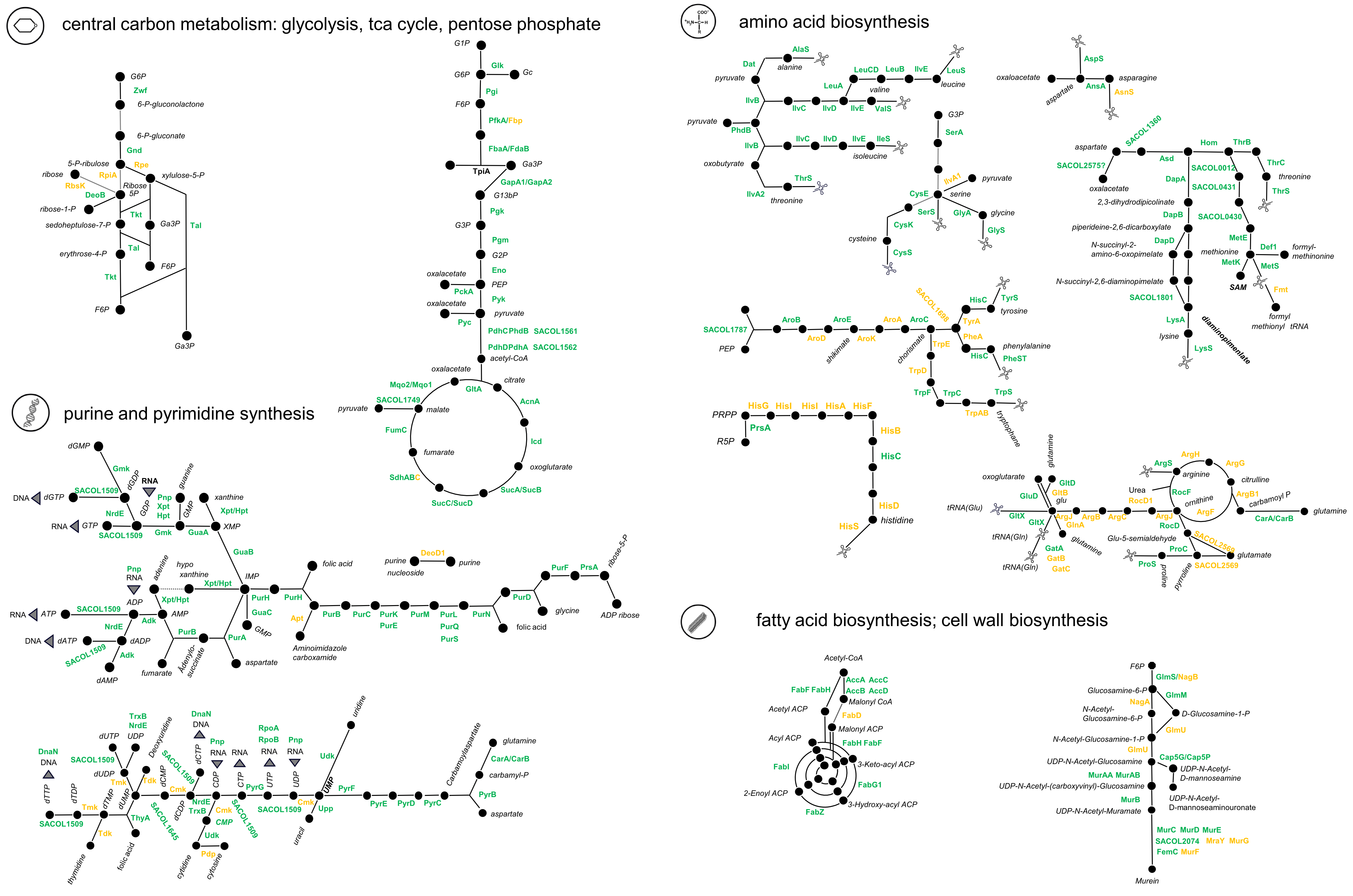

Supplement: Figure S2 — Identified proteins in the 2-D map (pI 4–7 and 6–10) involved in the main metabolic pathways. Green labeled proteins were identified whereas yellow labeled proteins were not identified in the 2-D gel based approach. (4.69 MB TIF) [file pone.0008176.s003.tif]

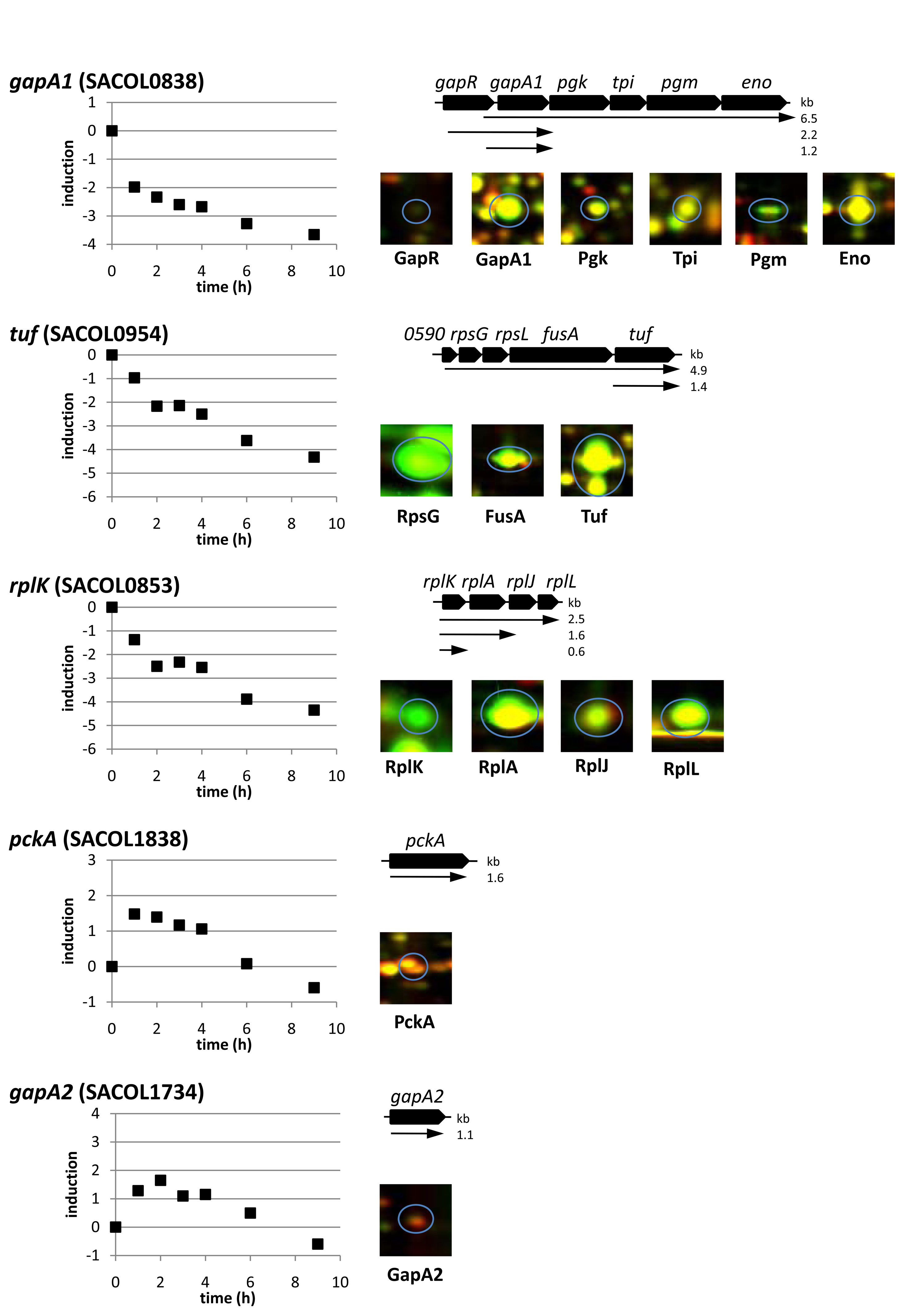

Supplement: Figure S3 — Northern blot analysis of selected genes. Total RNA was extracted from S. aureus COL cells cultured in BioExpress® medium at different time points during growth and stationary phase. Equal amounts of RNA were separated by denaturating RNA gel electrophoresis and blotted onto positively charged nylon membranes. The membranes were hybridized with digoxigenin-labeled RNA probes for the respective genes. The diagrams show the quantified Northern blot signal intensities bevor (open squars) and after scaling (closed squars) for the decrease in total RNA content. The schematic representations of the gene loci are based on the sequence of S. aureus COL. Major transcripts representing the predicted transcriptional organization of the operons based on the Northern blot analyses are shown as arrows. Dual channel false color images show the results from 2-D gel-based expression analysis for the genes analyzed by Northern blot: growing cells (green) and non-growing cells (red). (1.26 MB TIF) [file pone.0008176.s004.tif]

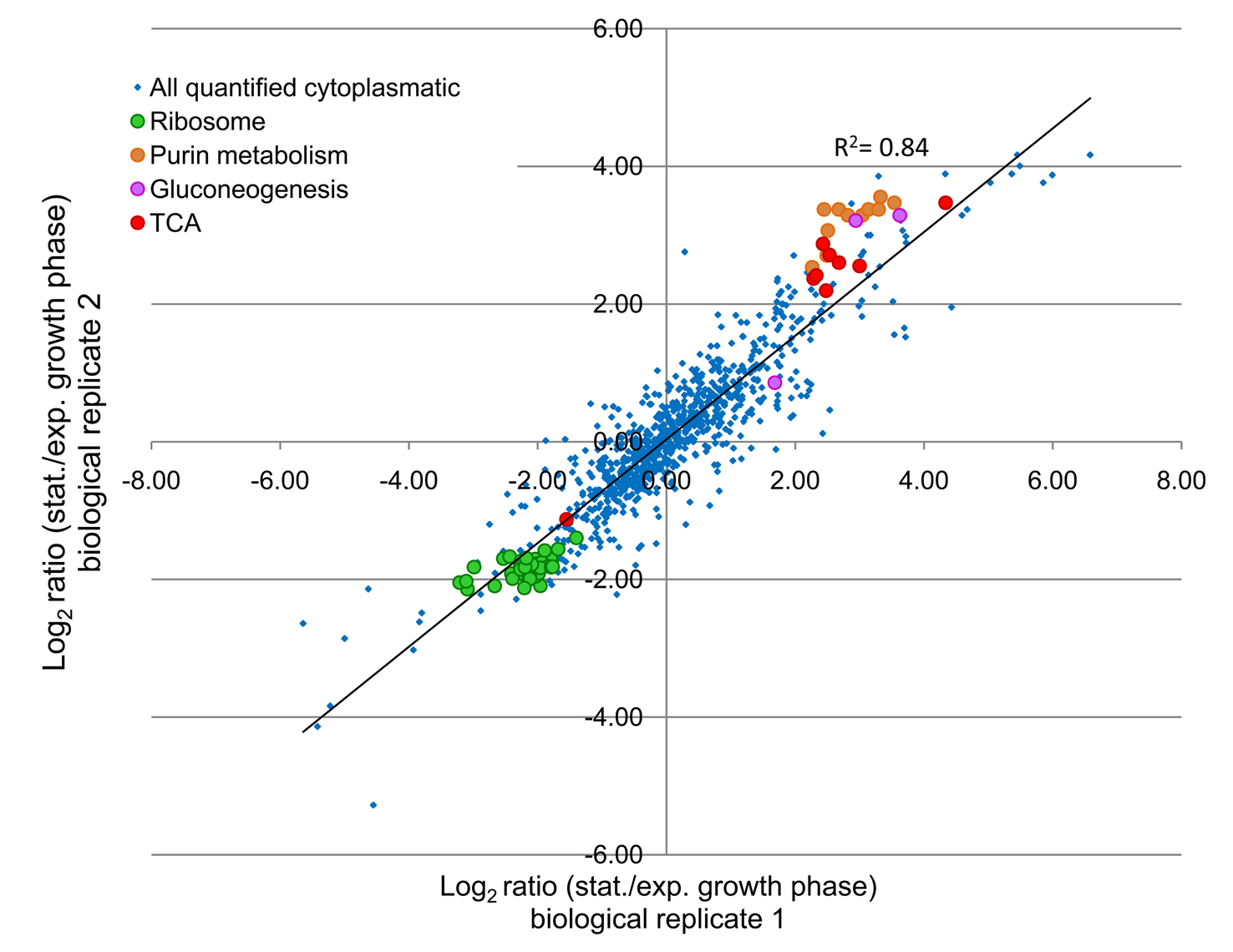

Supplement: Figure S4 — Correlation between the protein ratios of biological replicates used to obtain the quantitative data. The calculated correlation was 0.84. Selected regulated functional groups are highlighted. (0.26 MB TIF) [file pone.0008176.s005.tif]

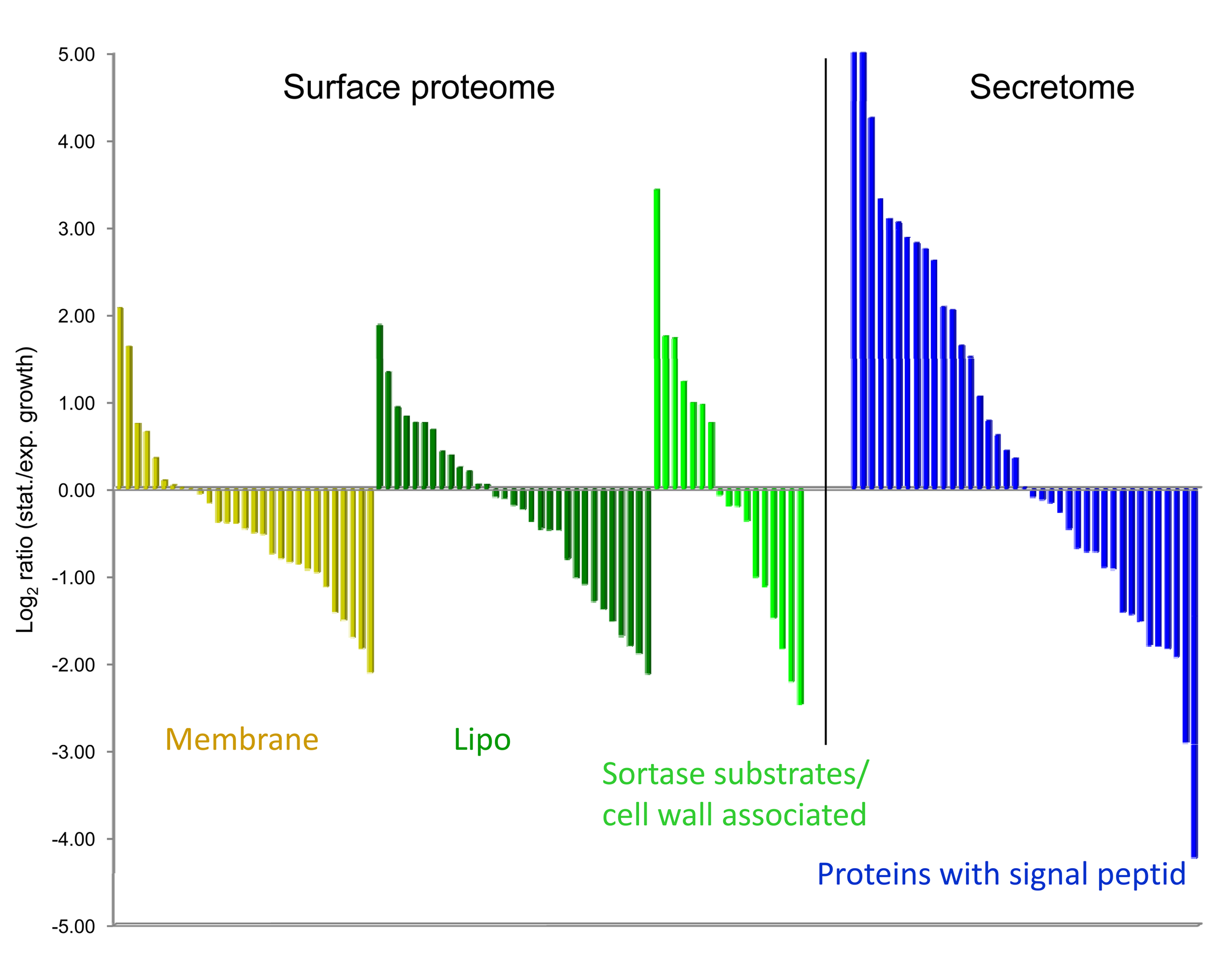

Supplement: Figure S5 — Regulation of proteins identified in the fraction of cell surface-associated and extracellular proteins. (1.46 MB TIF) [file pone.0008176.s006.tif]

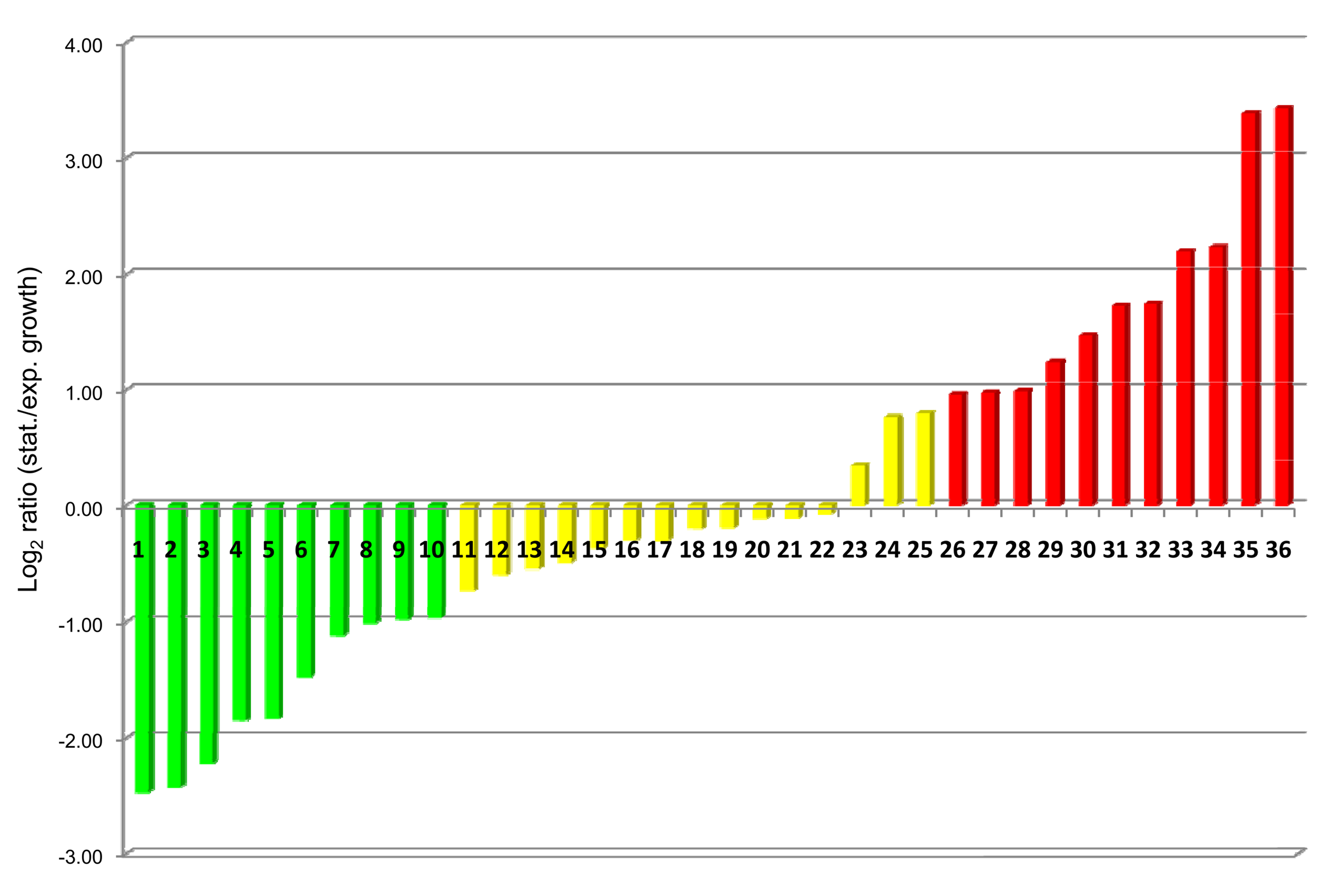

Supplement: Figure S6 — Regulated cell surface and cell wall-associated proteins. Class I proteins are colored in yellow, class II proteins in green and class III proteins in red. 1-SACOL2652 (ClfB), 2-SACOL1940, 3-SACOL2348, 4-SACOL1725 (RplT), 5-SACOL1066 (Fmt), 6-SACOL1825, 7-SACOL0938 (DltD), 8-SACOL0712, 9-SACOL2383, 10-SACOL1687, 11-SACOL0551, 12-SACOL1111, 13-SACOL1895, 14-SACOL1028 (HtrA), 15-SACOL0021 (YycH), 16-SACOL0539 (PurR), 17-SACOL0189, 18-SACOL1522 (EbpS), 19-SACOL1989, 20-SACOL0022 (YycI), 21-SACOL1140 (IsdA), 22-SACOL0610 (SdrE), 23-SACOL1788, 24-SACOL1836, 25-SACOL0968 (SpsA), 26-SACOL1514 (GpsA), 27-SACOL1168 (Efb), 28-SACOL2549, 29-SACOL0050 (Pls), 30-SACOL2019 (SdrH), 31-SACOL0856 (ClfA), 32-SACOL1062 (Atl), 33-SACOL2002 (Map), 34-SACOL0024, 35-SACOL1847, 36-SACOL2660 (IsaB). (0.65 MB TIF) [file pone.0008176.s007.tif]
