# Supplementary material for: A Proteomic View of an Important Human Pathogen – Towards the Quantification of the Entire Staphylococcus aureus Proteome
Source: PLoS One. 2009 Dec 4;4(12):e8176. doi: 10.1371/journal.pone.0008176 (PMC2781549; doi:10.1371/journal.pone.0008176)
Supplement: Table S1 — Signal intensities and calculated ratios from DNA microarray experiment. (0.32 MB PDF) [file pone.0008176.s008.pdf]

Supplementary Table 1: Signal intensities and calculated ratios from DNA microarray experiment

	SACOL[a]	signal intensity[b] exponential growth	signal intensity[b] stationary phase	signal intensity corrected[c] stationary phase	induction[d] stat/exp	induction corrected[e] stat/exp
1	**SACOL0001**	19826	18205	3641	-0,12	-2,44
2	**SACOL0002**	13466	13661	2732	0,02	-2,30
3	**SACOL0003**	38902	44576	8915	0,20	-2,13
4	**SACOL0004**	49958	41904	8381	-0,25	-2,58
5	**SACOL0005**	56220	45791	9158	-0,30	-2,62
6	**SACOL0006**	22737	12290	2458	-0,89	-3,21
7	**SACOL0007**	2025	486	97	-2,06	-4,38
8	**SACOL0008**	394	1907	381	2,27	-0,05
9	**SACOL0009**	2213	853	171	-1,38	-3,70
10	SACOL0010	8765	2876	575	-1,61	-3,93
11	SACOL0011	7396	3216	643	-1,20	-3,52
12	**SACOL0012**	12377	11997	2399	-0,04	-2,37
13	SACOL0013	30078	24947	4989	-0,27	-2,59
14	**SACOL0014**	39605	26670	5334	-0,57	-2,89
15	**SACOL0015**	30699	21702	4340	-0,50	-2,82
16	**SACOL0016**	23152	14507	2901	-0,67	-3,00
17	SACOL0017	2975	1464	293	-1,02	-3,34
18	**SACOL0018**	12676	2678	536	-2,24	-4,56
19	**SACOL0019**	27445	31430	6286	0,20	-2,13
20	**SACOL0020**	21679	18146	3629	-0,26	-2,58
21	**SACOL0021**	20636	12112	2422	-0,77	-3,09
22	**SACOL0022**	25574	13610	2722	-0,91	-3,23
23	SACOL0023	4760	3668	734	-0,38	-2,70
24	**SACOL0024**	6853	3026	605	-1,18	-3,50
25	SACOL0025	72	81	16	0,17	-2,16
26	**SACOL0026**	6418	7149	1430	0,16	-2,17
27	SACOL0027	181	237	47	0,39	-1,93
28	SACOL0028	304	731	146	1,26	-1,06
29	SACOL0029	753	2408	482	1,68	-0,65
30	SACOL0030	28	6	1	-2,11	-4,43
31	**SACOL0031**	1945	4188	838	1,11	-1,22
32	**SACOL0032**	1160	1360	272	0,23	-2,09
33	**SACOL0033**	19169	8499	1700	-1,17	-3,50
34	SACOL0034	13879	19108	3822	0,46	-1,86
35	SACOL0035	5589	9752	1950	0,80	-1,52
36	SACOL0036	209	193	39	-0,12	-2,44
37	SACOL0037	237	215	43	-0,15	-2,47
38	SACOL0038	669	383	77	-0,80	-3,12
39	SACOL0039	382	257	51	-0,57	-2,90
40	SACOL0040	503	305	61	-0,72	-3,04
41	SACOL0042	1075	1117	223	0,06	-2,27
42	SACOL0043	721	658	132	-0,13	-2,45
43	SACOL0044	569	469	94	-0,28	-2,60
44	**SACOL0045**	2318	664	133	-1,80	-4,12
45	SACOL0046	2053	2800	560	0,45	-1,87
46	SACOL0047	741	894	179	0,27	-2,05
47	SACOL0048	1561	1707	341	0,13	-2,19
48	SACOL0049	2737	3688	738	0,43	-1,89
49	**SACOL0050**	26757	37144	7429	0,47	-1,85
50	**SACOL0051**	8591	3592	718	-1,26	-3,58
51	SACOL0052	5696	1565	313	-1,86	-4,19
52	**SACOL0058**	12057	7086	1417	-0,77	-3,09
53	SACOL0061	1499	1917	383	0,36	-1,97
54	SACOL0063	621	502	100	-0,31	-2,63
55	SACOL0064	589	1382	276	1,23	-1,09
56	**SACOL0065**	644	12290	2458	4,25	1,93
57	**SACOL0067**	14449	8145	1629	-0,83	-3,15
58	SACOL0068	2980	3463	693	0,22	-2,11
59	SACOL0069	2896	3690	738	0,35	-1,97
60	SACOL0070	465	308	62	-0,60	-2,92
61	SACOL0071	251	159	32	-0,66	-2,98
62	**SACOL0072**	2751	3140	628	0,19	-2,13
63	SACOL0073	595	753	151	0,34	-1,98
64	SACOL0074	664	653	131	-0,02	-2,35
65	**SACOL0075**	3434	4215	843	0,30	-2,03
66	**SACOL0076**	11651	7144	1429	-0,71	-3,03
67	SACOL0077	3073	2123	425	-0,53	-2,86
68	**SACOL0078**	3028	647	129	-2,23	-4,55
69	SACOL0079	1696	2214	443	0,38	-1,94
70	**SACOL0080**	1871	2305	461	0,30	-2,02
71	SACOL0082	3699	3831	766	0,05	-2,27
72	SACOL0083	5077	4452	890	-0,19	-2,51

a) *S. aureus* COL Locus. Written in bold indicates that the corresponding protein was identified.
b) *LOWESS normalized background subtracted signal intensities.*
c) *Scaled stationary phase signal intensities to account for decrease in total RNA during stationary phase.*
d) *Log_2 of stationary phase versus exponential growth ratios.*
e) *Log_2 of scaled stationary phase versus exponential growth ratios.*

Supplementary Table 1: Signal intensities and calculated ratios from DNA microarray experiment

	SACOL[a]	signal intensity[b] exponential growth	signal intensity[b] stationary phase	signal intensity corrected[c] stationary phase	induction[d] stat/exp	induction corrected[e] stat/exp
73	**SACOL0084**	8325	6671	1334	-0,32	-2,64
74	**SACOL0085**	4610	14408	2882	1,64	-0,68
75	**SACOL0086**	2088	6292	1258	1,59	-0,73
76	SACOL0087	732	353	71	-1,05	-3,37
77	**SACOL0088**	8485	20233	4047	1,25	-1,07
78	SACOL0089	351	2702	540	2,95	0,62
79	SACOL0090	132	49	10	-1,42	-3,74
80	SACOL0091	984	508	102	-0,95	-3,28
81	SACOL0092	4111	19705	3941	2,26	-0,06
82	**SACOL0093**	25276	35448	7090	0,49	-1,83
83	**SACOL0095**	451	567	113	0,33	-1,99
84	**SACOL0096**	7894	4382	876	-0,85	-3,17
85	SACOL0097	3019	349	70	-3,11	-5,43
86	SACOL0098	2569	345	69	-2,89	-5,22
87	**SACOL0099**	1402	235	47	-2,58	-4,90
88	SACOL0100	281	99	20	-1,51	-3,83
89	SACOL0101	319	212	42	-0,59	-2,92
90	**SACOL0102**	446	316	63	-0,50	-2,82
91	SACOL0103	433	331	66	-0,39	-2,71
92	**SACOL0104**	714	594	119	-0,27	-2,59
93	SACOL0105	828	721	144	-0,20	-2,52
94	SACOL0106	1400	1409	282	0,01	-2,31
95	SACOL0107	1246	1242	248	0,00	-2,33
96	SACOL0108	536	824	165	0,62	-1,70
97	SACOL0109	3494	3209	642	-0,12	-2,44
98	**SACOL0110**	4566	4896	979	0,10	-2,22
99	**SACOL0111**	9781	29429	5886	1,59	-0,73
100	SACOL0113	1294	414	83	-1,64	-3,97
101	SACOL0114	889	264	53	-1,75	-4,07
102	SACOL0115	659	290	58	-1,19	-3,51
103	SACOL0116	904	152	30	-2,58	-4,90
104	SACOL0117	1480	1860	372	0,33	-1,99
105	**SACOL0118**	6125	4264	853	-0,52	-2,84
106	SACOL0119	1750	700	140	-1,32	-3,64
107	**SACOL0120**	3993	5708	1142	0,52	-1,81
108	SACOL0121	498	1413	283	1,50	-0,82
109	SACOL0122	4648	6746	1349	0,54	-1,78
110	**SACOL0124**	30071	33542	6708	0,16	-2,16
111	SACOL0125	349	421	84	0,27	-2,05
112	SACOL0126	549	587	117	0,10	-2,23
113	SACOL0127	518	503	101	-0,04	-2,36
114	**SACOL0128**	705	772	154	0,13	-2,19
115	**SACOL0129**	332	495	99	0,58	-1,74
116	SACOL0130	1914	2087	417	0,12	-2,20
117	**SACOL0135**	136	290	58	1,09	-1,23
118	**SACOL0136**	4027	155455	31091	5,27	2,95
119	**SACOL0137**	7026	263718	52744	5,23	2,91
120	**SACOL0138**	4542	107154	21431	4,56	2,24
121	**SACOL0139**	6584	127140	25428	4,27	1,95
122	**SACOL0140**	6626	70455	14091	3,41	1,09
123	**SACOL0141**	8726	108765	21753	3,64	1,32
124	**SACOL0142**	7772	85387	17077	3,46	1,14
125	**SACOL0143**	7206	61292	12258	3,09	0,77
126	**SACOL0144**	5440	45698	9140	3,07	0,75
127	**SACOL0145**	7526	40322	8064	2,42	0,10
128	SACOL0146	8266	43727	8745	2,40	0,08
129	**SACOL0147**	11806	49877	9975	2,08	-0,24
130	**SACOL0148**	13324	56049	11210	2,07	-0,25
131	**SACOL0149**	10743	53589	10718	2,32	0,00
132	**SACOL0150**	10281	49180	9836	2,26	-0,06
133	**SACOL0151**	7882	33544	6709	2,09	-0,23
134	**SACOL0152**	403	476	95	0,24	-2,08
135	SACOL0153	428	450	90	0,07	-2,25
136	**SACOL0154**	4909	19099	3820	1,96	-0,36
137	**SACOL0155**	839	6157	1231	2,88	0,55
138	SACOL0159	1076	293	59	-1,88	-4,20
139	SACOL0160	711	724	145	0,03	-2,30
140	**SACOL0161**	16004	14493	2899	-0,14	-2,47
141	**SACOL0162**	3444	2463	493	-0,48	-2,81
142	SACOL0163	3939	4667	933	0,24	-2,08
143	**SACOL0164**	4513	3086	617	-0,55	-2,87
144	SACOL0165	6540	3219	644	-1,02	-3,34

a) *S. aureus* COL Locus. Written in bold indicates that the corresponding protein was identified.
b) *LOWESS normalized background subtracted signal intensities.*
c) *Scaled stationary phase signal intensities to account for decrease in total RNA during stationary phase.*
d) *Log_2 of stationary phase versus exponential growth ratios.*
e) *Log_2 of scaled stationary phase versus exponential growth ratios.*

Supplementary Table 1: Signal intensities and calculated ratios from DNA microarray experiment

	SACOL[a]	signal intensity[b] exponential growth	signal intensity[b] stationary phase	signal intensity corrected[c] stationary phase	induction[d] stat/exp	induction corrected[e] stat/exp
145	**SACOL0166**	2052	1246	249	-0,72	-3,04
146	SACOL0167	1260	623	125	-1,02	-3,34
147	SACOL0168	1170	525	105	-1,16	-3,48
148	SACOL0169	939	182	36	-2,37	-4,69
149	SACOL0170	1293	404	81	-1,68	-4,00
150	**SACOL0171**	7262	1579	316	-2,20	-4,52
151	SACOL0172	11083	4017	803	-1,46	-3,79
152	**SACOL0173**	22742	6128	1226	-1,89	-4,21
153	SACOL0174	27	5	1	-2,58	-4,90
154	**SACOL0175**	45806	5633	1127	-3,02	-5,35
155	**SACOL0176**	1369	5869	1174	2,10	-0,22
156	SACOL0177	989	4217	843	2,09	-0,23
157	**SACOL0178**	2578	9754	1951	1,92	-0,40
158	SACOL0179	1032	3475	695	1,75	-0,57
159	**SACOL0180**	5071	3164	633	-0,68	-3,00
160	**SACOL0181**	7534	7257	1451	-0,05	-2,38
161	**SACOL0182**	9805	10507	2101	0,10	-2,22
162	SACOL0183	10562	12101	2420	0,20	-2,13
163	**SACOL0184**	2354	1209	242	-0,96	-3,28
164	SACOL0185	2933	155	31	-4,24	-6,56
165	SACOL0186	6235	340	68	-4,20	-6,52
166	**SACOL0187**	11089	541	108	-4,36	-6,68
167	SACOL0188	3546	321	64	-3,46	-5,79
168	**SACOL0189**	3345	3787	757	0,18	-2,14
169	**SACOL0190**	1160	13378	2676	3,53	1,21
170	**SACOL0191**	5932	3618	724	-0,71	-3,04
171	**SACOL0192**	1017	316	63	-1,69	-4,01
172	**SACOL0193**	1233	406	81	-1,60	-3,92
173	**SACOL0194**	2811	2011	402	-0,48	-2,81
174	**SACOL0195**	1215	1154	231	-0,07	-2,40
175	**SACOL0196**	1813	2294	459	0,34	-1,98
176	**SACOL0197**	1288	1780	356	0,47	-1,86
177	**SACOL0198**	745	1136	227	0,61	-1,71
178	**SACOL0199**	1268	145	29	-3,13	-5,45
179	**SACOL0200**	1847	822	164	-1,17	-3,49
180	**SACOL0201**	3801	1057	211	-1,85	-4,17
181	**SACOL0202**	3425	1070	214	-1,68	-4,00
182	**SACOL0203**	2747	1326	265	-1,05	-3,37
183	**SACOL0204**	912	1834	367	1,01	-1,31
184	SACOL0205	1047	2698	540	1,36	-0,96
185	SACOL0206	313	289	58	-0,12	-2,44
186	SACOL0207	554	596	119	0,11	-2,22
187	SACOL0208	326	278	56	-0,23	-2,55
188	**SACOL0209**	820	622	124	-0,40	-2,72
189	SACOL0210	83	229	46	1,46	-0,87
190	**SACOL0211**	331	7129	1426	4,43	2,11
191	**SACOL0212**	150	8290	1658	5,79	3,47
192	**SACOL0213**	103	14114	2823	7,10	4,78
193	**SACOL0214**	794	13381	2676	4,08	1,75
194	SACOL0215	463	22459	4492	5,60	3,28
195	**SACOL0216**	3766	865	173	-2,12	-4,45
196	**SACOL0217**	1223	468	94	-1,38	-3,71
197	SACOL0218	973	884	177	-0,14	-2,46
198	SACOL0219	717	817	163	0,19	-2,13
199	**SACOL0220**	1429	1437	287	0,01	-2,31
200	**SACOL0222**	15942	591	118	-4,75	-7,08
201	SACOL0223	195	315	63	0,69	-1,63
202	**SACOL0224**	378	1694	339	2,16	-0,16
203	SACOL0225	996	846	169	-0,24	-2,56
204	SACOL0228	444	444	89	0,00	-2,32
205	SACOL0229	520	529	106	0,02	-2,30
206	SACOL0230	437	534	107	0,29	-2,03
207	SACOL0231	48	69	14	0,52	-1,81
208	SACOL0232	1552	2361	472	0,61	-1,72
209	SACOL0233	1188	1834	367	0,63	-1,70
210	SACOL0234	1020	1428	286	0,49	-1,84
211	SACOL0235	926	1038	208	0,16	-2,16
212	**SACOL0236**	32757	28720	5744	-0,19	-2,51
213	**SACOL0237**	16371	11200	2240	-0,55	-2,87
214	**SACOL0238**	16748	8525	1705	-0,97	-3,30
215	**SACOL0239**	10898	4538	908	-1,26	-3,59
216	**SACOL0240**	17915	14849	2970	-0,27	-2,59

a) *S. aureus* COL Locus. Written in bold indicates that the corresponding protein was identified.
b) *LOWESS normalized background subtracted signal intensities.*
c) *Scaled stationary phase signal intensities to account for decrease in total RNA during stationary phase.*
d) *Log_2 of stationary phase versus exponential growth ratios.*
e) *Log_2 of scaled stationary phase versus exponential growth ratios.*

Supplementary Table 1: Signal intensities and calculated ratios from DNA microarray experiment

	SACOL[a]	signal intensity[b] exponential growth	signal intensity[b] stationary phase	signal intensity corrected[c] stationary phase	induction[d] stat/exp	induction corrected[e] stat/exp
217	**SACOL0241**	38628	45729	9146	0,24	-2,08
218	**SACOL0242**	17351	16095	3219	-0,11	-2,43
219	**SACOL0243**	49164	35670	7134	-0,46	-2,78
220	SACOL0244	6685	5051	1010	-0,40	-2,73
221	**SACOL0245**	4480	5161	1032	0,20	-2,12
222	SACOL0246	3841	3225	645	-0,25	-2,57
223	SACOL0247	871	4546	909	2,38	0,06
224	SACOL0248	655	4383	877	2,74	0,42
225	**SACOL0249**	1931	3856	771	1,00	-1,32
226	SACOL0250	496	2225	445	2,16	-0,16
227	SACOL0251	731	2518	504	1,78	-0,54
228	SACOL0252	779	236	47	-1,73	-4,05
229	**SACOL0253**	11495	4753	951	-1,27	-3,60
230	**SACOL0254**	15496	4850	970	-1,68	-4,00
231	**SACOL0255**	14913	3471	694	-2,10	-4,42
232	**SACOL0257**	2537	6759	1352	1,41	-0,91
233	SACOL0260	439	134	27	-1,71	-4,03
234	SACOL0261	1620	209	42	-2,95	-5,27
235	SACOL0262	1996	2486	497	0,32	-2,01
236	**SACOL0263**	8010	6421	1284	-0,32	-2,64
237	SACOL0264	354	392	78	0,15	-2,18
238	SACOL0265	507	405	81	-0,33	-2,65
239	SACOL0266	346	262	52	-0,40	-2,72
240	SACOL0267	300	555	111	0,89	-1,43
241	SACOL0268	767	1872	374	1,29	-1,03
242	SACOL0269	175	443	89	1,34	-0,98
243	**SACOL0270**	2597	2498	500	-0,06	-2,38
244	**SACOL0271**	54576	26211	5242	-1,06	-3,38
245	**SACOL0272**	8789	1804	361	-2,28	-4,61
246	**SACOL0273**	731	191	38	-1,94	-4,26
247	SACOL0274	5644	926	185	-2,61	-4,93
248	**SACOL0275**	5805	958	192	-2,60	-4,92
249	**SACOL0276**	6941	1863	373	-1,90	-4,22
250	**SACOL0277**	14692	3025	605	-2,28	-4,60
251	SACOL0278	13031	2634	527	-2,31	-4,63
252	**SACOL0279**	15306	3285	657	-2,22	-4,54
253	SACOL0280	14438	3773	755	-1,94	-4,26
254	**SACOL0281**	15686	19936	3987	0,35	-1,98
255	**SACOL0284**	2754	1078	216	-1,35	-3,67
256	SACOL0286	3045	2725	545	-0,16	-2,48
257	SACOL0287	9234	7930	1586	-0,22	-2,54
258	SACOL0289	4293	4372	874	0,03	-2,30
259	**SACOL0297**	4461	4515	903	0,02	-2,30
260	**SACOL0299**	4463	2015	403	-1,15	-3,47
261	SACOL0300	4828	2562	512	-0,91	-3,24
262	SACOL0301	723	198	40	-1,87	-4,19
263	**SACOL0302**	25219	4315	863	-2,55	-4,87
264	**SACOL0303**	1325	465	93	-1,51	-3,83
265	**SACOL0305**	4521	35804	7161	2,99	0,66
266	**SACOL0306**	2308	20864	4173	3,18	0,85
267	**SACOL0307**	4213	11370	2274	1,43	-0,89
268	SACOL0308	419	848	170	1,02	-1,31
269	SACOL0309	320	654	131	1,03	-1,29
270	SACOL0310	365	917	183	1,33	-0,99
271	**SACOL0311**	110	307	61	1,48	-0,85
272	**SACOL0312**	1773	2685	537	0,60	-1,72
273	**SACOL0313**	596	4950	990	3,06	0,73
274	**SACOL0314**	6608	4242	848	-0,64	-2,96
275	**SACOL0315**	887	1988	398	1,16	-1,16
276	**SACOL0316**	598	4890	978	3,03	0,71
277	**SACOL0317**	10279	11800	2360	0,20	-2,12
278	SACOL0318	2604	2518	504	-0,05	-2,37
279	**SACOL0319**	11787	15081	3016	0,36	-1,97
280	SACOL0320	10014	14756	2951	0,56	-1,76
281	**SACOL0321**	7501	11914	2383	0,67	-1,65
282	SACOL0323	2242	1386	277	-0,69	-3,02
283	SACOL0324	1417	566	113	-1,32	-3,65
284	SACOL0325	10544	7507	1501	-0,49	-2,81
285	SACOL0326	4668	3980	796	-0,23	-2,55
286	SACOL0327	3980	3361	672	-0,24	-2,57
287	**SACOL0328**	3309	1308	262	-1,34	-3,66
288	SACOL0329	2155	2078	416	-0,05	-2,37

a) *S. aureus* COL Locus. Written in bold indicates that the corresponding protein was identified.
b) *LOWESS normalized background subtracted signal intensities.*
c) *Scaled stationary phase signal intensities to account for decrease in total RNA during stationary phase.*
d) *Log_2 of stationary phase versus exponential growth ratios.*
e) *Log_2 of scaled stationary phase versus exponential growth ratios.*

Supplementary Table 1: Signal intensities and calculated ratios from DNA microarray experiment

	SACOL[a]	signal intensity[b] exponential growth	signal intensity[b] stationary phase	signal intensity corrected[c] stationary phase	induction[d] stat/exp	induction corrected[e] stat/exp
289	**SACOL0330**	7323	3442	688	-1,09	-3,41
290	SACOL0331	2056	1951	390	-0,08	-2,40
291	SACOL0332	6313	3858	772	-0,71	-3,03
292	SACOL0333	4804	3512	702	-0,45	-2,77
293	SACOL0334	1982	1338	268	-0,57	-2,89
294	SACOL0335	2357	2151	430	-0,13	-2,45
295	SACOL0336	2723	3152	630	0,21	-2,11
296	SACOL0337	2142	2798	560	0,39	-1,94
297	SACOL0338	3735	4473	895	0,26	-2,06
298	**SACOL0339**	2040	3343	669	0,71	-1,61
299	SACOL0340	1860	2836	567	0,61	-1,71
300	**SACOL0341**	2367	3593	719	0,60	-1,72
301	SACOL0342	3509	4645	929	0,40	-1,92
302	SACOL0343	3622	4243	849	0,23	-2,09
303	SACOL0344	1885	2449	490	0,38	-1,94
304	SACOL0345	2151	2775	555	0,37	-1,95
305	SACOL0346	2450	2719	544	0,15	-2,17
306	SACOL0347	3019	3069	614	0,02	-2,30
307	SACOL0348	2439	2610	522	0,10	-2,22
308	SACOL0350	2947	3259	652	0,15	-2,18
309	SACOL0351	3801	4080	816	0,10	-2,22
310	SACOL0352	2684	3001	600	0,16	-2,16
311	SACOL0353	3344	3510	702	0,07	-2,25
312	SACOL0354	5344	5749	1150	0,11	-2,22
313	SACOL0355	4881	5582	1116	0,19	-2,13
314	SACOL0356	4796	5713	1143	0,25	-2,07
315	**SACOL0357**	5586	7231	1446	0,37	-1,95
316	SACOL0358	5211	7310	1462	0,49	-1,83
317	SACOL0359	4573	6194	1239	0,44	-1,88
318	SACOL0361	5812	8263	1653	0,51	-1,81
319	SACOL0362	2114	3304	661	0,64	-1,68
320	SACOL0363	2896	5009	1002	0,79	-1,53
321	SACOL0364	3923	5946	1189	0,60	-1,72
322	SACOL0365	1662	1502	300	-0,15	-2,47
323	SACOL0366	790	759	152	-0,06	-2,38
324	SACOL0367	3275	2697	539	-0,28	-2,60
325	**SACOL0368**	3572	3235	647	-0,14	-2,46
326	SACOL0369	5785	4606	921	-0,33	-2,65
327	**SACOL0370**	6605	5561	1112	-0,25	-2,57
328	SACOL0371	4915	3678	736	-0,42	-2,74
329	SACOL0372	5049	3340	668	-0,60	-2,92
330	SACOL0373	4399	3305	661	-0,41	-2,73
331	SACOL0374	4011	3164	633	-0,34	-2,66
332	**SACOL0375**	7640	8016	1603	0,07	-2,25
333	**SACOL0376**	6394	7157	1431	0,16	-2,16
334	SACOL0378	4222	3884	777	-0,12	-2,44
335	SACOL0379	4024	3128	626	-0,36	-2,69
336	SACOL0380	2187	2498	500	0,19	-2,13
337	SACOL0381	3836	4318	864	0,17	-2,15
338	SACOL0382	2109	2101	420	-0,01	-2,33
339	**SACOL0383**	2820	2766	553	-0,03	-2,35
340	**SACOL0384**	3298	2476	495	-0,41	-2,74
341	**SACOL0385**	2601	2319	464	-0,17	-2,49
342	SACOL0387	2069	1500	300	-0,46	-2,79
343	SACOL0388	2321	1115	223	-1,06	-3,38
344	SACOL0389	766	497	99	-0,63	-2,95
345	SACOL0390	477	417	83	-0,19	-2,52
346	**SACOL0391**	3494	4009	802	0,20	-2,12
347	**SACOL0392**	2705	3658	732	0,44	-1,89
348	SACOL0394	6674	5489	1098	-0,28	-2,60
349	SACOL0395	9044	6537	1307	-0,47	-2,79
350	SACOL0396	6551	4685	937	-0,48	-2,81
351	SACOL0397	5433	2769	554	-0,97	-3,29
352	**SACOL0398**	4851	2200	440	-1,14	-3,46
353	**SACOL0399**	3908	22027	4405	2,49	0,17
354	**SACOL0400**	338	980	196	1,53	-0,79
355	SACOL0401	577	1484	297	1,36	-0,96
356	SACOL0402	541	1143	229	1,08	-1,24
357	SACOL0403	336	785	157	1,22	-1,10
358	SACOL0404	3521	629	126	-2,49	-4,81
359	SACOL0405	2146	175	35	-3,61	-5,93
360	SACOL0406	1382	522	104	-1,40	-3,73

a) *S. aureus* COL Locus. Written in bold indicates that the corresponding protein was identified.
b) *LOWESS normalized background subtracted signal intensities.*
c) *Scaled stationary phase signal intensities to account for decrease in total RNA during stationary phase.*
d) *Log_2 of stationary phase versus exponential growth ratios.*
e) *Log_2 of scaled stationary phase versus exponential growth ratios.*

Supplementary Table 1: Signal intensities and calculated ratios from DNA microarray experiment

	SACOL[a]	signal intensity[b] exponential growth	signal intensity[b] stationary phase	signal intensity corrected[c] stationary phase	induction[d] stat/exp	induction corrected[e] stat/exp
361	**SACOL0407**	331	373	75	0,17	-2,15
362	SACOL0408	3254	43791	8758	3,75	1,43
363	**SACOL0409**	2506	43739	8748	4,13	1,80
364	**SACOL0410**	656	6055	1211	3,21	0,89
365	**SACOL0411**	834	1039	208	0,32	-2,01
366	SACOL0412	860	2370	474	1,46	-0,86
367	**SACOL0413**	1328	12888	2578	3,28	0,96
368	**SACOL0414**	741	5845	1169	2,98	0,66
369	SACOL0415	1021	4882	976	2,26	-0,06
370	SACOL0416	516	1723	345	1,74	-0,58
371	SACOL0417	1525	234	47	-2,70	-5,02
372	SACOL0418	12606	1508	302	-3,06	-5,38
373	**SACOL0419**	5945	660	132	-3,17	-5,49
374	SACOL0420	4469	5505	1101	0,30	-2,02
375	**SACOL0421**	5378	6381	1276	0,25	-2,08
376	**SACOL0422**	4451	6081	1216	0,45	-1,87
377	**SACOL0424**	4126	4851	970	0,23	-2,09
378	SACOL0425	1140	293	59	-1,96	-4,28
379	**SACOL0426**	11160	9952	1990	-0,17	-2,49
380	**SACOL0427**	21978	2196	439	-3,32	-5,64
381	**SACOL0428**	34580	2116	423	-4,03	-6,35
382	**SACOL0429**	15293	720	144	-4,41	-6,73
383	**SACOL0430**	11630	761	152	-3,93	-6,26
384	**SACOL0431**	17206	1589	318	-3,44	-5,76
385	SACOL0432	2199	4487	897	1,03	-1,29
386	**SACOL0433**	14448	7432	1486	-0,96	-3,28
387	**SACOL0435**	18269	10627	2125	-0,78	-3,10
388	SACOL0436	5129	2646	529	-0,95	-3,28
389	**SACOL0437**	105501	7445	1489	-3,82	-6,15
390	**SACOL0438**	119876	9159	1832	-3,71	-6,03
391	**SACOL0439**	7165	567	113	-3,66	-5,98
392	SACOL0440	211	492	98	1,22	-1,10
393	**SACOL0442**	260	550	110	1,08	-1,24
394	SACOL0443	622	1112	222	0,84	-1,48
395	**SACOL0444**	1873	19325	3865	3,37	1,04
396	**SACOL0445**	553	1267	253	1,20	-1,13
397	**SACOL0446**	7475	53230	10646	2,83	0,51
398	**SACOL0447**	4179	2199	440	-0,93	-3,25
399	SACOL0448	6320	3624	725	-0,80	-3,12
400	**SACOL0449**	7162	7055	1411	-0,02	-2,34
401	SACOL0450	4890	3042	608	-0,68	-3,01
402	**SACOL0451**	12127	57086	11417	2,23	-0,09
403	**SACOL0452**	20592	134338	26868	2,71	0,38
404	**SACOL0453**	7572	16064	3213	1,09	-1,24
405	**SACOL0454**	1781	430	86	-2,05	-4,37
406	**SACOL0455**	4733	9859	1972	1,06	-1,26
407	**SACOL0456**	14568	65616	13123	2,17	-0,15
408	**SACOL0457**	4768	34226	6845	2,84	0,52
409	**SACOL0458**	4632	3410	682	-0,44	-2,76
410	**SACOL0459**	9365	6199	1240	-0,60	-2,92
411	**SACOL0460**	16300	17392	3478	0,09	-2,23
412	**SACOL0461**	10953	9616	1923	-0,19	-2,51
413	**SACOL0462**	1326	4293	859	1,70	-0,63
414	SACOL0463	379	239	48	-0,67	-2,99
415	**SACOL0464**	1206	1382	276	0,20	-2,13
416	SACOL0465	2516	3585	717	0,51	-1,81
417	**SACOL0466**	2585	5923	1185	1,20	-1,13
418	**SACOL0467**	3883	10689	2138	1,46	-0,86
419	**SACOL0468**	1407	7949	1590	2,50	0,18
420	SACOL0469	147	210	42	0,52	-1,80
421	SACOL0470	154	181	36	0,23	-2,09
422	SACOL0472	32	39	8	0,31	-2,02
423	SACOL0473	229	245	49	0,10	-2,22
424	SACOL0474	1545	1536	307	-0,01	-2,33
425	SACOL0475	673	871	174	0,37	-1,95
426	**SACOL0477**	9733	2323	465	-2,07	-4,39
427	**SACOL0478**	757	611	122	-0,31	-2,63
428	**SACOL0479**	548	1217	243	1,15	-1,17
429	**SACOL0480**	942	23334	4667	4,63	2,31
430	SACOL0481	2284	4050	810	0,83	-1,50
431	**SACOL0482**	2908	3780	756	0,38	-1,94
432	SACOL0483	4365	4483	897	0,04	-2,28

a) *S. aureus* COL Locus. Written in bold indicates that the corresponding protein was identified.
b) *LOWESS normalized background subtracted signal intensities.*
c) *Scaled stationary phase signal intensities to account for decrease in total RNA during stationary phase.*
d) *Log_2 of stationary phase versus exponential growth ratios.*
e) *Log_2 of scaled stationary phase versus exponential growth ratios.*

Supplementary Table 1: Signal intensities and calculated ratios from DNA microarray experiment

	SACOL[a]	signal intensity[b] exponential growth	signal intensity[b] stationary phase	signal intensity corrected[c] stationary phase	induction[d] stat/exp	induction corrected[e] stat/exp
433	**SACOL0484**	5658	5843	1169	0,05	-2,28
434	**SACOL0485**	7523	8645	1729	0,20	-2,12
435	**SACOL0486**	6177	21651	4330	1,81	-0,51
436	**SACOL0487**	11647	33638	6728	1,53	-0,79
437	**SACOL0488**	17719	37100	7420	1,07	-1,26
438	**SACOL0489**	16805	34751	6950	1,05	-1,27
439	SACOL0490	2219	6395	1279	1,53	-0,79
440	SACOL0491	2588	1081	216	-1,26	-3,58
441	**SACOL0494**	12710	18112	3622	0,51	-1,81
442	**SACOL0495**	13616	22148	4430	0,70	-1,62
443	**SACOL0496**	10665	9443	1889	-0,18	-2,50
444	SACOL0497	3559	3370	674	-0,08	-2,40
445	**SACOL0498**	16852	6809	1362	-1,31	-3,63
446	**SACOL0499**	10090	6194	1239	-0,70	-3,03
447	SACOL0500	3028	511	102	-2,57	-4,89
448	SACOL0501	1179	148	30	-2,99	-5,32
449	SACOL0502	5175	453	91	-3,51	-5,84
450	**SACOL0503**	3397	435	87	-2,96	-5,29
451	**SACOL0504**	9257	566	113	-4,03	-6,35
452	**SACOL0505**	22117	1918	384	-3,53	-5,85
453	**SACOL0506**	14834	925	185	-4,00	-6,32
454	**SACOL0507**	67305	34185	6837	-0,98	-3,30
455	**SACOL0508**	4812	1925	385	-1,32	-3,64
456	**SACOL0509**	8192	5585	1117	-0,55	-2,87
457	**SACOL0510**	3187	3273	655	0,04	-2,28
458	SACOL0511	7564	2089	418	-1,86	-4,18
459	**SACOL0512**	4415	1158	232	-1,93	-4,25
460	SACOL0513	3481	2553	511	-0,45	-2,77
461	**SACOL0514**	22127	12611	2522	-0,81	-3,13
462	**SACOL0515**	25161	11012	2202	-1,19	-3,51
463	**SACOL0516**	3677	4360	872	0,25	-2,08
464	SACOL0517	2201	2307	461	0,07	-2,25
465	SACOL0518	1443	1093	219	-0,40	-2,72
466	**SACOL0519**	12860	4653	931	-1,47	-3,79
467	**SACOL0520**	36486	18277	3655	-1,00	-3,32
468	**SACOL0521**	23076	29186	5837	0,34	-1,98
469	**SACOL0522**	7885	7552	1510	-0,06	-2,38
470	**SACOL0523**	1772	1889	378	0,09	-2,23
471	**SACOL0524**	2573	4062	812	0,66	-1,66
472	**SACOL0526**	19367	15645	3129	-0,31	-2,63
473	SACOL0527	20320	14336	2867	-0,50	-2,83
474	**SACOL0528**	14328	6253	1251	-1,20	-3,52
475	**SACOL0529**	12704	3281	656	-1,95	-4,28
476	SACOL0530	28192	6258	1252	-2,17	-4,49
477	SACOL0531	9426	2418	484	-1,96	-4,28
478	**SACOL0533**	25646	6920	1384	-1,89	-4,21
479	**SACOL0534**	19991	7597	1519	-1,40	-3,72
480	SACOL0535	17260	5462	1092	-1,66	-3,98
481	**SACOL0536**	8734	2774	555	-1,65	-3,98
482	SACOL0537	27145	3346	669	-3,02	-5,34
483	**SACOL0538**	81222	6225	1245	-3,71	-6,03
484	**SACOL0539**	34502	244787	48957	2,83	0,50
485	**SACOL0540**	48687	213850	42770	2,14	-0,19
486	**SACOL0541**	17985	47335	9467	1,40	-0,93
487	**SACOL0543**	38202	24924	4985	-0,62	-2,94
488	**SACOL0544**	48205	26682	5336	-0,85	-3,18
489	**SACOL0545**	22799	4976	995	-2,20	-4,52
490	**SACOL0546**	10671	10870	2174	0,03	-2,30
491	**SACOL0547**	10826	5887	1177	-0,88	-3,20
492	**SACOL0548**	24345	9933	1987	-1,29	-3,62
493	**SACOL0549**	37436	20593	4119	-0,86	-3,18
494	**SACOL0550**	16351	10255	2051	-0,67	-2,99
495	**SACOL0551**	12872	7718	1544	-0,74	-3,06
496	**SACOL0552**	9748	4201	840	-1,21	-3,54
497	**SACOL0553**	25240	6607	1321	-1,93	-4,26
498	**SACOL0554**	30743	11551	2310	-1,41	-3,73
499	**SACOL0555**	35708	14728	2946	-1,28	-3,60
500	**SACOL0556**	28124	6971	1394	-2,01	-4,33
501	**SACOL0557**	6583	8948	1790	0,44	-1,88
502	**SACOL0558**	6091	1649	330	-1,89	-4,21
503	**SACOL0559**	9767	3801	760	-1,36	-3,68
504	**SACOL0560**	6877	2783	557	-1,31	-3,63

a) *S. aureus* COL Locus. Written in bold indicates that the corresponding protein was identified.
b) *LOWESS normalized background subtracted signal intensities.*
c) *Scaled stationary phase signal intensities to account for decrease in total RNA during stationary phase.*
d) *Log_2 of stationary phase versus exponential growth ratios.*
e) *Log_2 of scaled stationary phase versus exponential growth ratios.*

Supplementary Table 1: Signal intensities and calculated ratios from DNA microarray experiment

	SACOL[a]	signal intensity[b] exponential growth	signal intensity[b] stationary phase	signal intensity corrected[c] stationary phase	induction[d] stat/exp	induction corrected[e] stat/exp
505	**SACOL0562**	22702	7054	1411	-1,69	-4,01
506	**SACOL0563**	444	1048	210	1,24	-1,08
507	**SACOL0564**	33557	22281	4456	-0,59	-2,91
508	**SACOL0565**	22390	15894	3179	-0,49	-2,82
509	**SACOL0566**	13389	4703	941	-1,51	-3,83
510	**SACOL0567**	18056	29799	5960	0,72	-1,60
511	**SACOL0568**	25012	40868	8174	0,71	-1,61
512	**SACOL0569**	21866	29597	5919	0,44	-1,89
513	**SACOL0572**	20135	13534	2707	-0,57	-2,90
514	**SACOL0573**	18939	10916	2183	-0,79	-3,12
515	**SACOL0574**	42645	39729	7946	-0,10	-2,42
516	**SACOL0575**	21151	67071	13414	1,66	-0,66
517	**SACOL0576**	16585	40275	8055	1,28	-1,04
518	**SACOL0577**	20159	46909	9382	1,22	-1,10
519	**SACOL0578**	22201	38453	7691	0,79	-1,53
520	**SACOL0579**	22194	38060	7612	0,78	-1,54
521	SACOL0580	22274	28403	5681	0,35	-1,97
522	**SACOL0581**	39475	32325	6465	-0,29	-2,61
523	**SACOL0582**	39249	34749	6950	-0,18	-2,50
524	**SACOL0583**	81876	20636	4127	-1,99	-4,31
525	**SACOL0584**	21673	4662	932	-2,22	-4,54
526	**SACOL0585**	51853	3811	762	-3,77	-6,09
527	**SACOL0587**	21030	3970	794	-2,41	-4,73
528	**SACOL0588**	68422	34497	6899	-0,99	-3,31
529	**SACOL0589**	104115	44660	8932	-1,22	-3,54
530	SACOL0590	175045	36788	7358	-2,25	-4,57
531	**SACOL0591**	109668	33177	6635	-1,72	-4,05
532	**SACOL0592**	132763	36364	7273	-1,87	-4,19
533	**SACOL0593**	128781	28923	5785	-2,15	-4,48
534	**SACOL0594**	102572	29904	5981	-1,78	-4,10
535	**SACOL0595**	752	1429	286	0,93	-1,40
536	**SACOL0596**	12053	30925	6185	1,36	-0,96
537	**SACOL0597**	43849	13595	2719	-1,69	-4,01
538	**SACOL0598**	999	2911	582	1,54	-0,78
539	**SACOL0599**	24843	6949	1390	-1,84	-4,16
540	**SACOL0600**	23598	42677	8535	0,85	-1,47
541	**SACOL0602**	9034	23478	4696	1,38	-0,94
542	**SACOL0603**	10059	2623	525	-1,94	-4,26
543	**SACOL0604**	6432	1345	269	-2,26	-4,58
544	**SACOL0606**	12945	14565	2913	0,17	-2,15
545	**SACOL0607**	1669	4194	839	1,33	-0,99
546	**SACOL0608**	2902	2695	539	-0,11	-2,43
547	**SACOL0609**	940	1599	320	0,77	-1,56
548	**SACOL0610**	6346	2592	518	-1,29	-3,61
549	**SACOL0611**	1905	4357	871	1,19	-1,13
550	**SACOL0612**	18356	22264	4453	0,28	-2,04
551	**SACOL0613**	14976	6981	1396	-1,10	-3,42
552	**SACOL0614**	25401	13476	2695	-0,91	-3,24
553	**SACOL0615**	37203	20904	4181	-0,83	-3,15
554	**SACOL0616**	8746	14153	2831	0,69	-1,63
555	**SACOL0617**	8244	47869	9574	2,54	0,22
556	**SACOL0618**	7433	53636	10727	2,85	0,53
557	**SACOL0619**	2303	2195	439	-0,07	-2,39
558	**SACOL0620**	20777	18757	3751	-0,15	-2,47
559	**SACOL0621**	6195	3114	623	-0,99	-3,31
560	**SACOL0622**	11179	6581	1316	-0,76	-3,09
561	SACOL0623	8154	3487	697	-1,23	-3,55
562	SACOL0624	8356	3338	668	-1,32	-3,65
563	SACOL0625	10482	23213	4643	1,15	-1,17
564	**SACOL0626**	8522	12128	2426	0,51	-1,81
565	**SACOL0627**	11004	5158	1032	-1,09	-3,42
566	SACOL0628	7488	3996	799	-0,91	-3,23
567	SACOL0629	2362	1419	284	-0,74	-3,06
568	**SACOL0630**	6635	4128	826	-0,68	-3,01
569	SACOL0631	6140	8317	1663	0,44	-1,88
570	**SACOL0632**	2307	4091	818	0,83	-1,50
571	**SACOL0633**	15492	9760	1952	-0,67	-2,99
572	**SACOL0634**	26651	12170	2434	-1,13	-3,45
573	SACOL0635	18957	13748	2750	-0,46	-2,79
574	**SACOL0636**	36835	11542	2308	-1,67	-4,00
575	**SACOL0637**	30034	9644	1929	-1,64	-3,96
576	**SACOL0638**	23727	18257	3651	-0,38	-2,70

a) *S. aureus* COL Locus. Written in bold indicates that the corresponding protein was identified.
b) *LOWESS normalized background subtracted signal intensities.*
c) *Scaled stationary phase signal intensities to account for decrease in total RNA during stationary phase.*
d) *Log_2 of stationary phase versus exponential growth ratios.*
e) *Log_2 of scaled stationary phase versus exponential growth ratios.*

Supplementary Table 1: Signal intensities and calculated ratios from DNA microarray experiment

	SACOL[a]	signal intensity[b] exponential growth	signal intensity[b] stationary phase	signal intensity corrected[c] stationary phase	induction[d] stat/exp	induction corrected[e] stat/exp
577	SACOL0639	11241	3209	642	-1,81	-4,13
578	SACOL0640	2239	1652	330	-0,44	-2,76
579	SACOL0641	2422	1207	241	-1,01	-3,33
580	**SACOL0643**	4904	13594	2719	1,47	-0,85
581	**SACOL0644**	6798	7351	1470	0,11	-2,21
582	SACOL0645	9511	7752	1550	-0,29	-2,62
583	SACOL0646	11581	6147	1229	-0,91	-3,24
584	SACOL0647	7359	3046	609	-1,27	-3,59
585	SACOL0649	5644	2171	434	-1,38	-3,70
586	SACOL0650	3644	1408	282	-1,37	-3,69
587	SACOL0651	4203	1085	217	-1,95	-4,28
588	SACOL0652	5579	1890	378	-1,56	-3,88
589	SACOL0653	7726	2705	541	-1,51	-3,84
590	SACOL0654	5862	2577	515	-1,19	-3,51
591	**SACOL0655**	7517	12272	2454	0,71	-1,61
592	**SACOL0656**	14934	20456	4091	0,45	-1,87
593	**SACOL0658**	14379	10539	2108	-0,45	-2,77
594	**SACOL0659**	1152	1842	368	0,68	-1,64
595	**SACOL0660**	205	395	79	0,95	-1,38
596	**SACOL0662**	7192	7541	1508	0,07	-2,25
597	**SACOL0663**	8101	7245	1449	-0,16	-2,48
598	SACOL0664	628	1887	377	1,59	-0,73
599	**SACOL0665**	1346	2194	439	0,70	-1,62
600	SACOL0666	2802	5377	1075	0,94	-1,38
601	SACOL0667	3973	9125	1825	1,20	-1,12
602	**SACOL0668**	2832	7128	1426	1,33	-0,99
603	**SACOL0669**	10811	12661	2532	0,23	-2,09
604	**SACOL0670**	15379	11044	2209	-0,48	-2,80
605	**SACOL0671**	8277	93238	18648	3,49	1,17
606	**SACOL0672**	1791	3600	720	1,01	-1,32
607	SACOL0673	45678	139871	27974	1,61	-0,71
608	SACOL0675	4676	2549	510	-0,88	-3,20
609	SACOL0676	281	575	115	1,03	-1,29
610	SACOL0677	495	986	197	0,99	-1,33
611	**SACOL0678**	4886	30194	6039	2,63	0,31
612	**SACOL0679**	8166	26215	5243	1,68	-0,64
613	SACOL0680	8321	24222	4844	1,54	-0,78
614	SACOL0681	8620	27786	5557	1,69	-0,63
615	**SACOL0682**	8961	25355	5071	1,50	-0,82
616	**SACOL0684**	8389	19474	3895	1,21	-1,11
617	SACOL0685	7831	22807	4561	1,54	-0,78
618	SACOL0686	4010	10631	2126	1,41	-0,92
619	**SACOL0687**	15970	12753	2551	-0,32	-2,65
620	**SACOL0688**	1420	202	40	-2,81	-5,13
621	SACOL0689	2392	259	52	-3,21	-5,53
622	SACOL0690	1955	227	45	-3,11	-5,43
623	**SACOL0691**	15496	16186	3237	0,06	-2,26
624	SACOL0692	1935	2553	511	0,40	-1,92
625	**SACOL0693**	2994	3109	622	0,05	-2,27
626	**SACOL0694**	21348	20095	4019	-0,09	-2,41
627	**SACOL0695**	5483	3583	717	-0,61	-2,94
628	**SACOL0696**	11144	13371	2674	0,26	-2,06
629	**SACOL0697**	20501	22315	4463	0,12	-2,20
630	**SACOL0698**	6254	7254	1451	0,21	-2,11
631	**SACOL0699**	5675	2128	426	-1,42	-3,74
632	**SACOL0700**	9850	14442	2888	0,55	-1,77
633	**SACOL0701**	3926	1532	306	-1,36	-3,68
634	**SACOL0703**	12006	12242	2448	0,03	-2,29
635	**SACOL0704**	8509	6458	1292	-0,40	-2,72
636	SACOL0705	7425	5314	1063	-0,48	-2,80
637	**SACOL0706**	7429	3232	646	-1,20	-3,52
638	**SACOL0707**	2345	21147	4229	3,17	0,85
639	**SACOL0708**	2189	18285	3657	3,06	0,74
640	**SACOL0709**	2531	20538	4108	3,02	0,70
641	SACOL0710	14607	21161	4232	0,53	-1,79
642	**SACOL0711**	17445	13162	2632	-0,41	-2,73
643	**SACOL0712**	7389	2765	553	-1,42	-3,74
644	SACOL0713	3854	3818	764	-0,01	-2,34
645	**SACOL0714**	3277	12948	2590	1,98	-0,34
646	**SACOL0715**	5160	14333	2867	1,47	-0,85
647	**SACOL0716**	3662	7905	1581	1,11	-1,21
648	**SACOL0717**	5597	8570	1714	0,61	-1,71

a) *S. aureus* COL Locus. Written in bold indicates that the corresponding protein was identified.
b) *LOWESS normalized background subtracted signal intensities.*
c) *Scaled stationary phase signal intensities to account for decrease in total RNA during stationary phase.*
d) *Log_2 of stationary phase versus exponential growth ratios.*
e) *Log_2 of scaled stationary phase versus exponential growth ratios.*

Supplementary Table 1: Signal intensities and calculated ratios from DNA microarray experiment

	SACOL[a]	signal intensity[b] exponential growth	signal intensity[b] stationary phase	signal intensity corrected[c] stationary phase	induction[d] stat/exp	induction corrected[e] stat/exp
649	**SACOL0718**	12046	7862	1572	-0,62	-2,94
650	**SACOL0720**	9214	6171	1234	-0,58	-2,90
651	**SACOL0721**	12351	9801	1960	-0,33	-2,66
652	**SACOL0722**	10831	9607	1921	-0,17	-2,49
653	SACOL0723	9208	23521	4704	1,35	-0,97
654	SACOL0724	4992	6789	1358	0,44	-1,88
655	SACOL0725	3549	907	181	-1,97	-4,29
656	SACOL0726	944	190	38	-2,32	-4,64
657	**SACOL0727**	23205	15283	3057	-0,60	-2,92
658	**SACOL0728**	11977	5245	1049	-1,19	-3,51
659	**SACOL0730**	17887	35308	7062	0,98	-1,34
660	**SACOL0731**	19255	41657	8331	1,11	-1,21
661	**SACOL0733**	13691	20728	4146	0,60	-1,72
662	SACOL0734	7426	10503	2101	0,50	-1,82
663	**SACOL0735**	6879	4818	964	-0,51	-2,84
664	SACOL0736	2470	3010	602	0,29	-2,04
665	**SACOL0737**	20170	15383	3077	-0,39	-2,71
666	**SACOL0738**	4720	16557	3311	1,81	-0,51
667	**SACOL0739**	1433	2782	556	0,96	-1,36
668	**SACOL0740**	1885	4726	945	1,33	-1,00
669	SACOL0741	2987	9688	1938	1,70	-0,62
670	**SACOL0742**	6066	48732	9746	3,01	0,68
671	**SACOL0743**	8816	4122	824	-1,10	-3,42
672	**SACOL0744**	7655	3896	779	-0,97	-3,30
673	**SACOL0745**	7468	3458	692	-1,11	-3,43
674	**SACOL0746**	8476	4081	816	-1,05	-3,38
675	SACOL0747	6220	3858	772	-0,69	-3,01
676	**SACOL0748**	11961	6955	1391	-0,78	-3,10
677	SACOL0749	7886	4601	920	-0,78	-3,10
678	**SACOL0750**	8414	16272	3254	0,95	-1,37
679	SACOL0751	9623	23831	4766	1,31	-1,01
680	SACOL0752	4411	4820	964	0,13	-2,19
681	**SACOL0753**	2152	3871	774	0,85	-1,47
682	**SACOL0754**	5540	26323	5265	2,25	-0,07
683	**SACOL0755**	5988	14451	2890	1,27	-1,05
684	**SACOL0756**	3032	4714	943	0,64	-1,69
685	SACOL0757	7526	5546	1109	-0,44	-2,76
686	SACOL0758	7993	7854	1571	-0,03	-2,35
687	**SACOL0761**	13870	5772	1154	-1,26	-3,59
688	**SACOL0762**	12639	13883	2777	0,14	-2,19
689	**SACOL0763**	11277	32036	6407	1,51	-0,82
690	**SACOL0764**	12215	32624	6525	1,42	-0,90
691	**SACOL0765**	38521	63312	12662	0,72	-1,61
692	**SACOL0766**	33428	59565	11913	0,83	-1,49
693	SACOL0767	26513	46000	9200	0,79	-1,53
694	**SACOL0768**	3599	24345	4869	2,76	0,44
695	SACOL0769	12049	9403	1881	-0,36	-2,68
696	SACOL0770	4901	6752	1350	0,46	-1,86
697	SACOL0771	8087	10140	2028	0,33	-2,00
698	**SACOL0772**	7147	6746	1349	-0,08	-2,41
699	**SACOL0773**	38230	10530	2106	-1,86	-4,18
700	**SACOL0776**	30983	16251	3250	-0,93	-3,25
701	**SACOL0777**	50464	22592	4518	-1,16	-3,48
702	**SACOL0778**	10204	8541	1708	-0,26	-2,58
703	**SACOL0779**	7408	11594	2319	0,65	-1,68
704	SACOL0780	6008	6867	1373	0,19	-2,13
705	**SACOL0781**	9629	13954	2791	0,54	-1,79
706	**SACOL0783**	9101	13688	2738	0,59	-1,73
707	**SACOL0784**	5973	9454	1891	0,66	-1,66
708	**SACOL0785**	4440	6916	1383	0,64	-1,68
709	**SACOL0787**	3687	27248	5450	2,89	0,56
710	**SACOL0788**	50917	2919	584	-4,12	-6,45
711	**SACOL0789**	5151	1544	309	-1,74	-4,06
712	SACOL0790	15464	3950	790	-1,97	-4,29
713	SACOL0791	34837	32657	6531	-0,09	-2,42
714	**SACOL0792**	27344	23262	4652	-0,23	-2,56
715	**SACOL0793**	20809	12890	2578	-0,69	-3,01
716	SACOL0796	1174	187	37	-2,65	-4,97
717	SACOL0797	1177	303	61	-1,96	-4,28
718	SACOL0798	1897	523	105	-1,86	-4,18
719	**SACOL0799**	2692	1680	336	-0,68	-3,00
720	**SACOL0800**	15763	18428	3686	0,23	-2,10

a) *S. aureus* COL Locus. Written in bold indicates that the corresponding protein was identified.
b) *LOWESS normalized background subtracted signal intensities.*
c) *Scaled stationary phase signal intensities to account for decrease in total RNA during stationary phase.*
d) *Log_2 of stationary phase versus exponential growth ratios.*
e) *Log_2 of scaled stationary phase versus exponential growth ratios.*

Supplementary Table 1: Signal intensities and calculated ratios from DNA microarray experiment

	SACOL[a]	signal intensity[b] exponential growth	signal intensity[b] stationary phase	signal intensity corrected[c] stationary phase	induction[d] stat/exp	induction corrected[e] stat/exp
721	**SACOL0801**	11915	14315	2863	0,26	-2,06
722	SACOL0802	1569	539	108	-1,54	-3,86
723	**SACOL0803**	4212	5554	1111	0,40	-1,92
724	**SACOL0804**	9835	16430	3286	0,74	-1,58
725	**SACOL0805**	16013	24329	4866	0,60	-1,72
726	**SACOL0806**	15752	14195	2839	-0,15	-2,47
727	SACOL0807	6078	3739	748	-0,70	-3,02
728	**SACOL0808**	4535	903	181	-2,33	-4,65
729	**SACOL0809**	6222	2559	512	-1,28	-3,60
730	SACOL0810	23896	9520	1904	-1,33	-3,65
731	**SACOL0811**	7280	5235	1047	-0,48	-2,80
732	**SACOL0812**	10604	5333	1067	-0,99	-3,31
733	SACOL0813	3123	1485	297	-1,07	-3,39
734	SACOL0814	2920	2938	588	0,01	-2,31
735	**SACOL0815**	37846	29432	5886	-0,36	-2,68
736	**SACOL0816**	29396	13579	2716	-1,11	-3,44
737	**SACOL0818**	15711	7542	1508	-1,06	-3,38
738	SACOL0820	486	315	63	-0,62	-2,95
739	**SACOL0821**	3110	7369	1474	1,24	-1,08
740	SACOL0822	1255	2551	510	1,02	-1,30
741	**SACOL0823**	19944	19843	3969	-0,01	-2,33
742	**SACOL0824**	12547	22570	4514	0,85	-1,47
743	**SACOL0825**	11663	14480	2896	0,31	-2,01
744	**SACOL0826**	25872	23918	4784	-0,11	-2,44
745	SACOL0827	16329	9727	1945	-0,75	-3,07
746	**SACOL0828**	26977	14981	2996	-0,85	-3,17
747	**SACOL0829**	31655	37556	7511	0,25	-2,08
748	**SACOL0830**	15873	246195	49239	3,96	1,63
749	**SACOL0831**	5472	42305	8461	2,95	0,63
750	**SACOL0832**	6178	32039	6408	2,37	0,05
751	**SACOL0833**	10004	7686	1537	-0,38	-2,70
752	**SACOL0834**	4496	15603	3121	1,80	-0,53
753	SACOL0835	37050	113615	22723	1,62	-0,71
754	**SACOL0837**	58531	45805	9161	-0,35	-2,68
755	**SACOL0838**	51474	33703	6741	-0,61	-2,93
756	**SACOL0839**	53158	54149	10830	0,03	-2,30
757	**SACOL0840**	45853	43798	8760	-0,07	-2,39
758	**SACOL0841**	47375	49096	9819	0,05	-2,27
759	**SACOL0842**	5302	7426	1485	0,49	-1,84
760	**SACOL0843**	18722	13957	2791	-0,42	-2,75
761	**SACOL0844**	83530	62491	12498	-0,42	-2,74
762	**SACOL0845**	48353	22248	4450	-1,12	-3,44
763	**SACOL0846**	30250	32206	6441	0,09	-2,23
764	**SACOL0847**	22147	18255	3651	-0,28	-2,60
765	SACOL0848	490	172	34	-1,51	-3,83
766	**SACOL0849**	2318	3220	644	0,47	-1,85
767	SACOL0850	2894	4136	827	0,52	-1,81
768	**SACOL0851**	11492	13115	2623	0,19	-2,13
769	SACOL0853	151	317	63	1,07	-1,25
770	SACOL0854	1305	12616	2523	3,27	0,95
771	**SACOL0855**	4477	13134	2627	1,55	-0,77
772	**SACOL0856**	5459	72766	14553	3,74	1,41
773	SACOL0857	977	1116	223	0,19	-2,13
774	SACOL0858	298	371	74	0,32	-2,00
775	**SACOL0859**	1630	344	69	-2,25	-4,57
776	**SACOL0860**	4307	1691	338	-1,35	-3,67
777	**SACOL0861**	38856	6789	1358	-2,52	-4,84
778	SACOL0862	3390	13450	2690	1,99	-0,33
779	SACOL0863	3842	14159	2832	1,88	-0,44
780	SACOL0864	3529	1316	263	-1,42	-3,74
781	SACOL0865	11595	20390	4078	0,81	-1,51
782	SACOL0866	20534	289914	57983	3,82	1,50
783	SACOL0869	1001	782	156	-0,36	-2,68
784	**SACOL0870**	5965	10499	2100	0,82	-1,51
785	**SACOL0871**	8495	41095	8219	2,27	-0,05
786	**SACOL0872**	2668	41497	8299	3,96	1,64
787	SACOL0873	2552	4746	949	0,90	-1,43
788	**SACOL0874**	9600	9060	1812	-0,08	-2,41
789	**SACOL0875**	5296	8467	1693	0,68	-1,65
790	**SACOL0876**	10514	18104	3621	0,78	-1,54
791	**SACOL0877**	8393	18886	3777	1,17	-1,15
792	**SACOL0879**	5444	6631	1326	0,28	-2,04

a) *S. aureus* COL Locus. Written in bold indicates that the corresponding protein was identified.
b) *LOWESS normalized background subtracted signal intensities.*
c) *Scaled stationary phase signal intensities to account for decrease in total RNA during stationary phase.*
d) *Log_2 of stationary phase versus exponential growth ratios.*
e) *Log_2 of scaled stationary phase versus exponential growth ratios.*

Supplementary Table 1: Signal intensities and calculated ratios from DNA microarray experiment

	SACOL[a]	signal intensity[b] exponential growth	signal intensity[b] stationary phase	signal intensity corrected[c] stationary phase	induction[d] stat/exp	induction corrected[e] stat/exp
793	SACOL0880	3600	10378	2076	1,53	-0,79
794	**SACOL0881**	3210	7289	1458	1,18	-1,14
795	**SACOL0882**	16526	8643	1729	-0,94	-3,26
796	**SACOL0883**	13574	9383	1877	-0,53	-2,85
797	**SACOL0884**	3675	3077	615	-0,26	-2,58
798	SACOL0885	1412	1324	265	-0,09	-2,41
799	**SACOL0886**	3181	2718	544	-0,23	-2,55
800	**SACOL0887**	1977	2129	426	0,11	-2,21
801	**SACOL0888**	2708	4089	818	0,59	-1,73
802	SACOL0889	4821	6286	1257	0,38	-1,94
803	**SACOL0890**	4471	9448	1890	1,08	-1,24
804	SACOL0892	1539	2693	539	0,81	-1,52
805	SACOL0893	839	1802	360	1,10	-1,22
806	SACOL0895	99	138	28	0,48	-1,84
807	SACOL0896	2225	3170	634	0,51	-1,81
808	SACOL0897	1600	2720	544	0,77	-1,56
809	SACOL0898	2049	5374	1075	1,39	-0,93
810	SACOL0899	1259	3361	672	1,42	-0,91
811	SACOL0900	1673	5673	1135	1,76	-0,56
812	SACOL0901	5434	9464	1893	0,80	-1,52
813	SACOL0902	5498	5783	1157	0,07	-2,25
814	SACOL0903	4576	5446	1089	0,25	-2,07
815	SACOL0904	3663	5564	1113	0,60	-1,72
816	SACOL0905	3386	5193	1039	0,62	-1,70
817	**SACOL0907**	1069	10807	2161	3,34	1,02
818	**SACOL0908**	3126	3288	658	0,07	-2,25
819	SACOL0909	775	1410	282	0,86	-1,46
820	SACOL0911	836	1031	206	0,30	-2,02
821	SACOL0913	1266	1902	380	0,59	-1,73
822	**SACOL0914**	41921	44567	8913	0,09	-2,23
823	**SACOL0915**	42725	48684	9737	0,19	-2,13
824	**SACOL0916**	47252	51776	10355	0,13	-2,19
825	**SACOL0917**	43685	43405	8681	-0,01	-2,33
826	**SACOL0918**	20256	27558	5512	0,44	-1,88
827	SACOL0919	2249	1375	275	-0,71	-3,03
828	SACOL0920	168	454	91	1,43	-0,89
829	**SACOL0921**	9167	46538	9308	2,34	0,02
830	**SACOL0922**	11024	24424	4885	1,15	-1,17
831	**SACOL0924**	30931	15061	3012	-1,04	-3,36
832	**SACOL0925**	17934	6932	1386	-1,37	-3,69
833	**SACOL0926**	30012	11506	2301	-1,38	-3,71
834	**SACOL0927**	14526	16505	3301	0,18	-2,14
835	**SACOL0928**	3180	2939	588	-0,11	-2,44
836	**SACOL0929**	2021	1731	346	-0,22	-2,55
837	**SACOL0930**	23290	10363	2073	-1,17	-3,49
838	**SACOL0931**	18578	11619	2324	-0,68	-3,00
839	**SACOL0932**	17268	7637	1527	-1,18	-3,50
840	SACOL0934	74662	16856	3371	-2,15	-4,47
841	**SACOL0935**	24402	5345	1069	-2,19	-4,51
842	**SACOL0936**	35403	7636	1527	-2,21	-4,53
843	**SACOL0937**	46861	8957	1791	-2,39	-4,71
844	**SACOL0938**	23447	4922	984	-2,25	-4,57
845	**SACOL0939**	5238	17093	3419	1,71	-0,62
846	**SACOL0940**	6726	7915	1583	0,23	-2,09
847	SACOL0942	5888	17094	3419	1,54	-0,78
848	**SACOL0943**	8969	18033	3607	1,01	-1,31
849	**SACOL0944**	17345	10317	2063	-0,75	-3,07
850	**SACOL0945**	2878	2542	508	-0,18	-2,50
851	**SACOL0946**	3128	2763	553	-0,18	-2,50
852	SACOL0947	4778	6327	1265	0,41	-1,92
853	SACOL0948	464	102	20	-2,18	-4,50
854	**SACOL0949**	6983	7883	1577	0,17	-2,15
855	SACOL0950	6296	6583	1317	0,06	-2,26
856	**SACOL0951**	15246	13570	2714	-0,17	-2,49
857	**SACOL0952**	23828	19387	3877	-0,30	-2,62
858	**SACOL0953**	50753	34247	6849	-0,57	-2,89
859	**SACOL0954**	48402	33575	6715	-0,53	-2,85
860	**SACOL0955**	47057	33853	6771	-0,48	-2,80
861	**SACOL0956**	6362	5753	1151	-0,15	-2,47
862	**SACOL0957**	2639	5281	1056	1,00	-1,32
863	**SACOL0958**	3398	5576	1115	0,71	-1,61
864	**SACOL0959**	3601	10741	2148	1,58	-0,75

a) *S. aureus* COL Locus. Written in bold indicates that the corresponding protein was identified.
b) *LOWESS normalized background subtracted signal intensities.*
c) *Scaled stationary phase signal intensities to account for decrease in total RNA during stationary phase.*
d) *Log_2 of stationary phase versus exponential growth ratios.*
e) *Log_2 of scaled stationary phase versus exponential growth ratios.*

Supplementary Table 1: Signal intensities and calculated ratios from DNA microarray experiment

	SACOL[a]	signal intensity[b] exponential growth	signal intensity[b] stationary phase	signal intensity corrected[c] stationary phase	induction[d] stat/exp	induction corrected[e] stat/exp
865	**SACOL0960**	9952	100840	20168	3,34	1,02
866	**SACOL0961**	12891	87536	17507	2,76	0,44
867	**SACOL0962**	2512	9220	1844	1,88	-0,45
868	SACOL0963	181	300	60	0,73	-1,59
869	SACOL0964	610	957	191	0,65	-1,67
870	SACOL0965	59	65	13	0,14	-2,18
871	**SACOL0966**	6741	6743	1349	0,00	-2,32
872	SACOL0967	7565	4367	873	-0,79	-3,11
873	**SACOL0968**	12938	5735	1147	-1,17	-3,50
874	**SACOL0969**	7483	4629	926	-0,69	-3,01
875	**SACOL0970**	6184	10796	2159	0,80	-1,52
876	**SACOL0971**	7496	13295	2659	0,83	-1,50
877	**SACOL0973**	11087	20622	4124	0,90	-1,43
878	SACOL0974	2696	2063	413	-0,39	-2,71
879	**SACOL0975**	22673	19602	3920	-0,21	-2,53
880	**SACOL0976**	35279	33410	6682	-0,08	-2,40
881	SACOL0977	63939	77834	15567	0,28	-2,04
882	SACOL0978	528	154	31	-1,78	-4,10
883	**SACOL0979**	12656	18580	3716	0,55	-1,77
884	SACOL0980	256	156	31	-0,71	-3,04
885	SACOL0981	239	155	31	-0,63	-2,95
886	SACOL0982	469	446	89	-0,07	-2,39
887	SACOL0983	3170	1583	317	-1,00	-3,32
888	**SACOL0984**	3058	2165	433	-0,50	-2,82
889	**SACOL0985**	12299	40710	8142	1,73	-0,60
890	SACOL0986	3817	5433	1087	0,51	-1,81
891	**SACOL0987**	41681	6209	1242	-2,75	-5,07
892	**SACOL0988**	45026	12912	2582	-1,80	-4,12
893	SACOL0989	1130	785	157	-0,53	-2,85
894	**SACOL0991**	41741	23387	4677	-0,84	-3,16
895	**SACOL0992**	54857	35373	7075	-0,63	-2,95
896	**SACOL0993**	38957	30963	6193	-0,33	-2,65
897	**SACOL0994**	61495	41041	8208	-0,58	-2,91
898	**SACOL0995**	61421	44148	8830	-0,48	-2,80
899	**SACOL0996**	555	571	114	0,04	-2,28
900	SACOL0997	554	936	187	0,76	-1,56
901	SACOL0998	1058	1485	297	0,49	-1,83
902	SACOL0999	862	1360	272	0,66	-1,66
903	SACOL1000	1303	2343	469	0,85	-1,48
904	**SACOL1001**	10014	12819	2564	0,36	-1,97
905	**SACOL1002**	13533	21187	4237	0,65	-1,68
906	**SACOL1003**	13006	8995	1799	-0,53	-2,85
907	SACOL1004	1265	544	109	-1,22	-3,54
908	**SACOL1005**	4771	14170	2834	1,57	-0,75
909	**SACOL1006**	7719	24452	4890	1,66	-0,66
910	SACOL1007	13969	40125	8025	1,52	-0,80
911	**SACOL1008**	2749	4980	996	0,86	-1,46
912	**SACOL1009**	11132	14067	2813	0,34	-1,98
913	**SACOL1010**	11707	13610	2722	0,22	-2,10
914	**SACOL1011**	9546	13732	2746	0,52	-1,80
915	SACOL1012	15990	7458	1492	-1,10	-3,42
916	**SACOL1013**	30551	14234	2847	-1,10	-3,42
917	**SACOL1016**	23407	11992	2398	-0,96	-3,29
918	**SACOL1017**	12709	8083	1617	-0,65	-2,97
919	**SACOL1018**	21952	7595	1519	-1,53	-3,85
920	SACOL1019	8953	5332	1066	-0,75	-3,07
921	**SACOL1020**	13926	31031	6206	1,16	-1,17
922	**SACOL1021**	12112	9963	1993	-0,28	-2,60
923	**SACOL1022**	12280	11907	2381	-0,04	-2,37
924	**SACOL1023**	19703	9272	1854	-1,09	-3,41
925	SACOL1024	19268	9027	1805	-1,09	-3,42
926	**SACOL1025**	2502	1198	240	-1,06	-3,38
927	**SACOL1026**	34316	8976	1795	-1,93	-4,26
928	**SACOL1028**	2012	1785	357	-0,17	-2,50
929	**SACOL1030**	1989	1450	290	-0,46	-2,78
930	**SACOL1031**	6594	11470	2294	0,80	-1,52
931	SACOL1032	1512	1819	364	0,27	-2,06
932	SACOL1033	9921	126886	25377	3,68	1,36
933	**SACOL1034**	8937	6558	1312	-0,45	-2,77
934	SACOL1035	22497	9442	1888	-1,25	-3,57
935	**SACOL1036**	5022	2996	599	-0,75	-3,07
936	SACOL1038	1105	2150	430	0,96	-1,36

a) *S. aureus* COL Locus. Written in bold indicates that the corresponding protein was identified.
b) *LOWESS normalized background subtracted signal intensities.*
c) *Scaled stationary phase signal intensities to account for decrease in total RNA during stationary phase.*
d) *Log_2 of stationary phase versus exponential growth ratios.*
e) *Log_2 of scaled stationary phase versus exponential growth ratios.*

Supplementary Table 1: Signal intensities and calculated ratios from DNA microarray experiment

	SACOL[a]	signal intensity[b] exponential growth	signal intensity[b] stationary phase	signal intensity corrected[c] stationary phase	induction[d] stat/exp	induction corrected[e] stat/exp
937	SACOL1039	706	1251	250	0,82	-1,50
938	SACOL1040	647	1061	212	0,72	-1,61
939	SACOL1041	2834	15169	3034	2,42	0,10
940	**SACOL1042**	7481	1339	268	-2,48	-4,80
941	**SACOL1043**	7147	1458	292	-2,29	-4,62
942	SACOL1044	2742	712	142	-1,94	-4,27
943	**SACOL1045**	2842	1556	311	-0,87	-3,19
944	**SACOL1047**	2801	3553	711	0,34	-1,98
945	**SACOL1048**	6390	16229	3246	1,34	-0,98
946	**SACOL1049**	13438	4698	940	-1,52	-3,84
947	SACOL1050	28893	13318	2664	-1,12	-3,44
948	**SACOL1051**	28772	11486	2297	-1,32	-3,65
949	**SACOL1052**	26353	10971	2194	-1,26	-3,59
950	**SACOL1053**	24738	10155	2031	-1,28	-3,61
951	**SACOL1054**	8163	6952	1390	-0,23	-2,55
952	SACOL1055	1473	2458	492	0,74	-1,58
953	**SACOL1056**	3041	4339	868	0,51	-1,81
954	SACOL1057	3955	2657	531	-0,57	-2,90
955	**SACOL1058**	10522	4489	898	-1,23	-3,55
956	**SACOL1059**	8411	2564	513	-1,71	-4,04
957	SACOL1060	582	3122	624	2,42	0,10
958	**SACOL1062**	7708	47751	9550	2,63	0,31
959	**SACOL1063**	3174	3168	634	0,00	-2,32
960	SACOL1064	4928	4281	856	-0,20	-2,52
961	**SACOL1065**	13974	8481	1696	-0,72	-3,04
962	**SACOL1066**	8445	3641	728	-1,21	-3,54
963	**SACOL1067**	5504	4643	929	-0,25	-2,57
964	**SACOL1068**	43558	17129	3426	-1,35	-3,67
965	**SACOL1069**	48652	19413	3883	-1,33	-3,65
966	**SACOL1070**	47081	21040	4208	-1,16	-3,48
967	SACOL1071	13589	13924	2785	0,04	-2,29
968	**SACOL1072**	14791	37050	7410	1,32	-1,00
969	**SACOL1073**	2775	25401	5080	3,19	0,87
970	**SACOL1074**	2817	29382	5876	3,38	1,06
971	**SACOL1075**	4711	34827	6965	2,89	0,56
972	**SACOL1076**	2648	23756	4751	3,17	0,84
973	**SACOL1077**	2569	19627	3925	2,93	0,61
974	**SACOL1078**	3705	28408	5682	2,94	0,62
975	**SACOL1079**	3768	25792	5158	2,78	0,45
976	**SACOL1080**	4804	26150	5230	2,44	0,12
977	**SACOL1081**	5101	31094	6219	2,61	0,29
978	**SACOL1082**	7659	41953	8391	2,45	0,13
979	**SACOL1083**	2946	14361	2872	2,29	-0,04
980	SACOL1084	4482	8529	1706	0,93	-1,39
981	**SACOL1085**	17656	29403	5881	0,74	-1,59
982	**SACOL1086**	19297	14938	2988	-0,37	-2,69
983	**SACOL1088**	5866	9115	1823	0,64	-1,69
984	**SACOL1089**	13422	11682	2336	-0,20	-2,52
985	**SACOL1090**	5909	16658	3332	1,50	-0,83
986	**SACOL1091**	14637	23836	4767	0,70	-1,62
987	**SACOL1092**	8888	16977	3395	0,93	-1,39
988	SACOL1093	468	919	184	0,97	-1,35
989	**SACOL1094**	9855	2193	439	-2,17	-4,49
990	SACOL1095	9248	2165	433	-2,09	-4,42
991	**SACOL1096**	21524	41788	8358	0,96	-1,36
992	**SACOL1098**	26794	34639	6928	0,37	-1,95
993	**SACOL1099**	52176	37511	7502	-0,48	-2,80
994	**SACOL1100**	6720	3911	782	-0,78	-3,10
995	**SACOL1101**	7504	7106	1421	-0,08	-2,40
996	**SACOL1102**	76557	49309	9862	-0,63	-2,96
997	**SACOL1103**	87104	69269	13854	-0,33	-2,65
998	**SACOL1104**	51281	43216	8643	-0,25	-2,57
999	**SACOL1105**	16482	13990	2798	-0,24	-2,56
1000	**SACOL1106**	9337	5055	1011	-0,89	-3,21
1001	**SACOL1107**	10437	2074	415	-2,33	-4,65
1002	**SACOL1108**	7942	1611	322	-2,30	-4,62
1003	**SACOL1109**	11162	1822	364	-2,62	-4,94
1004	**SACOL1110**	14436	1865	373	-2,95	-5,27
1005	**SACOL1111**	17412	3949	790	-2,14	-4,46
1006	**SACOL1112**	19504	42345	8469	1,12	-1,20
1007	**SACOL1113**	5211	14412	2882	1,47	-0,85
1008	**SACOL1114**	37595	2626	525	-3,84	-6,16

a) *S. aureus* COL Locus. Written in bold indicates that the corresponding protein was identified.
b) *LOWESS normalized background subtracted signal intensities.*
c) *Scaled stationary phase signal intensities to account for decrease in total RNA during stationary phase.*
d) *Log_2 of stationary phase versus exponential growth ratios.*
e) *Log_2 of scaled stationary phase versus exponential growth ratios.*

Supplementary Table 1: Signal intensities and calculated ratios from DNA microarray experiment

	SACOL[a]	signal intensity[b] exponential growth	signal intensity[b] stationary phase	signal intensity corrected[c] stationary phase	induction[d] stat/exp	induction corrected[e] stat/exp
1009	**SACOL1115**	4257	5538	1108	0,38	-1,94
1010	**SACOL1116**	38622	32773	6555	-0,24	-2,56
1011	**SACOL1118**	7087	759	152	-3,22	-5,55
1012	SACOL1119	2912	5516	1103	0,92	-1,40
1013	**SACOL1120**	9668	13440	2688	0,48	-1,85
1014	SACOL1121	8668	5241	1048	-0,73	-3,05
1015	**SACOL1122**	18171	7622	1524	-1,25	-3,58
1016	**SACOL1123**	7231	5398	1080	-0,42	-2,74
1017	**SACOL1124**	8366	4099	820	-1,03	-3,35
1018	**SACOL1125**	35963	19709	3942	-0,87	-3,19
1019	**SACOL1126**	29304	14910	2982	-0,97	-3,30
1020	**SACOL1128**	12377	21722	4344	0,81	-1,51
1021	SACOL1129	2037	6426	1285	1,66	-0,66
1022	**SACOL1130**	4896	3573	715	-0,45	-2,78
1023	SACOL1131	11223	7809	1562	-0,52	-2,85
1024	SACOL1132	3540	2042	408	-0,79	-3,12
1025	**SACOL1133**	9586	7690	1538	-0,32	-2,64
1026	**SACOL1134**	7124	3051	610	-1,22	-3,55
1027	**SACOL1135**	1270	2129	426	0,74	-1,58
1028	**SACOL1136**	130910	10619	2124	-3,62	-5,95
1029	SACOL1137	93617	14694	2939	-2,67	-4,99
1030	SACOL1138	224	399	80	0,83	-1,49
1031	**SACOL1140**	3588	1000	200	-1,84	-4,16
1032	SACOL1141	717	251	50	-1,51	-3,83
1033	**SACOL1142**	1145	394	79	-1,54	-3,86
1034	SACOL1143	941	295	59	-1,67	-4,00
1035	SACOL1144	2252	1147	229	-0,97	-3,30
1036	SACOL1145	1623	660	132	-1,30	-3,62
1037	SACOL1146	2844	1345	269	-1,08	-3,40
1038	**SACOL1147**	3311	3066	613	-0,11	-2,43
1039	**SACOL1148**	19197	4416	883	-2,12	-4,44
1040	**SACOL1149**	35276	9291	1858	-1,92	-4,25
1041	**SACOL1150**	3174	1350	270	-1,23	-3,56
1042	SACOL1151	3266	4401	880	0,43	-1,89
1043	**SACOL1152**	2672	3683	737	0,46	-1,86
1044	**SACOL1153**	12345	9549	1910	-0,37	-2,69
1045	**SACOL1154**	10262	7970	1594	-0,36	-2,69
1046	**SACOL1155**	22587	37548	7510	0,73	-1,59
1047	SACOL1156	210	146	29	-0,53	-2,85
1048	**SACOL1157**	11847	26659	5332	1,17	-1,15
1049	**SACOL1158**	35602	124194	24839	1,80	-0,52
1050	**SACOL1159**	20606	88747	17749	2,11	-0,22
1051	**SACOL1160**	2579	14440	2888	2,49	0,16
1052	**SACOL1161**	4585	23342	4668	2,35	0,03
1053	**SACOL1162**	5665	23261	4652	2,04	-0,28
1054	**SACOL1163**	5295	16007	3201	1,60	-0,73
1055	**SACOL1164**	3900	5190	1038	0,41	-1,91
1056	SACOL1165	3529	3638	728	0,04	-2,28
1057	**SACOL1166**	100	265	53	1,41	-0,92
1058	SACOL1167	8582	2923	585	-1,55	-3,88
1059	**SACOL1168**	10715	15123	3025	0,50	-1,82
1060	SACOL1169	4784	8922	1784	0,90	-1,42
1061	SACOL1171	2144	5572	1114	1,38	-0,94
1062	SACOL1172	4048	5798	1160	0,52	-1,80
1063	SACOL1173	676	730	146	0,11	-2,21
1064	SACOL1175	1205	1039	208	-0,21	-2,54
1065	SACOL1176	14536	58620	11724	2,01	-0,31
1066	SACOL1178	107	74	15	-0,54	-2,86
1067	SACOL1179	194	127	25	-0,62	-2,94
1068	SACOL1180	246	192	38	-0,36	-2,68
1069	SACOL1181	523	571	114	0,13	-2,20
1070	SACOL1182	493	1129	226	1,20	-1,13
1071	**SACOL1183**	2207	5209	1042	1,24	-1,08
1072	SACOL1184	180	64	13	-1,49	-3,81
1073	SACOL1185	2871	1009	202	-1,51	-3,83
1074	SACOL1187	799	1350	270	0,76	-1,56
1075	**SACOL1188**	1538	3602	720	1,23	-1,09
1076	**SACOL1189**	19809	89825	17965	2,18	-0,14
1077	**SACOL1190**	11743	9634	1927	-0,29	-2,61
1078	**SACOL1191**	45577	58959	11792	0,37	-1,95
1079	**SACOL1192**	37180	57099	11420	0,62	-1,70
1080	**SACOL1193**	37769	43342	8668	0,20	-2,12

a) *S. aureus* COL Locus. Written in bold indicates that the corresponding protein was identified.
b) *LOWESS normalized background subtracted signal intensities.*
c) *Scaled stationary phase signal intensities to account for decrease in total RNA during stationary phase.*
d) *Log_2 of stationary phase versus exponential growth ratios.*
e) *Log_2 of scaled stationary phase versus exponential growth ratios.*

Supplementary Table 1: Signal intensities and calculated ratios from DNA microarray experiment

	SACOL[a]	signal intensity[b] exponential growth	signal intensity[b] stationary phase	signal intensity corrected[c] stationary phase	induction[d] stat/exp	induction corrected[e] stat/exp
1081	**SACOL1194**	14796	4285	857	-1,79	-4,11
1082	**SACOL1195**	42159	5971	1194	-2,82	-5,14
1083	**SACOL1196**	51338	9295	1859	-2,47	-4,79
1084	**SACOL1197**	29781	11645	2329	-1,35	-3,68
1085	**SACOL1198**	27667	27779	5556	0,01	-2,32
1086	**SACOL1199**	21744	27171	5434	0,32	-2,00
1087	**SACOL1200**	17030	32276	6455	0,92	-1,40
1088	**SACOL1201**	41520	53521	10704	0,37	-1,96
1089	**SACOL1202**	10011	15004	3001	0,58	-1,74
1090	**SACOL1203**	11703	14442	2888	0,30	-2,02
1091	**SACOL1204**	22310	21734	4347	-0,04	-2,36
1092	**SACOL1205**	23103	22786	4557	-0,02	-2,34
1093	**SACOL1206**	14998	2533	507	-2,57	-4,89
1094	**SACOL1207**	7921	6545	1309	-0,28	-2,60
1095	**SACOL1208**	4679	2610	522	-0,84	-3,16
1096	**SACOL1209**	6644	3466	693	-0,94	-3,26
1097	**SACOL1210**	14452	3335	667	-2,12	-4,44
1098	SACOL1211	11154	1257	251	-3,15	-5,47
1099	**SACOL1212**	13093	1759	352	-2,90	-5,22
1100	**SACOL1213**	17925	2978	596	-2,59	-4,91
1101	**SACOL1214**	9072	1868	374	-2,28	-4,60
1102	**SACOL1215**	10389	2096	419	-2,31	-4,63
1103	**SACOL1216**	8404	2721	544	-1,63	-3,95
1104	**SACOL1217**	6645	2380	476	-1,48	-3,80
1105	SACOL1218	2903	3285	657	0,18	-2,14
1106	**SACOL1219**	4291	6750	1350	0,65	-1,67
1107	**SACOL1220**	16015	10507	2101	-0,61	-2,93
1108	**SACOL1221**	18822	21863	4373	0,22	-2,11
1109	**SACOL1222**	23006	25062	5012	0,12	-2,20
1110	**SACOL1223**	13514	25099	5020	0,89	-1,43
1111	**SACOL1224**	25411	32036	6407	0,33	-1,99
1112	**SACOL1225**	4990	3587	717	-0,48	-2,80
1113	SACOL1226	13328	148228	29646	3,48	1,15
1114	**SACOL1227**	20928	14564	2913	-0,52	-2,84
1115	**SACOL1228**	33978	28062	5612	-0,28	-2,60
1116	**SACOL1229**	44708	24592	4918	-0,86	-3,18
1117	**SACOL1230**	32651	15449	3090	-1,08	-3,40
1118	**SACOL1231**	38100	18056	3611	-1,08	-3,40
1119	**SACOL1234**	9489	12941	2588	0,45	-1,87
1120	SACOL1235	8303	13317	2663	0,68	-1,64
1121	**SACOL1236**	1954	3229	646	0,72	-1,60
1122	**SACOL1238**	5165	10633	2127	1,04	-1,28
1123	**SACOL1239**	41723	29036	5807	-0,52	-2,84
1124	**SACOL1240**	23960	16301	3260	-0,56	-2,88
1125	**SACOL1241**	8213	14380	2876	0,81	-1,51
1126	**SACOL1242**	30095	12186	2437	-1,30	-3,63
1127	**SACOL1243**	26200	10949	2190	-1,26	-3,58
1128	**SACOL1244**	23802	11950	2390	-0,99	-3,32
1129	**SACOL1245**	29860	13182	2636	-1,18	-3,50
1130	**SACOL1247**	19623	7397	1479	-1,41	-3,73
1131	**SACOL1248**	31092	15889	3178	-0,97	-3,29
1132	**SACOL1250**	33265	17568	3514	-0,92	-3,24
1133	**SACOL1251**	48281	17981	3596	-1,43	-3,75
1134	**SACOL1252**	43438	17550	3510	-1,31	-3,63
1135	**SACOL1253**	21917	16213	3243	-0,43	-2,76
1136	**SACOL1254**	10450	2988	598	-1,81	-4,13
1137	**SACOL1255**	10964	3554	711	-1,63	-3,95
1138	SACOL1256	14880	3913	783	-1,93	-4,25
1139	**SACOL1257**	6565	2712	542	-1,28	-3,60
1140	**SACOL1259**	8504	15860	3172	0,90	-1,42
1141	**SACOL1260**	10328	6272	1254	-0,72	-3,04
1142	**SACOL1261**	11469	6818	1364	-0,75	-3,07
1143	**SACOL1262**	28672	143852	28770	2,33	0,00
1144	**SACOL1263**	13793	76550	15310	2,47	0,15
1145	SACOL1264	1436	2203	441	0,62	-1,71
1146	SACOL1265	2547	1862	372	-0,45	-2,77
1147	SACOL1266	1807	1274	255	-0,50	-2,83
1148	**SACOL1267**	35857	15840	3168	-1,18	-3,50
1149	**SACOL1268**	30174	13738	2748	-1,14	-3,46
1150	**SACOL1269**	29656	38873	7775	0,39	-1,93
1151	**SACOL1270**	44659	50983	10197	0,19	-2,13
1152	**SACOL1271**	46614	52647	10529	0,18	-2,15

a) *S. aureus* COL Locus. Written in bold indicates that the corresponding protein was identified.
b) *LOWESS normalized background subtracted signal intensities.*
c) *Scaled stationary phase signal intensities to account for decrease in total RNA during stationary phase.*
d) *Log_2 of stationary phase versus exponential growth ratios.*
e) *Log_2 of scaled stationary phase versus exponential growth ratios.*

Supplementary Table 1: Signal intensities and calculated ratios from DNA microarray experiment

	SACOL[a]	signal intensity[b] exponential growth	signal intensity[b] stationary phase	signal intensity corrected[c] stationary phase	induction[d] stat/exp	induction corrected[e] stat/exp
1153	**SACOL1272**	35773	39051	7810	0,13	-2,20
1154	**SACOL1274**	69078	10451	2090	-2,72	-5,05
1155	**SACOL1276**	14762	7989	1598	-0,89	-3,21
1156	**SACOL1277**	27135	13739	2748	-0,98	-3,30
1157	**SACOL1278**	18793	9257	1851	-1,02	-3,34
1158	**SACOL1279**	22250	5858	1172	-1,93	-4,25
1159	**SACOL1280**	19288	3202	640	-2,59	-4,91
1160	**SACOL1281**	17396	7674	1535	-1,18	-3,50
1161	**SACOL1282**	12686	6012	1202	-1,08	-3,40
1162	**SACOL1283**	21714	17320	3464	-0,33	-2,65
1163	**SACOL1284**	46532	36314	7263	-0,36	-2,68
1164	**SACOL1285**	34999	32088	6418	-0,13	-2,45
1165	**SACOL1286**	47869	52556	10511	0,13	-2,19
1166	**SACOL1287**	53389	58852	11770	0,14	-2,18
1167	**SACOL1288**	18019	21492	4298	0,25	-2,07
1168	**SACOL1290**	10447	30296	6059	1,54	-0,79
1169	**SACOL1291**	8221	23003	4601	1,48	-0,84
1170	**SACOL1292**	28053	16574	3315	-0,76	-3,08
1171	**SACOL1293**	57979	24060	4812	-1,27	-3,59
1172	**SACOL1294**	30137	27325	5465	-0,14	-2,46
1173	**SACOL1295**	18415	22438	4488	0,29	-2,04
1174	**SACOL1296**	35707	9251	1850	-1,95	-4,27
1175	**SACOL1297**	21116	4217	843	-2,32	-4,65
1176	**SACOL1298**	25898	6879	1376	-1,91	-4,23
1177	**SACOL1299**	32684	6972	1394	-2,23	-4,55
1178	**SACOL1300**	33478	17503	3501	-0,94	-3,26
1179	**SACOL1301**	14853	8685	1737	-0,77	-3,10
1180	**SACOL1302**	22971	12960	2592	-0,83	-3,15
1181	**SACOL1303**	9820	8989	1798	-0,13	-2,45
1182	**SACOL1304**	14646	18214	3643	0,31	-2,01
1183	**SACOL1305**	7029	7404	1481	0,08	-2,25
1184	**SACOL1307**	18230	8890	1778	-1,04	-3,36
1185	**SACOL1308**	20535	37404	7481	0,87	-1,46
1186	**SACOL1309**	20256	44919	8984	1,15	-1,17
1187	**SACOL1310**	10000	12569	2514	0,33	-1,99
1188	**SACOL1312**	12401	13021	2604	0,07	-2,25
1189	SACOL1313	4573	5153	1031	0,17	-2,15
1190	**SACOL1314**	3487	3492	698	0,00	-2,32
1191	**SACOL1315**	13351	7855	1571	-0,77	-3,09
1192	**SACOL1316**	22154	6853	1371	-1,69	-4,01
1193	**SACOL1317**	6557	2207	441	-1,57	-3,89
1194	**SACOL1319**	14182	11486	2297	-0,30	-2,63
1195	**SACOL1320**	2445	3579	716	0,55	-1,77
1196	**SACOL1321**	15805	16192	3238	0,03	-2,29
1197	**SACOL1322**	5588	6144	1229	0,14	-2,19
1198	**SACOL1323**	7078	6948	1390	-0,03	-2,35
1199	SACOL1324	5523	4856	971	-0,19	-2,51
1200	**SACOL1325**	11209	29546	5909	1,40	-0,92
1201	**SACOL1326**	3027	4322	864	0,51	-1,81
1202	**SACOL1327**	5477	6347	1269	0,21	-2,11
1203	**SACOL1328**	24472	15282	3056	-0,68	-3,00
1204	**SACOL1329**	13859	7974	1595	-0,80	-3,12
1205	SACOL1331	953	611	122	-0,64	-2,96
1206	SACOL1332	589	502	100	-0,23	-2,55
1207	SACOL1338	187	138	28	-0,44	-2,76
1208	SACOL1340	808	1063	213	0,40	-1,93
1209	SACOL1345	3725	3244	649	-0,20	-2,52
1210	SACOL1346	3152	3047	609	-0,05	-2,37
1211	SACOL1347	2584	1590	318	-0,70	-3,02
1212	**SACOL1349**	4449	7041	1408	0,66	-1,66
1213	**SACOL1351**	3745	19445	3889	2,38	0,05
1214	SACOL1352	195	478	96	1,30	-1,02
1215	SACOL1353	522	1317	263	1,34	-0,99
1216	SACOL1354	4284	7002	1400	0,71	-1,61
1217	**SACOL1355**	2030	3622	724	0,84	-1,49
1218	SACOL1356	223	98	20	-1,19	-3,51
1219	**SACOL1357**	1460	1160	232	-0,33	-2,65
1220	**SACOL1358**	2884	9407	1881	1,71	-0,62
1221	SACOL1359	3839	6860	1372	0,84	-1,48
1222	**SACOL1360**	2138	9263	1853	2,12	-0,21
1223	SACOL1361	12466	6907	1381	-0,85	-3,17
1224	**SACOL1362**	14497	60821	12164	2,07	-0,25

a) *S. aureus* COL Locus. Written in bold indicates that the corresponding protein was identified.
b) *LOWESS normalized background subtracted signal intensities.*
c) *Scaled stationary phase signal intensities to account for decrease in total RNA during stationary phase.*
d) *Log_2 of stationary phase versus exponential growth ratios.*
e) *Log_2 of scaled stationary phase versus exponential growth ratios.*

Supplementary Table 1: Signal intensities and calculated ratios from DNA microarray experiment

	SACOL[a]	signal intensity[b] exponential growth	signal intensity[b] stationary phase	signal intensity corrected[c] stationary phase	induction[d] stat/exp	induction corrected[e] stat/exp
1225	**SACOL1363**	9703	30535	6107	1,65	-0,67
1226	**SACOL1364**	10929	23193	4639	1,09	-1,24
1227	**SACOL1365**	12185	14061	2812	0,21	-2,12
1228	**SACOL1366**	4527	9668	1934	1,09	-1,23
1229	**SACOL1367**	13562	5591	1118	-1,28	-3,60
1230	**SACOL1368**	16816	135186	27037	3,01	0,69
1231	SACOL1369	3392	8741	1748	1,37	-0,96
1232	SACOL1370	2634	3539	708	0,43	-1,90
1233	**SACOL1371**	4715	2947	589	-0,68	-3,00
1234	**SACOL1373**	5493	6258	1252	0,19	-2,13
1235	**SACOL1374**	33814	11094	2219	-1,61	-3,93
1236	SACOL1375	12957	2510	502	-2,37	-4,69
1237	**SACOL1376**	24643	16081	3216	-0,62	-2,94
1238	**SACOL1377**	38856	34645	6929	-0,17	-2,49
1239	**SACOL1378**	28553	5838	1168	-2,29	-4,61
1240	**SACOL1380**	11053	3263	653	-1,76	-4,08
1241	**SACOL1381**	8046	2784	557	-1,53	-3,85
1242	**SACOL1382**	6003	2647	529	-1,18	-3,50
1243	**SACOL1383**	8387	4455	891	-0,91	-3,23
1244	**SACOL1384**	17270	4946	989	-1,80	-4,13
1245	**SACOL1385**	13312	8198	1640	-0,70	-3,02
1246	**SACOL1386**	22261	13986	2797	-0,67	-2,99
1247	**SACOL1387**	1410	2559	512	0,86	-1,46
1248	**SACOL1388**	13945	9305	1861	-0,58	-2,91
1249	**SACOL1389**	11820	3944	789	-1,58	-3,91
1250	**SACOL1390**	14671	4272	854	-1,78	-4,10
1251	**SACOL1392**	4714	3367	673	-0,49	-2,81
1252	**SACOL1393**	5576	12162	2432	1,13	-1,20
1253	SACOL1394	4361	8197	1639	0,91	-1,41
1254	**SACOL1395**	22154	26099	5220	0,24	-2,09
1255	**SACOL1396**	16851	18909	3782	0,17	-2,16
1256	**SACOL1397**	4700	20687	4137	2,14	-0,18
1257	**SACOL1398**	2863	3027	605	0,08	-2,24
1258	**SACOL1399**	13707	34666	6933	1,34	-0,98
1259	SACOL1400	1051	1959	392	0,90	-1,42
1260	**SACOL1401**	4822	3458	692	-0,48	-2,80
1261	**SACOL1402**	3142	9433	1887	1,59	-0,74
1262	**SACOL1403**	19234	2728	546	-2,82	-5,14
1263	**SACOL1404**	44769	5433	1087	-3,04	-5,36
1264	**SACOL1405**	47834	5234	1047	-3,19	-5,51
1265	**SACOL1406**	49990	4857	971	-3,36	-5,69
1266	**SACOL1407**	64190	6333	1267	-3,34	-5,66
1267	**SACOL1408**	59593	6439	1288	-3,21	-5,53
1268	**SACOL1409**	59523	7070	1414	-3,07	-5,40
1269	**SACOL1410**	15747	37984	7597	1,27	-1,05
1270	**SACOL1411**	20255	46968	9394	1,21	-1,11
1271	SACOL1412	2128	2841	568	0,42	-1,91
1272	**SACOL1413**	3590	7760	1552	1,11	-1,21
1273	SACOL1414	9032	11078	2216	0,29	-2,03
1274	SACOL1415	3847	4160	832	0,11	-2,21
1275	SACOL1416	4156	2667	533	-0,64	-2,96
1276	**SACOL1417**	1965	1925	385	-0,03	-2,35
1277	SACOL1418	978	2833	567	1,53	-0,79
1278	**SACOL1419**	8322	6457	1291	-0,37	-2,69
1279	**SACOL1420**	436	1573	315	1,85	-0,47
1280	SACOL1421	392	2952	590	2,91	0,59
1281	SACOL1422	438	3439	688	2,97	0,65
1282	SACOL1423	165	1422	284	3,11	0,79
1283	**SACOL1424**	248	3185	637	3,68	1,36
1284	**SACOL1426**	12725	9020	1804	-0,50	-2,82
1285	**SACOL1427**	17440	3976	795	-2,13	-4,45
1286	**SACOL1428**	15275	3025	605	-2,34	-4,66
1287	**SACOL1429**	21453	24525	4905	0,19	-2,13
1288	**SACOL1430**	26912	33549	6710	0,32	-2,00
1289	**SACOL1431**	23339	27000	5400	0,21	-2,11
1290	**SACOL1432**	19262	18426	3685	-0,06	-2,39
1291	**SACOL1433**	20187	10493	2099	-0,94	-3,27
1292	**SACOL1434**	15990	9317	1863	-0,78	-3,10
1293	**SACOL1435**	8376	10089	2018	0,27	-2,05
1294	SACOL1436	1531	1191	238	-0,36	-2,68
1295	SACOL1438	4892	3479	696	-0,49	-2,81
1296	**SACOL1439**	17542	52743	10549	1,59	-0,73

a) *S. aureus* COL Locus. Written in bold indicates that the corresponding protein was identified.
b) *LOWESS normalized background subtracted signal intensities.*
c) *Scaled stationary phase signal intensities to account for decrease in total RNA during stationary phase.*
d) *Log_2 of stationary phase versus exponential growth ratios.*
e) *Log_2 of scaled stationary phase versus exponential growth ratios.*

Supplementary Table 1: Signal intensities and calculated ratios from DNA microarray experiment

	SACOL[a]	signal intensity[b] exponential growth	signal intensity[b] stationary phase	signal intensity corrected[c] stationary phase	induction[d] stat/exp	induction corrected[e] stat/exp
1297	**SACOL1440**	11134	35902	7180	1,69	-0,63
1298	**SACOL1441**	10000	36322	7264	1,86	-0,46
1299	SACOL1442	172	186	37	0,11	-2,21
1300	**SACOL1443**	11555	1623	325	-2,83	-5,15
1301	**SACOL1444**	15205	7259	1452	-1,07	-3,39
1302	**SACOL1445**	24789	12590	2518	-0,98	-3,30
1303	SACOL1446	19672	14057	2811	-0,48	-2,81
1304	**SACOL1447**	53583	59765	11953	0,16	-2,16
1305	**SACOL1448**	21576	89199	17840	2,05	-0,27
1306	**SACOL1449**	28615	96175	19235	1,75	-0,57
1307	**SACOL1450**	5499	21702	4340	1,98	-0,34
1308	**SACOL1451**	6271	30735	6147	2,29	-0,03
1309	SACOL1452	26635	24712	4942	-0,11	-2,43
1310	**SACOL1453**	18240	13927	2785	-0,39	-2,71
1311	**SACOL1455**	4160	4538	908	0,13	-2,20
1312	SACOL1456	10182	14092	2818	0,47	-1,85
1313	**SACOL1457**	9393	12072	2414	0,36	-1,96
1314	**SACOL1460**	17055	26492	5298	0,64	-1,69
1315	**SACOL1461**	30269	47148	9430	0,64	-1,68
1316	**SACOL1462**	17920	25903	5181	0,53	-1,79
1317	**SACOL1464**	12023	15099	3020	0,33	-1,99
1318	**SACOL1465**	11743	16451	3290	0,49	-1,84
1319	**SACOL1466**	12124	21937	4387	0,86	-1,47
1320	**SACOL1468**	2191	703	141	-1,64	-3,96
1321	**SACOL1471**	2514	2205	441	-0,19	-2,51
1322	**SACOL1472**	910	1917	383	1,07	-1,25
1323	SACOL1475	499	711	142	0,51	-1,81
1324	SACOL1476	600	792	158	0,40	-1,92
1325	SACOL1477	709	1381	276	0,96	-1,36
1326	SACOL1478	425	757	151	0,83	-1,49
1327	**SACOL1479**	20671	11625	2325	-0,83	-3,15
1328	**SACOL1480**	11023	8697	1739	-0,34	-2,66
1329	**SACOL1481**	1859	78	16	-4,57	-6,89
1330	**SACOL1483**	12067	3926	785	-1,62	-3,94
1331	**SACOL1484**	27616	53415	10683	0,95	-1,37
1332	SACOL1485	27378	54851	10970	1,00	-1,32
1333	SACOL1486	22899	34042	6808	0,57	-1,75
1334	SACOL1488	37845	32805	6561	-0,21	-2,53
1335	SACOL1489	30298	22813	4563	-0,41	-2,73
1336	**SACOL1490**	34881	29730	5946	-0,23	-2,55
1337	**SACOL1491**	6713	7751	1550	0,21	-2,11
1338	**SACOL1492**	6508	8037	1607	0,30	-2,02
1339	**SACOL1493**	21004	24266	4853	0,21	-2,11
1340	**SACOL1494**	15057	7662	1532	-0,97	-3,30
1341	**SACOL1495**	14490	6446	1289	-1,17	-3,49
1342	**SACOL1496**	20839	11781	2356	-0,82	-3,14
1343	**SACOL1497**	12950	9666	1933	-0,42	-2,74
1344	**SACOL1498**	12945	11173	2235	-0,21	-2,53
1345	**SACOL1499**	8426	6639	1328	-0,34	-2,67
1346	**SACOL1500**	25609	143253	28651	2,48	0,16
1347	SACOL1501	23456	9404	1881	-1,32	-3,64
1348	SACOL1502	22139	8131	1626	-1,45	-3,77
1349	**SACOL1503**	27525	7892	1578	-1,80	-4,12
1350	**SACOL1504**	18765	6632	1326	-1,50	-3,82
1351	**SACOL1505**	16595	5526	1105	-1,59	-3,91
1352	**SACOL1506**	12771	5166	1033	-1,31	-3,63
1353	SACOL1507	1332	418	84	-1,67	-3,99
1354	**SACOL1509**	9832	4782	956	-1,04	-3,36
1355	**SACOL1510**	23130	19057	3811	-0,28	-2,60
1356	**SACOL1511**	31653	21939	4388	-0,53	-2,85
1357	**SACOL1513**	122659	60095	12019	-1,03	-3,35
1358	**SACOL1514**	32859	21327	4265	-0,62	-2,95
1359	**SACOL1515**	41789	21535	4307	-0,96	-3,28
1360	**SACOL1516**	6220	14200	2840	1,19	-1,13
1361	**SACOL1518**	4049	4029	806	-0,01	-2,33
1362	**SACOL1519**	856	636	127	-0,43	-2,75
1363	**SACOL1520**	8158	4486	897	-0,86	-3,18
1364	**SACOL1522**	25150	72112	14422	1,52	-0,80
1365	SACOL1523	13967	12368	2474	-0,18	-2,50
1366	SACOL1524	14548	8125	1625	-0,84	-3,16
1367	SACOL1525	34257	35187	7037	0,04	-2,28
1368	SACOL1526	17576	23216	4643	0,40	-1,92

a) *S. aureus* COL Locus. Written in bold indicates that the corresponding protein was identified.
b) *LOWESS normalized background subtracted signal intensities.*
c) *Scaled stationary phase signal intensities to account for decrease in total RNA during stationary phase.*
d) *Log_2 of stationary phase versus exponential growth ratios.*
e) *Log_2 of scaled stationary phase versus exponential growth ratios.*

Supplementary Table 1: Signal intensities and calculated ratios from DNA microarray experiment

	SACOL[a]	signal intensity[b] exponential growth	signal intensity[b] stationary phase	signal intensity corrected[c] stationary phase	induction[d] stat/exp	induction corrected[e] stat/exp
1369	**SACOL1528**	3450	1948	390	-0,82	-3,15
1370	**SACOL1530**	9534	3052	610	-1,64	-3,97
1371	**SACOL1532**	10188	10939	2188	0,10	-2,22
1372	**SACOL1534**	13360	14805	2961	0,15	-2,17
1373	**SACOL1535**	38351	35299	7060	-0,12	-2,44
1374	**SACOL1536**	20171	19583	3917	-0,04	-2,36
1375	**SACOL1537**	14598	16235	3247	0,15	-2,17
1376	SACOL1539	1695	4278	856	1,34	-0,99
1377	**SACOL1540**	5920	6659	1332	0,17	-2,15
1378	**SACOL1541**	13319	15791	3158	0,25	-2,08
1379	**SACOL1542**	4063	8465	1693	1,06	-1,26
1380	**SACOL1543**	4810	8630	1726	0,84	-1,48
1381	SACOL1544	1892	3449	690	0,87	-1,46
1382	SACOL1545	1769	3447	689	0,96	-1,36
1383	**SACOL1546**	2930	24837	4967	3,08	0,76
1384	**SACOL1548**	4068	2478	496	-0,72	-3,04
1385	**SACOL1549**	19905	30016	6003	0,59	-1,73
1386	**SACOL1550**	8445	13399	2680	0,67	-1,66
1387	**SACOL1551**	1882	2454	491	0,38	-1,94
1388	**SACOL1552**	7159	4351	870	-0,72	-3,04
1389	**SACOL1553**	4828	5729	1146	0,25	-2,08
1390	**SACOL1554**	33933	33264	6653	-0,03	-2,35
1391	**SACOL1555**	5751	13421	2684	1,22	-1,10
1392	SACOL1556	3927	8287	1657	1,08	-1,24
1393	SACOL1557	5428	8913	1783	0,72	-1,61
1394	**SACOL1558**	7095	10413	2083	0,55	-1,77
1395	**SACOL1560**	28044	8993	1799	-1,64	-3,96
1396	**SACOL1561**	37356	11695	2339	-1,68	-4,00
1397	**SACOL1562**	40632	12580	2516	-1,69	-4,01
1398	**SACOL1563**	45552	17276	3455	-1,40	-3,72
1399	**SACOL1564**	21465	12138	2428	-0,82	-3,14
1400	**SACOL1565**	20357	10688	2138	-0,93	-3,25
1401	**SACOL1566**	50541	16165	3233	-1,64	-3,97
1402	SACOL1567	41432	12339	2468	-1,75	-4,07
1403	**SACOL1568**	23697	8977	1795	-1,40	-3,72
1404	**SACOL1569**	28158	14869	2974	-0,92	-3,24
1405	**SACOL1570**	34642	19170	3834	-0,85	-3,18
1406	**SACOL1571**	43470	25900	5180	-0,75	-3,07
1407	**SACOL1572**	44482	26147	5229	-0,77	-3,09
1408	SACOL1576	806	8647	1729	3,42	1,10
1409	SACOL1577	1632	8258	1652	2,34	0,02
1410	SACOL1578	259	369	74	0,51	-1,81
1411	SACOL1579	354	703	141	0,99	-1,33
1412	SACOL1581	2648	2640	528	0,00	-2,33
1413	**SACOL1582**	597	322	64	-0,89	-3,21
1414	SACOL1583	765	434	87	-0,82	-3,14
1415	SACOL1584	1168	809	162	-0,53	-2,85
1416	**SACOL1587**	4415	5280	1056	0,26	-2,06
1417	**SACOL1588**	15954	12382	2476	-0,37	-2,69
1418	**SACOL1589**	2687	6732	1346	1,33	-1,00
1419	**SACOL1591**	9297	13437	2687	0,53	-1,79
1420	**SACOL1592**	15792	10525	2105	-0,59	-2,91
1421	**SACOL1593**	16823	17737	3547	0,08	-2,25
1422	**SACOL1594**	20011	17155	3431	-0,22	-2,54
1423	**SACOL1595**	23055	20492	4098	-0,17	-2,49
1424	SACOL1596	5038	6935	1387	0,46	-1,86
1425	SACOL1597	460	799	160	0,80	-1,53
1426	SACOL1598	476	493	99	0,05	-2,27
1427	SACOL1599	489	577	115	0,24	-2,08
1428	SACOL1600	465	131	26	-1,83	-4,15
1429	SACOL1601	2213	402	80	-2,46	-4,78
1430	**SACOL1602**	4420	4985	997	0,17	-2,15
1431	**SACOL1603**	5806	5609	1122	-0,05	-2,37
1432	**SACOL1604**	39533	23344	4669	-0,76	-3,08
1433	SACOL1605	40287	21202	4240	-0,93	-3,25
1434	**SACOL1606**	40957	21643	4329	-0,92	-3,24
1435	**SACOL1607**	24162	11904	2381	-1,02	-3,34
1436	SACOL1608	7750	2484	497	-1,64	-3,96
1437	**SACOL1609**	14969	19883	3977	0,41	-1,91
1438	**SACOL1610**	24086	71692	14338	1,57	-0,75
1439	**SACOL1611**	3516	1248	250	-1,49	-3,82
1440	**SACOL1612**	10636	2737	547	-1,96	-4,28

a) *S. aureus* COL Locus. Written in bold indicates that the corresponding protein was identified.
b) *LOWESS normalized background subtracted signal intensities.*
c) *Scaled stationary phase signal intensities to account for decrease in total RNA during stationary phase.*
d) *Log_2 of stationary phase versus exponential growth ratios.*
e) *Log_2 of scaled stationary phase versus exponential growth ratios.*

Supplementary Table 1: Signal intensities and calculated ratios from DNA microarray experiment

	SACOL[a]	signal intensity[b] exponential growth	signal intensity[b] stationary phase	signal intensity corrected[c] stationary phase	induction[d] stat/exp	induction corrected[e] stat/exp
1441	**SACOL1613**	11860	2496	499	-2,25	-4,57
1442	**SACOL1614**	26315	15080	3016	-0,80	-3,13
1443	**SACOL1615**	30064	15612	3122	-0,95	-3,27
1444	**SACOL1616**	15380	14240	2848	-0,11	-2,43
1445	**SACOL1617**	13261	14600	2920	0,14	-2,18
1446	**SACOL1618**	9445	9084	1817	-0,06	-2,38
1447	**SACOL1619**	11665	9489	1898	-0,30	-2,62
1448	**SACOL1620**	11851	22966	4593	0,95	-1,37
1449	**SACOL1621**	8567	16598	3320	0,95	-1,37
1450	**SACOL1622**	3870	1923	385	-1,01	-3,33
1451	SACOL1623	9021	13145	2629	0,54	-1,78
1452	**SACOL1624**	24623	34869	6974	0,50	-1,82
1453	SACOL1625	31143	49165	9833	0,66	-1,66
1454	SACOL1626	33255	53606	10721	0,69	-1,63
1455	**SACOL1627**	30245	42349	8470	0,49	-1,84
1456	**SACOL1628**	29785	40880	8176	0,46	-1,87
1457	**SACOL1629**	657	931	186	0,50	-1,82
1458	**SACOL1630**	48662	47730	9546	-0,03	-2,35
1459	**SACOL1631**	64140	41807	8361	-0,62	-2,94
1460	**SACOL1633**	20596	9804	1961	-1,07	-3,39
1461	**SACOL1634**	25705	12325	2465	-1,06	-3,38
1462	**SACOL1635**	10328	3901	780	-1,40	-3,73
1463	**SACOL1636**	38159	14759	2952	-1,37	-3,69
1464	**SACOL1637**	26375	19270	3854	-0,45	-2,77
1465	**SACOL1638**	43723	33986	6797	-0,36	-2,69
1466	SACOL1639	33972	25376	5075	-0,42	-2,74
1467	SACOL1640	6981	1314	263	-2,41	-4,73
1468	**SACOL1641**	16432	5440	1088	-1,59	-3,92
1469	**SACOL1642**	22655	3953	791	-2,52	-4,84
1470	**SACOL1643**	1329	752	150	-0,82	-3,14
1471	SACOL1644	1552	807	161	-0,94	-3,27
1472	**SACOL1645**	4379	5719	1144	0,38	-1,94
1473	SACOL1646	3715	2851	570	-0,38	-2,70
1474	**SACOL1647**	3601	2928	586	-0,30	-2,62
1475	**SACOL1648**	10824	8030	1606	-0,43	-2,75
1476	**SACOL1649**	17434	12905	2581	-0,43	-2,76
1477	**SACOL1650**	43966	18790	3758	-1,23	-3,55
1478	**SACOL1651**	23336	11034	2207	-1,08	-3,40
1479	**SACOL1652**	27214	14365	2873	-0,92	-3,24
1480	**SACOL1653**	16350	9877	1975	-0,73	-3,05
1481	**SACOL1654**	22898	18157	3631	-0,33	-2,66
1482	**SACOL1655**	21138	13348	2670	-0,66	-2,99
1483	SACOL1656	494	1301	260	1,40	-0,92
1484	SACOL1657	350	632	126	0,85	-1,47
1485	**SACOL1658**	1275	2220	444	0,80	-1,52
1486	**SACOL1659**	7245	2179	436	-1,73	-4,05
1487	**SACOL1660**	42753	7293	1459	-2,55	-4,87
1488	**SACOL1661**	38003	5583	1117	-2,77	-5,09
1489	**SACOL1662**	42302	5184	1037	-3,03	-5,35
1490	**SACOL1663**	26822	3203	641	-3,07	-5,39
1491	**SACOL1664**	28190	3232	646	-3,12	-5,45
1492	**SACOL1665**	5275	2219	444	-1,25	-3,57
1493	**SACOL1666**	11806	7035	1407	-0,75	-3,07
1494	**SACOL1667**	32364	14073	2815	-1,20	-3,52
1495	**SACOL1668**	21331	10518	2104	-1,02	-3,34
1496	**SACOL1669**	29912	10632	2126	-1,49	-3,81
1497	**SACOL1670**	3449	4076	815	0,24	-2,08
1498	**SACOL1671**	17340	14962	2992	-0,21	-2,53
1499	**SACOL1672**	22965	20427	4085	-0,17	-2,49
1500	**SACOL1673**	28818	7611	1522	-1,92	-4,24
1501	**SACOL1674**	19191	5159	1032	-1,90	-4,22
1502	**SACOL1675**	23204	5552	1110	-2,06	-4,39
1503	**SACOL1676**	12647	6292	1258	-1,01	-3,33
1504	**SACOL1677**	22474	13394	2679	-0,75	-3,07
1505	**SACOL1678**	3094	7096	1419	1,20	-1,12
1506	**SACOL1681**	44640	41528	8306	-0,10	-2,43
1507	**SACOL1682**	7776	7272	1454	-0,10	-2,42
1508	**SACOL1683**	19614	11359	2272	-0,79	-3,11
1509	**SACOL1685**	20137	3307	661	-2,61	-4,93
1510	**SACOL1686**	33295	6089	1218	-2,45	-4,77
1511	**SACOL1687**	8393	4465	893	-0,91	-3,23
1512	**SACOL1688**	8210	7352	1470	-0,16	-2,48

a) *S. aureus* COL Locus. Written in bold indicates that the corresponding protein was identified.
b) *LOWESS normalized background subtracted signal intensities.*
c) *Scaled stationary phase signal intensities to account for decrease in total RNA during stationary phase.*
d) *Log_2 of stationary phase versus exponential growth ratios.*
e) *Log_2 of scaled stationary phase versus exponential growth ratios.*

Supplementary Table 1: Signal intensities and calculated ratios from DNA microarray experiment

	SACOL[a]	signal intensity[b] exponential growth	signal intensity[b] stationary phase	signal intensity corrected[c] stationary phase	induction[d] stat/exp	induction corrected[e] stat/exp
1513	**SACOL1689**	16560	25272	5054	0,61	-1,71
1514	**SACOL1690**	8097	2267	453	-1,84	-4,16
1515	**SACOL1691**	20798	4518	904	-2,20	-4,52
1516	**SACOL1692**	7201	4140	828	-0,80	-3,12
1517	**SACOL1693**	14924	8878	1776	-0,75	-3,07
1518	**SACOL1694**	21886	7146	1429	-1,61	-3,94
1519	**SACOL1695**	41102	12083	2417	-1,77	-4,09
1520	**SACOL1696**	41991	19237	3847	-1,13	-3,45
1521	**SACOL1697**	42687	22544	4509	-0,92	-3,24
1522	**SACOL1698**	32562	16029	3206	-1,02	-3,34
1523	**SACOL1699**	38700	18709	3742	-1,05	-3,37
1524	**SACOL1700**	30439	4716	943	-2,69	-5,01
1525	SACOL1701	65768	10052	2010	-2,71	-5,03
1526	**SACOL1702**	73656	9796	1959	-2,91	-5,23
1527	SACOL1703	11037	8434	1687	-0,39	-2,71
1528	**SACOL1704**	11950	10181	2036	-0,23	-2,55
1529	**SACOL1705**	15356	22288	4458	0,54	-1,78
1530	SACOL1706	1226	1696	339	0,47	-1,85
1531	SACOL1707	298	613	123	1,04	-1,28
1532	SACOL1708	502	1608	322	1,68	-0,64
1533	**SACOL1709**	21627	12920	2584	-0,74	-3,07
1534	**SACOL1710**	13739	9666	1933	-0,51	-2,83
1535	SACOL1711	1561	7713	1543	2,30	-0,02
1536	SACOL1712	10587	5966	1193	-0,83	-3,15
1537	**SACOL1714**	62649	28071	5614	-1,16	-3,48
1538	**SACOL1715**	40628	16255	3251	-1,32	-3,64
1539	**SACOL1716**	37333	9576	1915	-1,96	-4,28
1540	**SACOL1717**	36082	15188	3038	-1,25	-3,57
1541	SACOL1718	33903	14629	2926	-1,21	-3,53
1542	SACOL1719	39963	24788	4958	-0,69	-3,01
1543	**SACOL1720**	20905	7619	1524	-1,46	-3,78
1544	**SACOL1721**	12877	9004	1801	-0,52	-2,84
1545	**SACOL1722**	3584	2113	423	-0,76	-3,08
1546	**SACOL1723**	13346	10331	2066	-0,37	-2,69
1547	**SACOL1724**	25550	16801	3360	-0,60	-2,93
1548	**SACOL1725**	33724	3777	755	-3,16	-5,48
1549	**SACOL1726**	74037	10578	2116	-2,81	-5,13
1550	**SACOL1727**	141055	21538	4308	-2,71	-5,03
1551	**SACOL1728**	3867	13606	2721	1,81	-0,51
1552	**SACOL1729**	14688	6755	1351	-1,12	-3,44
1553	**SACOL1731**	17664	18370	3674	0,06	-2,27
1554	**SACOL1732**	21957	22576	4515	0,04	-2,28
1555	**SACOL1733**	18110	17338	3468	-0,06	-2,38
1556	**SACOL1734**	2215	13220	2644	2,58	0,26
1557	**SACOL1735**	5042	9442	1888	0,91	-1,42
1558	**SACOL1736**	15061	24679	4936	0,71	-1,61
1559	**SACOL1737**	8221	18725	3745	1,19	-1,13
1560	SACOL1738	4924	2285	457	-1,11	-3,43
1561	**SACOL1739**	5223	12011	2402	1,20	-1,12
1562	**SACOL1740**	4702	19804	3961	2,07	-0,25
1563	**SACOL1741**	13079	43542	8708	1,74	-0,59
1564	**SACOL1742**	14528	57349	11470	1,98	-0,34
1565	**SACOL1743**	29463	10253	2051	-1,52	-3,84
1566	**SACOL1745**	3648	4605	921	0,34	-1,99
1567	**SACOL1746**	21273	24431	4886	0,20	-2,12
1568	**SACOL1747**	26129	7179	1436	-1,86	-4,19
1569	**SACOL1748**	17862	6196	1239	-1,53	-3,85
1570	**SACOL1749**	19272	5526	1105	-1,80	-4,12
1571	**SACOL1750**	11211	9311	1862	-0,27	-2,59
1572	**SACOL1751**	17696	15086	3017	-0,23	-2,55
1573	**SACOL1752**	27501	16174	3235	-0,77	-3,09
1574	**SACOL1753**	4298	12266	2453	1,51	-0,81
1575	**SACOL1756**	4193	12488	2498	1,57	-0,75
1576	SACOL1757	1492	2104	421	0,50	-1,83
1577	**SACOL1758**	4340	17649	3530	2,02	-0,30
1578	**SACOL1759**	6981	41022	8204	2,55	0,23
1579	**SACOL1760**	8224	6494	1299	-0,34	-2,66
1580	**SACOL1761**	17426	19801	3960	0,18	-2,14
1581	**SACOL1762**	38037	50011	10002	0,39	-1,93
1582	**SACOL1763**	14936	3587	717	-2,06	-4,38
1583	**SACOL1764**	15196	3278	656	-2,21	-4,53
1584	**SACOL1765**	13871	3069	614	-2,18	-4,50

a) *S. aureus* COL Locus. Written in bold indicates that the corresponding protein was identified.
b) *LOWESS normalized background subtracted signal intensities.*
c) *Scaled stationary phase signal intensities to account for decrease in total RNA during stationary phase.*
d) *Log_2 of stationary phase versus exponential growth ratios.*
e) *Log_2 of scaled stationary phase versus exponential growth ratios.*

Supplementary Table 1: Signal intensities and calculated ratios from DNA microarray experiment

	SACOL[a]	signal intensity[b] exponential growth	signal intensity[b] stationary phase	signal intensity corrected[c] stationary phase	induction[d] stat/exp	induction corrected[e] stat/exp
1585	SACOL1766	72	39	8	-0,89	-3,21
1586	**SACOL1767**	9544	23161	4632	1,28	-1,04
1587	**SACOL1768**	1749	3110	622	0,83	-1,49
1588	**SACOL1769**	4117	529	106	-2,96	-5,28
1589	**SACOL1770**	2392	6387	1277	1,42	-0,90
1590	**SACOL1771**	9257	10304	2061	0,15	-2,17
1591	**SACOL1772**	41630	15590	3118	-1,42	-3,74
1592	**SACOL1773**	20067	6869	1374	-1,55	-3,87
1593	**SACOL1774**	6158	7553	1511	0,29	-2,03
1594	**SACOL1775**	6492	3321	664	-0,97	-3,29
1595	**SACOL1776**	4921	2760	552	-0,83	-3,16
1596	**SACOL1777**	7529	9239	1848	0,30	-2,03
1597	**SACOL1778**	253	217	43	-0,23	-2,55
1598	**SACOL1779**	5567	5330	1066	-0,06	-2,38
1599	SACOL1781	1237	1242	248	0,01	-2,32
1600	**SACOL1782**	12555	30021	6004	1,26	-1,06
1601	**SACOL1783**	828	39357	7871	5,57	3,25
1602	**SACOL1784**	1281	7523	1505	2,55	0,23
1603	**SACOL1785**	1323	9616	1923	2,86	0,54
1604	**SACOL1786**	8057	13958	2792	0,79	-1,53
1605	**SACOL1787**	3654	3358	672	-0,12	-2,44
1606	**SACOL1788**	3870	55058	11012	3,83	1,51
1607	**SACOL1789**	10295	113920	22784	3,47	1,15
1608	**SACOL1790**	36256	33370	6674	-0,12	-2,44
1609	**SACOL1791**	36130	26243	5249	-0,46	-2,78
1610	**SACOL1792**	35741	38157	7631	0,09	-2,23
1611	**SACOL1793**	19136	11594	2319	-0,72	-3,04
1612	**SACOL1794**	8387	20519	4104	1,29	-1,03
1613	**SACOL1795**	12554	30823	6165	1,30	-1,03
1614	**SACOL1796**	2457	3304	661	0,43	-1,89
1615	**SACOL1797**	4004	6965	1393	0,80	-1,52
1616	**SACOL1798**	47361	18514	3703	-1,36	-3,68
1617	**SACOL1799**	44982	17669	3534	-1,35	-3,67
1618	**SACOL1800**	20431	15024	3005	-0,44	-2,77
1619	**SACOL1801**	23519	16248	3250	-0,53	-2,86
1620	**SACOL1802**	8307	44664	8933	2,43	0,10
1621	SACOL1803	13542	13359	2672	-0,02	-2,34
1622	**SACOL1804**	10753	10369	2074	-0,05	-2,37
1623	**SACOL1805**	6507	5300	1060	-0,30	-2,62
1624	SACOL1806	764	1459	292	0,93	-1,39
1625	**SACOL1807**	29982	8569	1714	-1,81	-4,13
1626	**SACOL1808**	16419	5289	1058	-1,63	-3,96
1627	**SACOL1809**	10270	2251	450	-2,19	-4,51
1628	**SACOL1810**	6735	3741	748	-0,85	-3,17
1629	**SACOL1811**	4652	2683	537	-0,79	-3,12
1630	**SACOL1812**	12746	51076	10215	2,00	-0,32
1631	**SACOL1814**	5372	7061	1412	0,39	-1,93
1632	SACOL1815	4784	2754	551	-0,80	-3,12
1633	**SACOL1816**	7969	10290	2058	0,37	-1,95
1634	**SACOL1817**	16118	4612	922	-1,81	-4,13
1635	**SACOL1818**	64350	11404	2281	-2,50	-4,82
1636	**SACOL1819**	51848	12892	2578	-2,01	-4,33
1637	**SACOL1820**	46948	14918	2984	-1,65	-3,98
1638	**SACOL1821**	1757	6365	1273	1,86	-0,46
1639	SACOL1822	3764	2408	482	-0,64	-2,97
1640	SACOL1823	3685	2664	533	-0,47	-2,79
1641	**SACOL1824**	1293	1264	253	-0,03	-2,35
1642	**SACOL1825**	26801	25246	5049	-0,09	-2,41
1643	SACOL1826	8027	11900	2380	0,57	-1,75
1644	SACOL1827	472	378	76	-0,32	-2,64
1645	SACOL1828	859	483	97	-0,83	-3,15
1646	**SACOL1829**	4006	4389	878	0,13	-2,19
1647	SACOL1830	2026	1151	230	-0,82	-3,14
1648	**SACOL1831**	23386	41526	8305	0,83	-1,49
1649	SACOL1832	3721	1905	381	-0,97	-3,29
1650	SACOL1833	1086	587	117	-0,89	-3,21
1651	**SACOL1835**	5044	10675	2135	1,08	-1,24
1652	**SACOL1836**	4194	5056	1011	0,27	-2,05
1653	**SACOL1837**	7411	2906	581	-1,35	-3,67
1654	**SACOL1838**	11337	57604	11521	2,35	0,02
1655	**SACOL1839**	1828	2145	429	0,23	-2,09
1656	**SACOL1840**	8010	2076	415	-1,95	-4,27

a) *S. aureus* COL Locus. Written in bold indicates that the corresponding protein was identified.
b) *LOWESS normalized background subtracted signal intensities.*
c) *Scaled stationary phase signal intensities to account for decrease in total RNA during stationary phase.*
d) *Log_2 of stationary phase versus exponential growth ratios.*
e) *Log_2 of scaled stationary phase versus exponential growth ratios.*

Supplementary Table 1: Signal intensities and calculated ratios from DNA microarray experiment

	SACOL[a]	signal intensity[b] exponential growth	signal intensity[b] stationary phase	signal intensity corrected[c] stationary phase	induction[d] stat/exp	induction corrected[e] stat/exp
1657	**SACOL1841**	12978	2955	591	-2,13	-4,46
1658	**SACOL1842**	2813	3686	737	0,39	-1,93
1659	**SACOL1843**	14380	4716	943	-1,61	-3,93
1660	SACOL1844	14580	4182	836	-1,80	-4,12
1661	SACOL1845	1655	920	184	-0,85	-3,17
1662	SACOL1846	8077	98129	19626	3,60	1,28
1663	**SACOL1847**	1876	17933	3587	3,26	0,93
1664	**SACOL1848**	3580	2860	572	-0,32	-2,65
1665	SACOL1849	1867	1345	269	-0,47	-2,79
1666	SACOL1850	1349	1013	203	-0,41	-2,74
1667	SACOL1851	738	1433	287	0,96	-1,37
1668	SACOL1854	7079	13867	2773	0,97	-1,35
1669	SACOL1858	6900	6649	1330	-0,05	-2,38
1670	SACOL1859	4129	3720	744	-0,15	-2,47
1671	SACOL1860	5175	1472	294	-1,81	-4,14
1672	**SACOL1861**	12689	7248	1450	-0,81	-3,13
1673	SACOL1863	13	6	1	-1,04	-3,36
1674	SACOL1865	128	190	38	0,58	-1,75
1675	SACOL1866	428	829	166	0,95	-1,37
1676	**SACOL1867**	308	732	146	1,25	-1,07
1677	**SACOL1868**	271	267	53	-0,02	-2,34
1678	SACOL1869	382	232	46	-0,72	-3,04
1679	**SACOL1870**	420	514	103	0,29	-2,03
1680	SACOL1871	3129	91848	18370	4,88	2,55
1681	SACOL1872	2806	102446	20489	5,19	2,87
1682	**SACOL1874**	580	916	183	0,66	-1,66
1683	SACOL1875	1582	2150	430	0,44	-1,88
1684	SACOL1877	406	633	127	0,64	-1,68
1685	SACOL1878	408	612	122	0,58	-1,74
1686	**SACOL1880**	385	299	60	-0,37	-2,69
1687	SACOL1881	301	179	36	-0,74	-3,07
1688	SACOL1882	1066	5347	1069	2,33	0,00
1689	**SACOL1883**	7402	13357	2671	0,85	-1,47
1690	**SACOL1885**	13550	7244	1449	-0,90	-3,23
1691	SACOL1886	71	55	11	-0,37	-2,69
1692	**SACOL1887**	19272	9279	1856	-1,05	-3,38
1693	**SACOL1888**	17119	12038	2408	-0,51	-2,83
1694	**SACOL1889**	21162	13862	2772	-0,61	-2,93
1695	SACOL1890	12923	7698	1540	-0,75	-3,07
1696	**SACOL1891**	17494	31395	6279	0,84	-1,48
1697	**SACOL1892**	13546	13669	2734	0,01	-2,31
1698	**SACOL1893**	26332	25344	5069	-0,06	-2,38
1699	**SACOL1894**	9014	20712	4142	1,20	-1,12
1700	**SACOL1895**	7690	60572	12114	2,98	0,66
1701	SACOL1896	1142	1998	400	0,81	-1,51
1702	**SACOL1897**	5217	4475	895	-0,22	-2,54
1703	**SACOL1898**	1626	2848	570	0,81	-1,51
1704	**SACOL1899**	31467	32651	6530	0,05	-2,27
1705	**SACOL1900**	29926	35131	7026	0,23	-2,09
1706	**SACOL1902**	819	1292	258	0,66	-1,66
1707	**SACOL1903**	2398	2949	590	0,30	-2,02
1708	**SACOL1904**	22771	7116	1423	-1,68	-4,00
1709	**SACOL1905**	6156	5614	1123	-0,13	-2,45
1710	**SACOL1906**	4372	5389	1078	0,30	-2,02
1711	**SACOL1907**	6572	4306	861	-0,61	-2,93
1712	**SACOL1908**	1392	9459	1892	2,76	0,44
1713	**SACOL1909**	426	1129	226	1,41	-0,92
1714	**SACOL1912**	415	4996	999	3,59	1,27
1715	**SACOL1913**	4871	4651	930	-0,07	-2,39
1716	SACOL1914	7005	5222	1044	-0,42	-2,75
1717	SACOL1915	392	1595	319	2,02	-0,30
1718	**SACOL1916**	543	524	105	-0,05	-2,37
1719	**SACOL1917**	2610	3954	791	0,60	-1,72
1720	**SACOL1919**	6995	7394	1479	0,08	-2,24
1721	**SACOL1920**	8079	19367	3873	1,26	-1,06
1722	**SACOL1921**	8071	19829	3966	1,30	-1,03
1723	**SACOL1922**	13993	10532	2106	-0,41	-2,73
1724	**SACOL1923**	6327	8761	1752	0,47	-1,85
1725	**SACOL1924**	14003	6441	1288	-1,12	-3,44
1726	**SACOL1925**	48375	14445	2889	-1,74	-4,07
1727	SACOL1926	25291	7192	1438	-1,81	-4,14
1728	**SACOL1927**	11241	18385	3677	0,71	-1,61

a) *S. aureus* COL Locus. Written in bold indicates that the corresponding protein was identified.
b) *LOWESS normalized background subtracted signal intensities.*
c) *Scaled stationary phase signal intensities to account for decrease in total RNA during stationary phase.*
d) *Log_2 of stationary phase versus exponential growth ratios.*
e) *Log_2 of scaled stationary phase versus exponential growth ratios.*

Supplementary Table 1: Signal intensities and calculated ratios from DNA microarray experiment

	SACOL[a]	signal intensity[b] exponential growth	signal intensity[b] stationary phase	signal intensity corrected[c] stationary phase	induction[d] stat/exp	induction corrected[e] stat/exp
1729	**SACOL1928**	31917	11239	2248	-1,51	-3,83
1730	**SACOL1929**	19463	12731	2546	-0,61	-2,93
1731	**SACOL1930**	18908	12535	2507	-0,59	-2,91
1732	**SACOL1931**	6995	6054	1211	-0,21	-2,53
1733	**SACOL1932**	2311	5652	1130	1,29	-1,03
1734	**SACOL1933**	5877	58271	11654	3,31	0,99
1735	SACOL1934	2721	2946	589	0,11	-2,21
1736	SACOL1935	4633	3370	674	-0,46	-2,78
1737	**SACOL1936**	12576	14050	2810	0,16	-2,16
1738	**SACOL1937**	20780	21493	4299	0,05	-2,27
1739	SACOL1938	18447	16077	3215	-0,20	-2,52
1740	**SACOL1939**	8404	7845	1569	-0,10	-2,42
1741	**SACOL1940**	6133	3684	737	-0,74	-3,06
1742	**SACOL1941**	7544	15887	3177	1,07	-1,25
1743	**SACOL1942**	11347	7686	1537	-0,56	-2,88
1744	**SACOL1943**	8804	7790	1558	-0,18	-2,50
1745	**SACOL1944**	7058	7028	1406	-0,01	-2,33
1746	SACOL1945	11644	13504	2701	0,21	-2,11
1747	**SACOL1946**	16822	12573	2515	-0,42	-2,74
1748	**SACOL1947**	6237	4446	889	-0,49	-2,81
1749	SACOL1948	783	372	74	-1,08	-3,40
1750	**SACOL1950**	6994	3848	770	-0,86	-3,18
1751	**SACOL1951**	17877	8829	1766	-1,02	-3,34
1752	**SACOL1952**	2492	14989	2998	2,59	0,27
1753	SACOL1953	477	200	40	-1,25	-3,57
1754	SACOL1954	7328	5220	1044	-0,49	-2,81
1755	SACOL1955	6782	5840	1168	-0,22	-2,54
1756	SACOL1956	15058	26909	5382	0,84	-1,48
1757	**SACOL1957**	12996	7389	1478	-0,81	-3,14
1758	**SACOL1958**	20404	14924	2985	-0,45	-2,77
1759	**SACOL1960**	28238	14983	2997	-0,91	-3,24
1760	**SACOL1961**	33575	13772	2754	-1,29	-3,61
1761	**SACOL1962**	23659	8738	1748	-1,44	-3,76
1762	**SACOL1963**	2701	7923	1585	1,55	-0,77
1763	**SACOL1964**	59129	26530	5306	-1,16	-3,48
1764	**SACOL1965**	42385	11742	2348	-1,85	-4,17
1765	**SACOL1966**	29990	8946	1789	-1,75	-4,07
1766	**SACOL1967**	29364	10730	2146	-1,45	-3,77
1767	**SACOL1968**	8526	4663	933	-0,87	-3,19
1768	**SACOL1969**	13095	8370	1674	-0,65	-2,97
1769	**SACOL1970**	1152	224	45	-2,37	-4,69
1770	SACOL1971	1066	576	115	-0,89	-3,21
1771	SACOL1972	1650	2500	500	0,60	-1,72
1772	**SACOL1973**	3456	4880	976	0,50	-1,82
1773	**SACOL1974**	13806	21077	4215	0,61	-1,71
1774	**SACOL1975**	7277	10931	2186	0,59	-1,73
1775	**SACOL1976**	3787	2011	402	-0,91	-3,24
1776	SACOL1977	1758	1687	337	-0,06	-2,38
1777	SACOL1978	7418	28135	5627	1,92	-0,40
1778	**SACOL1979**	6101	20861	4172	1,77	-0,55
1779	**SACOL1980**	1709	16535	3307	3,27	0,95
1780	SACOL1981	25122	15923	3185	-0,66	-2,98
1781	**SACOL1982**	4751	6012	1202	0,34	-1,98
1782	**SACOL1984**	10443	15818	3164	0,60	-1,72
1783	**SACOL1985**	7599	7536	1507	-0,01	-2,33
1784	SACOL1986	893	898	180	0,01	-2,31
1785	**SACOL1987**	9786	40278	8056	2,04	-0,28
1786	**SACOL1988**	1903	5879	1176	1,63	-0,69
1787	**SACOL1989**	10667	10995	2199	0,04	-2,28
1788	**SACOL1990**	10271	7569	1514	-0,44	-2,76
1789	**SACOL1991**	20339	11050	2210	-0,88	-3,20
1790	**SACOL1992**	6752	42835	8567	2,67	0,34
1791	**SACOL1993**	6506	4297	859	-0,60	-2,92
1792	**SACOL1994**	10430	6818	1364	-0,61	-2,94
1793	**SACOL1995**	12763	7942	1588	-0,68	-3,01
1794	**SACOL1996**	16952	12474	2495	-0,44	-2,76
1795	SACOL1997	15448	12267	2453	-0,33	-2,65
1796	SACOL1998	10096	3174	635	-1,67	-3,99
1797	SACOL1999	668	3465	693	2,38	0,05
1798	**SACOL2000**	18101	17179	3436	-0,08	-2,40
1799	**SACOL2002**	1790	5608	1122	1,65	-0,67
1800	**SACOL2003**	2782	4389	878	0,66	-1,66

a) *S. aureus* COL Locus. Written in bold indicates that the corresponding protein was identified.
b) *LOWESS normalized background subtracted signal intensities.*
c) *Scaled stationary phase signal intensities to account for decrease in total RNA during stationary phase.*
d) *Log_2 of stationary phase versus exponential growth ratios.*
e) *Log_2 of scaled stationary phase versus exponential growth ratios.*

Supplementary Table 1: Signal intensities and calculated ratios from DNA microarray experiment

	SACOL[a]	signal intensity[b] exponential growth	signal intensity[b] stationary phase	signal intensity corrected[c] stationary phase	induction[d] stat/exp	induction corrected[e] stat/exp
1801	**SACOL2004**	195	134	27	-0,54	-2,86
1802	**SACOL2006**	422	263	53	-0,68	-3,00
1803	SACOL2009	1605	986	197	-0,70	-3,02
1804	**SACOL2010**	408	263	53	-0,63	-2,95
1805	SACOL2011	2535	1262	252	-1,01	-3,33
1806	**SACOL2012**	5863	11993	2399	1,03	-1,29
1807	SACOL2013	3662	8419	1684	1,20	-1,12
1808	**SACOL2016**	15391	27519	5504	0,84	-1,48
1809	**SACOL2017**	24391	37183	7437	0,61	-1,71
1810	**SACOL2018**	2341	3452	690	0,56	-1,76
1811	**SACOL2019**	3372	20292	4058	2,59	0,27
1812	**SACOL2020**	4577	15081	3016	1,72	-0,60
1813	**SACOL2021**	9921	23417	4683	1,24	-1,08
1814	SACOL2023	24564	22490	4498	-0,13	-2,45
1815	**SACOL2025**	14202	14702	2940	0,05	-2,27
1816	**SACOL2026**	5465	4817	963	-0,18	-2,50
1817	**SACOL2028**	19215	25765	5153	0,42	-1,90
1818	**SACOL2029**	18703	28078	5616	0,59	-1,74
1819	SACOL2030	10400	8817	1763	-0,24	-2,56
1820	SACOL2031	1862	639	128	-1,54	-3,87
1821	SACOL2033	1860	239	48	-2,96	-5,28
1822	SACOL2034	1883	273	55	-2,78	-5,11
1823	**SACOL2035**	3310	1414	283	-1,23	-3,55
1824	**SACOL2036**	6769	6977	1395	0,04	-2,28
1825	**SACOL2037**	3849	1405	281	-1,45	-3,78
1826	**SACOL2038**	16200	6238	1248	-1,38	-3,70
1827	SACOL2039	12765	4908	982	-1,38	-3,70
1828	SACOL2040	13206	5608	1122	-1,24	-3,56
1829	**SACOL2041**	25003	10001	2000	-1,32	-3,64
1830	**SACOL2042**	155725	4450	890	-5,13	-7,45
1831	**SACOL2043**	158425	3818	764	-5,37	-7,70
1832	**SACOL2044**	210013	4487	897	-5,55	-7,87
1833	**SACOL2045**	250154	7676	1535	-5,03	-7,35
1834	**SACOL2046**	214657	4472	894	-5,58	-7,91
1835	**SACOL2047**	263660	5192	1038	-5,67	-7,99
1836	**SACOL2048**	134383	3880	776	-5,11	-7,44
1837	**SACOL2049**	164688	4155	831	-5,31	-7,63
1838	**SACOL2050**	32764	1013	203	-5,02	-7,34
1839	**SACOL2052**	46243	11609	2322	-1,99	-4,32
1840	**SACOL2053**	45282	13977	2795	-1,70	-4,02
1841	**SACOL2054**	49433	113109	22622	1,19	-1,13
1842	**SACOL2055**	42593	90788	18158	1,09	-1,23
1843	**SACOL2056**	59777	104463	20893	0,81	-1,52
1844	**SACOL2057**	39080	22991	4598	-0,77	-3,09
1845	**SACOL2058**	37621	30061	6012	-0,32	-2,65
1846	SACOL2059	41805	26861	5372	-0,64	-2,96
1847	**SACOL2060**	15596	15515	3103	-0,01	-2,33
1848	**SACOL2061**	11991	19082	3816	0,67	-1,65
1849	**SACOL2062**	13729	21197	4239	0,63	-1,70
1850	**SACOL2063**	12733	17804	3561	0,48	-1,84
1851	**SACOL2064**	12417	13958	2792	0,17	-2,15
1852	SACOL2066	484	946	189	0,97	-1,35
1853	SACOL2067	532	835	167	0,65	-1,67
1854	SACOL2068	7529	7825	1565	0,06	-2,27
1855	SACOL2070	1637	2612	522	0,67	-1,65
1856	SACOL2071	2448	4172	834	0,77	-1,55
1857	**SACOL2072**	55523	5264	1053	-3,40	-5,72
1858	**SACOL2073**	42548	41568	8314	-0,03	-2,36
1859	**SACOL2074**	34280	18932	3786	-0,86	-3,18
1860	**SACOL2075**	5910	4359	872	-0,44	-2,76
1861	SACOL2076	13090	54722	10944	2,06	-0,26
1862	SACOL2077	5236	12042	2408	1,20	-1,12
1863	**SACOL2078**	7410	15255	3051	1,04	-1,28
1864	**SACOL2079**	22853	17321	3464	-0,40	-2,72
1865	**SACOL2080**	7046	4175	835	-0,75	-3,08
1866	SACOL2081	1006	565	113	-0,83	-3,15
1867	**SACOL2082**	25921	7647	1529	-1,76	-4,08
1868	**SACOL2083**	1196	4558	912	1,93	-0,39
1869	**SACOL2084**	1130	4696	939	2,06	-0,27
1870	**SACOL2085**	1566	6997	1399	2,16	-0,16
1871	**SACOL2086**	1365	5794	1159	2,09	-0,24
1872	**SACOL2088**	857	2677	535	1,64	-0,68

a) *S. aureus* COL Locus. Written in bold indicates that the corresponding protein was identified.
b) *LOWESS normalized background subtracted signal intensities.*
c) *Scaled stationary phase signal intensities to account for decrease in total RNA during stationary phase.*
d) *Log_2 of stationary phase versus exponential growth ratios.*
e) *Log_2 of scaled stationary phase versus exponential growth ratios.*

Supplementary Table 1: Signal intensities and calculated ratios from DNA microarray experiment

	SACOL[a]	signal intensity[b] exponential growth	signal intensity[b] stationary phase	signal intensity corrected[c] stationary phase	induction[d] stat/exp	induction corrected[e] stat/exp
1873	SACOL2089	1612	3513	703	1,12	-1,20
1874	**SACOL2090**	948	3541	708	1,90	-0,42
1875	**SACOL2091**	16798	13231	2646	-0,34	-2,67
1876	**SACOL2092**	30560	48154	9631	0,66	-1,67
1877	SACOL2093	22356	43784	8757	0,97	-1,35
1878	**SACOL2094**	33506	23375	4675	-0,52	-2,84
1879	**SACOL2095**	61877	42656	8531	-0,54	-2,86
1880	**SACOL2096**	74078	40881	8176	-0,86	-3,18
1881	**SACOL2097**	99195	54584	10917	-0,86	-3,18
1882	**SACOL2098**	83961	53227	10645	-0,66	-2,98
1883	**SACOL2099**	64682	38331	7666	-0,75	-3,08
1884	**SACOL2100**	78354	49588	9918	-0,66	-2,98
1885	**SACOL2101**	86628	52209	10442	-0,73	-3,05
1886	SACOL2102	99315	55366	11073	-0,84	-3,16
1887	**SACOL2103**	47922	41961	8392	-0,19	-2,51
1888	**SACOL2104**	51721	50417	10083	-0,04	-2,36
1889	**SACOL2105**	66262	75413	15083	0,19	-2,14
1890	**SACOL2106**	73320	78342	15668	0,10	-2,23
1891	**SACOL2107**	8373	1762	352	-2,25	-4,57
1892	**SACOL2108**	16485	3841	768	-2,10	-4,42
1893	**SACOL2109**	72904	16674	3335	-2,13	-4,45
1894	**SACOL2110**	45571	13841	2768	-1,72	-4,04
1895	**SACOL2111**	48369	11619	2324	-2,06	-4,38
1896	**SACOL2112**	4408	1109	222	-1,99	-4,31
1897	**SACOL2113**	30435	10495	2099	-1,54	-3,86
1898	**SACOL2114**	5104	31811	6362	2,64	0,32
1899	**SACOL2115**	46463	50209	10042	0,11	-2,21
1900	**SACOL2116**	22853	19015	3803	-0,27	-2,59
1901	**SACOL2117**	122633	35699	7140	-1,78	-4,10
1902	**SACOL2118**	4351	2830	566	-0,62	-2,94
1903	**SACOL2119**	32101	6518	1304	-2,30	-4,62
1904	**SACOL2121**	7894	5788	1158	-0,45	-2,77
1905	**SACOL2122**	17432	7113	1423	-1,29	-3,62
1906	**SACOL2123**	2114	1716	343	-0,30	-2,62
1907	**SACOL2124**	7961	8063	1613	0,02	-2,30
1908	**SACOL2125**	12009	12942	2588	0,11	-2,21
1909	**SACOL2126**	6568	7991	1598	0,28	-2,04
1910	**SACOL2127**	4350	19299	3860	2,15	-0,17
1911	**SACOL2128**	4178	17701	3540	2,08	-0,24
1912	**SACOL2130**	17682	14975	2995	-0,24	-2,56
1913	**SACOL2131**	3683	21026	4205	2,51	0,19
1914	**SACOL2132**	3913	24057	4811	2,62	0,30
1915	**SACOL2133**	15777	26272	5254	0,74	-1,59
1916	SACOL2134	16084	4745	949	-1,76	-4,08
1917	**SACOL2135**	19629	9291	1858	-1,08	-3,40
1918	**SACOL2136**	15930	105407	21081	2,73	0,40
1919	SACOL2137	3264	8227	1645	1,33	-0,99
1920	SACOL2138	2625	6776	1355	1,37	-0,95
1921	**SACOL2142**	4940	5915	1183	0,26	-2,06
1922	SACOL2143	7521	12516	2503	0,73	-1,59
1923	**SACOL2144**	20508	25240	5048	0,30	-2,02
1924	**SACOL2145**	7677	199510	39902	4,70	2,38
1925	**SACOL2146**	1321	2962	592	1,16	-1,16
1926	**SACOL2147**	4213	11262	2252	1,42	-0,90
1927	**SACOL2148**	5184	50648	10130	3,29	0,97
1928	**SACOL2149**	7196	55574	11115	2,95	0,63
1929	SACOL2150	1457	2420	484	0,73	-1,59
1930	**SACOL2151**	36012	33029	6606	-0,12	-2,45
1931	**SACOL2152**	22549	22798	4560	0,02	-2,31
1932	**SACOL2153**	26956	30593	6119	0,18	-2,14
1933	**SACOL2154**	2142	29360	5872	3,78	1,46
1934	**SACOL2156**	9132	10845	2169	0,25	-2,07
1935	**SACOL2157**	12542	5394	1079	-1,22	-3,54
1936	SACOL2158	36609	6553	1311	-2,48	-4,80
1937	**SACOL2159**	10405	2820	564	-1,88	-4,21
1938	**SACOL2160**	15464	4847	969	-1,67	-4,00
1939	**SACOL2161**	22315	9377	1875	-1,25	-3,57
1940	SACOL2162	32876	8706	1741	-1,92	-4,24
1941	**SACOL2163**	10986	53646	10729	2,29	-0,03
1942	**SACOL2164**	5516	2017	403	-1,45	-3,77
1943	**SACOL2165**	5238	2235	447	-1,23	-3,55
1944	**SACOL2166**	8505	3924	785	-1,12	-3,44

a) *S. aureus* COL Locus. Written in bold indicates that the corresponding protein was identified.
b) *LOWESS normalized background subtracted signal intensities.*
c) *Scaled stationary phase signal intensities to account for decrease in total RNA during stationary phase.*
d) *Log_2 of stationary phase versus exponential growth ratios.*
e) *Log_2 of scaled stationary phase versus exponential growth ratios.*

Supplementary Table 1: Signal intensities and calculated ratios from DNA microarray experiment

	SACOL[a]	signal intensity[b] exponential growth	signal intensity[b] stationary phase	signal intensity corrected[c] stationary phase	induction[d] stat/exp	induction corrected[e] stat/exp
1945	**SACOL2167**	5289	2413	483	-1,13	-3,45
1946	SACOL2168	4136	12404	2481	1,58	-0,74
1947	**SACOL2169**	4553	18326	3665	2,01	-0,31
1948	SACOL2170	3638	1288	258	-1,50	-3,82
1949	**SACOL2171**	5546	4206	841	-0,40	-2,72
1950	**SACOL2173**	62	1054	211	4,09	1,77
1951	**SACOL2174**	4938	57055	11411	3,53	1,21
1952	**SACOL2175**	13297	187179	37436	3,82	1,49
1953	**SACOL2176**	9965	90291	18058	3,18	0,86
1954	**SACOL2177**	8575	26927	5385	1,65	-0,67
1955	**SACOL2178**	8938	38393	7679	2,10	-0,22
1956	**SACOL2179**	10827	27635	5527	1,35	-0,97
1957	SACOL2180	200	1337	267	2,74	0,42
1958	**SACOL2181**	770	3431	686	2,16	-0,17
1959	SACOL2182	303	3275	655	3,43	1,11
1960	**SACOL2183**	216	3211	642	3,89	1,57
1961	SACOL2184	168	2374	475	3,82	1,50
1962	SACOL2185	323	4970	994	3,94	1,62
1963	SACOL2186	141	3351	670	4,58	2,25
1964	**SACOL2188**	3068	7366	1473	1,26	-1,06
1965	**SACOL2189**	2117	7529	1506	1,83	-0,49
1966	SACOL2190	2735	2546	509	-0,10	-2,43
1967	SACOL2191	1792	1922	384	0,10	-2,22
1968	**SACOL2192**	2731	10339	2068	1,92	-0,40
1969	SACOL2193	1267	3866	773	1,61	-0,71
1970	**SACOL2194**	206	733	147	1,83	-0,49
1971	SACOL2195	5085	7120	1424	0,49	-1,84
1972	**SACOL2196**	4585	9414	1883	1,04	-1,28
1973	**SACOL2197**	2642	273142	54628	6,69	4,37
1974	**SACOL2198**	385	959	192	1,32	-1,00
1975	SACOL2199	1485	2971	594	1,00	-1,32
1976	SACOL2200	3571	5243	1049	0,55	-1,77
1977	SACOL2201	12522	13380	2676	0,10	-2,23
1978	SACOL2202	7609	8555	1711	0,17	-2,15
1979	**SACOL2203**	5650	7401	1480	0,39	-1,93
1980	SACOL2204	14059	14786	2957	0,07	-2,25
1981	**SACOL2206**	58305	8357	1671	-2,80	-5,12
1982	**SACOL2207**	102703	15822	3164	-2,70	-5,02
1983	**SACOL2208**	23020	11623	2325	-0,99	-3,31
1984	**SACOL2209**	20576	9768	1954	-1,07	-3,40
1985	**SACOL2210**	8665	4548	910	-0,93	-3,25
1986	**SACOL2211**	12324	6711	1342	-0,88	-3,20
1987	**SACOL2212**	77673	14355	2871	-2,44	-4,76
1988	**SACOL2213**	52860	7770	1554	-2,77	-5,09
1989	**SACOL2214**	110227	15835	3167	-2,80	-5,12
1990	**SACOL2215**	114097	25601	5120	-2,16	-4,48
1991	**SACOL2217**	141889	25955	5191	-2,45	-4,77
1992	**SACOL2218**	155215	25507	5101	-2,61	-4,93
1993	**SACOL2219**	144475	23150	4630	-2,64	-4,96
1994	**SACOL2220**	107162	23084	4617	-2,21	-4,54
1995	**SACOL2221**	140233	17139	3428	-3,03	-5,35
1996	**SACOL2222**	151706	18590	3718	-3,03	-5,35
1997	**SACOL2223**	179303	19734	3947	-3,18	-5,51
1998	**SACOL2224**	134304	16148	3230	-3,06	-5,38
1999	**SACOL2225**	122036	13602	2720	-3,17	-5,49
2000	SACOL2226	179310	19934	3987	-3,17	-5,49
2001	**SACOL2228**	212408	20367	4073	-3,38	-5,70
2002	**SACOL2229**	154770	18827	3765	-3,04	-5,36
2003	**SACOL2230**	199755	22033	4407	-3,18	-5,50
2004	**SACOL2231**	123191	15683	3137	-2,97	-5,30
2005	**SACOL2232**	110042	13833	2767	-2,99	-5,31
2006	**SACOL2233**	119803	12607	2521	-3,25	-5,57
2007	**SACOL2234**	146955	21287	4257	-2,79	-5,11
2008	**SACOL2235**	163138	18972	3794	-3,10	-5,43
2009	**SACOL2236**	228532	20173	4035	-3,50	-5,82
2010	**SACOL2237**	132548	14728	2946	-3,17	-5,49
2011	**SACOL2238**	170513	18717	3743	-3,19	-5,51
2012	**SACOL2239**	149933	17961	3592	-3,06	-5,38
2013	**SACOL2240**	218170	21830	4366	-3,32	-5,64
2014	SACOL2241	24919	26913	5383	0,11	-2,21
2015	**SACOL2242**	9647	4533	907	-1,09	-3,41
2016	**SACOL2243**	16679	13601	2720	-0,29	-2,62

a) *S. aureus* COL Locus. Written in bold indicates that the corresponding protein was identified.
b) *LOWESS normalized background subtracted signal intensities.*
c) *Scaled stationary phase signal intensities to account for decrease in total RNA during stationary phase.*
d) *Log_2 of stationary phase versus exponential growth ratios.*
e) *Log_2 of scaled stationary phase versus exponential growth ratios.*

Supplementary Table 1: Signal intensities and calculated ratios from DNA microarray experiment

	SACOL[a]	signal intensity[b] exponential growth	signal intensity[b] stationary phase	signal intensity corrected[c] stationary phase	induction[d] stat/exp	induction corrected[e] stat/exp
2017	**SACOL2245**	3257	4836	967	0,57	-1,75
2018	**SACOL2246**	4511	1980	396	-1,19	-3,51
2019	SACOL2247	572	283	57	-1,02	-3,34
2020	SACOL2248	782	834	167	0,09	-2,23
2021	SACOL2250	7991	7153	1431	-0,16	-2,48
2022	SACOL2251	4521	5843	1169	0,37	-1,95
2023	**SACOL2252**	11390	14261	2852	0,32	-2,00
2024	**SACOL2253**	18386	19558	3912	0,09	-2,23
2025	**SACOL2255**	4949	11486	2297	1,21	-1,11
2026	SACOL2256	4563	1427	285	-1,68	-4,00
2027	**SACOL2257**	3753	1045	209	-1,85	-4,17
2028	SACOL2258	3393	2325	465	-0,55	-2,87
2029	**SACOL2261**	19881	17606	3521	-0,18	-2,50
2030	**SACOL2262**	16367	17918	3584	0,13	-2,19
2031	SACOL2263	23330	22210	4442	-0,07	-2,39
2032	**SACOL2264**	24858	19062	3812	-0,38	-2,70
2033	**SACOL2265**	28391	20235	4047	-0,49	-2,81
2034	**SACOL2266**	29185	22221	4444	-0,39	-2,72
2035	**SACOL2267**	4003	3040	608	-0,40	-2,72
2036	**SACOL2268**	26923	14541	2908	-0,89	-3,21
2037	**SACOL2269**	28275	12808	2562	-1,14	-3,46
2038	**SACOL2270**	12232	6645	1329	-0,88	-3,20
2039	SACOL2271	12616	8021	1604	-0,65	-2,98
2040	**SACOL2272**	16061	14496	2899	-0,15	-2,47
2041	**SACOL2273**	7944	14512	2902	0,87	-1,45
2042	**SACOL2274**	7699	2351	470	-1,71	-4,03
2043	**SACOL2275**	39990	9267	1853	-2,11	-4,43
2044	**SACOL2276**	3788	2683	537	-0,50	-2,82
2045	**SACOL2277**	17032	2748	550	-2,63	-4,95
2046	**SACOL2278**	1726	3830	766	1,15	-1,17
2047	SACOL2279	193	169	34	-0,19	-2,51
2048	**SACOL2280**	11734	4416	883	-1,41	-3,73
2049	**SACOL2281**	8143	3618	724	-1,17	-3,49
2050	**SACOL2282**	11955	4620	924	-1,37	-3,69
2051	**SACOL2283**	4651	3138	628	-0,57	-2,89
2052	**SACOL2284**	4132	2625	525	-0,65	-2,98
2053	**SACOL2285**	3796	2600	520	-0,55	-2,87
2054	SACOL2286	3716	2578	516	-0,53	-2,85
2055	**SACOL2287**	4617	5628	1126	0,29	-2,04
2056	SACOL2289	2354	661	132	-1,83	-4,15
2057	SACOL2290	3164	2893	579	-0,13	-2,45
2058	**SACOL2291**	61877	3463	693	-4,16	-6,48
2059	**SACOL2292**	8844	13845	2769	0,65	-1,68
2060	**SACOL2293**	25070	33580	6716	0,42	-1,90
2061	SACOL2294	1704	3333	667	0,97	-1,35
2062	SACOL2295	18696	28870	5774	0,63	-1,70
2063	**SACOL2296**	2185	11387	2277	2,38	0,06
2064	**SACOL2297**	10758	6485	1297	-0,73	-3,05
2065	SACOL2298	5890	2298	460	-1,36	-3,68
2066	SACOL2299	6905	1676	335	-2,04	-4,36
2067	**SACOL2300**	1296	10502	2100	3,02	0,70
2068	**SACOL2301**	9523	52389	10478	2,46	0,14
2069	**SACOL2302**	5193	11908	2382	1,20	-1,12
2070	**SACOL2303**	8879	12703	2541	0,52	-1,81
2071	**SACOL2304**	12837	16476	3295	0,36	-1,96
2072	SACOL2305	391	313	63	-0,32	-2,64
2073	**SACOL2306**	14199	2103	421	-2,76	-5,08
2074	SACOL2307	435	348	70	-0,32	-2,64
2075	SACOL2308	8932	27091	5418	1,60	-0,72
2076	**SACOL2309**	15353	15315	3063	0,00	-2,33
2077	SACOL2310	5418	6523	1305	0,27	-2,05
2078	SACOL2311	4971	6188	1238	0,32	-2,01
2079	SACOL2312	7078	6089	1218	-0,22	-2,54
2080	**SACOL2313**	5934	6086	1217	0,04	-2,29
2081	**SACOL2314**	3826	2254	451	-0,76	-3,09
2082	**SACOL2315**	1255	969	194	-0,37	-2,70
2083	**SACOL2316**	1152	5149	1030	2,16	-0,16
2084	**SACOL2317**	1370	611	122	-1,16	-3,49
2085	**SACOL2318**	9620	11451	2290	0,25	-2,07
2086	**SACOL2319**	10871	14026	2805	0,37	-1,95
2087	SACOL2320	1231	6506	1301	2,40	0,08
2088	**SACOL2321**	2286	17139	3428	2,91	0,58

a) *S. aureus* COL Locus. Written in bold indicates that the corresponding protein was identified.
b) *LOWESS normalized background subtracted signal intensities.*
c) *Scaled stationary phase signal intensities to account for decrease in total RNA during stationary phase.*
d) *Log_2 of stationary phase versus exponential growth ratios.*
e) *Log_2 of scaled stationary phase versus exponential growth ratios.*

Supplementary Table 1: Signal intensities and calculated ratios from DNA microarray experiment

	SACOL[a]	signal intensity[b] exponential growth	signal intensity[b] stationary phase	signal intensity corrected[c] stationary phase	induction[d] stat/exp	induction corrected[e] stat/exp
2089	**SACOL2322**	2921	4277	855	0,55	-1,77
2090	**SACOL2323**	622	3161	632	2,34	0,02
2091	**SACOL2324**	671	3123	625	2,22	-0,10
2092	**SACOL2325**	1374	1405	281	0,03	-2,29
2093	SACOL2326	392	493	99	0,33	-1,99
2094	**SACOL2327**	5930	56532	11306	3,25	0,93
2095	**SACOL2328**	2878	8218	1644	1,51	-0,81
2096	**SACOL2329**	2297	2531	506	0,14	-2,18
2097	**SACOL2330**	1901	4425	885	1,22	-1,10
2098	**SACOL2332**	4980	2962	592	-0,75	-3,07
2099	SACOL2333	10910	4708	942	-1,21	-3,53
2100	**SACOL2334**	6283	5584	1117	-0,17	-2,49
2101	**SACOL2335**	9575	7012	1402	-0,45	-2,77
2102	**SACOL2338**	14628	12334	2467	-0,25	-2,57
2103	**SACOL2339**	922	718	144	-0,36	-2,68
2104	**SACOL2340**	5029	2342	468	-1,10	-3,42
2105	**SACOL2341**	19295	18611	3722	-0,05	-2,37
2106	**SACOL2342**	13098	11871	2374	-0,14	-2,46
2107	**SACOL2343**	5469	13912	2782	1,35	-0,97
2108	**SACOL2344**	7846	25125	5025	1,68	-0,64
2109	**SACOL2345**	1889	4289	858	1,18	-1,14
2110	**SACOL2346**	14415	9279	1856	-0,64	-2,96
2111	**SACOL2347**	3823	647	129	-2,56	-4,88
2112	**SACOL2348**	2758	305	61	-3,18	-5,50
2113	SACOL2349	4707	7272	1454	0,63	-1,69
2114	**SACOL2350**	10322	4696	939	-1,14	-3,46
2115	**SACOL2352**	5176	3870	774	-0,42	-2,74
2116	**SACOL2353**	4486	4551	910	0,02	-2,30
2117	**SACOL2354**	877	2644	529	1,59	-0,73
2118	SACOL2356	351	769	154	1,13	-1,19
2119	SACOL2357	588	795	159	0,44	-1,89
2120	SACOL2358	2219	5144	1029	1,21	-1,11
2121	**SACOL2359**	4187	9861	1972	1,24	-1,09
2122	**SACOL2360**	1594	12785	2557	3,00	0,68
2123	SACOL2361	1941	18257	3651	3,23	0,91
2124	**SACOL2362**	12092	14784	2957	0,29	-2,03
2125	**SACOL2363**	86	53	11	-0,69	-3,01
2126	**SACOL2364**	16833	14372	2874	-0,23	-2,55
2127	**SACOL2365**	2371	23475	4695	3,31	0,99
2128	**SACOL2366**	1616	12518	2504	2,95	0,63
2129	**SACOL2367**	5669	5296	1059	-0,10	-2,42
2130	**SACOL2368**	4436	6968	1394	0,65	-1,67
2131	**SACOL2369**	5236	7523	1505	0,52	-1,80
2132	SACOL2371	4668	3494	699	-0,42	-2,74
2133	SACOL2372	86	91	18	0,09	-2,24
2134	**SACOL2373**	250	318	64	0,34	-1,98
2135	**SACOL2374**	12008	13021	2604	0,12	-2,21
2136	**SACOL2375**	12242	12143	2429	-0,01	-2,33
2137	**SACOL2376**	3253	1149	230	-1,50	-3,82
2138	SACOL2377	1952	3587	717	0,88	-1,44
2139	**SACOL2378**	1619	1488	298	-0,12	-2,44
2140	**SACOL2379**	5116	45992	9198	3,17	0,85
2141	SACOL2380	95	91	18	-0,08	-2,40
2142	**SACOL2381**	9003	4536	907	-0,99	-3,31
2143	**SACOL2382**	5942	1884	377	-1,66	-3,98
2144	**SACOL2383**	12581	3374	675	-1,90	-4,22
2145	**SACOL2384**	4946	4224	845	-0,23	-2,55
2146	**SACOL2385**	5816	7089	1418	0,29	-2,04
2147	SACOL2386	290	196	39	-0,56	-2,88
2148	SACOL2388	498	775	155	0,64	-1,68
2149	SACOL2389	5882	9616	1923	0,71	-1,61
2150	SACOL2390	8997	16966	3393	0,92	-1,41
2151	SACOL2391	9293	19001	3800	1,03	-1,29
2152	SACOL2392	12334	17902	3580	0,54	-1,78
2153	SACOL2393	2336	3028	606	0,37	-1,95
2154	SACOL2394	1853	2800	560	0,60	-1,73
2155	SACOL2395	1349	2349	470	0,80	-1,52
2156	SACOL2396	1175	3927	785	1,74	-0,58
2157	SACOL2397	1130	3214	643	1,51	-0,81
2158	SACOL2398	599	2002	400	1,74	-0,58
2159	SACOL2399	450	1159	232	1,37	-0,96
2160	**SACOL2400**	4098	3034	607	-0,43	-2,76

a) *S. aureus* COL Locus. Written in bold indicates that the corresponding protein was identified.
b) *LOWESS normalized background subtracted signal intensities.*
c) *Scaled stationary phase signal intensities to account for decrease in total RNA during stationary phase.*
d) *Log_2 of stationary phase versus exponential growth ratios.*
e) *Log_2 of scaled stationary phase versus exponential growth ratios.*

Supplementary Table 1: Signal intensities and calculated ratios from DNA microarray experiment

	SACOL[a]	signal intensity[b] exponential growth	signal intensity[b] stationary phase	signal intensity corrected[c] stationary phase	induction[d] stat/exp	induction corrected[e] stat/exp
2161	**SACOL2401**	10609	903	181	-3,55	-5,88
2162	**SACOL2402**	1166	1133	227	-0,04	-2,36
2163	**SACOL2403**	3527	2356	471	-0,58	-2,90
2164	SACOL2404	666	2302	460	1,79	-0,53
2165	SACOL2405	1963	5572	1114	1,50	-0,82
2166	**SACOL2407**	9625	7717	1543	-0,32	-2,64
2167	SACOL2408	8365	6337	1267	-0,40	-2,72
2168	SACOL2409	171	310	62	0,85	-1,47
2169	**SACOL2410**	11511	4485	897	-1,36	-3,68
2170	**SACOL2411**	10069	3970	794	-1,34	-3,66
2171	**SACOL2412**	20798	4401	880	-2,24	-4,56
2172	**SACOL2413**	3656	5250	1050	0,52	-1,80
2173	SACOL2414	1471	337	67	-2,13	-4,45
2174	**SACOL2415**	2499	14627	2925	2,55	0,23
2175	**SACOL2416**	2160	24705	4941	3,52	1,19
2176	**SACOL2418**	18056	5355	1071	-1,75	-4,08
2177	SACOL2419	146	1307	261	3,16	0,84
2178	SACOL2420	48	140	28	1,53	-0,79
2179	SACOL2421	320	487	97	0,61	-1,71
2180	SACOL2422	482	797	159	0,73	-1,60
2181	SACOL2423	10905	3740	748	-1,54	-3,87
2182	**SACOL2424**	32847	10209	2042	-1,69	-4,01
2183	**SACOL2425**	37747	14124	2825	-1,42	-3,74
2184	**SACOL2426**	34931	14076	2815	-1,31	-3,63
2185	**SACOL2427**	44550	16725	3345	-1,41	-3,74
2186	**SACOL2428**	26889	17461	3492	-0,62	-2,94
2187	SACOL2429	377	591	118	0,65	-1,68
2188	SACOL2430	731	563	113	-0,38	-2,70
2189	SACOL2431	416	304	61	-0,45	-2,77
2190	SACOL2433	4130	15223	3045	1,88	-0,44
2191	**SACOL2434**	2355	9293	1859	1,98	-0,34
2192	**SACOL2435**	18478	29521	5904	0,68	-1,65
2193	**SACOL2436**	32024	57109	11422	0,83	-1,49
2194	**SACOL2437**	3783	2689	538	-0,49	-2,81
2195	**SACOL2438**	3297	2489	498	-0,41	-2,73
2196	**SACOL2439**	6535	8769	1754	0,42	-1,90
2197	SACOL2440	1105	1263	253	0,19	-2,13
2198	**SACOL2441**	759	4538	908	2,58	0,26
2199	**SACOL2442**	2798	1851	370	-0,60	-2,92
2200	**SACOL2443**	1772	3278	656	0,89	-1,43
2201	**SACOL2445**	3784	9369	1874	1,31	-1,01
2202	**SACOL2446**	9677	4985	997	-0,96	-3,28
2203	**SACOL2448**	12061	5323	1065	-1,18	-3,50
2204	**SACOL2449**	10245	5642	1128	-0,86	-3,18
2205	**SACOL2450**	8570	13612	2722	0,67	-1,65
2206	**SACOL2451**	7188	11019	2204	0,62	-1,71
2207	**SACOL2452**	7580	12227	2445	0,69	-1,63
2208	**SACOL2453**	8065	12209	2442	0,60	-1,72
2209	SACOL2454	284	160	32	-0,83	-3,15
2210	**SACOL2456**	10294	7569	1514	-0,44	-2,77
2211	SACOL2458	5073	3661	732	-0,47	-2,79
2212	**SACOL2459**	7135	53601	10720	2,91	0,59
2213	SACOL2460	3270	3047	609	-0,10	-2,42
2214	**SACOL2461**	4760	11366	2273	1,26	-1,07
2215	**SACOL2462**	9815	24417	4883	1,31	-1,01
2216	**SACOL2463**	8054	3922	784	-1,04	-3,36
2217	**SACOL2464**	3325	7386	1477	1,15	-1,17
2218	**SACOL2465**	3549	8206	1641	1,21	-1,11
2219	SACOL2466	3765	2990	598	-0,33	-2,65
2220	**SACOL2467**	7384	6864	1373	-0,11	-2,43
2221	**SACOL2469**	1816	3279	656	0,85	-1,47
2222	**SACOL2470**	1925	519	104	-1,89	-4,21
2223	**SACOL2471**	6411	928	186	-2,79	-5,11
2224	SACOL2472	3618	199	40	-4,18	-6,51
2225	SACOL2473	6324	255	51	-4,63	-6,95
2226	SACOL2474	7290	300	60	-4,60	-6,92
2227	SACOL2475	4409	284	57	-3,96	-6,28
2228	**SACOL2476**	4043	559	112	-2,86	-5,18
2229	SACOL2477	1054	105	21	-3,33	-5,65
2230	SACOL2478	3763	769	154	-2,29	-4,61
2231	SACOL2479	3439	708	142	-2,28	-4,60
2232	SACOL2481	553	4526	905	3,03	0,71

a) *S. aureus* COL Locus. Written in bold indicates that the corresponding protein was identified.
b) *LOWESS normalized background subtracted signal intensities.*
c) *Scaled stationary phase signal intensities to account for decrease in total RNA during stationary phase.*
d) *Log_2 of stationary phase versus exponential growth ratios.*
e) *Log_2 of scaled stationary phase versus exponential growth ratios.*

Supplementary Table 1: Signal intensities and calculated ratios from DNA microarray experiment

	SACOL[a]	signal intensity[b] exponential growth	signal intensity[b] stationary phase	signal intensity corrected[c] stationary phase	induction[d] stat/exp	induction corrected[e] stat/exp
2233	**SACOL2483**	10681	12897	2579	0,27	-2,05
2234	**SACOL2484**	13893	58454	11691	2,07	-0,25
2235	SACOL2486	2888	1498	300	-0,95	-3,27
2236	SACOL2487	5904	5292	1058	-0,16	-2,48
2237	**SACOL2488**	11310	13956	2791	0,30	-2,02
2238	**SACOL2489**	13082	13343	2669	0,03	-2,29
2239	SACOL2491	35384	7592	1518	-2,22	-4,54
2240	SACOL2492	410	261	52	-0,65	-2,97
2241	SACOL2493	1979	2587	517	0,39	-1,94
2242	SACOL2495	9793	5449	1090	-0,85	-3,17
2243	SACOL2496	15341	10325	2065	-0,57	-2,89
2244	SACOL2497	5868	5378	1076	-0,13	-2,45
2245	**SACOL2498**	6904	5384	1077	-0,36	-2,68
2246	**SACOL2499**	4155	3279	656	-0,34	-2,66
2247	**SACOL2500**	5448	6054	1211	0,15	-2,17
2248	**SACOL2501**	1848	2123	425	0,20	-2,12
2249	SACOL2502	5365	12844	2569	1,26	-1,06
2250	SACOL2503	1921	2495	499	0,38	-1,94
2251	SACOL2504	1603	2330	466	0,54	-1,78
2252	SACOL2505	1810	2351	470	0,38	-1,94
2253	SACOL2506	720	89	18	-3,01	-5,34
2254	SACOL2507	139	100	20	-0,48	-2,80
2255	**SACOL2508**	4343	3449	690	-0,33	-2,65
2256	SACOL2509	1280	734	147	-0,80	-3,13
2257	SACOL2510	58	92	18	0,66	-1,66
2258	SACOL2511	2771	20142	4028	2,86	0,54
2259	SACOL2513	237	200	40	-0,24	-2,56
2260	**SACOL2514**	1537	653	131	-1,24	-3,56
2261	**SACOL2515**	3199	355	71	-3,17	-5,49
2262	**SACOL2516**	7939	1164	233	-2,77	-5,09
2263	SACOL2517	8004	6470	1294	-0,31	-2,63
2264	**SACOL2518**	11140	6479	1296	-0,78	-3,10
2265	**SACOL2519**	9461	32329	6466	1,77	-0,55
2266	**SACOL2520**	19490	2713	543	-2,84	-5,17
2267	**SACOL2521**	1070	6949	1390	2,70	0,38
2268	SACOL2522	5411	13523	2705	1,32	-1,00
2269	SACOL2523	347	808	162	1,22	-1,10
2270	SACOL2524	1093	1213	243	0,15	-2,17
2271	**SACOL2525**	836	970	194	0,22	-2,11
2272	**SACOL2527**	2565	13678	2736	2,41	0,09
2273	**SACOL2528**	1668	1642	328	-0,02	-2,34
2274	**SACOL2529**	885	11832	2366	3,74	1,42
2275	SACOL2530	4061	47208	9442	3,54	1,22
2276	**SACOL2531**	3739	37271	7454	3,32	1,00
2277	**SACOL2532**	941	1635	327	0,80	-1,52
2278	**SACOL2533**	2099	9062	1812	2,11	-0,21
2279	**SACOL2534**	11188	111460	22292	3,32	0,99
2280	**SACOL2535**	6276	2004	401	-1,65	-3,97
2281	**SACOL2536**	1508	1145	229	-0,40	-2,72
2282	SACOL2537	395	437	87	0,15	-2,17
2283	SACOL2538	445	824	165	0,89	-1,43
2284	**SACOL2539**	13531	12168	2434	-0,15	-2,48
2285	SACOL2541	3434	4810	962	0,49	-1,84
2286	SACOL2542	1508	2097	419	0,48	-1,85
2287	SACOL2544	3232	3290	658	0,03	-2,30
2288	SACOL2545	9907	4996	999	-0,99	-3,31
2289	SACOL2546	9727	4756	951	-1,03	-3,35
2290	SACOL2547	773	5411	1082	2,81	0,49
2291	**SACOL2548**	1421	3611	722	1,35	-0,98
2292	**SACOL2549**	21481	30046	6009	0,48	-1,84
2293	SACOL2550	10372	16924	3385	0,71	-1,62
2294	**SACOL2551**	8332	11260	2252	0,43	-1,89
2295	**SACOL2552**	21084	1230	246	-4,10	-6,42
2296	**SACOL2553**	10984	50277	10055	2,19	-0,13
2297	SACOL2554	18894	101299	20260	2,42	0,10
2298	SACOL2554.1	2455	235	47	-3,38	-5,71
2299	**SACOL2555**	1842	1731	346	-0,09	-2,41
2300	SACOL2556	19867	2478	496	-3,00	-5,32
2301	**SACOL2557**	7705	3687	737	-1,06	-3,39
2302	**SACOL2560**	6193	7589	1518	0,29	-2,03
2303	**SACOL2561**	6756	3081	616	-1,13	-3,45
2304	**SACOL2562**	4266	6237	1247	0,55	-1,77

a) *S. aureus* COL Locus. Written in bold indicates that the corresponding protein was identified.
b) *LOWESS normalized background subtracted signal intensities.*
c) *Scaled stationary phase signal intensities to account for decrease in total RNA during stationary phase.*
d) *Log_2 of stationary phase versus exponential growth ratios.*
e) *Log_2 of scaled stationary phase versus exponential growth ratios.*

Supplementary Table 1: Signal intensities and calculated ratios from DNA microarray experiment

	SACOL[a]	signal intensity[b] exponential growth	signal intensity[b] stationary phase	signal intensity corrected[c] stationary phase	induction[d] stat/exp	induction corrected[e] stat/exp
2305	**SACOL2563**	1582	16193	3239	3,36	1,03
2306	SACOL2564	704	153	31	-2,20	-4,52
2307	SACOL2565	1948	310	62	-2,65	-4,98
2308	**SACOL2566**	457	972	194	1,09	-1,23
2309	SACOL2567	9408	5079	1016	-0,89	-3,21
2310	SACOL2568	415	6733	1347	4,02	1,70
2311	**SACOL2569**	2401	43995	8799	4,20	1,87
2312	**SACOL2570**	815	3814	763	2,23	-0,10
2313	SACOL2571	275	512	102	0,90	-1,42
2314	**SACOL2572**	4801	5603	1121	0,22	-2,10
2315	SACOL2573	2378	1516	303	-0,65	-2,97
2316	**SACOL2574**	9628	11207	2241	0,22	-2,10
2317	**SACOL2575**	18998	33794	6759	0,83	-1,49
2318	**SACOL2576**	11062	31703	6341	1,52	-0,80
2319	**SACOL2577**	10036	29745	5949	1,57	-0,75
2320	SACOL2578	11904	46521	9304	1,97	-0,36
2321	**SACOL2579**	8515	36444	7289	2,10	-0,22
2322	SACOL2580	5850	19836	3967	1,76	-0,56
2323	**SACOL2581**	9442	2267	453	-2,06	-4,38
2324	**SACOL2582**	18486	6447	1289	-1,52	-3,84
2325	**SACOL2583**	8146	2265	453	-1,85	-4,17
2326	**SACOL2584**	15106	10117	2023	-0,58	-2,90
2327	**SACOL2585**	9595	1187	237	-3,01	-5,34
2328	**SACOL2587**	1466	2219	444	0,60	-1,72
2329	SACOL2588	1677	4807	961	1,52	-0,80
2330	SACOL2589	980	2990	598	1,61	-0,71
2331	**SACOL2590**	1168	1223	245	0,07	-2,26
2332	**SACOL2591**	989	896	179	-0,14	-2,46
2333	SACOL2592	2326	1629	326	-0,51	-2,84
2334	SACOL2593	3469	3108	622	-0,16	-2,48
2335	**SACOL2594**	2701	2518	504	-0,10	-2,42
2336	**SACOL2596**	12115	34503	6901	1,51	-0,81
2337	**SACOL2597**	3940	18913	3783	2,26	-0,06
2338	SACOL2598	3797	1944	389	-0,97	-3,29
2339	SACOL2599	3016	1195	239	-1,34	-3,66
2340	SACOL2600	2183	2759	552	0,34	-1,98
2341	**SACOL2601**	3732	7790	1558	1,06	-1,26
2342	SACOL2602	9593	21530	4306	1,17	-1,16
2343	SACOL2603	6721	12728	2546	0,92	-1,40
2344	**SACOL2605**	1513	12019	2404	2,99	0,67
2345	**SACOL2606**	17199	5523	1105	-1,64	-3,96
2346	**SACOL2607**	490	258	52	-0,93	-3,25
2347	**SACOL2608**	3956	2102	420	-0,91	-3,23
2348	**SACOL2609**	6474	6675	1335	0,04	-2,28
2349	SACOL2610	4750	2885	577	-0,72	-3,04
2350	SACOL2612	1425	1173	235	-0,28	-2,60
2351	SACOL2613	6438	5686	1137	-0,18	-2,50
2352	**SACOL2614**	8968	9697	1939	0,11	-2,21
2353	**SACOL2615**	12476	7217	1443	-0,79	-3,11
2354	**SACOL2616**	9438	6798	1360	-0,47	-2,80
2355	SACOL2617	3161	3419	684	0,11	-2,21
2356	**SACOL2618**	2723	9178	1836	1,75	-0,57
2357	**SACOL2619**	37252	15143	3029	-1,30	-3,62
2358	**SACOL2620**	9509	10226	2045	0,10	-2,22
2359	SACOL2621	2265	11451	2290	2,34	0,02
2360	**SACOL2622**	14871	26957	5391	0,86	-1,46
2361	**SACOL2623**	26691	9979	1996	-1,42	-3,74
2362	**SACOL2624**	16029	35638	7128	1,15	-1,17
2363	**SACOL2625**	1938	21662	4332	3,48	1,16
2364	SACOL2626	763	754	151	-0,02	-2,34
2365	**SACOL2627**	19343	1208	242	-4,00	-6,32
2366	**SACOL2628**	21815	1572	314	-3,79	-6,12
2367	**SACOL2630**	8122	3315	663	-1,29	-3,61
2368	SACOL2631	4231	4180	836	-0,02	-2,34
2369	**SACOL2632**	14159	676	135	-4,39	-6,71
2370	SACOL2634	392	321	64	-0,29	-2,61
2371	SACOL2635	1811	1976	395	0,13	-2,20
2372	**SACOL2636**	3355	3149	630	-0,09	-2,41
2373	SACOL2638	17095	13764	2753	-0,31	-2,63
2374	**SACOL2639**	4953	8082	1616	0,71	-1,62
2375	**SACOL2641**	2235	5371	1074	1,26	-1,06
2376	SACOL2642	4885	22317	4463	2,19	-0,13

a) *S. aureus* COL Locus. Written in bold indicates that the corresponding protein was identified.
b) *LOWESS normalized background subtracted signal intensities.*
c) *Scaled stationary phase signal intensities to account for decrease in total RNA during stationary phase.*
d) *Log_2 of stationary phase versus exponential growth ratios.*
e) *Log_2 of scaled stationary phase versus exponential growth ratios.*

Supplementary Table 1: Signal intensities and calculated ratios from DNA microarray experiment

	SACOL[a]	signal intensity[b] exponential growth	signal intensity[b] stationary phase	signal intensity corrected[c] stationary phase	induction[d] stat/exp	induction corrected[e] stat/exp
2377	SACOL2643	2432	1833	367	-0,41	-2,73
2378	SACOL2644	2605	2245	449	-0,21	-2,54
2379	**SACOL2645**	14467	9550	1910	-0,60	-2,92
2380	SACOL2646	11853	8624	1725	-0,46	-2,78
2381	**SACOL2647**	10320	6986	1397	-0,56	-2,88
2382	SACOL2648	201	216	43	0,11	-2,22
2383	**SACOL2650**	18175	14201	2840	-0,36	-2,68
2384	**SACOL2651**	4243	4367	873	0,04	-2,28
2385	**SACOL2652**	44090	4183	837	-3,40	-5,72
2386	SACOL2653	1592	937	187	-0,77	-3,09
2387	SACOL2654	3814	2304	461	-0,73	-3,05
2388	SACOL2655	709	1382	276	0,96	-1,36
2389	SACOL2656	287	535	107	0,90	-1,42
2390	SACOL2657	799	1398	280	0,81	-1,51
2391	SACOL2658	150	85	17	-0,81	-3,13
2392	**SACOL2659**	997	800	160	-0,32	-2,64
2393	**SACOL2660**	13261	56000	11200	2,08	-0,24
2394	SACOL2661	1962	6155	1231	1,65	-0,67
2395	SACOL2662	324	616	123	0,92	-1,40
2396	**SACOL2663**	8430	4373	875	-0,95	-3,27
2397	**SACOL2664**	3730	2600	520	-0,52	-2,84
2398	**SACOL2665**	17341	5679	1136	-1,61	-3,93
2399	**SACOL2666**	29039	19747	3949	-0,56	-2,88
2400	**SACOL2667**	2275	6265	1253	1,46	-0,86
2401	SACOL2668	3951	14200	2840	1,85	-0,48
2402	SACOL2669	3841	11086	2217	1,53	-0,79
2403	SACOL2670	12879	46541	9308	1,85	-0,47
2404	**SACOL2671**	10039	39225	7845	1,97	-0,36
2405	SACOL2672	4363	2132	426	-1,03	-3,35
2406	SACOL2673	2068	1185	237	-0,80	-3,12
2407	**SACOL2674**	2790	1591	318	-0,81	-3,13
2408	SACOL2675	4646	2451	490	-0,92	-3,24
2409	SACOL2676	11358	19252	3850	0,76	-1,56
2410	SACOL2677	910	408	82	-1,16	-3,48
2411	**SACOL2678**	1497	1453	291	-0,04	-2,37
2412	SACOL2680	1828	943	189	-0,95	-3,28
2413	**SACOL2681**	6005	22064	4413	1,88	-0,44
2414	**SACOL2682**	3134	8463	1693	1,43	-0,89
2415	SACOL2683	1367	1050	210	-0,38	-2,70
2416	SACOL2684	1337	821	164	-0,70	-3,03
2417	SACOL2685	468	210	42	-1,16	-3,48
2418	SACOL2686	2028	374	75	-2,44	-4,76
2419	SACOL2687	834	95	19	-3,13	-5,45
2420	**SACOL2688**	6935	5017	1003	-0,47	-2,79
2421	SACOL2689	743	1528	306	1,04	-1,28
2422	SACOL2690	1270	1819	364	0,52	-1,80
2423	SACOL2691	1027	1250	250	0,28	-2,04
2424	**SACOL2694**	1378	28917	5783	4,39	2,07
2425	SACOL2696	2490	2442	488	-0,03	-2,35
2426	SACOL2697	6123	4803	961	-0,35	-2,67
2427	SACOL2698	5821	3369	674	-0,79	-3,11
2428	SACOL2699	9117	3511	702	-1,38	-3,70
2429	SACOL2700	4076	1239	248	-1,72	-4,04
2430	**SACOL2701**	6501	2205	441	-1,56	-3,88
2431	SACOL2702	3816	963	193	-1,99	-4,31
2432	SACOL2703	2986	698	140	-2,10	-4,42
2433	**SACOL2704**	2966	731	146	-2,02	-4,34
2434	**SACOL2705**	27264	5940	1188	-2,20	-4,52
2435	**SACOL2706**	15947	2798	560	-2,51	-4,83
2436	**SACOL2707**	18728	3264	653	-2,52	-4,84
2437	**SACOL2708**	11108	3157	631	-1,82	-4,14
2438	**SACOL2709**	11853	3508	702	-1,76	-4,08
2439	**SACOL2710**	7669	2603	521	-1,56	-3,88
2440	**SACOL2711**	844	14829	2966	4,14	1,81
2441	**SACOL2712**	597	2352	470	1,98	-0,34
2442	SACOL2713	5682	3184	637	-0,84	-3,16
2443	SACOL2714	993	1386	277	0,48	-1,84
2444	**SACOL2715**	2885	6765	1353	1,23	-1,09
2445	SACOL2716	6556	7294	1459	0,15	-2,17
2446	**SACOL2717**	1694	4530	906	1,42	-0,90
2447	**SACOL2718**	3665	1432	286	-1,36	-3,68
2448	SACOL2719	4700	4238	848	-0,15	-2,47

a) *S. aureus* COL Locus. Written in bold indicates that the corresponding protein was identified.
b) *LOWESS normalized background subtracted signal intensities.*
c) *Scaled stationary phase signal intensities to account for decrease in total RNA during stationary phase.*
d) *Log_2 of stationary phase versus exponential growth ratios.*
e) *Log_2 of scaled stationary phase versus exponential growth ratios.*

Supplementary Table 1: Signal intensities and calculated ratios from DNA microarray experiment

	SACOL[a]	signal intensity[b] exponential growth	signal intensity[b] stationary phase	signal intensity corrected[c] stationary phase	induction[d] stat/exp	induction corrected[e] stat/exp
2449	SACOL2720	1543	8270	1654	2,42	0,10
2450	SACOL2721	2596	5037	1007	0,96	-1,37
2451	**SACOL2722**	718	2344	469	1,71	-0,61
2452	SACOL2723	5426	15178	3036	1,48	-0,84
2453	SACOL2724	235	279	56	0,24	-2,08
2454	**SACOL2725**	292	278	56	-0,07	-2,39
2455	SACOL2727	1399	1070	214	-0,39	-2,71
2456	**SACOL2728**	512	370	74	-0,47	-2,79
2457	**SACOL2731**	74212	3113	623	-4,58	-6,90
2458	**SACOL2732**	381	390	78	0,03	-2,29
2459	SACOL2733	1366	986	197	-0,47	-2,79
2460	SACOL2734	1589	1382	276	-0,20	-2,52
2461	**SACOL2735**	15007	8617	1723	-0,80	-3,12
2462	**SACOL2736**	31912	14119	2824	-1,18	-3,50
2463	**SACOL2737**	22523	9257	1851	-1,28	-3,60
2464	**SACOL2738**	15207	5264	1053	-1,53	-3,85
2465	SACOL2739	15334	3698	740	-2,05	-4,37
2466	SACOL2740	64787	9235	1847	-2,81	-5,13
2467	**SACOLA0001**	21797	9147	1829	-1,25	-3,57
2468	**SACOLA0002**	60117	59035	11807	-0,03	-2,35
2469	**SACOLA0003**	14536	19472	3894	0,42	-1,90

a) *S. aureus* COL Locus. Written in bold indicates that the corresponding protein was identified.
b) *LOWESS normalized background subtracted signal intensities.*
c) *Scaled stationary phase signal intensities to account for decrease in total RNA during stationary phase.*
d) *Log_2 of stationary phase versus exponential growth ratios.*
e) *Log_2 of scaled stationary phase versus exponential growth ratios.*
