# Supplementary material for: A Proteomic View of an Important Human Pathogen – Towards the Quantification of the Entire Staphylococcus aureus Proteome
Source: PLoS One. 2009 Dec 4;4(12):e8176. doi: 10.1371/journal.pone.0008176 (PMC2781549; doi:10.1371/journal.pone.0008176)
Supplement: Table S2 — Identified proteins with corresponding quantitative value. (0.29 MB PDF) [file pone.0008176.s009.pdf]

Supplementary Table 2: Identified proteins with corresponding quantitative value

	gi accession	SACOL	gene	Cytosolic GeLC-MS/MS	Cytosolic 2D-PAGE	Membrane GeLC-MS/MS	Surface GeLC-MS/MS	Extracellular GeLC-MS/MS	Localization
1	57651109	SACOL0001	dnaA	-0.27		-0.19			Cytoplasmic
2	57651110	SACOL0002	dnaN	0.19	0.12	0.34	0.62		Cytoplasmic
3	57651111	SACOL0003	-	-0.25					Cytoplasmic
4	57651112	SACOL0004	-		-0.11	-0.22			Cytoplasmic
5	57651113	SACOL0005	gyrB	-0.61	-0.06	-0.74	-0.49		Cytoplasmic
6	57651114	SACOL0006	gyrA	-0.94	-0.44	-0.72	-0.53		Cytoplasmic
7	57651115	SACOL0007	-	n. qu.	n. qu.	n. qu.	n. qu.	n. qu.	Cytoplasmic
8	57651116	SACOL0008	hutH	4.40			on in stat		Cytoplasmic
9	57651117	SACOL0009	serS	-0.52	-0.44				Cytoplasmic
10	57651120	SACOL0012	-		1.14	0.69			Cytoplasmic
11	57651122	SACOL0014	-			0.44			Integral membrane
12	57651123	SACOL0015	rplI	-0.66	0.25			1.18	Cytoplasmic
13	57651124	SACOL0016	dnaB	0.69	0.33	0.33			Cytoplasmic
14	57651126	SACOL0018	purA	-0.46	-0.17		-0.69	-0.62	Cytoplasmic
15	57651127	SACOL0019	yycF	0.42	0.13		0.70		Cytoplasmic
16	57651128	SACOL0020	yycG			0.01			Integral membrane
17	57651129	SACOL0021	yycH			-0.17	-0.36		Cell wall associated
18	57651130	SACOL0022	yycI			-0.10			Cell wall associated
19	57651132	SACOL0024	-					2.23	Sortase substrate
20	57651134	SACOL0026	-	n. qu.	n. qu.	n. qu.	n. qu.	n. qu.	Cytoplasmic
21	57651138	SACOL0031	-	n. qu.	n. qu.	n. qu.	n. qu.	n. qu.	Cytoplasmic
22	57651139	SACOL0032	maoC	-0.85					Cytoplasmic
23	57651140	SACOL0033	mecA	0.28	-0.10	0.88		-0.27	Signal peptide
24	57652414	SACOL0045	-			-0.66			Cytoplasmic
25	57652419	SACOL0050	pls	0.41			1.23		Sortase substrate
26	57652420	SACOL0051	-	-0.43	0.41	-0.07			Cytoplasmic
27	57652423	SACOL0058	-	1.08		0.70			Cytoplasmic
28	57652429	SACOL0065	-	n. qu.	n. qu.	n. qu.	n. qu.	n. qu.	Cytoplasmic
29	57652431	SACOL0067	-	-1.79					Cytoplasmic
30	57652436	SACOL0072	-	0.71					Cytoplasmic
31	57652439	SACOL0075	-	n. qu.	n. qu.	n. qu.	n. qu.	n. qu.	Integral membrane
32	57652440	SACOL0076	-	0.92		0.82			Cytoplasmic
33	57652442	SACOL0078	plc					-1.45	Signal peptide
34	57652444	SACOL0080	-	n. qu.	n. qu.	n. qu.	n. qu.	n. qu.	Lipo
35	57652448	SACOL0084	-	n. qu.	n. qu.	n. qu.	n. qu.	n. qu.	Cytoplasmic
36	57652449	SACOL0085	-	n. qu.	n. qu.	n. qu.	n. qu.	n. qu.	Cytoplasmic
37	57652450	SACOL0086	-	n. qu.	n. qu.	n. qu.	n. qu.	n. qu.	Integral membrane
38	57652452	SACOL0088	-			-0.06			Integral membrane
39	57652457	SACOL0093	-			0.87			Integral membrane
40	57652458	SACOL0095	spa	n. qu.	n. qu.	n. qu.	n. qu.	n. qu.	Sortase substrate
41	57652459	SACOL0096	sarS	-0.69		-0.22			Cytoplasmic
42	57652462	SACOL0099	sirA			-1.50	-2.12		Lipo
43	57652465	SACOL0102	-	n. qu.	n. qu.	n. qu.	n. qu.	n. qu.	Cytoplasmic
44	57652467	SACOL0104	-	n. qu.	n. qu.	n. qu.	n. qu.	n. qu.	Cytoplasmic
45	57652473	SACOL0110	-			0.38			Integral membrane
46	57652474	SACOL0111	-	1.13	0.86	1.16	1.19		Cytoplasmic
47	57652481	SACOL0118	sodA1	0.28	0.45				Cytoplasmic
48	57652483	SACOL0120	-			0.88			Cytoplasmic
49	57651144	SACOL0123	deoC1	-0.53	0.06				Cytoplasmic
50	57651145	SACOL0124	deoB	0.08	-0.51			-2.08	Cytoplasmic
51	57651149	SACOL0128	-			0.56			Lipo
52	57651150	SACOL0129	-	n. qu.	n. qu.	n. qu.	n. qu.	n. qu.	Signal peptide
53	57651152	SACOL0135	-		0.26				Cytoplasmic
54	57651153	SACOL0136	cap5A			1.85			Integral membrane
55	57651154	SACOL0137	cap5B	1.34		1.58			Cytoplasmic
56	57651155	SACOL0138	cap5C	1.33		1.34			Cytoplasmic
57	57651156	SACOL0139	cap5D			0.78			Integral membrane
58	57651157	SACOL0140	cap5E	1.23					Cytoplasmic
59	57651158	SACOL0141	cap5F	2.31					Cytoplasmic
60	57651159	SACOL0142	cap5G	1.84	1.30	2.17	2.62		Cytoplasmic
61	57651160	SACOL0143	cap5H			1.53			Cytoplasmic
62	57651161	SACOL0144	cap5I	0.37	0.80	0.57			Cytoplasmic
63	57651162	SACOL0145	cap5J			on in stat			Integral membrane
64	57651164	SACOL0147	cap5L			0.89			Cytoplasmic
65	57651165	SACOL0148	cap5M			-0.03			Signal peptide
66	57651166	SACOL0149	cap5N			-0.31			Cytoplasmic
67	57651167	SACOL0150	cap5O	1.94	1.33	2.96			Cytoplasmic
68	57651168	SACOL0151	cap5P	0.69	0.19	0.92			Cytoplasmic
69	57651169	SACOL0152	-	-0.34					Cytoplasmic
70	57651171	SACOL0154	aldA1	4.18			on in stat		Cytoplasmic
71	57651172	SACOL0155	-	n. qu.	n. qu.	n. qu.	n. qu.	n. qu.	Integral membrane
72	57651173	SACOL0156	-	-0.15					Cytoplasmic

xyz - quantified with significant quantitative value, no change in protein amount
xyz - quantified with significant quantitative value, down-regulated/degraded in stat. phase
xyz - quantified with significant quantitative value, up-regulated in stat. phase
xyz - quantified in one biological replicate only (therefore not considered as reliable)
n.qu. - identified but not quantified

Continued Supplementary Table 2:

	gi accession	SACOL	gene	Cytosolic GeLC-MS/MS	Cytosolic 2D-PAGE	Membrane GeLC-MS/MS	Surface GeLC-MS/MS	Extracellular GeLC-MS/MS	Localization
73	57651174	SACOL0157	-	1.14	0.65				Cytoplasmic
74	57651177	SACOL0161	-	n. qu.	n. qu.	n. qu.	n. qu.	n. qu.	Cytoplasmic
75	57651178	SACOL0162	-		0.72				Cytoplasmic
76	57651180	SACOL0164	-	n. qu.	n. qu.	n. qu.	n. qu.	n. qu.	Cytoplasmic
77	57651182	SACOL0166	-			1.00			Integral membrane
78	57651187	SACOL0171	brnQ1			-0.61			Integral membrane
79	57651189	SACOL0173	ipdC	0.18	-0.19				Cytoplasmic
80	57651191	SACOL0175	-			-0.42			Integral membrane
81	57651192	SACOL0176	-	n. qu.	n. qu.	n. qu.	n. qu.	n. qu.	Cytoplasmic
82	57651194	SACOL0178	-			3.93			Integral membrane
83	57651196	SACOL0180	-	-0.14		0.12			Cytoplasmic
84	57651197	SACOL0181	-	n. qu.	n. qu.	n. qu.	n. qu.	n. qu.	Cytoplasmic
85	57651198	SACOL0182	-	1.13					Cytoplasmic
86	57651200	SACOL0184	-	n. qu.	n. qu.	n. qu.	n. qu.	n. qu.	Cytoplasmic
87	57651203	SACOL0187	-			0.31	0.42		Lipo
88	57651205	SACOL0189	-			-0.29			Cell wall associated
89	57651206	SACOL0190	acpD	1.52					Cytoplasmic
90	57651207	SACOL0191	-	n. qu.	n. qu.	n. qu.	n. qu.	n. qu.	Signal peptide
91	57651208	SACOL0192	-		2.92	on in stat			Cytoplasmic
92	57651209	SACOL0193	-			2.94			Lipo
93	57651210	SACOL0194	-	n. qu.	n. qu.	n. qu.	n. qu.	n. qu.	Integral membrane
94	57651211	SACOL0195	-	n. qu.	n. qu.	n. qu.	n. qu.	n. qu.	Integral membrane
95	57651212	SACOL0196	-	n. qu.	n. qu.	n. qu.	n. qu.	n. qu.	Cytoplasmic
96	57651213	SACOL0197	-	n. qu.	n. qu.	n. qu.	n. qu.	n. qu.	Cytoplasmic
97	57651214	SACOL0198	-	2.91					Cytoplasmic
98	57651215	SACOL0199	-			-2.23			Integral membrane
99	57651216	SACOL0200	-			on in stat			Integral membrane
100	57651217	SACOL0201	-	n. qu.	n. qu.	n. qu.	n. qu.	n. qu.	Cytoplasmic
101	57651218	SACOL0202	-	n. qu.	n. qu.	n. qu.	n. qu.	n. qu.	Integral membrane
102	57651219	SACOL0203	-	n. qu.	n. qu.	n. qu.	n. qu.	n. qu.	Lipo
103	57651220	SACOL0204	pflB	1.93					Cytoplasmic
104	57652487	SACOL0209	-					on in stat	Signal peptide
105	57652489	SACOL0211	-	n. qu.	n. qu.	n. qu.	n. qu.	n. qu.	Integral membrane
106	57652490	SACOL0212	-	n. qu.	n. qu.	n. qu.	n. qu.	n. qu.	Cytoplasmic
107	57652491	SACOL0213	-			on in stat			Cytoplasmic
108	57652492	SACOL0214	-	n. qu.	n. qu.	n. qu.	n. qu.	n. qu.	Cytoplasmic
109	57652494	SACOL0216	-			-2.02			Integral membrane
110	57652495	SACOL0217	-			-1.47	-1.89		Lipo
111	57652498	SACOL0220	-	-0.14					Cytoplasmic
112	57652499	SACOL0222	ldh1	-3.38	-1.06				Cytoplasmic
113	57652501	SACOL0224	-			on in stat			Integral membrane
114	57652511	SACOL0236	ispD	0.26					Cytoplasmic
115	57652512	SACOL0237	-			0.10			Cytoplasmic
116	57652513	SACOL0238	-	0.13	-1.01	0.52	0.73		Cytoplasmic
117	57652514	SACOL0239	-		1.52	-0.12			Cytoplasmic
118	57652515	SACOL0240	ispD	0.41	0.20	0.65	0.70		Cytoplasmic
119	57652516	SACOL0241	-	0.70	0.82	0.52	0.76		Cytoplasmic
120	57652517	SACOL0242	-	-0.95	2.27	-0.46			Cytoplasmic
121	57652518	SACOL0243	-	0.67	0.18	0.58	0.62		Cytoplasmic
122	57652520	SACOL0245	lytS	n. qu.	n. qu.	n. qu.	n. qu.	n. qu.	Integral membrane
123	57652524	SACOL0249	-		-2.24				Cytoplasmic
124	57652528	SACOL0253	rbsK	0.99					Cytoplasmic
125	57652529	SACOL0254	-	2.02					Cytoplasmic
126	57652530	SACOL0255	-			1.48			Integral membrane
127	57652531	SACOL0257	-	n. qu.	n. qu.	n. qu.	n. qu.	n. qu.	Cytoplasmic
128	57652537	SACOL0263	lytM				-2.64	-1.42	Signal peptide
129	57652544	SACOL0270	-				-0.84	0.34	Signal peptide
130	57652545	SACOL0271	-	-0.31			0.43	1.28	Cytoplasmic
131	57652546	SACOL0272	-			-2.10	-2.11	1.87	Integral membrane
132	57652547	SACOL0273	-			-0.21			Integral membrane
133	57652549	SACOL0275	-			-0.12	-0.39		Integral membrane
134	57652550	SACOL0276	yukA			-0.32			Integral membrane
135	57652551	SACOL0277	-	-0.93					Cytoplasmic
136	57652553	SACOL0279	-	0.07					Cytoplasmic
137	57652555	SACOL0281	-	n. qu.	n. qu.	n. qu.	n. qu.	n. qu.	Cytoplasmic
138	57652556	SACOL0282	-	0.33		-0.02			Cytoplasmic
139	57652558	SACOL0284	-			0.38			Integral membrane
140	57652565	SACOL0292	-	n. qu.	n. qu.	n. qu.	n. qu.	n. qu.	Cytoplasmic
141	57651226	SACOL0297	-	0.90					Cytoplasmic
142	57651227	SACOL0299	-		-1.34	-0.10			Lipo
143	57651230	SACOL0302	brnQ2			-1.75			Integral membrane
144	57651231	SACOL0303	-	n. qu.	n. qu.	n. qu.	n. qu.	n. qu.	Signal peptide

xyz - quantified with significant quantitative value, no change in protein amount
xyz - quantified with significant quantitative value, down-regulated/degraded in stat. phase
xyz - quantified with significant quantitative value, up-regulated in stat. phase
xyz - quantified in one biological replicate only (therefore not considered as reliable)
n.qu. - identified but not quantified

Continued Supplementary Table 2:

	gi accession	SACOL	gene	Cytosolic GeLC-MS/MS	Cytosolic 2D-PAGE	Membrane GeLC-MS/MS	Surface GeLC-MS/MS	Extracellular GeLC-MS/MS	Localization
145	57651232	SACOL0305	-			0.71			Integral membrane
146	57651233	SACOL0306	-			0.69			Cytoplasmic
147	57651234	SACOL0307	pfoR			on in stat			Integral membrane
148	57651238	SACOL0311	-			on in stat			Integral membrane
149	57651239	SACOL0312	nanA	4.81	2.47				Cytoplasmic
150	57651240	SACOL0313	-	n. qu.	n. qu.	n. qu.	n. qu.	n. qu.	Cytoplasmic
151	57651241	SACOL0314	-		-0.90				Cytoplasmic
152	57651242	SACOL0315	-	2.08					Cytoplasmic
153	57651243	SACOL0316	-			on in stat			Integral membrane
154	57651244	SACOL0317	-					0.78	Signal peptide
155	57651246	SACOL0319	-			-0.59			Cytoplasmic
156	57651248	SACOL0321	-	-0.24					Cytoplasmic
157	57651255	SACOL0328	-	n. qu.	n. qu.	n. qu.	n. qu.	n. qu.	Cytoplasmic
158	57651257	SACOL0330	-	n. qu.	n. qu.	n. qu.	n. qu.	n. qu.	Cytoplasmic
159	57651266	SACOL0339	ssb1					-1.92	Cytoplasmic
160	57651268	SACOL0341	-	n. qu.	n. qu.	n. qu.	n. qu.	n. qu.	Cytoplasmic
161	57651284	SACOL0357	dut					-0.14	Cytoplasmic
162	57651295	SACOL0368	-	n. qu.	n. qu.	n. qu.	n. qu.	n. qu.	Cytoplasmic
163	57651297	SACOL0370	-					-1.12	Cytoplasmic
164	57651302	SACOL0375	-	n. qu.	n. qu.	n. qu.	n. qu.	n. qu.	Cytoplasmic
165	57651303	SACOL0376	-			0.01		-0.85	Cytoplasmic
166	57651304	SACOL0377	-	n. qu.	n. qu.	n. qu.	n. qu.	n. qu.	Cytoplasmic
167	57652572	SACOL0383	-	n. qu.	n. qu.	n. qu.	n. qu.	n. qu.	Cytoplasmic
168	57652573	SACOL0384	-	n. qu.	n. qu.	n. qu.	n. qu.	n. qu.	Cytoplasmic
169	57652574	SACOL0385	-	n. qu.	n. qu.	n. qu.	n. qu.	n. qu.	Cytoplasmic
170	57652580	SACOL0391	-	0.01					Cytoplasmic
171	57652581	SACOL0392	-	n. qu.	n. qu.	n. qu.	n. qu.	n. qu.	Cytoplasmic
172	57652587	SACOL0398	-	n. qu.	n. qu.	n. qu.	n. qu.	n. qu.	Cytoplasmic
173	57652588	SACOL0399	-	0.03	-0.04	0.18			Cytoplasmic
174	57652589	SACOL0400	ulaA	n. qu.	n. qu.	n. qu.	n. qu.	n. qu.	Integral membrane
175	57652596	SACOL0407	glpT			on in stat			Integral membrane
176	57652598	SACOL0409	-	2.78	0.98	on in stat			Cytoplasmic
177	57652599	SACOL0410	-	2.71					Cytoplasmic
178	57652600	SACOL0411	-	n. qu.	n. qu.	n. qu.	n. qu.	n. qu.	Integral membrane
179	57652602	SACOL0413	-	2.93					Cytoplasmic
180	57652603	SACOL0414	-	n. qu.	n. qu.	n. qu.	n. qu.	n. qu.	Lipo
181	57652608	SACOL0419	-	n. qu.	n. qu.	n. qu.	n. qu.	n. qu.	Cytoplasmic
182	57652610	SACOL0421	-	n. qu.	n. qu.	n. qu.	n. qu.	n. qu.	Integral membrane
183	57652611	SACOL0422	-	-0.01		0.14			Cytoplasmic
184	57652612	SACOL0424	-			0.26			Integral membrane
185	57652614	SACOL0426	-	0.70	0.21		0.93		Cytoplasmic
186	57652615	SACOL0427	-	-0.83	-1.10				Cytoplasmic
187	57652616	SACOL0428	metE	-1.17	-0.55		-0.87		Cytoplasmic
188	57652617	SACOL0429	-		0.32				Cytoplasmic
189	57652618	SACOL0430	-		0.95				Cytoplasmic
190	57652619	SACOL0431	-		0.48				Cytoplasmic
191	57652621	SACOL0433	-			-0.37			Integral membrane
192	57652622	SACOL0434	-	n. qu.	n. qu.	n. qu.	n. qu.	n. qu.	Cytoplasmic
193	57652623	SACOL0435	-	-0.17	0.00	-0.36	-0.32		Cytoplasmic
194	57652625	SACOL0437	rpsF				-1.19		Cytoplasmic
195	57652626	SACOL0438	ssb2	-1.15	-1.50				Cytoplasmic
196	57652627	SACOL0439	rpsR	-2.04					Cytoplasmic
197	57652629	SACOL0442	-					2.75	Signal peptide
198	57652631	SACOL0444	-	1.66		1.32	0.76	1.37	Lipo
199	57652632	SACOL0445	-	n. qu.	n. qu.	n. qu.	n. qu.	n. qu.	Cytoplasmic
200	57652633	SACOL0446	-			0.91			Integral membrane
201	57652634	SACOL0447	-	-0.07					Cytoplasmic
202	57652636	SACOL0449	-			-0.43	-0.37		Lipo
203	57652638	SACOL0451	ahpF	0.65	0.01				Cytoplasmic
204	57652639	SACOL0452	ahpC	0.90	0.43	0.94		0.89	Cytoplasmic
205	57652640	SACOL0453	-	0.63					Cytoplasmic
206	57652641	SACOL0454	-			-0.06			Integral membrane
207	57652642	SACOL0455	-	-0.42	-0.56			0.77	Cytoplasmic
208	57652643	SACOL0456	-	1.22	-0.02	1.19			Cytoplasmic
209	57652644	SACOL0457	-	2.23				1.57	Cytoplasmic
210	57652645	SACOL0458	xpt	1.81	0.38				Cytoplasmic
211	57652646	SACOL0459	pbuX	n. qu.	n. qu.	n. qu.	n. qu.	n. qu.	Integral membrane
212	57652647	SACOL0460	guaB	0.90	-0.70		1.30		Cytoplasmic
213	57652648	SACOL0461	guaA	0.16	-0.05	0.05			Cytoplasmic
214	57652649	SACOL0462	-			0.15			Cytoplasmic
215	57652651	SACOL0464	-	n. qu.	n. qu.	n. qu.	n. qu.	n. qu.	Cytoplasmic
216	57651307	SACOL0466	-	n. qu.	n. qu.	n. qu.	n. qu.	n. qu.	Integral membrane

xyz - quantified with significant quantitative value, no change in protein amount
xyz - quantified with significant quantitative value, down-regulated/degraded in stat. phase
xyz - quantified with significant quantitative value, up-regulated in stat. phase
xyz - quantified in one biological replicate only (therefore not considered as reliable)
n.qu. - identified but not quantified

Continued Supplementary Table 2:

	gi accession	SACOL	gene	Cytosolic GeLC-MS/MS	Cytosolic 2D-PAGE	Membrane GeLC-MS/MS	Surface GeLC-MS/MS	Extracellular GeLC-MS/MS	Localization
217	57651308	SACOL0467	-	0.72	0.08				Cytoplasmic
218	57651309	SACOL0468	-					0.02	Signal peptide
219	57651317	SACOL0476	hsdM1		-0.68				Cytoplasmic
220	57651318	SACOL0477	-	-0.53					Cytoplasmic
221	57651319	SACOL0478	-					-1.93	Signal peptide
222	57651320	SACOL0479	-	n. qu.	n. qu.	n. qu.	n. qu.	n. qu.	Signal peptide
223	57651321	SACOL0480	-	n. qu.	n. qu.	n. qu.	n. qu.	n. qu.	Signal peptide
224	57651323	SACOL0482	-	n. qu.	n. qu.	n. qu.	n. qu.	n. qu.	Lipo
225	57651325	SACOL0484	-			0.46			Lipo
226	57651326	SACOL0485	-	n. qu.	n. qu.	n. qu.	n. qu.	n. qu.	Lipo
227	57651327	SACOL0486	-	1.36	3.08	1.14	0.68	2.62	Lipo
228	57651328	SACOL0487	-			-0.76		2.78	Cytoplasmic
229	57651329	SACOL0488	-	-0.50					Cytoplasmic
230	57651330	SACOL0489	-	n. qu.	n. qu.	n. qu.	n. qu.	n. qu.	Cytoplasmic
231	57651333	SACOL0494	nuoF	n. qu.	n. qu.	n. qu.	n. qu.	n. qu.	Integral membrane
232	57651334	SACOL0495	-			-1.10			Signal peptide
233	57651335	SACOL0496	-	0.10					Cytoplasmic
234	57651337	SACOL0498	-			-0.06			Integral membrane
235	57651338	SACOL0499	-	-1.02					Cytoplasmic
236	57651342	SACOL0503	-	-0.51	0.44				Cytoplasmic
237	57651343	SACOL0504	-			-0.45			Cytoplasmic
238	57651344	SACOL0505	-			-0.37			Integral membrane
239	57651345	SACOL0506	-			0.01	-0.23	1.66	Lipo
240	57651346	SACOL0507	-			-1.98		-1.80	Signal peptide
241	57651347	SACOL0508	-	n. qu.	n. qu.	n. qu.	n. qu.	n. qu.	Integral membrane
242	57651348	SACOL0509	-	0.32					Cytoplasmic
243	57651349	SACOL0510	-	n. qu.	n. qu.	n. qu.	n. qu.	n. qu.	Cytoplasmic
244	57651351	SACOL0512	-			-0.95			Integral membrane
245	57651353	SACOL0514	gltB	-1.92					Cytoplasmic
246	57651354	SACOL0515	gltD	-1.72	-0.57				Cytoplasmic
247	57651355	SACOL0516	-			2.97			Integral membrane
248	57651358	SACOL0519	-	-0.54					Cytoplasmic
249	57651359	SACOL0520	dnaX	0.49	-0.74				Cytoplasmic
250	57651360	SACOL0521	-	0.05					Cytoplasmic
251	57651361	SACOL0522	recR	-0.50					Cytoplasmic
252	57651362	SACOL0523	-	n. qu.	n. qu.	n. qu.	n. qu.	n. qu.	Cytoplasmic
253	57651363	SACOL0524	tmk	-0.08					Cytoplasmic
254	57651364	SACOL0525	-	1.02					Cytoplasmic
255	57651365	SACOL0526	-	-0.21		-0.37			Cytoplasmic
256	57651367	SACOL0528	-	-0.12					Cytoplasmic
257	57651368	SACOL0529	-			-0.86			Cytoplasmic
258	57651372	SACOL0533	metS	-0.27	-0.57	0.46			Cytoplasmic
259	57651373	SACOL0534	-	0.15	0.06				Cytoplasmic
260	57651375	SACOL0536	ksgA	-0.83	-0.56	-0.74			Cytoplasmic
261	57651377	SACOL0538	ispE			0.23			Cytoplasmic
262	57651378	SACOL0539	purR	-0.24	0.14	-0.30			Cell wall associated
263	57650037	SACOL0540	-	0.52					Cytoplasmic
264	57650038	SACOL0541	spoVG	0.42					Cytoplasmic
265	57650039	SACOL0542	-	n. qu.	n. qu.	n. qu.	n. qu.	n. qu.	Cytoplasmic
266	57650040	SACOL0543	glmU	-0.54					Cytoplasmic
267	57650041	SACOL0544	prsA	0.15	0.27	0.07	0.12		Cytoplasmic
268	57650042	SACOL0545	rplY	-1.40	-1.63	-1.18	-1.24	0.04	Cytoplasmic
269	57650043	SACOL0546	pth			-0.20			Cytoplasmic
270	57650044	SACOL0547	mfd			-0.29			Cytoplasmic
271	57650045	SACOL0548	-	n. qu.	n. qu.	n. qu.	n. qu.	n. qu.	Integral membrane
272	57650046	SACOL0549	-	-0.23	-1.21	-0.03			Cytoplasmic
273	57650047	SACOL0550	-	-0.30					Cytoplasmic
274	57650048	SACOL0551	-			-0.74			Cell wall associated
275	57651384	SACOL0552	-	-1.46	0.08			0.88	Cytoplasmic
276	57651385	SACOL0553	-			-0.81			Cytoplasmic
277	57651386	SACOL0554	hpt	-1.04	-0.81				Cytoplasmic
278	57651387	SACOL0555	-	0.18		-0.41	-0.80		Integral membrane
279	57651388	SACOL0556	-		-1.14		-1.23		Cytoplasmic
280	57651389	SACOL0557	cysK	0.56	0.64		0.86	0.51	Cytoplasmic
281	57651390	SACOL0558	folP	0.00		0.13			Cytoplasmic
282	57651391	SACOL0559	folB	-0.40					Cytoplasmic
283	57651392	SACOL0560	folK	-1.37					Cytoplasmic
284	57651394	SACOL0562	lysS	-0.63	-0.19	-0.57	-0.39		Cytoplasmic
285	57651395	SACOL0563	-			0.44			Cytoplasmic
286	57651396	SACOL0564	-	-0.06	-0.13	-0.82	-0.29	-1.38	Cytoplasmic
287	57651397	SACOL0565	-	-0.45	-0.41				Cytoplasmic
288	57651398	SACOL0566	nupC			0.70			Integral membrane

xyz - quantified with significant quantitative value, no change in protein amount
xyz - quantified with significant quantitative value, down-regulated/degraded in stat. phase
xyz - quantified with significant quantitative value, up-regulated in stat. phase
xyz - quantified in one biological replicate only (therefore not considered as reliable)
n.qu. - identified but not quantified

Continued Supplementary Table 2:

	gi accession	SACOL	gene	Cytosolic GeLC-MS/MS	Cytosolic 2D-PAGE	Membrane GeLC-MS/MS	Surface GeLC-MS/MS	Extracellular GeLC-MS/MS	Localization
289	57651399	SACOL0567	ctsR	n. qu.	n. qu.	n. qu.	n. qu.	n. qu.	Cytoplasmic
290	57651400	SACOL0568	-	0.38					Cytoplasmic
291	57651401	SACOL0569	-	0.17		0.25			Cytoplasmic
292	122063323	SACOL0570	clpC		-0.48				Cytoplasmic
293	57651402	SACOL0572	radA		1.18	0.70			Cytoplasmic
294	57651403	SACOL0573	-			-0.13			Integral membrane
295	57651404	SACOL0574	gltX	0.18	0.07	0.53		0.29	Cytoplasmic
296	57651405	SACOL0575	cysE	0.29	1.35	0.59			Cytoplasmic
297	57651406	SACOL0576	cysS	0.30	0.18	0.82			Cytoplasmic
298	57651407	SACOL0577	-	n. qu.	n. qu.	n. qu.	n. qu.	n. qu.	Cytoplasmic
299	57651408	SACOL0578	-	0.23	0.51	0.73	0.71		Cytoplasmic
300	57651409	SACOL0579	-	-0.90	-1.04				Cytoplasmic
301	57651411	SACOL0581	secE	n. qu.	n. qu.	n. qu.	n. qu.	n. qu.	Integral membrane
302	57651412	SACOL0582	nusG	-0.26		-0.37	-0.06	0.32	Cytoplasmic
303	57651413	SACOL0583	rplK	-1.95	-1.83	-1.52	-1.42		Cytoplasmic
304	57651414	SACOL0584	rplA	-2.40	-0.84	-0.89	-0.98	-1.73	Cytoplasmic
305	57651415	SACOL0585	rplJ	-1.76	-0.60	-1.63	-1.42	-1.80	Cytoplasmic
306	57651416	SACOL0586	rplL	-1.67	-2.18				Cytoplasmic
307	57651417	SACOL0587	-	-1.15	1.68				Cytoplasmic
308	57651418	SACOL0588	rpoB	-0.74	-0.82	-0.89	-0.80		Cytoplasmic
309	57651419	SACOL0589	rpoC	-0.62	-0.15	-0.68	-0.59		Cytoplasmic
310	57651421	SACOL0591	rpsL	-2.01		-1.89	-1.46		Cytoplasmic
311	57651422	SACOL0592	rpsG	-2.06	-2.24	-1.62	-1.28		Cytoplasmic
312	57651423	SACOL0593	fusA	-0.86	-0.67	-0.58	-0.46	-2.19	Cytoplasmic
313	57651424	SACOL0594	tuf	-0.81	-0.96	-0.55		-2.37	Cytoplasmic
314	57651425	SACOL0595	-	0.56		-0.55			Cytoplasmic
315	57651426	SACOL0596	-	1.79	0.88		0.82	1.15	Cytoplasmic
316	57651427	SACOL0597	-	0.24	0.30		0.88	0.42	Cytoplasmic
317	57651428	SACOL0598	-	n. qu.	n. qu.	n. qu.	n. qu.	n. qu.	Cytoplasmic
318	57651429	SACOL0599	-	1.91	1.90				Cytoplasmic
319	57651430	SACOL0600	ilvE	1.43	0.99				Cytoplasmic
320	57651432	SACOL0602	-		0.93				Cytoplasmic
321	57651433	SACOL0603	-	-1.42	-0.57	-1.13			Cytoplasmic
322	57651434	SACOL0604	-	-1.65					Cytoplasmic
323	57651435	SACOL0606	-	0.39		0.56			Cytoplasmic
324	57651436	SACOL0607	-	0.50	0.31	0.76			Cytoplasmic
325	57651437	SACOL0608	sdrC	n. qu.	n. qu.	n. qu.	n. qu.	n. qu.	Sortase substrate
326	57651438	SACOL0609	sdrD	n. qu.	n. qu.	n. qu.	n. qu.	n. qu.	Sortase substrate
327	57651439	SACOL0610	sdrE				-0.06		Sortase substrate
328	57651440	SACOL0611	-	0.83	1.41		0.59		Cytoplasmic
329	57651441	SACOL0612	-		0.27	0.66	0.76		Cytoplasmic
330	57651442	SACOL0613	-		-0.72				Cytoplasmic
331	57651443	SACOL0614	-	-1.48	-0.40				Cytoplasmic
332	57651444	SACOL0615	-	n. qu.	n. qu.	n. qu.	n. qu.	n. qu.	Cytoplasmic
333	57651445	SACOL0616	nagB	0.68					Cytoplasmic
334	57651446	SACOL0617	-	1.10	0.83		1.92		Cytoplasmic
335	57651447	SACOL0618	-	0.72	0.35				Cytoplasmic
336	57651448	SACOL0619	-	0.86					Cytoplasmic
337	57651449	SACOL0620	proP			-0.22			Integral membrane
338	57651450	SACOL0621	-			1.98			Cytoplasmic
339	57651451	SACOL0622	atoB	n. qu.	n. qu.	n. qu.	n. qu.	n. qu.	Cytoplasmic
340	57651455	SACOL0626	thiD1	0.67	0.03		0.82		Cytoplasmic
341	57651456	SACOL0627	ung	0.56	0.20				Cytoplasmic
342	57651459	SACOL0630	-			-0.99			Integral membrane
343	57651461	SACOL0632	-			-0.70			Integral membrane
344	57651462	SACOL0633	-		0.87		0.67	0.45	Cytoplasmic
345	57651463	SACOL0634	pta	-0.18	-0.10		0.45	-0.39	Cytoplasmic
346	57651465	SACOL0636	mvk	-0.61	0.80				Cytoplasmic
347	57651466	SACOL0637	mvaD	-0.67	-0.01				Cytoplasmic
348	57650049	SACOL0638	-		-0.99	-0.77			Cytoplasmic
349	57650054	SACOL0643	-			-0.63			Integral membrane
350	57650055	SACOL0644	-			-0.55			Integral membrane
351	57650066	SACOL0655	-	1.49					Cytoplasmic
352	57650067	SACOL0656	-	2.05	2.01	2.49			Cytoplasmic
353	57650068	SACOL0658	-	0.81	0.34	0.81			Cytoplasmic
354	57650069	SACOL0659	-	-0.37					Cytoplasmic
355	57650070	SACOL0660	-	0.40	-0.85				Cytoplasmic
356	57650072	SACOL0662	-	n. qu.	n. qu.	n. qu.	n. qu.	n. qu.	Cytoplasmic
357	57650073	SACOL0663	argS	-0.28	-0.70	0.83			Cytoplasmic
358	57650075	SACOL0665	-			0.66	0.20		Lipo
359	57650078	SACOL0668	-	0.81		0.70			Cytoplasmic
360	57650079	SACOL0669	-				1.43	2.09	Signal peptide

xyz - quantified with significant quantitative value, no change in protein amount
xyz - quantified with significant quantitative value, down-regulated/degraded in stat. phase
xyz - quantified with significant quantitative value, up-regulated in stat. phase
xyz - quantified in one biological replicate only (therefore not considered as reliable)
n.qu. - identified but not quantified

Continued Supplementary Table 2:

	gi accession	SACOL	gene	Cytosolic GeLC-MS/MS	Cytosolic 2D-PAGE	Membrane GeLC-MS/MS	Surface GeLC-MS/MS	Extracellular GeLC-MS/MS	Localization
361	57650080	SACOL0670	-			-2.05			Integral membrane
362	57650081	SACOL0671	-		0.43				Cytoplasmic
363	57650082	SACOL0672	sarA	0.14	1.11	-0.10	0.19		Cytoplasmic
364	57650086	SACOL0678	-			1.43			Cytoplasmic
365	57650087	SACOL0679	-			0.73			Integral membrane
366	57651467	SACOL0682	-	n. qu.	n. qu.	n. qu.	n. qu.	n. qu.	Integral membrane
367	57651468	SACOL0684	-	n. qu.	n. qu.	n. qu.	n. qu.	n. qu.	Integral membrane
368	57651471	SACOL0687	-			-0.35			Integral membrane
369	57651472	SACOL0688	-	n. qu.	n. qu.	n. qu.	n. qu.	n. qu.	Lipo
370	57651475	SACOL0691	sirR	0.32					Cytoplasmic
371	57651477	SACOL0693	tagA			-0.85			Cytoplasmic
372	57651478	SACOL0694	-	-0.86		-0.45			Cytoplasmic
373	57651479	SACOL0695	-			-0.64			Integral membrane
374	57651480	SACOL0696	tagB			0.09			Cytoplasmic
375	57651481	SACOL0697	tagX			0.17			Cytoplasmic
376	57651482	SACOL0698	tagD	0.15	0.08				Cytoplasmic
377	57651483	SACOL0699	pbp4			-1.89	-1.84		Integral membrane
378	57651484	SACOL0700	abcA			1.11			Integral membrane
379	57651485	SACOL0701	-			-0.19			Integral membrane
380	57651487	SACOL0703	-			1.72			Integral membrane
381	57651488	SACOL0704	-	-1.97		-2.51			Cytoplasmic
382	57651490	SACOL0706	-	n. qu.	n. qu.	n. qu.	n. qu.	n. qu.	Integral membrane
383	57651491	SACOL0707	-	3.35					Cytoplasmic
384	57651492	SACOL0708	-	n. qu.	n. qu.	n. qu.	n. qu.	n. qu.	Cytoplasmic
385	57651493	SACOL0709	-	3.31					Cytoplasmic
386	57651495	SACOL0711	-			0.32			Integral membrane
387	57651496	SACOL0712	-			-0.67	-1.01		Cell wall associated
388	57651498	SACOL0714	-	0.42	0.45				Cytoplasmic
389	57651499	SACOL0715	-	n. qu.	n. qu.	n. qu.	n. qu.	n. qu.	Cytoplasmic
390	57651500	SACOL0716	-	1.12		1.10			Cytoplasmic
391	57651501	SACOL0717	-			0.10			Integral membrane
392	57651502	SACOL0718	-		1.55	0.45			Cytoplasmic
393	57651503	SACOL0720	-			0.21			Integral membrane
394	57651504	SACOL0721	-	-0.02	-0.24	-0.33	0.22		Cytoplasmic
395	57651505	SACOL0722	-	n. qu.	n. qu.	n. qu.	n. qu.	n. qu.	Integral membrane
396	57651510	SACOL0727	-	-0.38	-0.21				Cytoplasmic
397	57651511	SACOL0728	-	-1.25					Cytoplasmic
398	57651512	SACOL0730	-			0.91			Cytoplasmic
399	57651513	SACOL0731	-	1.31	1.30	1.14			Cytoplasmic
400	57651514	SACOL0733	-	n. qu.	n. qu.	n. qu.	n. qu.	n. qu.	Integral membrane
401	57651516	SACOL0735	-			-0.80			Integral membrane
402	57651518	SACOL0737	-			-0.28			Lipo
403	57651519	SACOL0738	-	-0.52					Cytoplasmic
404	57651520	SACOL0739	-	0.03					Cytoplasmic
405	57651521	SACOL0740	-	0.23		0.13			Cytoplasmic
406	57651523	SACOL0742	-	-1.71		-1.57			Signal peptide
407	57651524	SACOL0743	uppP			-0.65			Integral membrane
408	57651525	SACOL0744	-			-0.39			Integral membrane
409	57651526	SACOL0745	-			-0.34			Integral membrane
410	57651527	SACOL0746	norR	-0.84	-0.27	-0.88	-0.62		Cytoplasmic
411	57651529	SACOL0748	-	1.15					Cytoplasmic
412	57651531	SACOL0750	-			1.96			Integral membrane
413	57651534	SACOL0753	-	0.72					Cytoplasmic
414	57651535	SACOL0754	norA			2.26			Integral membrane
415	57651536	SACOL0755	-					-0.46	Signal peptide
416	57651537	SACOL0756	-	n. qu.	n. qu.	n. qu.	n. qu.	n. qu.	Cytoplasmic
417	57651540	SACOL0761	nagA	-0.48					Cytoplasmic
418	57651541	SACOL0762	-	n. qu.	n. qu.	n. qu.	n. qu.	n. qu.	Integral membrane
419	57651542	SACOL0763	-	0.43					Cytoplasmic
420	57651543	SACOL0764	-			-0.21			Integral membrane
421	57651544	SACOL0765	saeS			-0.16			Integral membrane
422	57651545	SACOL0766	saeR		0.16				Cytoplasmic
423	57651547	SACOL0768	-	1.17		1.13	0.76	2.38	Lipo
424	57651551	SACOL0772	-	-1.49					Cytoplasmic
425	57651552	SACOL0773	pabA	-0.72	-0.81				Cytoplasmic
426	57651553	SACOL0776	-	-0.07	-0.18	-0.15	0.04		Cytoplasmic
427	57651554	SACOL0777	-	-0.23	1.19	-0.07	-0.06		Cytoplasmic
428	57651555	SACOL0778	-		0.85	-0.74	-0.16	-1.69	Integral membrane
429	57651556	SACOL0779	-			-0.87			Cytoplasmic
430	57651558	SACOL0781	-			0.04			Cytoplasmic
431	57651559	SACOL0783	opuBB			0.46	0.00		Integral membrane
432	57651560	SACOL0784	hisC	1.22	0.95	0.83			Cytoplasmic

xyz [yellow] - quantified with significant quantitative value, no change in protein amount
xyz [green] - quantified with significant quantitative value, down-regulated/degraded in stat. phase
xyz [red] - quantified with significant quantitative value, up-regulated in stat. phase
xyz - quantified in one biological replicate only (therefore not considered as reliable)
n.qu. - identified but not quantified

Continued Supplementary Table 2:

	gi accession	SACOL	gene	Cytosolic GeLC-MS/MS	Cytosolic 2D-PAGE	Membrane GeLC-MS/MS	Surface GeLC-MS/MS	Extracellular GeLC-MS/MS	Localization
433	57651561	SACOL0785	-	0.29	0.30				Cytoplasmic
434	57651563	SACOL0787	-	1.42					Cytoplasmic
435	57651564	SACOL0788	-			-0.86			Integral membrane
436	57651565	SACOL0789	-	-1.15	0.12				Cytoplasmic
437	57651568	SACOL0792	nrdE	-0.83	-0.26		-0.40		Cytoplasmic
438	57651569	SACOL0793	nrdF		-0.06	-0.11	-0.37		Integral membrane
439	57651574	SACOL0799	-			-1.49	-1.52		Lipo
440	57651575	SACOL0800	-				0.37		Cytoplasmic
441	57651576	SACOL0801	murB	-0.27	-0.06				Cytoplasmic
442	57651578	SACOL0803	-			-0.76	-1.02		Lipo
443	57651579	SACOL0804	-	n. qu.	n. qu.	n. qu.	n. qu.	n. qu.	Cytoplasmic
444	57651580	SACOL0805	-	0.61		0.84			Cytoplasmic
445	57651581	SACOL0806	pepT	0.40					Cytoplasmic
446	57651583	SACOL0808	-	n. qu.	n. qu.	n. qu.	n. qu.	n. qu.	Integral membrane
447	57651584	SACOL0809	-			-0.14			Integral membrane
448	57651586	SACOL0811	-	-0.33					Cytoplasmic
449	57651587	SACOL0812	-	-0.16					Cytoplasmic
450	57650091	SACOL0815	-	0.62	0.53		0.89	3.11	Cytoplasmic
451	57650092	SACOL0816	secA	0.24	-0.40	-0.09	0.23		Cytoplasmic
452	57650094	SACOL0818	prfB	n. qu.	n. qu.	n. qu.	n. qu.	n. qu.	Cytoplasmic
453	57650097	SACOL0821	-	1.72	0.47	1.74			Cytoplasmic
454	57650099	SACOL0823	uvrB	0.43		0.58			Cytoplasmic
455	57650100	SACOL0824	uvrA	-0.09	-1.08	-0.36			Cytoplasmic
456	57650101	SACOL0825	hprK	0.50	0.46	0.60			Cytoplasmic
457	57650102	SACOL0826	lgt			0.44			Integral membrane
458	57650104	SACOL0828	-			0.12			Cytoplasmic
459	57650105	SACOL0829	trxB	0.33	0.03		0.68	-0.16	Cytoplasmic
460	57650106	SACOL0830	-	0.33		-0.16			Cytoplasmic
461	57650107	SACOL0831	-	0.71	2.08	0.89			Cytoplasmic
462	57650108	SACOL0832	-	-0.51	1.25	-0.61			Cytoplasmic
463	57650109	SACOL0833	clpP	-0.21	0.94	0.25	0.28		Cytoplasmic
464	57650110	SACOL0834	-	0.90	1.75	1.13			Cytoplasmic
465	57650113	SACOL0837	gapR	-0.61	-0.53	-0.91			Cytoplasmic
466	57650114	SACOL0838	gapA1	-0.95	-0.75	-1.73		-1.51	Cytoplasmic
467	57650115	SACOL0839	pgk	-0.39	-0.37	-0.26		-1.10	Cytoplasmic
468	57650116	SACOL0840	tpiA	-0.47	-0.40		-0.13	-0.67	Cytoplasmic
469	57650117	SACOL0841	pgm	-0.96	-1.00				Cytoplasmic
470	57650118	SACOL0842	eno	0.09	-0.50		0.42		Cytoplasmic
471	57650119	SACOL0843	-			-0.05			Integral membrane
472	57650120	SACOL0844	secG			-0.10			Integral membrane
473	57650121	SACOL0845	est	-0.08	0.00				Cytoplasmic
474	57650122	SACOL0846	-	0.13		0.36			Cytoplasmic
475	57650123	SACOL0847	smpB	-0.64					Cytoplasmic
476	57650125	SACOL0849	-	0.77					Cytoplasmic
477	57650127	SACOL0851	-	1.62		1.37	0.93	3.94	Lipo
478	57650130	SACOL0855	-	-1.24					Cytoplasmic
479	57650131	SACOL0856	clfA				1.72	0.30	Sortase substrate
480	57650134	SACOL0859	-	n. qu.	n. qu.	n. qu.	n. qu.	n. qu.	Signal peptide
481	57650135	SACOL0860	nuc					1.24	Integral membrane
482	57650136	SACOL0861	-	n. qu.	n. qu.	n. qu.	n. qu.	n. qu.	Cytoplasmic
483	57650144	SACOL0870	-	n. qu.	n. qu.	n. qu.	n. qu.	n. qu.	Integral membrane
484	57650145	SACOL0871	-	1.55					Cytoplasmic
485	57650146	SACOL0872	-	3.19					Cytoplasmic
486	57650148	SACOL0874	-	-0.87			-0.21		Cytoplasmic
487	57650149	SACOL0875	-	0.57					Cytoplasmic
488	57650150	SACOL0876	-	1.26	2.18				Cytoplasmic
489	57650151	SACOL0877	gcvH	1.21					Cytoplasmic
490	57650152	SACOL0879	-	0.75	0.61				Cytoplasmic
491	57650154	SACOL0881	-	n. qu.	n. qu.	n. qu.	n. qu.	n. qu.	Cytoplasmic
492	57650155	SACOL0882	-		1.33	-0.30			Cytoplasmic
493	57650156	SACOL0883	-			0.05			Integral membrane
494	57650157	SACOL0884	-			0.47	-0.11		Lipo
495	57650159	SACOL0886	sek					-0.90	Signal peptide
496	57650160	SACOL0887	sei					-0.92	Signal peptide
497	57650161	SACOL0888	-			-0.66	-1.29		Lipo
498	57650163	SACOL0890	-	-0.98	-1.53				Cytoplasmic
499	57651597	SACOL0907	seb	3.58			5.23	2.82	Signal peptide
500	57651598	SACOL0908	-					-0.15	Signal peptide
501	57651602	SACOL0912	-	0.87					Cytoplasmic
502	57651604	SACOL0914	-	-0.42	-0.54	-0.27	-0.22		Cytoplasmic
503	57651605	SACOL0915	sufD	-0.41	0.24	0.20	0.03		Cytoplasmic
504	57651606	SACOL0916	-	-0.25	-0.31	-0.05			Cytoplasmic

xyz - quantified with significant quantitative value, no change in protein amount
xyz - quantified with significant quantitative value, down-regulated/degraded in stat. phase
xyz - quantified with significant quantitative value, up-regulated in stat. phase
xyz - quantified in one biological replicate only (therefore not considered as reliable)
n.qu. - identified but not quantified

Continued Supplementary Table 2:

	gi accession	SACOL	gene	Cytosolic GeLC-MS/MS	Cytosolic 2D-PAGE	Membrane GeLC-MS/MS	Surface GeLC-MS/MS	Extracellular GeLC-MS/MS	Localization
505	57651607	SACOL0917	-	-0.91			-0.59		Cytoplasmic
506	57651608	SACOL0918	sufB	-0.42	-2.19	0.09	-0.12		Cytoplasmic
507	57651611	SACOL0921	-			-1.29			Integral membrane
508	57651612	SACOL0922	-	n. qu.	n. qu.	n. qu.	n. qu.	n. qu.	Cytoplasmic
509	57651613	SACOL0924	-	n. qu.	n. qu.	n. qu.	n. qu.	n. qu.	Cytoplasmic
510	57651614	SACOL0925	-	n. qu.	n. qu.	n. qu.	n. qu.	n. qu.	Integral membrane
511	57651615	SACOL0926	-	n. qu.	n. qu.	n. qu.	n. qu.	n. qu.	Cytoplasmic
512	57651616	SACOL0927	lipA	-1.14	2.18	-2.22	-1.41		Cytoplasmic
513	57651617	SACOL0928	-	-1.04					Cytoplasmic
514	57651618	SACOL0929	-	n. qu.	n. qu.	n. qu.	n. qu.	n. qu.	Cytoplasmic
515	57651619	SACOL0930	-	-0.18		-0.07			Cytoplasmic
516	57651620	SACOL0931	-	-0.07	-0.20				Cytoplasmic
517	57651621	SACOL0932	-	0.23	0.59	0.35	0.80		Cytoplasmic
518	57651624	SACOL0935	dltA	-0.52	0.03	-0.42			Cytoplasmic
519	57651625	SACOL0936	dltB	n. qu.	n. qu.	n. qu.	n. qu.	n. qu.	Integral membrane
520	57651626	SACOL0937	dltC			-0.19			Cytoplasmic
521	57651627	SACOL0938	dltD			-0.78	-1.12		Cell wall associated
522	57651628	SACOL0939	-	-0.95					Cytoplasmic
523	57651629	SACOL0940	-	0.61					Cytoplasmic
524	57651631	SACOL0943	-	n. qu.	n. qu.	n. qu.	n. qu.	n. qu.	Cytoplasmic
525	57651632	SACOL0944	-	-0.42	1.68	-0.43	-0.25		Cytoplasmic
526	57651633	SACOL0945	-	0.61	0.21	0.56			Cytoplasmic
527	57651634	SACOL0946	-	n. qu.	n. qu.	n. qu.	n. qu.	n. qu.	Integral membrane
528	57651637	SACOL0949	mnhG			0.11			Integral membrane
529	57651639	SACOL0951	mnhE			0.17			Integral membrane
530	57651640	SACOL0952	mnhD			0.79			Integral membrane
531	57651641	SACOL0953	mnhC	n. qu.	n. qu.	n. qu.	n. qu.	n. qu.	Integral membrane
532	57651642	SACOL0954	mnhB	n. qu.	n. qu.	n. qu.	n. qu.	n. qu.	Integral membrane
533	57651643	SACOL0955	mnhA			0.60			Integral membrane
534	57651644	SACOL0956	kapB	0.69					Cytoplasmic
535	57651645	SACOL0957	-	-0.28	-0.16				Cytoplasmic
536	57651646	SACOL0958	-	n. qu.	n. qu.	n. qu.	n. qu.	n. qu.	Cytoplasmic
537	57651647	SACOL0959	-	1.94	-0.83				Cytoplasmic
538	57651648	SACOL0960	rocD	3.27	0.71	on in stat	3.33		Cytoplasmic
539	57651649	SACOL0961	gluD	1.83	-1.69	1.93	2.42		Cytoplasmic
540	57651650	SACOL0962	-				3.40	3.05	Signal peptide
541	57651654	SACOL0966	pgi	0.30	0.09			0.31	Cytoplasmic
542	57651656	SACOL0968	spsA			0.79			Cell wall associated
543	57651657	SACOL0969	spsB	0.05		-0.04	-0.85		Integral membrane
544	57651658	SACOL0970	rexB	1.01		1.30			Cytoplasmic
545	57651659	SACOL0971	rexA	0.94		1.27			Cytoplasmic
546	57651660	SACOL0973	-	0.50	-0.37		0.79	-1.80	Cytoplasmic
547	57651662	SACOL0975	-	0.44	0.03				Cytoplasmic
548	57651663	SACOL0976	-	-0.70	-1.39	-0.83	-0.79		Cytoplasmic
549	57651666	SACOL0979	clpB	0.28	0.41	0.21			Cytoplasmic
550	57650172	SACOL0984	-	-0.45					Cytoplasmic
551	57650173	SACOL0985	-				1.03		Signal peptide
552	57650175	SACOL0987	fabH	-0.87	-0.99	-0.89	-0.94		Cytoplasmic
553	57650176	SACOL0988	fabF	-0.28	-0.58		-0.13	-2.05	Cytoplasmic
554	57650179	SACOL0991	oppB			2.67			Integral membrane
555	57650180	SACOL0992	oppC			2.01	2.07		Integral membrane
556	57650181	SACOL0993	oppD	1.31	2.76	1.99	2.48		Cytoplasmic
557	57650182	SACOL0994	oppF	1.54	2.01	2.07	2.43		Cytoplasmic
558	57650183	SACOL0995	-	2.32		2.14	1.87		Lipo
559	57650184	SACOL0996	-			1.07			Lipo
560	57650189	SACOL1001	trpS	0.17	1.24				Cytoplasmic
561	57650190	SACOL1002	spxA	0.70					Signal peptide
562	57650191	SACOL1003	-	-3.48	-3.55				Cytoplasmic
563	57650193	SACOL1005	pepF	0.79	-0.57	1.27			Cytoplasmic
564	57650194	SACOL1006	-			-1.44			Cytoplasmic
565	57650196	SACOL1008	-	-0.37	-0.55				Cytoplasmic
566	57650197	SACOL1009	-	0.11					Cytoplasmic
567	57650198	SACOL1010	relA1	0.50	0.47	0.74			Cytoplasmic
568	57650199	SACOL1011	ppnK	0.48	2.06	0.31			Cytoplasmic
569	57650201	SACOL1013	mgtE			0.10			Integral membrane
570	57650202	SACOL1016	fabI	0.16	0.24	0.03	0.27		Cytoplasmic
571	57650203	SACOL1017	-			0.44			Integral membrane
572	57650204	SACOL1018	-			0.88			Integral membrane
573	57650206	SACOL1020	-	2.44			2.74	on in stat	Cytoplasmic
574	57650207	SACOL1021	-	n. qu.	n. qu.	n. qu.	n. qu.	n. qu.	Integral membrane
575	57650208	SACOL1022	ypfP			off in stat			Cytoplasmic
576	57650209	SACOL1023	murE	-0.49	-0.32				Cytoplasmic

xyz - quantified with significant quantitative value, no change in protein amount
xyz - quantified with significant quantitative value, down-regulated/degraded in stat. phase
xyz - quantified with significant quantitative value, up-regulated in stat. phase
xyz - quantified in one biological replicate only (therefore not considered as reliable)
n.qu. - identified but not quantified

Continued Supplementary Table 2:

	gi accession	SACOL	gene	Cytosolic GeLC-MS/MS	Cytosolic 2D-PAGE	Membrane GeLC-MS/MS	Surface GeLC-MS/MS	Extracellular GeLC-MS/MS	Localization
577	57650211	SACOL1025	prfC	-0.43		-0.62			Cytoplasmic
578	57650212	SACOL1026	-			-0.02			Integral membrane
579	57650214	SACOL1028	htrA			-0.49			Cell wall associated
580	57650215	SACOL1030	-	n. qu.	n. qu.	n. qu.	n. qu.	n. qu.	Integral membrane
581	57650216	SACOL1031	-	0.55		0.70			Integral membrane
582	57650219	SACOL1034	-	0.54	0.48	0.41			Cytoplasmic
583	57650221	SACOL1036	-	n. qu.	n. qu.	n. qu.	n. qu.	n. qu.	Integral membrane
584	57650227	SACOL1042	-	0.21					Cytoplasmic
585	57650228	SACOL1043	-			-0.59			Cytoplasmic
586	57650230	SACOL1045	-			0.49			Lipo
587	57650232	SACOL1047	-	n. qu.	n. qu.	n. qu.	n. qu.	n. qu.	Integral membrane
588	57650233	SACOL1048	-	n. qu.	n. qu.	n. qu.	n. qu.	n. qu.	Cytoplasmic
589	57650234	SACOL1049	menA			-0.83			Integral membrane
590	57650236	SACOL1051	-	n. qu.	n. qu.	n. qu.	n. qu.	n. qu.	Cytoplasmic
591	57650237	SACOL1052	menD	-0.65	0.17				Cytoplasmic
592	57650238	SACOL1053	-	n. qu.	n. qu.	n. qu.	n. qu.	n. qu.	Cytoplasmic
593	57650239	SACOL1054	menB	-0.04	-0.19	-0.66	-0.48		Cytoplasmic
594	57650241	SACOL1056	sspB1					-0.12	Signal peptide
595	57650243	SACOL1058	-	0.52		-0.33	0.68		Cytoplasmic
596	57650244	SACOL1059	-	n. qu.	n. qu.	n. qu.	n. qu.	n. qu.	Integral membrane
597	57650246	SACOL1062	atl		0.73	0.31	1.74	-0.15	Cell wall associated
598	57650247	SACOL1063	-	n. qu.	n. qu.	n. qu.	n. qu.	n. qu.	Cytoplasmic
599	57650249	SACOL1065	-				-1.22	0.43	Signal peptide
600	57650250	SACOL1066	-			-0.94	-1.84		Cell wall associated
601	57650251	SACOL1067	qoxD	n. qu.	n. qu.	n. qu.	n. qu.	n. qu.	Integral membrane
602	57650252	SACOL1068	qoxC			0.52			Integral membrane
603	57650253	SACOL1069	qoxA			0.33	-0.96		Integral membrane
604	57650254	SACOL1070	qoxB			0.03	-0.46		Lipo
605	57651672	SACOL1072	folD	2.07	1.45	2.53	2.42		Cytoplasmic
606	57651673	SACOL1073	purE	3.51	4.26				Cytoplasmic
607	57651674	SACOL1074	purK	3.17	1.21				Cytoplasmic
608	57651675	SACOL1075	purC	3.44	1.52	on in stat			Cytoplasmic
609	57651676	SACOL1076	purS	3.26					Cytoplasmic
610	57651677	SACOL1077	purQ	2.79	1.13				Cytoplasmic
611	57651678	SACOL1078	purL	3.02	-0.67	3.01			Cytoplasmic
612	57651679	SACOL1079	purF	3.33	0.73				Cytoplasmic
613	57651680	SACOL1080	purM	2.91		3.04	2.72		Cytoplasmic
614	57651681	SACOL1081	purN	3.05	0.34				Cytoplasmic
615	57651682	SACOL1082	purH	2.35	1.72	on in stat	2.27		Cytoplasmic
616	57651683	SACOL1083	purD	2.60	1.94				Cytoplasmic
617	57651685	SACOL1085	-			0.88			Cytoplasmic
618	57651686	SACOL1086	-			1.41			Integral membrane
619	57651688	SACOL1088	-			0.26			Integral membrane
620	57651689	SACOL1089	-		0.92	0.90			Cytoplasmic
621	57651690	SACOL1090	-	-1.12		-1.02	-0.78		Cytoplasmic
622	57651691	SACOL1091	ptsH	0.12				-1.50	Cytoplasmic
623	57651692	SACOL1092	ptsI	0.05	0.05	0.50	0.36		Cytoplasmic
624	57651694	SACOL1094	cydA			-1.56			Integral membrane
625	57651696	SACOL1096	-	0.38	-0.36				Cytoplasmic
626	57651698	SACOL1098	-	0.17	1.61	0.15	0.15		Cytoplasmic
627	57651699	SACOL1099	-	n. qu.	n. qu.	n. qu.	n. qu.	n. qu.	Cytoplasmic
628	57651700	SACOL1100	def	-0.57	-0.42		-0.37		Cytoplasmic
629	57651701	SACOL1101	-			-0.42	-0.18	-1.20	Lipo
630	57651702	SACOL1102	pdhA	-0.31	0.84	-0.06	0.08	-2.26	Cytoplasmic
631	57651703	SACOL1103	pdhB	-0.20	-0.42	-0.26	0.09	-2.30	Cytoplasmic
632	57651704	SACOL1104	pdhC	-0.24	1.95	-0.33	0.50	-1.84	Cytoplasmic
633	57651705	SACOL1105	pdhD	0.02	0.67	-0.33	-0.41	-0.85	Cytoplasmic
634	57651706	SACOL1106	-	n. qu.	n. qu.	n. qu.	n. qu.	n. qu.	Cytoplasmic
635	57651707	SACOL1107	-	-1.28					Cytoplasmic
636	57651708	SACOL1108	-		-1.02	-1.21			Cytoplasmic
637	57651709	SACOL1109	-	n. qu.	n. qu.	n. qu.	n. qu.	n. qu.	Integral membrane
638	57651710	SACOL1110	-	n. qu.	n. qu.	n. qu.	n. qu.	n. qu.	Integral membrane
639	57651711	SACOL1111	-			-0.60			Cell wall associated
640	57651712	SACOL1112	-			-0.14			Integral membrane
641	57651713	SACOL1113	-			2.07			Integral membrane
642	57651714	SACOL1114	-			-0.10			Integral membrane
643	57651715	SACOL1115	-	0.45	1.57	0.48	1.02		Cytoplasmic
644	57651716	SACOL1116	-	0.33	-0.38	0.66			Cytoplasmic
645	57651718	SACOL1118	typA	-1.90	-1.88	-2.38	-1.73		Cytoplasmic
646	57651720	SACOL1120	-	0.68	0.43				Cytoplasmic
647	57651722	SACOL1122	-			-0.20			Integral membrane
648	57651723	SACOL1123	pyc	0.47	0.56	0.58	1.36	-1.17	Cytoplasmic

xyz - quantified with significant quantitative value, no change in protein amount
xyz - quantified with significant quantitative value, down-regulated/degraded in stat. phase
xyz - quantified with significant quantitative value, up-regulated in stat. phase
xyz - quantified in one biological replicate only (therefore not considered as reliable)
n.qu. - identified but not quantified

Continued Supplementary Table 2:

	gi accession	SACOL	gene	Cytosolic GeLC-MS/MS	Cytosolic 2D-PAGE	Membrane GeLC-MS/MS	Surface GeLC-MS/MS	Extracellular GeLC-MS/MS	Localization
649	57651724	SACOL1124	ctaA			0.46			Integral membrane
650	57651725	SACOL1125	ctaB			off in stat			Integral membrane
651	57651726	SACOL1126	-	n. qu.	n. qu.	n. qu.	n. qu.	n. qu.	Integral membrane
652	57651728	SACOL1128	-			1.12			Cytoplasmic
653	57651730	SACOL1130	-		0.09	-0.17			Signal peptide
654	57651733	SACOL1133	-			0.00			Cytoplasmic
655	57651734	SACOL1134	kdtB	-0.35	-0.27				Cytoplasmic
656	57651735	SACOL1135	-			0.15			Cytoplasmic
657	57651736	SACOL1136	-	-1.07					Cytoplasmic
658	57651739	SACOL1140	isdA					-0.10	Sortase substrate
659	57651741	SACOL1142	isdD	n. qu.	n. qu.	n. qu.	n. qu.	n. qu.	Integral membrane
660	57651746	SACOL1147	-	-0.09		0.35			Cytoplasmic
661	57651747	SACOL1148	pheS	-0.66	0.10				Cytoplasmic
662	57651748	SACOL1149	pheT	-0.71	-0.34	-0.66			Cytoplasmic
663	57651749	SACOL1150	rnhC			-0.74			Cytoplasmic
664	57651751	SACOL1152	-	n. qu.	n. qu.	n. qu.	n. qu.	n. qu.	Integral membrane
665	57651752	SACOL1153	-	-0.29					Cytoplasmic
666	57651753	SACOL1154	-		-0.73	-0.02			Cytoplasmic
667	57651754	SACOL1155	trxA	0.88			1.22		Cytoplasmic
668	57650256	SACOL1157	uvrC			1.28			Cytoplasmic
669	57650257	SACOL1158	sdhC			3.04			Integral membrane
670	57650258	SACOL1159	sdhA	2.86	2.26	3.46	2.81		Signal peptide
671	57650259	SACOL1160	sdhB	2.91	1.85	3.36	3.76		Cytoplasmic
672	57650260	SACOL1161	murI	1.41	2.53	0.90			Cytoplasmic
673	57650261	SACOL1162	-	0.94					Cytoplasmic
674	57650262	SACOL1163	-	0.77	-1.50				Cytoplasmic
675	57650263	SACOL1164	-	n. qu.	n. qu.	n. qu.	n. qu.	n. qu.	Cell wall associated
676	57650265	SACOL1166	-	n. qu.	n. qu.	n. qu.	n. qu.	n. qu.	Cytoplasmic
677	57650267	SACOL1168	efb					0.98	Cell wall associated
678	57650282	SACOL1183	-			0.35			Integral membrane
679	57650287	SACOL1188	-	-0.37					Cytoplasmic
680	57650288	SACOL1189	-		1.16				Cytoplasmic
681	57650289	SACOL1190	-	-1.09					Cytoplasmic
682	57650290	SACOL1191	mraZ		-2.63				Cytoplasmic
683	57650291	SACOL1192	-	-0.88	0.19	-0.45			Cytoplasmic
684	57650292	SACOL1193	-	n. qu.	n. qu.	n. qu.	n. qu.	n. qu.	Integral membrane
685	57650293	SACOL1194	pbp1			-0.50	-0.76		Signal peptide
686	57650294	SACOL1195	mraY			-0.39			Integral membrane
687	57650295	SACOL1196	murD	-0.67		0.23			Cytoplasmic
688	57650296	SACOL1197	divIB			-0.84	-1.42		Integral membrane
689	57651755	SACOL1198	ftsA	0.23	-0.81	0.23	0.50		Cytoplasmic
690	57651756	SACOL1199	ftsZ	0.25	0.48	0.19	0.34	-1.13	Cytoplasmic
691	57651757	SACOL1200	-	0.42	-0.16				Cytoplasmic
692	57651758	SACOL1201	-	0.72	0.41				Cytoplasmic
693	57651759	SACOL1202	ylmF	0.62			0.52		Cytoplasmic
694	57651760	SACOL1203	ylmG			-0.28			Integral membrane
695	57651761	SACOL1204	ylmH			-0.78			Cytoplasmic
696	57651762	SACOL1205	-	0.60			0.88		Cytoplasmic
697	57651763	SACOL1206	ileS	-0.78	-0.98	-0.80	-0.89		Cytoplasmic
698	57651764	SACOL1207	-	-0.76					Cytoplasmic
699	57651765	SACOL1208	lspA	n. qu.	n. qu.	n. qu.	n. qu.	n. qu.	Integral membrane
700	57651766	SACOL1209	-	-0.57	0.70				Cytoplasmic
701	57651767	SACOL1210	pyrR	0.88	-0.34	1.18			Cytoplasmic
702	57651769	SACOL1212	pyrB	1.51					Cytoplasmic
703	57651770	SACOL1213	pyrC	0.90	-0.58				Cytoplasmic
704	57651771	SACOL1214	carA		1.51	0.33			Cytoplasmic
705	57651772	SACOL1215	carB	1.07	1.05	0.95	0.98		Cytoplasmic
706	57651773	SACOL1216	pyrF	0.66					Cytoplasmic
707	57651774	SACOL1217	pyrE	0.16	0.50				Cytoplasmic
708	57651776	SACOL1219	-	n. qu.	n. qu.	n. qu.	n. qu.	n. qu.	Cytoplasmic
709	57651777	SACOL1220	-	-0.33	0.99	-0.44			Cytoplasmic
710	57651778	SACOL1221	gmk	-0.51	-1.21				Cytoplasmic
711	57651779	SACOL1222	rpoZ	-0.59					Cytoplasmic
712	57651780	SACOL1223	coaBC	0.33	0.34	-0.35	0.27		Cytoplasmic
713	57651781	SACOL1224	priA		0.61	0.19			Cytoplasmic
714	57651782	SACOL1225	-			0.33	0.24	2.86	Lipo
715	57651784	SACOL1227	def2	-0.69					Cytoplasmic
716	57651785	SACOL1228	fmt	0.09					Cytoplasmic
717	57651786	SACOL1229	sun	-0.59	-0.64	-0.35			Cytoplasmic
718	57651787	SACOL1230	-			-3.07			Cytoplasmic
719	57651788	SACOL1231	-	-0.16					Cytoplasmic
720	57651789	SACOL1234	-		1.17	0.12			Cytoplasmic

xyz - quantified with significant quantitative value, no change in protein amount
xyz - quantified with significant quantitative value, down-regulated/degraded in stat. phase
xyz - quantified with significant quantitative value, up-regulated in stat. phase
xyz - quantified in one biological replicate only (therefore not considered as reliable)
n.qu. - identified but not quantified

Continued Supplementary Table 2:

	gi accession	SACOL	gene	Cytosolic GeLC-MS/MS	Cytosolic 2D-PAGE	Membrane GeLC-MS/MS	Surface GeLC-MS/MS	Extracellular GeLC-MS/MS	Localization
721	57651791	SACOL1236	-	0.49	0.59	0.53			Cytoplasmic
722	57651792	SACOL1238	rpmB	-2.36		-2.03	-1.61		Cytoplasmic
723	57651793	SACOL1239	-	0.42		0.35			Cytoplasmic
724	57651794	SACOL1240	-	-0.41		-0.36	-0.09		Cytoplasmic
725	57651795	SACOL1241	recG			0.11			Cytoplasmic
726	57651796	SACOL1242	-			-0.08			Cytoplasmic
727	57651797	SACOL1243	plsX	-0.40	-0.55	-0.35	-0.37		Cytoplasmic
728	57651798	SACOL1244	fabD	-0.71					Cytoplasmic
729	57651799	SACOL1245	fabG1	-0.68	-0.64	-0.33	-0.37		Cytoplasmic
730	57651801	SACOL1247	acpP	-0.49			-0.92	-2.83	Cytoplasmic
731	57651802	SACOL1248	rnc			-1.15			Cytoplasmic
732	57651803	SACOL1250	-	-0.53		-0.54			Cytoplasmic
733	57651804	SACOL1251	-	-0.38		-0.34	-0.04		Cytoplasmic
734	57651805	SACOL1252	-	-1.15					Cytoplasmic
735	57651806	SACOL1253	ffh	-0.47		-0.36	-0.46		Cytoplasmic
736	57651807	SACOL1254	rpsP	-2.29		-1.37			Signal peptide
737	57651808	SACOL1255	rimM	-1.34					Cytoplasmic
738	57651810	SACOL1257	rplS	-1.79		-1.90	-1.41		Cytoplasmic
739	57651811	SACOL1259	-			0.88			Integral membrane
740	57651812	SACOL1260	-		0.59	0.18			Cytoplasmic
741	57651813	SACOL1261	rnhB	n. qu.	n. qu.	n. qu.	n. qu.	n. qu.	Cytoplasmic
742	57651814	SACOL1262	sucC	2.33	1.53	3.24	3.56		Cytoplasmic
743	57651815	SACOL1263	sucD	2.37	1.47	2.34		0.82	Cytoplasmic
744	57651819	SACOL1267	topA	-0.52		-0.35			Cytoplasmic
745	57651820	SACOL1268	gid			off in stat			Cytoplasmic
746	57651821	SACOL1269	xerC	n. qu.	n. qu.	n. qu.	n. qu.	n. qu.	Cytoplasmic
747	57651822	SACOL1270	hslV	0.09	-0.10				Cytoplasmic
748	57651823	SACOL1271	hslU	-0.29	-0.33	-0.12	-0.12		Cytoplasmic
749	57651824	SACOL1272	codY	0.72	0.57	0.55	0.69		Cytoplasmic
750	57651825	SACOL1274	rpsB	-1.74	-1.60	-1.50	-1.34		Cytoplasmic
751	57651827	SACOL1276	tsf		-0.81	-0.25	-0.19	0.41	Cytoplasmic
752	57651828	SACOL1277	pyrH		0.26		-0.28		Cytoplasmic
753	57651829	SACOL1278	frr	-0.46	0.33				Cytoplasmic
754	57651830	SACOL1279	uppS			-0.60			Cytoplasmic
755	57651831	SACOL1280	cdsA	n. qu.	n. qu.	n. qu.	n. qu.	n. qu.	Integral membrane
756	57651832	SACOL1281	-			-0.83	-1.70		Integral membrane
757	57651833	SACOL1282	proS	-0.31	-0.88	0.23			Cytoplasmic
758	57651834	SACOL1283	-	-0.78		-0.77			Cytoplasmic
759	57651835	SACOL1284	-	0.04					Cytoplasmic
760	57651836	SACOL1285	-	-0.02	-0.18	0.08	0.24		Cytoplasmic
761	57651837	SACOL1286	-	0.08					Cytoplasmic
762	57651838	SACOL1287	-	-0.76					Cytoplasmic
763	57651839	SACOL1288	infB	0.18	1.41	0.54	0.38		Cytoplasmic
764	57651840	SACOL1289	rbfA	-0.24	-1.54				Cytoplasmic
765	57651841	SACOL1290	truB	0.43	1.05				Cytoplasmic
766	57651842	SACOL1291	ribF	0.37	0.19	-0.38			Cytoplasmic
767	57651843	SACOL1292	rpsO	-2.05	-5.16				Cytoplasmic
768	57651844	SACOL1293	pnp	-1.04	-0.51	-0.19	-0.69		Cytoplasmic
769	57651845	SACOL1294	-	0.27	1.04	0.15	0.46		Cytoplasmic
770	57651846	SACOL1295	-			-1.17			Integral membrane
771	57651847	SACOL1296	-	-0.15	-0.76				Cytoplasmic
772	57651848	SACOL1297	-	-0.98		-0.32			Cytoplasmic
773	57651849	SACOL1298	-	-1.02		-0.81			Cytoplasmic
774	57651850	SACOL1299	-	-1.18					Cytoplasmic
775	57651851	SACOL1300	-	1.92		1.33			Integral membrane
776	57651852	SACOL1301	-			-0.31			Integral membrane
777	57651853	SACOL1302	pgsA			0.22			Integral membrane
778	57651854	SACOL1303	-	0.65	1.30	1.12			Cytoplasmic
779	57651855	SACOL1304	recA	0.49	0.57	0.59	0.64	-0.54	Cytoplasmic
780	57651856	SACOL1305	-			-0.62			Signal peptide
781	57651858	SACOL1307	-	-0.18	-0.47	-0.23			Cytoplasmic
782	57651859	SACOL1308	-		-0.23				Cytoplasmic
783	57651860	SACOL1309	-	-1.34					Cytoplasmic
784	57651861	SACOL1310	-	0.46					Cytoplasmic
785	57651862	SACOL1312	miaB	-2.34		-2.70			Cytoplasmic
786	57651864	SACOL1314	-			0.70			Integral membrane
787	57651865	SACOL1315	hexA	-0.82	0.18	-0.85			Cytoplasmic
788	57651866	SACOL1316	hexB	-0.21	0.36	-0.02			Cytoplasmic
789	57651867	SACOL1317	glpP	-0.89		-0.66			Cytoplasmic
790	57651868	SACOL1319	glpF			2.84			Integral membrane
791	57651869	SACOL1320	glpK	2.22	2.28	2.39	2.52		Cytoplasmic
792	57651870	SACOL1321	glpD		0.18	0.09	0.34		Cytoplasmic

xyz - quantified with significant quantitative value, no change in protein amount
xyz - quantified with significant quantitative value, down-regulated/degraded in stat. phase
xyz - quantified with significant quantitative value, up-regulated in stat. phase
xyz - quantified in one biological replicate only (therefore not considered as reliable)
n.qu. - identified but not quantified

Continued Supplementary Table 2:

	gi accession	SACOL	gene	Cytosolic GeLC-MS/MS	Cytosolic 2D-PAGE	Membrane GeLC-MS/MS	Surface GeLC-MS/MS	Extracellular GeLC-MS/MS	Localization
793	57651871	SACOL1322	-		0.88	0.19			Cytoplasmic
794	57651872	SACOL1323	miaA		1.30				Cytoplasmic
795	57651874	SACOL1325	gpxA1	0.54	0.81				Cytoplasmic
796	57650297	SACOL1326	-			0.52			Cytoplasmic
797	57650298	SACOL1327	-			-0.79			Cytoplasmic
798	57650299	SACOL1328	glnR	n. qu.	n. qu.	n. qu.	n. qu.	n. qu.	Cytoplasmic
799	57650300	SACOL1329	femC	-0.62	-0.75	-0.82	-0.24	-0.67	Cytoplasmic
800	57650318	SACOL1349	-	n. qu.	n. qu.	n. qu.	n. qu.	n. qu.	Cytoplasmic
801	57650320	SACOL1351	cls1			1.21			Integral membrane
802	57650324	SACOL1355	-	n. qu.	n. qu.	n. qu.	n. qu.	n. qu.	Cytoplasmic
803	57650326	SACOL1357	-			-0.73	-1.51		Integral membrane
804	57650327	SACOL1358	-			0.67			Signal peptide
805	57650329	SACOL1360	-		0.12	1.31			Cytoplasmic
806	57650331	SACOL1362	hom	0.42	0.13	-0.14	0.18		Cytoplasmic
807	57650332	SACOL1363	thrC	0.68	0.38	0.34	0.26		Cytoplasmic
808	57650333	SACOL1364	thrB		0.40				Cytoplasmic
809	57650334	SACOL1365	-	0.25	0.11	0.10	0.63		Cytoplasmic
810	57650335	SACOL1366	-	0.27					Cytoplasmic
811	57650336	SACOL1367	-			-0.91			Integral membrane
812	57650337	SACOL1368	kataA	1.31	1.41		0.75	0.17	Cytoplasmic
813	57650340	SACOL1371	guaC	-0.66	-0.62				Cytoplasmic
814	57650342	SACOL1373	-			off in stat		2.49	Cytoplasmic
815	57650343	SACOL1374	lexA	-0.07	-0.17				Cytoplasmic
816	57650345	SACOL1376	-	-0.39					Cytoplasmic
817	57650346	SACOL1377	tkt	0.14	0.24	0.24	-0.05		Cytoplasmic
818	57650347	SACOL1378	-			-1.20			Signal peptide
819	57650349	SACOL1380	-	n. qu.	n. qu.	n. qu.	n. qu.	n. qu.	Integral membrane
820	57650350	SACOL1381	sbcD	-0.58					Cytoplasmic
821	57650351	SACOL1382	sbcC	-0.42		-0.03			Cytoplasmic
822	57650352	SACOL1383	mscL			0.08			Integral membrane
823	57650353	SACOL1384	opuD1			0.24	-1.12		Integral membrane
824	57650354	SACOL1385	acnA	1.35	1.49	1.66	1.15	-0.72	Cytoplasmic
825	57650355	SACOL1386	-	0.10	-0.20				Cytoplasmic
826	57650356	SACOL1387	-	0.81					Cytoplasmic
827	57650357	SACOL1388	-	n. qu.	n. qu.	n. qu.	n. qu.	n. qu.	Integral membrane
828	57650358	SACOL1389	parE		1.74	-0.41			Cytoplasmic
829	57650359	SACOL1390	parC	-0.68	0.96	-0.84			Cytoplasmic
830	57650360	SACOL1392	-			-1.15			Integral membrane
831	57650361	SACOL1393	-	1.51		1.57			Cytoplasmic
832	57650363	SACOL1395	-			0.35			Integral membrane
833	57650364	SACOL1396	fmtC			-0.03			Integral membrane
834	57650365	SACOL1397	msrA	1.06					Cytoplasmic
835	57650366	SACOL1398	-			-1.31	-1.71		Signal peptide
836	57650367	SACOL1399	dmpI	1.38					Cytoplasmic
837	57650369	SACOL1401	tyrA			-0.80			Signal peptide
838	57650370	SACOL1402	-	1.15					Cytoplasmic
839	57650371	SACOL1403	trpE	-0.50		-0.82	-0.31		Cytoplasmic
840	57650372	SACOL1404	trpG	-0.30		-0.63			Cytoplasmic
841	57650373	SACOL1405	trpD	-0.18		-0.29	0.14		Cytoplasmic
842	57650374	SACOL1406	trpC	-0.34		-2.05			Cytoplasmic
843	57650375	SACOL1407	trpF	-0.87	0.89				Signal peptide
844	57650376	SACOL1408	trpB			-2.73	-1.38		Cytoplasmic
845	57650377	SACOL1409	trpA	-0.23		-0.63	-0.15		Cytoplasmic
846	57651875	SACOL1410	femA	-0.47		0.01	0.13		Cytoplasmic
847	57651876	SACOL1411	-	0.31	0.52	0.51	0.83		Cytoplasmic
848	57651878	SACOL1413	-	n. qu.	n. qu.	n. qu.	n. qu.	n. qu.	Cytoplasmic
849	57651882	SACOL1417	-	n. qu.	n. qu.	n. qu.	n. qu.	n. qu.	Integral membrane
850	57651884	SACOL1419	-	0.30	-0.27	0.72			Cytoplasmic
851	57651885	SACOL1420	-			0.94			Cytoplasmic
852	57651889	SACOL1424	-			1.98			Lipo
853	57651891	SACOL1426	-	-0.35		-0.06			Cytoplasmic
854	57651892	SACOL1427	-	-0.77	-0.38	-0.56	-0.38		Cytoplasmic
855	57651893	SACOL1428	lysC			-0.87			Cytoplasmic
856	57651894	SACOL1429	asd	0.42	1.08		0.59		Cytoplasmic
857	57651895	SACOL1430	dapA	0.50	0.44				Cytoplasmic
858	57651896	SACOL1431	dapB	0.84	0.22				Cytoplasmic
859	57651897	SACOL1432	dapD	0.42	-0.06				Cytoplasmic
860	57651898	SACOL1433	-	-1.08	-0.83				Cytoplasmic
861	57651899	SACOL1434	-	0.46	0.39				Cytoplasmic
862	57651900	SACOL1435	lysA	0.36	0.36		0.53		Cytoplasmic
863	57651902	SACOL1437	-	-0.82				-3.63	Cytoplasmic
864	57651904	SACOL1439	-	1.11					Cytoplasmic

xyz - quantified with significant quantitative value, no change in protein amount
xyz - quantified with significant quantitative value, down-regulated/degraded in stat. phase
xyz - quantified with significant quantitative value, up-regulated in stat. phase
xyz - quantified in one biological replicate only (therefore not considered as reliable)
n.qu. - identified but not quantified

Continued Supplementary Table 2:

	gi accession	SACOL	gene	Cytosolic GeLC-MS/MS	Cytosolic 2D-PAGE	Membrane GeLC-MS/MS	Surface GeLC-MS/MS	Extracellular GeLC-MS/MS	Localization
865	57651905	SACOL1440	-			-0.67			Integral membrane
866	57651906	SACOL1441	-	-0.35	-0.45	-0.63			Cytoplasmic
867	57651908	SACOL1443	brnQ3	n. qu.	n. qu.	n. qu.	n. qu.	n. qu.	Integral membrane
868	57651909	SACOL1444	-			0.02			Cytoplasmic
869	57651910	SACOL1445	-	-0.48	-0.60	-0.60	-0.18		Cytoplasmic
870	57651912	SACOL1447	-	0.46	-7.21	0.76	1.07		Cytoplasmic
871	57651913	SACOL1448	sucB	2.62	2.13	3.04	3.25		Cytoplasmic
872	57651914	SACOL1449	sucA	2.65	2.13	2.13	2.75		Cytoplasmic
873	57651915	SACOL1450	arlS			0.68			Integral membrane
874	57651916	SACOL1451	arlR	0.72					Cytoplasmic
875	57651918	SACOL1453	murG		1.56	-0.12			Cytoplasmic
876	57650378	SACOL1455	-			-0.05	0.01		Integral membrane
877	57650380	SACOL1457	-		0.15			0.62	Cytoplasmic
878	57650381	SACOL1460	-	0.94	1.14	1.52	1.48		Cytoplasmic
879	57650382	SACOL1461	folA	1.46	1.24				Cytoplasmic
880	57650383	SACOL1462	thyA	0.59	0.79				Cytoplasmic
881	57650384	SACOL1464	-	-0.26			0.45		Cytoplasmic
882	57650385	SACOL1465	-			-0.99			Cytoplasmic
883	57650386	SACOL1466	-	0.40					Cytoplasmic
884	57650388	SACOL1468	-	n. qu.	n. qu.	n. qu.	n. qu.	n. qu.	Integral membrane
885	57650390	SACOL1471	-	-0.03					Cytoplasmic
886	57651379	SACOL1472	ebh	n. qu.	n. qu.	n. qu.	n. qu.	n. qu.	Cell wall associated
887	57650395	SACOL1479	-			-2.94			Cytoplasmic
888	57650396	SACOL1480	-	0.01	0.48	0.33			Cytoplasmic
889	57650397	SACOL1481	-			-1.95			Integral membrane
890	57650398	SACOL1482	-	n. qu.	n. qu.	n. qu.	n. qu.	n. qu.	Signal peptide
891	57650399	SACOL1483	-	-0.66	-0.11				Cytoplasmic
892	57650400	SACOL1484	-	0.14		-0.26	0.34		Cytoplasmic
893	57650405	SACOL1490	pbp2	-0.45		-0.40	-0.45		Signal peptide
894	57650406	SACOL1491	-	-0.74					Cytoplasmic
895	57650407	SACOL1492	nth		0.65				Cytoplasmic
896	57650408	SACOL1493	-	n. qu.	n. qu.	n. qu.	n. qu.	n. qu.	Cytoplasmic
897	57650409	SACOL1494	asnS	0.19		-0.50	0.12		Cytoplasmic
898	57650410	SACOL1495	-			-0.30			Cytoplasmic
899	57650411	SACOL1496	birA	n. qu.	n. qu.	n. qu.	n. qu.	n. qu.	Cytoplasmic
900	57650412	SACOL1497	papS	-0.86		-0.01			Cytoplasmic
901	57650413	SACOL1498	-	-0.41		-0.06			Cytoplasmic
902	57650414	SACOL1499	-	-1.24			-1.66		Cytoplasmic
903	57650415	SACOL1500	-			-0.24			Integral membrane
904	57650418	SACOL1503	-	-0.25					Cytoplasmic
905	57650419	SACOL1504	aroA	-0.21		1.02			Cytoplasmic
906	57650420	SACOL1505	aroB	-0.18	0.21				Cytoplasmic
907	57650421	SACOL1506	aroC	-0.01	0.96				Cytoplasmic
908	57650424	SACOL1509	-	2.34	1.15				Cytoplasmic
909	57650425	SACOL1510	-			-0.09			Cytoplasmic
910	57650426	SACOL1511	-	-1.98		-1.67			Cytoplasmic
911	57650427	SACOL1512	-	-0.41		-0.36			Cytoplasmic
912	57650428	SACOL1513	hup	0.13	0.56				Cytoplasmic
913	57650429	SACOL1514	gpsA	0.41	0.46	0.87	0.96		Cell wall associated
914	57650430	SACOL1515	-		0.36	0.14	0.39		Cytoplasmic
915	57650431	SACOL1516	rpsA	0.06	2.41	0.55	0.50	0.16	Cytoplasmic
916	57650432	SACOL1518	cmk	-0.59					Cytoplasmic
917	57650433	SACOL1519	ansA	-0.07	0.29	0.14			Cytoplasmic
918	57650434	SACOL1520	-	-0.29	-0.21				Cytoplasmic
919	57650436	SACOL1522	-			-0.10	-0.20	3.87	Cell wall associated
920	57650441	SACOL1528	-			0.32			Lipo
921	57650443	SACOL1530	-		-4.37				Cytoplasmic
922	57650444	SACOL1531	-			-0.42			Lipo
923	57650445	SACOL1532	-			0.09			Cytoplasmic
924	57650446	SACOL1533	-		-4.37	0.80			Cytoplasmic
925	57650447	SACOL1534	srrB			0.91			Integral membrane
926	57650448	SACOL1535	srrA	0.99	0.78	0.71			Cytoplasmic
927	57650449	SACOL1536	rluB	-0.61	0.98				Cytoplasmic
928	57650450	SACOL1537	scpB	-0.62					Cytoplasmic
929	57650451	SACOL1538	scpA			-1.01			Cytoplasmic
930	57650453	SACOL1540	xerD	0.06		0.30			Cytoplasmic
931	57650454	SACOL1541	-	0.25	-0.11				Cytoplasmic
932	57650455	SACOL1542	-	n. qu.	n. qu.	n. qu.	n. qu.	n. qu.	Cytoplasmic
933	57650456	SACOL1543	-	1.12	0.71	1.43	1.76		Cytoplasmic
934	57650459	SACOL1546	proC	2.07	0.87				Cytoplasmic
935	57650460	SACOL1548	-	0.38					Cytoplasmic
936	57650461	SACOL1549	zwf	-0.03	0.25	-0.01			Cytoplasmic

xyz - quantified with significant quantitative value, no change in protein amount
xyz - quantified with significant quantitative value, down-regulated/degraded in stat. phase
xyz - quantified with significant quantitative value, up-regulated in stat. phase
xyz - quantified in one biological replicate only (therefore not considered as reliable)
n.qu. - identified but not quantified

Continued Supplementary Table 2:

	gi accession	SACOL	gene	Cytosolic GeLC-MS/MS	Cytosolic 2D-PAGE	Membrane GeLC-MS/MS	Surface GeLC-MS/MS	Extracellular GeLC-MS/MS	Localization
937	57650462	SACOL1550	-	-0.21		-0.57			Cytoplasmic
938	57650463	SACOL1551	malA	2.09					Cytoplasmic
939	57650464	SACOL1552	malR			1.66			Cytoplasmic
940	57650465	SACOL1553	-	-0.11					Cytoplasmic
941	57650466	SACOL1554	gnd	-0.21	-0.08	-0.10	-0.02		Cytoplasmic
942	57650467	SACOL1555	-	0.96	0.39	1.14			Cytoplasmic
943	57650470	SACOL1558	-	0.04			0.50		Cytoplasmic
944	57650472	SACOL1560	-	-0.68	-1.15	-0.87			Cytoplasmic
945	57650473	SACOL1561	-	-0.72	-1.02	-0.77	-0.45		Cytoplasmic
946	57650474	SACOL1562	-	-1.13	-0.55	-0.73	-0.91		Cytoplasmic
947	57650475	SACOL1563	lpdA	-0.68					Cytoplasmic
948	57650476	SACOL1564	recN	-0.01	-0.61	0.48			Cytoplasmic
949	57650477	SACOL1565	argR	0.16	-0.45	-0.10			Cytoplasmic
950	57650478	SACOL1566	ispA	-0.63	0.30	-0.35			Cytoplasmic
951	57650480	SACOL1568	xseA	-0.26	1.38	0.25			Cytoplasmic
952	57650481	SACOL1569	nusB	-0.64					Cytoplasmic
953	57650482	SACOL1570	-	0.58	-0.40				Cytoplasmic
954	57650483	SACOL1571	accC	-0.30	-0.03	-0.45	-0.13		Cytoplasmic
955	57650484	SACOL1572	accB	-0.51			-1.80		Cytoplasmic
956	57650485	SACOL1574	-			-0.02			Lipo
957	57650486	SACOL1575	-			1.96			Integral membrane
958	57650493	SACOL1582	-					on in stat	Signal peptide
959	57651923	SACOL1587	efp	-0.03					Cytoplasmic
960	57651924	SACOL1588	-	0.52	-0.08	0.44			Cytoplasmic
961	57651925	SACOL1589	-			-0.14	-1.09		Lipo
962	57651927	SACOL1591	-	-0.27	-0.17	0.32	0.22		Cytoplasmic
963	57651928	SACOL1592	-	-0.60		-0.97			Integral membrane
964	57651929	SACOL1593	-	1.93	1.66	1.68	2.59		Cytoplasmic
965	57651930	SACOL1594	-	1.82	1.04	1.46	2.54		Cytoplasmic
966	57651931	SACOL1595	gcvT	1.82			2.59		Cytoplasmic
967	57651938	SACOL1602	-	-0.61					Cytoplasmic
968	57651939	SACOL1603	-	0.03					Cytoplasmic
969	57651940	SACOL1604	glk	-0.17	-0.12	-0.39	-0.05		Cytoplasmic
970	57651942	SACOL1606	-			-0.65			Integral membrane
971	57651943	SACOL1607	-	0.30					Cytoplasmic
972	57651945	SACOL1609	pbp3			-0.34	-0.50	-2.01	Integral membrane
973	57651946	SACOL1610	sodA2	0.92	0.67		1.20	0.49	Cytoplasmic
974	57651947	SACOL1611	-	-0.68	-1.06				Cytoplasmic
975	57651948	SACOL1612	-			-1.16			Integral membrane
976	57651949	SACOL1613	-			-1.19			Cytoplasmic
977	57651950	SACOL1614	nfo	-0.56	-0.79		-0.38		Cytoplasmic
978	57651951	SACOL1615	-	-0.23		0.02			Cytoplasmic
979	57651952	SACOL1616	-	0.82					Cytoplasmic
980	57651953	SACOL1617	-	-1.24					Cytoplasmic
981	57651954	SACOL1618	rpoD	-0.52	-0.59	-0.64			Cytoplasmic
982	57651955	SACOL1619	dnaG	-0.38					Cytoplasmic
983	57651956	SACOL1620	-		0.29	0.61	0.79		Cytoplasmic
984	57651957	SACOL1621	-		4.07	1.15			Cytoplasmic
985	57651958	SACOL1622	glyS	-0.31	-0.42	-0.52			Cytoplasmic
986	57651960	SACOL1624	era	-0.32	0.01	0.18			Cytoplasmic
987	57651963	SACOL1627	-	0.45	-4.15				Cytoplasmic
988	57651964	SACOL1628	-	0.54	1.79				Cytoplasmic
989	57651965	SACOL1629	-			-0.13			Signal peptide
990	57651966	SACOL1630	-	-0.05	-0.91	0.35	0.35	-0.53	Integral membrane
991	57651967	SACOL1631	-			0.31			Integral membrane
992	57651968	SACOL1632	rpsU	-3.93					Cytoplasmic
993	57651969	SACOL1633	-			off in stat			Cytoplasmic
994	57651970	SACOL1634	-	-0.45					Cytoplasmic
995	57651971	SACOL1635	prmA	-1.50		-1.54			Cytoplasmic
996	57651972	SACOL1636	dnaJ	-0.57		-1.05			Cytoplasmic
997	57651973	SACOL1637	dnaK	-0.67	-0.87	-0.67	-0.36	-0.96	Cytoplasmic
998	57651974	SACOL1638	grpE	-0.94	-0.99				Cytoplasmic
999	57651977	SACOL1641	lepA	-0.44		-0.57	-0.54		Cytoplasmic
1000	57651978	SACOL1642	rpsT	-2.57					Cytoplasmic
1001	57651979	SACOL1643	-			0.02			Cytoplasmic
1002	57651981	SACOL1645	-	-0.13					Cytoplasmic
1003	57651983	SACOL1647	-	-1.55					Cytoplasmic
1004	57651984	SACOL1648	-	-1.70					Cytoplasmic
1005	57651985	SACOL1649	-	0.43	-0.33				Cytoplasmic
1006	57651986	SACOL1650	nadD	-1.20					Cytoplasmic
1007	57651987	SACOL1651	-	-0.69					Cytoplasmic
1008	57651988	SACOL1652	aroE	0.04	-0.34				Cytoplasmic

xyz - quantified with significant quantitative value, no change in protein amount
xyz - quantified with significant quantitative value, down-regulated/degraded in stat. phase
xyz - quantified with significant quantitative value, up-regulated in stat. phase
xyz - quantified in one biological replicate only (therefore not considered as reliable)
n.qu. - identified but not quantified

Continued Supplementary Table 2:

	gi accession	SACOL	gene	Cytosolic GeLC-MS/MS	Cytosolic 2D-PAGE	Membrane GeLC-MS/MS	Surface GeLC-MS/MS	Extracellular GeLC-MS/MS	Localization
1009	57651989	SACOL1653	-		1.47	-0.11			Cytoplasmic
1010	57651990	SACOL1654	-			-4.34			Cytoplasmic
1011	57651991	SACOL1655	mtn	0.12	0.03				Cytoplasmic
1012	57651994	SACOL1658	-			1.21			Cytoplasmic
1013	57651995	SACOL1659	-			2.18			Integral membrane
1014	57651996	SACOL1660	-	1.32					Cytoplasmic
1015	57651997	SACOL1661	-		1.58	1.31			Cytoplasmic
1016	57651998	SACOL1662	-	1.48			0.75		Cytoplasmic
1017	57651999	SACOL1663	-	1.35		1.46			Cytoplasmic
1018	57652000	SACOL1664	-	0.99					Cytoplasmic
1019	57652001	SACOL1665	greA	-0.90	-1.08			-0.70	Cytoplasmic
1020	57652002	SACOL1666	udk	-1.44	-1.28	-0.75	-1.04		Cytoplasmic
1021	57650494	SACOL1667	-	-0.45					Cytoplasmic
1022	57650495	SACOL1668	-	-2.92		-2.62			Cytoplasmic
1023	57650496	SACOL1669	-	-1.26	-1.59	-1.22			Cytoplasmic
1024	57650497	SACOL1670	-	-0.38					Cytoplasmic
1025	57650498	SACOL1671	-	-1.35					Cytoplasmic
1026	57650499	SACOL1672	-	-0.02					Cytoplasmic
1027	57650500	SACOL1673	alaS	-0.59	-0.02	-0.60	-0.33		Cytoplasmic
1028	57650501	SACOL1674	-	-0.38		0.04			Cytoplasmic
1029	57650502	SACOL1675	-	-0.97					Cytoplasmic
1030	57650503	SACOL1676	trmU	-0.96	-0.41				Cytoplasmic
1031	57650504	SACOL1677	-		-0.36				Cytoplasmic
1032	57650505	SACOL1678	-			1.41			Cytoplasmic
1033	57650508	SACOL1681	-	0.46	0.28	0.52	0.71		Cytoplasmic
1034	57650509	SACOL1682	-			0.26			Cytoplasmic
1035	57650510	SACOL1683	-	-0.95	1.51	-0.84			Cytoplasmic
1036	57650511	SACOL1685	aspS	-1.07	-0.94	-1.13	-1.83		Cytoplasmic
1037	57650512	SACOL1686	hisS	-0.73		-0.99	-0.63		Cytoplasmic
1038	57650513	SACOL1687	-			-0.96			Cell wall associated
1039	57650514	SACOL1688	-	0.46	0.25				Cytoplasmic
1040	57650515	SACOL1689	relA2	0.88		1.10			Cytoplasmic
1041	57650516	SACOL1690	apt	-1.23					Cytoplasmic
1042	57650517	SACOL1691	recJ	-1.39		-0.99			Cytoplasmic
1043	57650518	SACOL1692	-			-0.41	-0.84		Integral membrane
1044	57650519	SACOL1693	yajC			-0.10			Integral membrane
1045	57650520	SACOL1694	tgt	-1.09	-1.23	-1.06			Cytoplasmic
1046	57650521	SACOL1695	queA	-1.54	-0.92				Cytoplasmic
1047	57650522	SACOL1696	ruvB	-0.54		-0.69			Cytoplasmic
1048	57650523	SACOL1697	ruvA	-0.70	-0.72	-0.39			Cytoplasmic
1049	57650524	SACOL1698	-			-0.16			Cytoplasmic
1050	57650525	SACOL1699	-	-0.11		0.03			Cytoplasmic
1051	57650526	SACOL1700	rpmA	-2.05					Cytoplasmic
1052	57650528	SACOL1702	rplU	-2.04		-1.89	-1.61	-1.05	Cytoplasmic
1053	57650530	SACOL1704	mreC	0.02		-0.12	-0.11	3.09	Signal peptide
1054	57650531	SACOL1705	-			-0.16			Signal peptide
1055	57650535	SACOL1709	folC	-1.39		-1.71			Cytoplasmic
1056	57650536	SACOL1710	valS	-0.79	0.41	-0.94	-0.54		Cytoplasmic
1057	57650539	SACOL1714	hemL1	-0.26	-0.23	-0.30	-0.52		Cytoplasmic
1058	57650540	SACOL1715	hemB	-0.06	-0.26				Cytoplasmic
1059	57650541	SACOL1716	hemD	-0.37					Cytoplasmic
1060	57650542	SACOL1717	hemC	0.04					Cytoplasmic
1061	57650545	SACOL1720	-		0.98				Cytoplasmic
1062	57650546	SACOL1721	clpX	-0.88		-0.67	-0.48		Cytoplasmic
1063	57650547	SACOL1722	tig	-0.57	-0.24		-0.54	0.07	Cytoplasmic
1064	57650548	SACOL1723	-	1.04		0.79			Cytoplasmic
1065	57650549	SACOL1724	-	-0.18	-0.18				Cytoplasmic
1066	57650550	SACOL1725	rplT	-1.90		-1.86			Cell wall associated
1067	57650551	SACOL1726	rpmI	-1.81					Cytoplasmic
1068	57650552	SACOL1727	infC	-1.63	-2.35		-0.92		Cytoplasmic
1069	57650553	SACOL1728	-			-0.33			Integral membrane
1070	57650554	SACOL1729	thrS	-0.13	-0.36	-0.81	-0.44		Cytoplasmic
1071	57650555	SACOL1731	dnaI	0.60	1.83	0.54			Cytoplasmic
1072	57650556	SACOL1732	-			0.12			Cytoplasmic
1073	57650557	SACOL1733	-	0.94					Cytoplasmic
1074	57650558	SACOL1734	gapA2	3.46	1.94		on in stat		Signal peptide
1075	57650559	SACOL1735	coaE		-0.10				Cytoplasmic
1076	57650560	SACOL1736	fpg	-0.05					Cytoplasmic
1077	57650561	SACOL1737	polA	0.48	-0.02	0.96	1.06		Cytoplasmic
1078	57650563	SACOL1739	-			-0.08			Integral membrane
1079	57650564	SACOL1740	phoP	1.15	1.10	1.04			Cytoplasmic
1080	57650565	SACOL1741	icd	2.38	1.83	2.87	2.49		Cytoplasmic

xyz - quantified with significant quantitative value, no change in protein amount
xyz - quantified with significant quantitative value, down-regulated/degraded in stat. phase
xyz - quantified with significant quantitative value, up-regulated in stat. phase
xyz - quantified in one biological replicate only (therefore not considered as reliable)
n.qu. - identified but not quantified

Continued Supplementary Table 2:

	gi accession	SACOL	gene	Cytosolic GeLC-MS/MS	Cytosolic 2D-PAGE	Membrane GeLC-MS/MS	Surface GeLC-MS/MS	Extracellular GeLC-MS/MS	Localization
1081	57650566	SACOL1742	gltA	2.78	3.01	2.51	3.42	2.26	Cytoplasmic
1082	57650567	SACOL1743	-	n. qu.	n. qu.	n. qu.	n. qu.	n. qu.	Integral membrane
1083	57650568	SACOL1745	pyk	-0.47	0.00	0.09	0.14		Cytoplasmic
1084	57650569	SACOL1746	pfkA	-0.78	-0.54	-0.34	-0.70		Cytoplasmic
1085	57650570	SACOL1747	accA	-0.68	-0.47	-0.58			Cytoplasmic
1086	57650571	SACOL1748	accD	-0.60	0.92	-0.71	-0.43		Cytoplasmic
1087	57650572	SACOL1749	-	-0.83	-0.67				Cytoplasmic
1088	57650573	SACOL1750	dnaE			0.67			Cytoplasmic
1089	57650574	SACOL1751	-	-0.05	-0.15				Cytoplasmic
1090	57652003	SACOL1752	-		1.88	-0.17			Cytoplasmic
1091	57652004	SACOL1753	-	1.07	1.35	1.23	1.69		Cytoplasmic
1092	57652005	SACOL1756	pepQ	0.88	0.51				Cytoplasmic
1093	57652007	SACOL1758	ald2	3.43					Cytoplasmic
1094	57652008	SACOL1759	-	1.73	1.33	0.79	1.93		Cytoplasmic
1095	57652009	SACOL1760	ackA	-0.31	0.03	-0.24	-0.11		Cytoplasmic
1096	57652010	SACOL1761	-	-0.74		-0.18			Cytoplasmic
1097	57652011	SACOL1762	-	0.28	0.40		0.67	0.31	Cytoplasmic
1098	57652012	SACOL1763	-			0.55			Integral membrane
1099	57652013	SACOL1764	thiI	-1.66	0.44	-1.41			Cytoplasmic
1100	57652014	SACOL1765	-	-1.48		-1.74			Cytoplasmic
1101	57652016	SACOL1767	-	0.45		-0.05			Integral membrane
1102	57652017	SACOL1768	-	n. qu.	n. qu.	n. qu.	n. qu.	n. qu.	Cytoplasmic
1103	57652018	SACOL1769	rpsD	-2.12	-1.13	-1.93	-1.66		Cytoplasmic
1104	57652019	SACOL1770	-	1.86	0.43	2.31			Cytoplasmic
1105	57652020	SACOL1771	-	n. qu.	n. qu.	n. qu.	n. qu.	n. qu.	Cytoplasmic
1106	57652021	SACOL1772	-	0.40	1.51	-0.95		0.93	Cytoplasmic
1107	57652022	SACOL1773	serA	0.54	0.85	0.62	0.71		Cytoplasmic
1108	57652023	SACOL1774	-	0.40		0.56			Cytoplasmic
1109	57652024	SACOL1775	-			-0.43			Integral membrane
1110	57652025	SACOL1776	-			-0.98			Cytoplasmic
1111	57652026	SACOL1777	-			-0.14	-0.46	2.43	Integral membrane
1112	57652027	SACOL1778	tyrS	-0.55	-0.20	-0.66			Cytoplasmic
1113	57652028	SACOL1779	-	n. qu.	n. qu.	n. qu.	n. qu.	n. qu.	Integral membrane
1114	57652030	SACOL1782	fhs	1.88	1.85		2.44		Cytoplasmic
1115	57652031	SACOL1783	acs	5.37	0.71				Integral membrane
1116	57652032	SACOL1784	acuA			on in stat			Cytoplasmic
1117	57652033	SACOL1785	acuC	n. qu.	n. qu.	n. qu.	n. qu.	n. qu.	Cytoplasmic
1118	57652034	SACOL1786	ccpA	0.99	1.47	0.86	1.15		Cytoplasmic
1119	57652035	SACOL1787	-	-0.30	0.30		-0.06		Cytoplasmic
1120	57652036	SACOL1788	-	0.57		0.35			Cell wall associated
1121	57652037	SACOL1789	-	0.00		0.15	0.03	-0.66	Integral membrane
1122	57652038	SACOL1790	murC	-0.50	-0.31	-0.41	-0.12		Cytoplasmic
1123	57652039	SACOL1791	-			0.00			Cytoplasmic
1124	57652040	SACOL1792	-	-0.20	-0.45		0.10		Cytoplasmic
1125	57652041	SACOL1793	-	-0.14	0.20	-0.08			Cytoplasmic
1126	57652042	SACOL1794	-	0.22					Cytoplasmic
1127	57652043	SACOL1795	pepA1	0.80	0.68	0.88		1.68	Cytoplasmic
1128	57652044	SACOL1796	-			-0.16			Integral membrane
1129	57652045	SACOL1797	-	1.58					Cytoplasmic
1130	57652046	SACOL1798	trmB	-1.51	0.38	-1.12			Cytoplasmic
1131	57652047	SACOL1799	-	1.90		2.11			Cytoplasmic
1132	57652048	SACOL1800	dat	0.56	0.23		0.29	0.36	Cytoplasmic
1133	57652049	SACOL1801	-	0.07	0.13	-0.05	-0.06		Cytoplasmic
1134	57652050	SACOL1802	-	-0.10		0.41		1.06	Signal peptide
1135	57652052	SACOL1804	-			0.31			Integral membrane
1136	57652053	SACOL1805	-		-0.28				Cytoplasmic
1137	57652055	SACOL1807	-	-0.17			0.04		Cytoplasmic
1138	57652056	SACOL1808	leuS	-0.50	-0.64				Cytoplasmic
1139	57652057	SACOL1809	-	n. qu.	n. qu.	n. qu.	n. qu.	n. qu.	Integral membrane
1140	57652058	SACOL1810	-	n. qu.	n. qu.	n. qu.	n. qu.	n. qu.	Cytoplasmic
1141	57652059	SACOL1811	-	n. qu.	n. qu.	n. qu.	n. qu.	n. qu.	Cytoplasmic
1142	57652060	SACOL1812	rot	1.07		1.31			Integral membrane
1143	57652061	SACOL1814	-	0.93		0.74			Cytoplasmic
1144	57652063	SACOL1816	putA		2.15	3.83			Integral membrane
1145	57652064	SACOL1817	ribH	-0.37	-0.65	-1.34	0.21		Cytoplasmic
1146	57652065	SACOL1818	ribBA	-4.53					Cytoplasmic
1147	57652066	SACOL1819	ribE	-1.12	-0.37				Cytoplasmic
1148	57652067	SACOL1820	ribD	-0.39	-0.15				Cytoplasmic
1149	57652068	SACOL1821	-	n. qu.	n. qu.	n. qu.	n. qu.	n. qu.	Cytoplasmic
1150	57652071	SACOL1824	arsC	n. qu.	n. qu.	n. qu.	n. qu.	n. qu.	Cytoplasmic
1151	57652072	SACOL1825	-	-0.99		-0.87	-1.48		Cell wall associated
1152	57652076	SACOL1829	-			0.31			Integral membrane

xyz - quantified with significant quantitative value, no change in protein amount
xyz - quantified with significant quantitative value, down-regulated/degraded in stat. phase
xyz - quantified with significant quantitative value, up-regulated in stat. phase
xyz - quantified in one biological replicate only (therefore not considered as reliable)
n.qu. - identified but not quantified

Continued Supplementary Table 2:

	gi accession	SACOL	gene	Cytosolic GeLC-MS/MS	Cytosolic 2D-PAGE	Membrane GeLC-MS/MS	Surface GeLC-MS/MS	Extracellular GeLC-MS/MS	Localization
1153	57652078	SACOL1831	tal	0.89	0.76		1.30	0.63	Cytoplasmic
1154	57652081	SACOL1835	-	1.29	0.27				Cytoplasmic
1155	57652082	SACOL1836	-	0.03		-0.09	0.76		Cell wall associated
1156	57650575	SACOL1837	metK	-0.44	0.13	-0.59	-0.02		Cytoplasmic
1157	57650576	SACOL1838	pckA	3.76	1.87			2.68	Cytoplasmic
1158	57650577	SACOL1839	-	n. qu.	n. qu.	n. qu.	n. qu.	n. qu.	Cytoplasmic
1159	57650578	SACOL1840	-		2.53				Cytoplasmic
1160	57650579	SACOL1841	-	-3.23					Cytoplasmic
1161	57650580	SACOL1842	-	n. qu.	n. qu.	n. qu.	n. qu.	n. qu.	Cytoplasmic
1162	57650581	SACOL1843	-			0.40			Cytoplasmic
1163	57650585	SACOL1847	-			3.38			Cell wall associated
1164	57650586	SACOL1848	-	n. qu.	n. qu.	n. qu.	n. qu.	n. qu.	Lipo
1165	57650596	SACOL1861	hsdS	-0.28					Cytoplasmic
1166	57650597	SACOL1862	hsdM2		-0.68				Cytoplasmic
1167	57650602	SACOL1867	splC	n. qu.	n. qu.	n. qu.	n. qu.	n. qu.	Signal peptide
1168	57650603	SACOL1868	splB					1.64	Signal peptide
1169	57650605	SACOL1870	-					3.32	Signal peptide
1170	57650608	SACOL1873	epiF	4.75	3.58	4.47			Cytoplasmic
1171	57650609	SACOL1874	epiP					2.88	Signal peptide
1172	57650614	SACOL1880	lukD					-0.68	Signal peptide
1173	57650617	SACOL1883	-	n. qu.	n. qu.	n. qu.	n. qu.	n. qu.	Cytoplasmic
1174	57650618	SACOL1885	-	-0.09					Cytoplasmic
1175	57650620	SACOL1887	hemG		1.64	0.08			Cytoplasmic
1176	57650621	SACOL1888	hemH	0.13					Cytoplasmic
1177	57650622	SACOL1889	hemE	-0.44		-0.23			Cytoplasmic
1178	57650624	SACOL1891	-	1.23	0.78			0.48	Cytoplasmic
1179	57650625	SACOL1892	-			-0.03			Integral membrane
1180	57650626	SACOL1893	-			-0.07			Cytoplasmic
1181	57650627	SACOL1894	-		0.91				Cytoplasmic
1182	57650628	SACOL1895	-	-0.69		-0.55			Cell wall associated
1183	57650630	SACOL1897	-	0.27		-0.51	-0.09	3.06	Lipo
1184	57650631	SACOL1898	cbf1	0.36	0.66	0.65			Cytoplasmic
1185	57650632	SACOL1899	-	1.50		0.49	0.75		Integral membrane
1186	57650633	SACOL1900	-	0.78		0.29			Cytoplasmic
1187	57650634	SACOL1902	-	0.04	-0.23				Cytoplasmic
1188	57650635	SACOL1903	-			-0.56			Integral membrane
1189	57650636	SACOL1904	-	-4.78	-2.70	off in stat	-4.56		Cytoplasmic
1190	57650637	SACOL1905	-	-0.15					Cytoplasmic
1191	57650638	SACOL1906	-	n. qu.	n. qu.	n. qu.	n. qu.	n. qu.	Cytoplasmic
1192	57650639	SACOL1907	-	0.57	2.16	1.29			Cytoplasmic
1193	57650640	SACOL1908	fumC	2.64	1.90	2.27			Cytoplasmic
1194	57650641	SACOL1909	-	n. qu.	n. qu.	n. qu.	n. qu.	n. qu.	Integral membrane
1195	57650644	SACOL1912	-	0.92	0.16	0.26	0.66	0.95	Cytoplasmic
1196	57650645	SACOL1913	-	-0.55					Cytoplasmic
1197	57650648	SACOL1916	-	n. qu.	n. qu.	n. qu.	n. qu.	n. qu.	Integral membrane
1198	57650649	SACOL1917	-	n. qu.	n. qu.	n. qu.	n. qu.	n. qu.	Integral membrane
1199	57650651	SACOL1919	-	-0.83					Cytoplasmic
1200	57650652	SACOL1920	-		1.05	1.59			Cytoplasmic
1201	57650653	SACOL1921	bcp	0.79	0.50		1.26		Cytoplasmic
1202	57652083	SACOL1922	hemL2	-0.12	0.00	-0.79			Cytoplasmic
1203	57652084	SACOL1923	-	n. qu.	n. qu.	n. qu.	n. qu.	n. qu.	Integral membrane
1204	57652085	SACOL1924	-			-0.16			Integral membrane
1205	57652086	SACOL1925	-	0.11					Cytoplasmic
1206	57652088	SACOL1927	-			0.75			Integral membrane
1207	57652089	SACOL1928	-	n. qu.	n. qu.	n. qu.	n. qu.	n. qu.	Integral membrane
1208	57652090	SACOL1929	-			-0.36			Integral membrane
1209	57652091	SACOL1930	-	-1.48					Cytoplasmic
1210	57652092	SACOL1931	-		1.52				Cytoplasmic
1211	57652093	SACOL1932	-			0.37			Integral membrane
1212	57652094	SACOL1933	-	1.52	1.20		2.00		Cytoplasmic
1213	57652097	SACOL1936	-	-0.20	-0.39				Cytoplasmic
1214	57652098	SACOL1937	pepS	0.27	0.39			-0.98	Cytoplasmic
1215	57652100	SACOL1939	-	0.01					Cytoplasmic
1216	57652101	SACOL1940	-			-2.42			Cell wall associated
1217	57652102	SACOL1941	-			-0.28	-0.74		Integral membrane
1218	57652103	SACOL1942	vraR	0.28	-0.05	0.18	0.26		Cytoplasmic
1219	57652104	SACOL1943	vraS			0.01			Integral membrane
1220	57652105	SACOL1944	-			0.21			Integral membrane
1221	57652107	SACOL1946	-	-0.13			0.20		Cytoplasmic
1222	57652108	SACOL1947	-	n. qu.	n. qu.	n. qu.	n. qu.	n. qu.	Integral membrane
1223	57652111	SACOL1950	-	0.00					Cytoplasmic
1224	57652112	SACOL1951	-	-0.21		0.00			Cytoplasmic

xyz - quantified with significant quantitative value, no change in protein amount
xyz - quantified with significant quantitative value, down-regulated/degraded in stat. phase
xyz - quantified with significant quantitative value, up-regulated in stat. phase
xyz - quantified in one biological replicate only (therefore not considered as reliable)
n.qu. - identified but not quantified

Continued Supplementary Table 2:

	gi accession	SACOL	gene	Cytosolic GeLC-MS/MS	Cytosolic 2D-PAGE	Membrane GeLC-MS/MS	Surface GeLC-MS/MS	Extracellular GeLC-MS/MS	Localization
1225	57652113	SACOL1952	-	1.38	1.28	1.69	0.72		Cytoplasmic
1226	57652118	SACOL1957	-	n. qu.	n. qu.	n. qu.	n. qu.	n. qu.	Cytoplasmic
1227	57652119	SACOL1958	-	0.27		0.25			Cytoplasmic
1228	57652120	SACOL1960	gatB	-0.32		-0.33	-0.17		Cytoplasmic
1229	57652121	SACOL1961	gatA	-0.30	-0.22	-0.26	-0.13		Cytoplasmic
1230	57652122	SACOL1962	gatC	-0.36					Cytoplasmic
1231	57652123	SACOL1963	-			2.17			Integral membrane
1232	57650654	SACOL1964	camS			-0.32	0.05	2.23	Lipo
1233	57650655	SACOL1965	ligA	-0.64	-1.04	-0.30			Cytoplasmic
1234	57650656	SACOL1966	pcrA	-0.87	-0.46	-1.05			Cytoplasmic
1235	57650657	SACOL1967	-	n. qu.	n. qu.	n. qu.	n. qu.	n. qu.	Cytoplasmic
1236	57650658	SACOL1968	-	0.10					Cytoplasmic
1237	57650659	SACOL1969	purB	-0.14	0.61	-1.06	-0.36		Cytoplasmic
1238	57650660	SACOL1970	sspB2					-1.80	Signal peptide
1239	57650663	SACOL1973	-			0.33			Integral membrane
1240	57650664	SACOL1974	nadE	0.61	0.48				Cytoplasmic
1241	57650665	SACOL1975	-	0.38	-1.18				Cytoplasmic
1242	57650666	SACOL1976	-	-0.12					Cytoplasmic
1243	57650669	SACOL1979	-			4.75			Integral membrane
1244	57650670	SACOL1980	-		2.02	2.10			Cytoplasmic
1245	57650672	SACOL1982	ppaC	-0.15	-0.64	0.11	0.04		Cytoplasmic
1246	57650674	SACOL1984	aldA2	0.89	2.46	0.83			Cytoplasmic
1247	57650675	SACOL1985	-	0.10	-0.08				Cytoplasmic
1248	57650677	SACOL1987	-			0.66			Signal peptide
1249	57650678	SACOL1988	-	1.68					Cytoplasmic
1250	57650679	SACOL1989	-			0.02	-0.19		Cell wall associated
1251	57650680	SACOL1990	-			-1.07			Integral membrane
1252	57650681	SACOL1991	-			-0.45			Integral membrane
1253	57650682	SACOL1992	-	1.56	0.94	on in stat			Cytoplasmic
1254	57650683	SACOL1993	-			0.18			Integral membrane
1255	57650684	SACOL1994	-		1.31	-0.21			Cytoplasmic
1256	57650685	SACOL1995	-	n. qu.	n. qu.	n. qu.	n. qu.	n. qu.	Integral membrane
1257	57650686	SACOL1996	-			-0.56			Cytoplasmic
1258	57650690	SACOL2000	-	0.54	0.15	-0.06	0.10		Cytoplasmic
1259	57650691	SACOL2002	map					2.19	Cell wall associated
1260	57650692	SACOL2003	hlb					1.51	Signal peptide
1261	57650693	SACOL2004	-	n. qu.	n. qu.	n. qu.	n. qu.	n. qu.	Cytoplasmic
1262	57650694	SACOL2006	-	n. qu.	n. qu.	n. qu.	n. qu.	n. qu.	Lipo
1263	57650696	SACOL2010	-			-1.07			Lipo
1264	57650698	SACOL2012	-			1.29			Cytoplasmic
1265	57650701	SACOL2016	groEL	0.11	-0.23	0.43	0.11	-0.92	Cytoplasmic
1266	57650702	SACOL2017	groES	-0.24	-0.25			-0.85	Cytoplasmic
1267	57650703	SACOL2018	-			0.90			Integral membrane
1268	57650704	SACOL2019	-					1.47	Cell wall associated
1269	57650705	SACOL2020	-	0.82	0.16				Cytoplasmic
1270	57650706	SACOL2021	-	1.25					Cytoplasmic
1271	57650710	SACOL2025	argC2			1.09			Integral membrane
1272	57650711	SACOL2026	agrA			1.64			Cytoplasmic
1273	57650712	SACOL2028	-		0.35	0.60			Cytoplasmic
1274	57650713	SACOL2029	cscA	0.33	1.45				Cytoplasmic
1275	57650718	SACOL2035	-	-0.06	-0.26	-0.15	-0.17		Cytoplasmic
1276	57650719	SACOL2036	-			0.05			Cytoplasmic
1277	57650720	SACOL2037	-			-0.07			Integral membrane
1278	57650721	SACOL2038	-		0.80	-0.55			Cytoplasmic
1279	57650724	SACOL2041	-	-0.49					Cytoplasmic
1280	57650725	SACOL2042	ilvD	-1.62	-1.01	-0.96	-1.22		Cytoplasmic
1281	57650726	SACOL2043	ilvB	-2.31	-1.61	-2.07			Cytoplasmic
1282	57650727	SACOL2044	-	n. qu.	n. qu.	n. qu.	n. qu.	n. qu.	Cytoplasmic
1283	57650728	SACOL2045	ilvC	-1.16	-1.07	-1.63	-0.79	-2.31	Cytoplasmic
1284	57650729	SACOL2046	leuA	-1.62	-1.41	-0.94	-1.39	-2.21	Cytoplasmic
1285	57650730	SACOL2047	leuB	-1.40	-0.85	-1.43	-1.09		Cytoplasmic
1286	57650731	SACOL2048	leuC	-2.55		-2.32	-1.84		Cytoplasmic
1287	57650732	SACOL2049	leuD	-2.67	-2.30	-2.47	-2.00		Cytoplasmic
1288	57650733	SACOL2050	ilvA2	-1.90	-3.36	-1.59	-1.29	-1.95	Cytoplasmic
1289	57650734	SACOL2052	-	n. qu.	n. qu.	n. qu.	n. qu.	n. qu.	Cytoplasmic
1290	57650735	SACOL2053	-	-1.83	-0.57	-1.50	-1.53		Cytoplasmic
1291	57650736	SACOL2054	rpoF	-1.76	-1.29				Cytoplasmic
1292	57650737	SACOL2055	rsbW	-0.24	-1.12	0.08	0.25		Cytoplasmic
1293	57650738	SACOL2056	rsbV	-0.09					Cytoplasmic
1294	57650739	SACOL2057	rsbU	0.87	0.93	0.91	1.38		Cytoplasmic
1295	57650740	SACOL2058	-	-0.25					Cytoplasmic
1296	57650742	SACOL2060	alr	0.64	1.61				Cytoplasmic

xyz - quantified with significant quantitative value, no change in protein amount
xyz - quantified with significant quantitative value, down-regulated/degraded in stat. phase
xyz - quantified with significant quantitative value, up-regulated in stat. phase
xyz - quantified in one biological replicate only (therefore not considered as reliable)
n.qu. - identified but not quantified

Continued Supplementary Table 2:

	gi accession	SACOL	gene	Cytosolic GeLC-MS/MS	Cytosolic 2D-PAGE	Membrane GeLC-MS/MS	Surface GeLC-MS/MS	Extracellular GeLC-MS/MS	Localization
1297	57650743	SACOL2061	acpS	-0.47					Cytoplasmic
1298	57650744	SACOL2062	-			0.74			Integral membrane
1299	57650745	SACOL2063	-			0.93			Integral membrane
1300	57650746	SACOL2064	-	n. qu.	n. qu.	n. qu.	n. qu.	n. qu.	Integral membrane
1301	57650754	SACOL2072	-	-1.92		-1.93			Cytoplasmic
1302	57650755	SACOL2073	murF	-0.53					Cytoplasmic
1303	57650756	SACOL2074	ddl	-0.12	-0.20		0.34		Cytoplasmic
1304	57650757	SACOL2075	ftsW			0.11			Integral membrane
1305	57650760	SACOL2078	-	n. qu.	n. qu.	n. qu.	n. qu.	n. qu.	Cytoplasmic
1306	57650761	SACOL2079	cls2			0.55			Integral membrane
1307	57650762	SACOL2080	-	0.15					Cytoplasmic
1308	57650764	SACOL2082	-			-0.60			Lipo
1309	57650765	SACOL2083	thiE	2.22					Cytoplasmic
1310	57650766	SACOL2084	thiM	2.62					Cytoplasmic
1311	57650767	SACOL2085	thiD2	n. qu.	n. qu.	n. qu.	n. qu.	n. qu.	Cytoplasmic
1312	57650768	SACOL2086	-	2.67		2.86			Cytoplasmic
1313	57650769	SACOL2088	-				0.17	-0.72	Signal peptide
1314	57650771	SACOL2090	ywpF	1.59		1.24			Cytoplasmic
1315	57650772	SACOL2091	fabZ	-0.26	0.13	-0.86	0.42		Cytoplasmic
1316	57650773	SACOL2092	murAA	-0.67	-0.12	-0.32	-0.22		Cytoplasmic
1317	57652124	SACOL2094	atpC	0.18					Cytoplasmic
1318	57652125	SACOL2095	atpD	0.14	0.00	-0.01	0.38	-0.80	Cytoplasmic
1319	57652126	SACOL2096	atpG	0.07	-0.16	-0.09	0.37		Cytoplasmic
1320	57652127	SACOL2097	atpA		0.08	-0.27	0.35	-0.85	Cytoplasmic
1321	57652128	SACOL2098	atpH	0.09	0.04	-0.25	0.26		Cytoplasmic
1322	57652129	SACOL2099	atpF	0.28		0.11	-0.05		Integral membrane
1323	57652130	SACOL2100	atpE	n. qu.	n. qu.	n. qu.	n. qu.	n. qu.	Integral membrane
1324	57652131	SACOL2101	atpB			0.97			Integral membrane
1325	57652133	SACOL2103	-	0.38	0.65	0.26			Cytoplasmic
1326	57652134	SACOL2104	upp	0.29	0.54	0.34	0.54		Cytoplasmic
1327	57652135	SACOL2105	glyA	0.02	-0.08	-0.54	0.20		Cytoplasmic
1328	57652136	SACOL2106	-	2.32	1.09				Cytoplasmic
1329	57652137	SACOL2107	-			-0.66			Cytoplasmic
1330	57652138	SACOL2108	-	n. qu.	n. qu.	n. qu.	n. qu.	n. qu.	Cytoplasmic
1331	57652139	SACOL2109	-	n. qu.	n. qu.	n. qu.	n. qu.	n. qu.	Cytoplasmic
1332	57652140	SACOL2110	prfA	-0.55	-0.80			0.68	Cytoplasmic
1333	57652141	SACOL2111	tdk	-1.01					Cytoplasmic
1334	57652142	SACOL2112	rpmE	-2.17	0.23	-2.10	-2.02		Cytoplasmic
1335	57652143	SACOL2113	rho	-0.59	-0.64	-0.70	-0.50		Cytoplasmic
1336	57652144	SACOL2114	-	1.04	0.54	1.12	1.46		Cytoplasmic
1337	57652145	SACOL2115	-			off in stat			Cytoplasmic
1338	57652146	SACOL2116	murAB	-0.41	-0.24	-0.24	-0.11		Cytoplasmic
1339	57652147	SACOL2117	fbaA	-0.82	-1.21	-1.04	-0.19	-1.52	Cytoplasmic
1340	57652148	SACOL2118	-	-0.15		0.25			Cytoplasmic
1341	57652149	SACOL2119	pyrG	-0.70	-0.83	-0.43	-0.75		Cytoplasmic
1342	57652150	SACOL2120	rpoE	-0.96					Cytoplasmic
1343	57652151	SACOL2121	-	0.49					Cytoplasmic
1344	57652152	SACOL2122	-	-0.61		-0.67			Cytoplasmic
1345	57652153	SACOL2123	-	-0.50					Cytoplasmic
1346	57652154	SACOL2124	-	-0.07					Cytoplasmic
1347	57652155	SACOL2125	-	0.17	-0.35				Cytoplasmic
1348	57652156	SACOL2126	luxS	-0.13	0.04		-0.12		Cytoplasmic
1349	57652157	SACOL2127	-	n. qu.	n. qu.	n. qu.	n. qu.	n. qu.	Integral membrane
1350	57652158	SACOL2128	pdp	3.06					Cytoplasmic
1351	57652159	SACOL2129	deoC2	3.06	0.06				Cytoplasmic
1352	57652160	SACOL2130	deoD2	0.21	-0.19			0.12	Cytoplasmic
1353	57652161	SACOL2131	-	0.42	-0.13	0.27	0.57		Cytoplasmic
1354	57652162	SACOL2132	-			1.20			Cytoplasmic
1355	57652163	SACOL2133	-	1.38	0.90	0.67			Cytoplasmic
1356	57652165	SACOL2135	manA1		-0.51				Cytoplasmic
1357	57652166	SACOL2136	-	1.02	1.11				Cytoplasmic
1358	57652170	SACOL2142	-	n. qu.	n. qu.	n. qu.	n. qu.	n. qu.	Cytoplasmic
1359	57652172	SACOL2144	-	1.47	2.11	1.55			Cytoplasmic
1360	57652173	SACOL2145	glmS	0.81	0.93	1.25	1.00		Cytoplasmic
1361	57652174	SACOL2146	-	n. qu.	n. qu.	n. qu.	n. qu.	n. qu.	Integral membrane
1362	57652175	SACOL2147	-			0.81			Cytoplasmic
1363	57652176	SACOL2148	-	1.16	0.66				Cytoplasmic
1364	57652177	SACOL2149	mtlD	1.12	1.24				Cytoplasmic
1365	57652179	SACOL2151	glmM	0.33	0.89	0.58	0.72		Cytoplasmic
1366	57652180	SACOL2152	-			0.28	-0.15	2.05	Signal peptide
1367	57652181	SACOL2153	-			0.24	0.09		Integral membrane
1368	57652182	SACOL2154	rocF	4.02	2.15	on in stat	0.68		Cytoplasmic

xyz - quantified with significant quantitative value, no change in protein amount
xyz - quantified with significant quantitative value, down-regulated/degraded in stat. phase
xyz - quantified with significant quantitative value, up-regulated in stat. phase
xyz - quantified in one biological replicate only (therefore not considered as reliable)
n.qu. - identified but not quantified

Continued Supplementary Table 2:

	gi accession	SACOL	gene	Cytosolic GeLC-MS/MS	Cytosolic 2D-PAGE	Membrane GeLC-MS/MS	Surface GeLC-MS/MS	Extracellular GeLC-MS/MS	Localization
1369	57652183	SACOL2156	-	0.57	1.18	0.62	0.87		Cytoplasmic
1370	57652184	SACOL2157	-	n. qu.	n. qu.	n. qu.	n. qu.	n. qu.	Integral membrane
1371	57652186	SACOL2159	-			0.30			Integral membrane
1372	57652187	SACOL2160	-	n. qu.	n. qu.	n. qu.	n. qu.	n. qu.	Integral membrane
1373	57652188	SACOL2161	-	-0.10	0.37	-0.06	-0.19		Cytoplasmic
1374	57652190	SACOL2163	-	2.78	2.98		4.15	3.12	Cytoplasmic
1375	57652191	SACOL2164	-	n. qu.	n. qu.	n. qu.	n. qu.	n. qu.	Integral membrane
1376	57652192	SACOL2165	-	n. qu.	n. qu.	n. qu.	n. qu.	n. qu.	Integral membrane
1377	57652193	SACOL2166	-	n. qu.	n. qu.	n. qu.	n. qu.	n. qu.	Integral membrane
1378	57652194	SACOL2167	-	-1.15		-1.33	-1.39	0.25	Lipo
1379	57652196	SACOL2169	-	-0.15		0.21			Cytoplasmic
1380	57652198	SACOL2171	-	0.11	0.31				Cytoplasmic
1381	57652199	SACOL2173	-	1.20	0.94	0.82	1.22		Cytoplasmic
1382	57652200	SACOL2174	-			1.62			Integral membrane
1383	57652201	SACOL2175	-	1.50		1.55	1.63		Integral membrane
1384	57652202	SACOL2176	opuD2			1.83			Integral membrane
1385	57652203	SACOL2177	-	1.06					Cytoplasmic
1386	57652204	SACOL2178	-	1.89					Cytoplasmic
1387	57650775	SACOL2179	-			-1.19	-0.79		Signal peptide
1388	57650777	SACOL2181	lacE	n. qu.	n. qu.	n. qu.	n. qu.	n. qu.	Integral membrane
1389	57650779	SACOL2183	lacD	n. qu.	n. qu.	n. qu.	n. qu.		Cytoplasmic
1390	57650784	SACOL2188	lacR			1.10			Cytoplasmic
1391	57650785	SACOL2189	-	1.14					Cytoplasmic
1392	57650788	SACOL2192	-	2.02	-0.27	2.12			Cytoplasmic
1393	57650790	SACOL2194	hysA					-2.92	Signal peptide
1394	57650792	SACOL2196	-	0.96	1.30				Cytoplasmic
1395	57650793	SACOL2197	-				5.93	4.25	Signal peptide
1396	57650794	SACOL2198	budA1	n. qu.	n. qu.	n. qu.	n. qu.	n. qu.	Cytoplasmic
1397	57650799	SACOL2203	-			0.14			Cytoplasmic
1398	57650801	SACOL2206	rpsI		-6.96	-1.94	-1.47		Cytoplasmic
1399	57650802	SACOL2207	rplM		-1.30	-1.89	-1.37	-0.06	Cytoplasmic
1400	57650803	SACOL2208	truA	-0.32					Cytoplasmic
1401	57650804	SACOL2209	-	n. qu.	n. qu.	n. qu.	n. qu.	n. qu.	Integral membrane
1402	57650805	SACOL2210	-			0.08			Cytoplasmic
1403	57650806	SACOL2211	-	n. qu.	n. qu.	n. qu.	n. qu.	n. qu.	Cytoplasmic
1404	57650807	SACOL2212	rplQ	-1.62	-0.70	-1.34	-1.26	-0.19	Cytoplasmic
1405	57650808	SACOL2213	rpoA	-0.92	-1.15	-1.03	-0.67	-1.25	Cytoplasmic
1406	57650809	SACOL2214	rpsK	-2.38		-1.42	-1.40		Cytoplasmic
1407	57650810	SACOL2215	rpsM	-1.94		-1.38	-1.21		Cytoplasmic
1408	57650812	SACOL2217	infA	-1.01					Cytoplasmic
1409	57650813	SACOL2218	adk	-0.93	-0.98				Cytoplasmic
1410	57650814	SACOL2219	secY			-0.02			Integral membrane
1411	57650815	SACOL2220	rplO	-2.05		-1.99			Cytoplasmic
1412	57650816	SACOL2221	rpmD	-1.83					Cytoplasmic
1413	57650817	SACOL2222	rpsE	-2.77	-2.06	-1.83	-1.53		Cytoplasmic
1414	57650818	SACOL2223	rplR	-2.04	-1.61				Cytoplasmic
1415	57650819	SACOL2224	rplF	-2.00	-1.34		-1.61	-1.61	Cytoplasmic
1416	57650820	SACOL2225	rpsH	-1.99	-1.29	-1.34	-1.73		Cytoplasmic
1417	57650822	SACOL2227	rplE	-1.98	-1.21	-1.81	-1.38	-1.41	Cytoplasmic
1418	57650823	SACOL2228	rplX	-1.89				0.10	Cytoplasmic
1419	57650824	SACOL2229	rplN	-1.87	-1.50	-1.45	-1.27		Cytoplasmic
1420	57650825	SACOL2230	rpsQ	-2.63	-1.80				Cytoplasmic
1421	57650826	SACOL2231	rpmC	-1.95	0.56		-1.48		Cytoplasmic
1422	57650827	SACOL2232	rplP	-1.89		-1.90			Cytoplasmic
1423	57650828	SACOL2233	rpsC	-2.05	-1.76	-1.67	-1.60		Cytoplasmic
1424	57650829	SACOL2234	rplV	-2.11	-2.05	-2.04	-1.55		Signal peptide
1425	57650830	SACOL2235	rpsS	-2.08			-1.45		Cytoplasmic
1426	57650831	SACOL2236	rplB	-2.07		-1.83			Cytoplasmic
1427	57650832	SACOL2237	rplW	-2.00			-1.72		Cytoplasmic
1428	57650833	SACOL2238	rplD	-1.76		-1.90	-1.44		Cytoplasmic
1429	57650834	SACOL2239	rplC	-1.84	-1.70	-1.72	-1.74	-1.29	Cytoplasmic
1430	57650835	SACOL2240	rpsJ	-2.12	-1.35	-1.63	-1.67		Cytoplasmic
1431	57650837	SACOL2242	-			-0.65			Integral membrane
1432	57650838	SACOL2243	topB			-1.05			Cytoplasmic
1433	57650839	SACOL2245	-	1.74					Cytoplasmic
1434	57650840	SACOL2246	-	n. qu.	n. qu.	n. qu.	n. qu.	n. qu.	Integral membrane
1435	57650845	SACOL2252	-			1.10	0.65		Integral membrane
1436	57650846	SACOL2253	femX	-1.14	0.81	-0.62			Cytoplasmic
1437	57650847	SACOL2255	-	1.58	1.24	1.49			Cytoplasmic
1438	57650849	SACOL2257	-	n. qu.	n. qu.	n. qu.	n. qu.	n. qu.	Integral membrane
1439	57650853	SACOL2261	moaA	n. qu.	n. qu.	n. qu.	n. qu.	n. qu.	Cytoplasmic
1440	57650854	SACOL2262	mobA	-0.63		-0.18			Cytoplasmic

xyz - quantified with significant quantitative value, no change in protein amount
xyz - quantified with significant quantitative value, down-regulated/degraded in stat. phase
xyz - quantified with significant quantitative value, up-regulated in stat. phase
xyz - quantified in one biological replicate only (therefore not considered as reliable)
n.qu. - identified but not quantified

Continued Supplementary Table 2:

	gi accession	SACOL	gene	Cytosolic GeLC-MS/MS	Cytosolic 2D-PAGE	Membrane GeLC-MS/MS	Surface GeLC-MS/MS	Extracellular GeLC-MS/MS	Localization
1441	57650856	SACOL2264	moaE	-0.75	-0.20				Cytoplasmic
1442	57652205	SACOL2265	mobB	-0.21					Cytoplasmic
1443	57652206	SACOL2266	-	0.22	0.46	0.77			Cytoplasmic
1444	57652207	SACOL2267	moaC	0.57					Cytoplasmic
1445	57652208	SACOL2268	moaB	-0.03	-0.83	1.71	-0.02		Cytoplasmic
1446	57652209	SACOL2269	-	-0.25		0.07			Cytoplasmic
1447	57652210	SACOL2270	modC			-0.95			Cytoplasmic
1448	57652212	SACOL2272	modA	0.76		0.65	0.04		Lipo
1449	57652213	SACOL2273	fdhD	0.71	0.90				Cytoplasmic
1450	57652214	SACOL2274	-	0.49					Cytoplasmic
1451	57652215	SACOL2275	-	n. qu.	n. qu.	n. qu.	n. qu.	n. qu.	Integral membrane
1452	57652216	SACOL2276	-	n. qu.	n. qu.	n. qu.	n. qu.	n. qu.	Cytoplasmic
1453	57652217	SACOL2277	-	-1.28		-1.29	-1.69	-0.16	Lipo
1454	57652218	SACOL2278	-	0.71					Cytoplasmic
1455	57652220	SACOL2280	ureA	n. qu.	n. qu.	n. qu.	n. qu.	n. qu.	Cytoplasmic
1456	57652221	SACOL2281	ureB	-1.14					Cytoplasmic
1457	57652222	SACOL2282	ureC	-0.82	-2.89				Cytoplasmic
1458	57652223	SACOL2283	ureE	-0.50	-0.63				Cytoplasmic
1459	57652224	SACOL2284	ureF			-1.23			Cytoplasmic
1460	57652225	SACOL2285	ureG	-0.21			-0.45		Cytoplasmic
1461	57652227	SACOL2287	sarR	-0.91	-1.22	-1.17	-0.65		Cytoplasmic
1462	57652228	SACOL2288	-	0.71					Cytoplasmic
1463	57652231	SACOL2291	-	-4.91	-3.54	-3.62		-3.26	Lipo
1464	57652232	SACOL2292	nhaC	n. qu.	n. qu.	n. qu.	n. qu.	n. qu.	Integral membrane
1465	57652233	SACOL2293	-	0.53	0.76	0.54			Cytoplasmic
1466	57652236	SACOL2296	-	2.23	1.06				Cytoplasmic
1467	57652237	SACOL2297	-			-0.44	-0.16		Cytoplasmic
1468	57652240	SACOL2300	-	0.93	1.06	1.15			Cytoplasmic
1469	57652241	SACOL2301	-	1.27	1.55	1.50	1.34		Cytoplasmic
1470	57652242	SACOL2302	-	0.22		0.51	-0.30	0.61	Signal peptide
1471	57652243	SACOL2303	-	n. qu.	n. qu.	n. qu.	n. qu.	n. qu.	Cytoplasmic
1472	57652244	SACOL2304	-		4.17	1.03			Cytoplasmic
1473	57652246	SACOL2306	-			-0.53			Integral membrane
1474	57650858	SACOL2309	-			1.38			Integral membrane
1475	57650862	SACOL2313	-	0.23	0.40				Cytoplasmic
1476	57650863	SACOL2314	-	n. qu.	n. qu.	n. qu.	n. qu.	n. qu.	Integral membrane
1477	57650864	SACOL2315	-			off in stat			Cytoplasmic
1478	57650865	SACOL2316	-			on in stat			Integral membrane
1479	57650866	SACOL2317	-			0.21			Cytoplasmic
1480	57650867	SACOL2318	-	0.23					Cytoplasmic
1481	57650868	SACOL2319	-	n. qu.	n. qu.	n. qu.	n. qu.	n. qu.	Integral membrane
1482	57650870	SACOL2321	-	1.13	1.21				Cytoplasmic
1483	57650871	SACOL2322	-	1.86					Cytoplasmic
1484	57650872	SACOL2323	hutI	4.58	2.31				Cytoplasmic
1485	57650873	SACOL2324	hutU	4.81	2.45	on in stat			Cytoplasmic
1486	57650874	SACOL2325	-	n. qu.	n. qu.	n. qu.	n. qu.	n. qu.	Cytoplasmic
1487	57650876	SACOL2327	hutG	2.13	1.26		2.00	2.88	Cytoplasmic
1488	57650877	SACOL2328	-			0.04			Integral membrane
1489	57650878	SACOL2329	rpiA	0.53		0.30			Cytoplasmic
1490	57650879	SACOL2330	-	0.42					Cytoplasmic
1491	57650881	SACOL2332	galM	-0.80					Cytoplasmic
1492	57650883	SACOL2334	-			1.26			Integral membrane
1493	57650884	SACOL2335	-		0.33	1.01			Cytoplasmic
1494	57650886	SACOL2338	-			0.28			Integral membrane
1495	57650887	SACOL2339	-	n. qu.	n. qu.	n. qu.	n. qu.	n. qu.	Cytoplasmic
1496	57650888	SACOL2340	gltS			2.40			Integral membrane
1497	57650889	SACOL2341	fni	-0.30					Cytoplasmic
1498	57650890	SACOL2342	-			0.06			Integral membrane
1499	57650891	SACOL2343	-	0.26					Cytoplasmic
1500	57650892	SACOL2344	-	0.97					Cytoplasmic
1501	57650893	SACOL2345	-	-3.64	0.29	-3.50			Cytoplasmic
1502	57650894	SACOL2346	-			0.56			Integral membrane
1503	57650895	SACOL2347	-	n. qu.	n. qu.	n. qu.	n. qu.	n. qu.	Integral membrane
1504	57650896	SACOL2348	-			-1.68	-2.22		Cell wall associated
1505	57650898	SACOL2350	tcaB	n. qu.	n. qu.	n. qu.	n. qu.	n. qu.	Integral membrane
1506	57650900	SACOL2352	tcaA			-0.83			Integral membrane
1507	57650901	SACOL2353	tcaR	-0.21					Cytoplasmic
1508	57650902	SACOL2354	-	n. qu.	n. qu.	n. qu.	n. qu.	n. qu.	Integral membrane
1509	57650906	SACOL2359	-			0.39			Integral membrane
1510	57650907	SACOL2360	-	n. qu.	n. qu.	n. qu.	n. qu.	n. qu.	Cytoplasmic
1511	57650909	SACOL2362	mqo1	1.50	0.71		2.44		Cytoplasmic
1512	57650910	SACOL2363	-			-0.99			Integral membrane

xyz - quantified with significant quantitative value, no change in protein amount
xyz - quantified with significant quantitative value, down-regulated/degraded in stat. phase
xyz - quantified with significant quantitative value, up-regulated in stat. phase
xyz - quantified in one biological replicate only (therefore not considered as reliable)
n.qu. - identified but not quantified

Continued Supplementary Table 2:

	gi accession	SACOL	gene	Cytosolic GeLC-MS/MS	Cytosolic 2D-PAGE	Membrane GeLC-MS/MS	Surface GeLC-MS/MS	Extracellular GeLC-MS/MS	Localization
1513	57650911	SACOL2364	-			-0.40			Cytoplasmic
1514	57650912	SACOL2365	-			1.94	1.34		Lipo
1515	57650913	SACOL2366	-	2.05					Cytoplasmic
1516	57650914	SACOL2367	-	0.10	0.31	-0.09			Cytoplasmic
1517	57650915	SACOL2368	-	-0.82					Cytoplasmic
1518	57650916	SACOL2369	-	-0.64					Cytoplasmic
1519	57650919	SACOL2373	-			-1.29			Integral membrane
1520	57650920	SACOL2374	-			-0.47			Cytoplasmic
1521	57650921	SACOL2375	-			-0.70			Integral membrane
1522	57650922	SACOL2376	-	n. qu.	n. qu.	n. qu.	n. qu.	n. qu.	Integral membrane
1523	57650924	SACOL2378	-			-0.04			Cytoplasmic
1524	57650925	SACOL2379	-	1.35	1.39				Cytoplasmic
1525	57650927	SACOL2381	-			0.36	-0.38		Integral membrane
1526	57650928	SACOL2382	-			0.45			Integral membrane
1527	57650929	SACOL2383	-			-0.98			Cell wall associated
1528	57650930	SACOL2384	sarZ	0.89	-0.06	0.40	0.91		Cytoplasmic
1529	57650931	SACOL2385	-	0.88	-0.58				Cytoplasmic
1530	57650945	SACOL2400	-	-0.39					Cytoplasmic
1531	57650946	SACOL2401	-			-1.21			Integral membrane
1532	57650947	SACOL2402	-	0.14					Cytoplasmic
1533	57650948	SACOL2403	-			-1.11	-0.80		Lipo
1534	57650952	SACOL2407	-			0.06	-0.47		Lipo
1535	57650955	SACOL2410	-		1.43	-0.22			Cytoplasmic
1536	57650956	SACOL2411	-			-0.39			Integral membrane
1537	57650957	SACOL2412	-	-0.13		-0.01	-0.45	1.12	Lipo
1538	57650958	SACOL2413	-	n. qu.	n. qu.	n. qu.	n. qu.	n. qu.	Integral membrane
1539	57650960	SACOL2415	gpm	1.41	1.20	1.97			Cytoplasmic
1540	57650961	SACOL2416	-			1.06			Integral membrane
1541	57650962	SACOL2418	-			-1.91		-0.09	Signal peptide
1542	57650968	SACOL2424	bioW	n. qu.	n. qu.	n. qu.	n. qu.	n. qu.	Cytoplasmic
1543	57650969	SACOL2425	-	n. qu.	n. qu.	n. qu.	n. qu.	n. qu.	Cytoplasmic
1544	57650970	SACOL2426	bioB		0.97		2.39		Cytoplasmic
1545	57650971	SACOL2427	bioA		1.16	1.29			Cytoplasmic
1546	57650972	SACOL2428	bioD			1.20			Cytoplasmic
1547	57650977	SACOL2434	-	n. qu.	n. qu.	n. qu.	n. qu.	n. qu.	Integral membrane
1548	57650978	SACOL2435	-			0.85			Cytoplasmic
1549	57652248	SACOL2436	-			-0.27	-0.52		Integral membrane
1550	57652249	SACOL2437	bcr	n. qu.	n. qu.	n. qu.	n. qu.	n. qu.	Integral membrane
1551	57652250	SACOL2438	-	n. qu.	n. qu.	n. qu.	n. qu.	n. qu.	Cytoplasmic
1552	57652251	SACOL2439	-			-0.68			Signal peptide
1553	57652253	SACOL2441	-	n. qu.	n. qu.	n. qu.	n. qu.	n. qu.	Integral membrane
1554	57652254	SACOL2442	-			-0.24			Integral membrane
1555	57652255	SACOL2443	-			0.32			Integral membrane
1556	57652256	SACOL2445	-			-0.02			Integral membrane
1557	57652257	SACOL2446	-	-0.34					Cytoplasmic
1558	57652258	SACOL2448	-	-0.54					Cytoplasmic
1559	57652259	SACOL2449	-			0.65			Integral membrane
1560	57652260	SACOL2450	-			2.51			Integral membrane
1561	57652261	SACOL2451	-			1.31	0.83		Lipo
1562	57652262	SACOL2452	-			1.39			Integral membrane
1563	57652263	SACOL2453	-	1.43	1.76	1.70	1.94		Cytoplasmic
1564	57652265	SACOL2456	-	-0.30	0.71				Cytoplasmic
1565	57652268	SACOL2459	pnbA	1.89					Cytoplasmic
1566	57652270	SACOL2461	-			0.56			Integral membrane
1567	57652271	SACOL2462	-			-0.08			Cytoplasmic
1568	57652272	SACOL2463	pepA2	-0.28					Cytoplasmic
1569	57652273	SACOL2464	-	n. qu.	n. qu.	n. qu.	n. qu.	n. qu.	Cytoplasmic
1570	57652274	SACOL2465	-	n. qu.	n. qu.	n. qu.	n. qu.	n. qu.	Cytoplasmic
1571	57652276	SACOL2467	-	0.23		0.35	0.38		Lipo
1572	57652277	SACOL2469	-			1.24			Integral membrane
1573	57652278	SACOL2470	-			-1.17			Integral membrane
1574	57652279	SACOL2471	-	n. qu.	n. qu.	n. qu.	n. qu.	n. qu.	Integral membrane
1575	57652284	SACOL2476	-			-0.95	-1.80		Lipo
1576	57650983	SACOL2483	-			2.07			Integral membrane
1577	57650984	SACOL2484	-	1.17			1.75		Cytoplasmic
1578	57650988	SACOL2488	-	1.38	0.88				Cytoplasmic
1579	57650989	SACOL2489	-	0.10					Cytoplasmic
1580	57650997	SACOL2498	-			1.12			Lipo
1581	57650998	SACOL2499	-	0.35	0.89	0.25			Cytoplasmic
1582	57650999	SACOL2500	-	0.24					Cytoplasmic
1583	57651000	SACOL2501	-	0.78	-0.06	0.73			Integral membrane
1584	57651007	SACOL2508	galU	0.37	-0.54				Signal peptide

xyz - quantified with significant quantitative value, no change in protein amount
xyz - quantified with significant quantitative value, down-regulated/degraded in stat. phase
xyz - quantified with significant quantitative value, up-regulated in stat. phase
xyz - quantified in one biological replicate only (therefore not considered as reliable)
n.qu. - identified but not quantified

Continued Supplementary Table 2:

	gi accession	SACOL	gene	Cytosolic GeLC-MS/MS	Cytosolic 2D-PAGE	Membrane GeLC-MS/MS	Surface GeLC-MS/MS	Extracellular GeLC-MS/MS	Localization
1585	57651012	SACOL2514	gntP			1.25			Integral membrane
1586	57651013	SACOL2515	gntK	n. qu.	n. qu.	n. qu.	n. qu.	n. qu.	Cytoplasmic
1587	57651014	SACOL2516	gntR	n. qu.	n. qu.	n. qu.	n. qu.	n. qu.	Cytoplasmic
1588	57651016	SACOL2518	-	-0.28	-0.88	-0.21			Cytoplasmic
1589	57651017	SACOL2519	-	-0.57	0.54				Cytoplasmic
1590	57651018	SACOL2520	-			-0.75			Integral membrane
1591	57651019	SACOL2521	-			3.52			Integral membrane
1592	57651023	SACOL2525	-			0.76			Cytoplasmic
1593	57651024	SACOL2527	-	3.08	3.58	on in stat	3.75		Cytoplasmic
1594	57651025	SACOL2528	-	n. qu.	n. qu.	n. qu.	n. qu.	n. qu.	Integral membrane
1595	57651026	SACOL2529	-	-1.36					Cytoplasmic
1596	57651028	SACOL2531	-	0.68					Cytoplasmic
1597	57651029	SACOL2532	-	0.86					Cytoplasmic
1598	57651030	SACOL2533	-	0.80					Cytoplasmic
1599	57651031	SACOL2534	frp		1.03	2.45	1.77		Cytoplasmic
1600	57651032	SACOL2535	-	-0.22					Cytoplasmic
1601	57651033	SACOL2536	-	n. qu.	n. qu.	n. qu.	n. qu.	n. qu.	Cytoplasmic
1602	57651036	SACOL2539	srtA			0.22		2.62	Signal peptide
1603	57651044	SACOL2548	-	n. qu.	n. qu.	n. qu.	n. qu.	n. qu.	Integral membrane
1604	57651045	SACOL2549	-			0.42	0.99		Cell wall associated
1605	57651047	SACOL2551	-	0.60					Cytoplasmic
1606	57651048	SACOL2552	-			-0.02			Integral membrane
1607	57651049	SACOL2553	-	0.58	4.40	0.59	0.79		Cytoplasmic
1608	57651052	SACOL2555	-	n. qu.	n. qu.	n. qu.	n. qu.	n. qu.	Cytoplasmic
1609	57651054	SACOL2557	-					-1.84	Signal peptide
1610	57651055	SACOL2559	-	n. qu.	n. qu.	n. qu.	n. qu.	n. qu.	Cytoplasmic
1611	57651056	SACOL2560	-	0.93		1.08			Cytoplasmic
1612	57651057	SACOL2561	-	-0.39		0.23	-0.13		Cytoplasmic
1613	57651058	SACOL2562	ogt	1.62					Cytoplasmic
1614	57651059	SACOL2563	-	2.43	1.68	2.28	2.97		Cytoplasmic
1615	57652288	SACOL2566	-			0.64			Integral membrane
1616	57652291	SACOL2569	-	4.87		4.21			Cytoplasmic
1617	57652292	SACOL2570	-	n. qu.	n. qu.	n. qu.	n. qu.	n. qu.	Cytoplasmic
1618	57652294	SACOL2572	-			-0.18			Integral membrane
1619	57652296	SACOL2574	-	-0.83	-0.05	-0.91			Cytoplasmic
1620	57652297	SACOL2575	-	1.44	0.12				Cytoplasmic
1621	57652298	SACOL2576	crtN			1.05			Signal peptide
1622	57652299	SACOL2577	crtM			1.51			Cytoplasmic
1623	57652301	SACOL2579	-		2.39	1.81			Cytoplasmic
1624	57652303	SACOL2581	-					-4.24	Signal peptide
1625	57652304	SACOL2582	-			-0.52	-0.92	3.56	Integral membrane
1626	57652305	SACOL2583	-	-1.59					Cytoplasmic
1627	57652306	SACOL2584	isaA	-1.42	-1.21		-0.38		Signal peptide
1628	57652307	SACOL2585	-	n. qu.	n. qu.	n. qu.	n. qu.	n. qu.	Integral membrane
1629	57652308	SACOL2587	-	0.11		0.10			Cytoplasmic
1630	57652311	SACOL2590	-	n. qu.	n. qu.	n. qu.	n. qu.	n. qu.	Cytoplasmic
1631	57652312	SACOL2591	-	0.23					Cytoplasmic
1632	57652315	SACOL2594	-	n. qu.	n. qu.	n. qu.	n. qu.	n. qu.	Cytoplasmic
1633	57652316	SACOL2595	-		1.19				Cytoplasmic
1634	57652317	SACOL2596	-	0.88	0.94				Cytoplasmic
1635	57652318	SACOL2597	-	1.11	1.03			0.50	Cytoplasmic
1636	57652322	SACOL2601	-	0.76					Cytoplasmic
1637	57652325	SACOL2605	-	0.99					Cytoplasmic
1638	57652326	SACOL2606	pyrD		1.24	0.02			Cytoplasmic
1639	57652327	SACOL2607	-	n. qu.	n. qu.	n. qu.	n. qu.	n. qu.	Integral membrane
1640	57652328	SACOL2608	-	-1.90					Cytoplasmic
1641	57652329	SACOL2609	-	0.93	0.82				Cytoplasmic
1642	57652333	SACOL2614	panC	-0.27	0.30				Cytoplasmic
1643	57652334	SACOL2615	panB		-0.57				Cytoplasmic
1644	57652335	SACOL2616	-	0.16	-1.22	0.57			Cytoplasmic
1645	57652337	SACOL2618	ldh2	0.69	-0.08	1.03	1.07		Cytoplasmic
1646	57652338	SACOL2619	-			0.45			Integral membrane
1647	57652339	SACOL2620	-	n. qu.	n. qu.	n. qu.	n. qu.	n. qu.	Cytoplasmic
1648	57652341	SACOL2622	fdaB	0.02	-0.32		-0.02	-0.26	Cytoplasmic
1649	57652342	SACOL2623	mqo2	-0.30	1.91	0.21	0.36	-1.69	Cytoplasmic
1650	57652343	SACOL2624	-	0.40	0.39				Cytoplasmic
1651	57652344	SACOL2625	-	0.63					Cytoplasmic
1652	57652346	SACOL2627	betA		0.05	-2.65			Cytoplasmic
1653	57652347	SACOL2628	betB	-1.01					Cytoplasmic
1654	57652349	SACOL2630	-	-0.01					Cytoplasmic
1655	57652351	SACOL2632	cudT	n. qu.	n. qu.	n. qu.	n. qu.	n. qu.	Integral membrane
1656	57652354	SACOL2636	-			on in stat			Integral membrane

xyz - quantified with significant quantitative value, no change in protein amount
xyz - quantified with significant quantitative value, down-regulated/degraded in stat. phase
xyz - quantified with significant quantitative value, up-regulated in stat. phase
xyz - quantified in one biological replicate only (therefore not considered as reliable)
n.qu. - identified but not quantified

Continued Supplementary Table 2:

	gi accession	SACOL	gene	Cytosolic GeLC-MS/MS	Cytosolic 2D-PAGE	Membrane GeLC-MS/MS	Surface GeLC-MS/MS	Extracellular GeLC-MS/MS	Localization
1657	57652357	SACOL2639	cysJ	0.47	0.22				Cytoplasmic
1658	57652359	SACOL2641	gpxA2	n. qu.	n. qu.	n. qu.	n. qu.	n. qu.	Cytoplasmic
1659	57652363	SACOL2645	-			0.10			Integral membrane
1660	57652365	SACOL2647	-			0.03			Signal peptide
1661	57652367	SACOL2649	-			-2.99			Integral membrane
1662	57652368	SACOL2650	-	0.17		-0.16			Cytoplasmic
1663	57652369	SACOL2651	-	0.76					Cytoplasmic
1664	57652370	SACOL2652	clfB			off in stat	-2.47	-0.11	Sortase substrate
1665	57652377	SACOL2659	aur					-1.52	Signal peptide
1666	57652378	SACOL2660	isaB				3.43	1.33	Cell wall associated
1667	57652381	SACOL2663	-			2.41			Integral membrane
1668	57652382	SACOL2664	manA2	n. qu.	n. qu.	n. qu.	n. qu.	n. qu.	Cytoplasmic
1669	57652383	SACOL2665	-			-0.26			Integral membrane
1670	57652384	SACOL2666	-			-1.05	-1.21	-0.72	Signal peptide
1671	57652385	SACOL2667	-	0.48	-0.05	0.62			Cytoplasmic
1672	57652389	SACOL2671	-	n. qu.	n. qu.	n. qu.	n. qu.	n. qu.	Cytoplasmic
1673	57652392	SACOL2674	-	n. qu.	n. qu.	n. qu.	n. qu.	n. qu.	Cytoplasmic
1674	57652396	SACOL2678	-	3.94					Cytoplasmic
1675	57652398	SACOL2681	-			-0.46			Cytoplasmic
1676	57652399	SACOL2682	-	n. qu.	n. qu.	n. qu.	n. qu.	n. qu.	Cytoplasmic
1677	57652405	SACOL2688	icaR	1.09		1.05			Cytoplasmic
1678	57651062	SACOL2694	geh					2.74	Integral membrane
1679	57651069	SACOL2701	-	-0.69					Cytoplasmic
1680	57651072	SACOL2704	-	n. qu.	n. qu.	n. qu.	n. qu.	n. qu.	Cytoplasmic
1681	57651073	SACOL2705	-			1.26			Lipo
1682	57651074	SACOL2706	-	n. qu.	n. qu.	n. qu.	n. qu.	n. qu.	Cytoplasmic
1683	57651075	SACOL2707	-			0.57			Integral membrane
1684	57651076	SACOL2708	-		0.41	0.25	0.85		Cytoplasmic
1685	57651077	SACOL2709	-	n. qu.	n. qu.	n. qu.	n. qu.	n. qu.	Integral membrane
1686	57651078	SACOL2710	-		1.05				Cytoplasmic
1687	57651079	SACOL2711	-	1.92					Cytoplasmic
1688	57651080	SACOL2712	drp35	3.17					Cytoplasmic
1689	57651083	SACOL2715	-			-0.28			Integral membrane
1690	57651085	SACOL2717	-	1.02					Cytoplasmic
1691	57651086	SACOL2718	-			-0.42			Integral membrane
1692	57651090	SACOL2722	-	2.00	1.21				Cytoplasmic
1693	57651093	SACOL2725	-	n. qu.	n. qu.	n. qu.	n. qu.	n. qu.	Integral membrane
1694	57651096	SACOL2728	-	n. qu.	n. qu.	n. qu.	n. qu.	n. qu.	Cytoplasmic
1695	57651099	SACOL2731	-	off in stat					Cytoplasmic
1696	57651100	SACOL2732	-	n. qu.	n. qu.	n. qu.	n. qu.	n. qu.	Integral membrane
1697	57651103	SACOL2735	-	-0.25	1.79				Cytoplasmic
1698	57651104	SACOL2736	gidB			-0.77			Cytoplasmic
1699	57651105	SACOL2737	gidA	-1.18	-0.99		-0.58		Cytoplasmic
1700	57651106	SACOL2738	trmE	-0.36		-0.18			Cytoplasmic
1701	57659842	SACOLA0001	repC	-0.82		-0.68			Cytoplasmic
1702	57659841	SACOLA0002		n. qu.	n. qu.	n. qu.	n. qu.	n. qu.	Integral membrane
1703	57659840	SACOLA0003	pre	-1.15		-1.15			Cytoplasmic

xyz - quantified with significant quantitative value, no change in protein amount
xyz - quantified with significant quantitative value, down-regulated/degraded in stat. phase
xyz - quantified with significant quantitative value, up-regulated in stat. phase
xyz - quantified in one biological replicate only (therefore not considered as reliable)
n.qu. - identified but not quantified
