# Supplementary material for: A Proteomic View of an Important Human Pathogen – Towards the Quantification of the Entire Staphylococcus aureus Proteome
Source: PLoS One. 2009 Dec 4;4(12):e8176. doi: 10.1371/journal.pone.0008176 (PMC2781549; doi:10.1371/journal.pone.0008176)
Supplement: Table S3 — Oligonucleotides used in this study. (0.01 MB PDF) [file pone.0008176.s010.pdf]

Supplementary Table 3: Oligonucleotides used in this study

Gene identifier[a]	Gene name[a]	Primer	
		Direction	Sequence (5´ to 3´)[b]
SACOL0583	*rplK*	Forward	GCACATCGTGGCTAAAAAAG
		Reverse	CTAATACGACTCACTATAGGGAGATTATTCTACAACGATACCCA
SACOL0594	*tuf*	Forward	GTATCTGCTGCTGACGGTCC
		Reverse	CTAATACGACTCACTATAGGGAGAGGCATTACCATTTCAGTACC
SACOL0838	*gapA1*	Forward	CGTGCTCGTGCAGCGGCAG
		Reverse	CTAATACGACTCACTATAGGGAGATTATTTAGAAAGTTCAGCTAAG
SACOL1734	*gapA2*	Forward	GATTTAAGACGTGCACGTTCATG
		Reverse	CTAATACGACTCACTATAGGGAGATTAACTTGCACTTACAGTTTC
SACOL1838	*pckA*	Forward	ATGTCAGTAGACACATACAC
		Reverse	CTAATACGACTCACTATAGGGAGAATTGCACCATATTTGATTGC

[a] Based on TIGR annotation (http://www.tigr.org).

[b] The underlined nucleotides at the 5´ end represent the binding site for the T7 RNA polymerase
